# Synthesis and Biological Evaluation of New Chalcogen Semicarbazone (*S*, *Se*) and Their Azole Derivatives against Chagas Disease

**DOI:** 10.1021/acs.jmedchem.4c01535

**Published:** 2024-11-01

**Authors:** Mercedes Rubio-Hernández, Verónica Alcolea, Elany Barbosa da Silva, Miriam A. Giardini, Thaís H. M Fernandes, Nuria Martínez-Sáez, Anthony J. O’Donoghue, Jair L. Siqueira-Neto, Silvia Pérez-Silanes

**Affiliations:** † ISTUN Institute of Tropical Health, Department of Pharmaceutical Sciences, 16754 Universidad de Navarra, 31008 Pamplona, Spain; ‡ Skaggs School of Pharmacy and Pharmaceutical Sciences and Center for Discovery and Innovation in Parasitic Diseases, 8784 University of California, San Diego, 9500 Gilman Drive, La Jolla, California 92093, United States; § Department of Pharmaceutical Sciences, 16754 Universidad de Navarra, 31008 Pamplona, Spain

## Abstract

Chagas disease is caused by the eukaryote parasite . Current treatment exhibits limited efficacy and selenium-based compounds emerged as promising candidates for new therapies which is surpassing its bioisoster, sulfur. We designed new thiosemicarbazones, thiazoles, selenosemicarbazones and selenazoles, using isosteric substitution. We synthesized 57 new chalcogen compounds which were evaluated against , C2C12 cells and cruzain, the main target of this parasite. Additionally, human cathepsin L, was tested for selectivity. Three compounds were selected, based on their activity against the intracellular amastigotes (EC_50_ < 1 μM, SI > 10) and cruzain (IC_50_ < 100 nM, SI > 5.55) which compared favorably with the approved drug, Benznidazole, and the well-established cruzain inhibitor K777. Seleno-compounds demonstrated enhanced activity and selenazoles showed a decrease in selenium-associated toxicity. Compound 4-methyl-2-(2-(1-(3-nitrophenyl)­ethylidene)­hydrazineyl)-1,3-selenazole (***Se*** **2h**) emerged as a promising candidate, and its binding to cruzain was investigated. Pharmacokinetic assessment was conducted, showing a favorable profile for subsequent *in vivo* assays.

## Graphical abstract

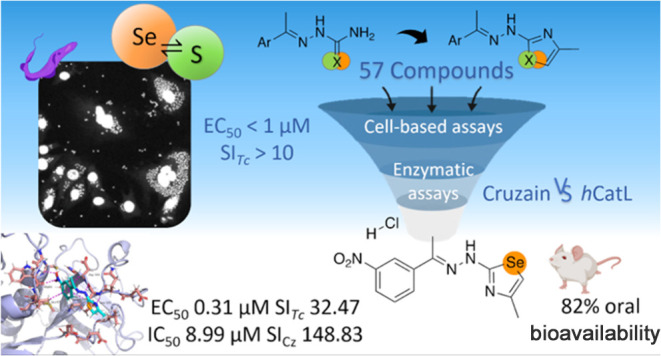

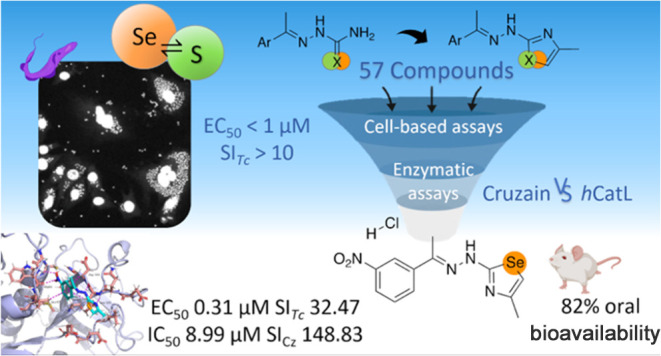

## 1 Introduction

Chagas disease (CD), also known as American trypanosomiasis, is a neglected tropical disease (NTD) caused by () that affects around 6–7 million people worldwide.^1^ This vector-borne disease mainly affects impoverished communities in Latin America, but due to immigration, an increasing number of cases are being diagnosed in nonendemic regions such as the USA, Canada, and several European countries.^1^ Indeed, Spain has the highest number of CD cases in Europe, due to vertical transmission.^2–4^

This parasitic disease develops in two phases: an asymptomatic or mildly symptomatic acute phase, followed by a latent period, and finally a chronic phase characterized by heart damage (Chagas heart disease) and digestive symptoms (megacolon and megaesophagus).^5^ The existing treatment for CD consists of two drugs, Benznidazole (**BZ**) and Nifurtimox. Both cause several side effects, and their efficacy is reduced in the chronic phase which is a problem because CD is mostly diagnosed in the late stage. Therefore, there is an urgent need for novel therapeutic alternatives.^1,4^

 is an eukaryotic parasite transmitted by Triatominae bugs. This parasite undergoes a complex lifecycle involving three forms: epimastigote (vector only), trypomastigote (infective and extracellular form, responsible for the acute phase in the host), and amastigote (intracellular form, present in the chronic stage within the host).^6^ Ideally, new treatment options for CD should demonstrate efficacy, particularly in the chronic phase of the illness. Cell-based assays serve as an initial step in assessing the activity of novel compounds. These assays employ *-*infected cells, enabling researchers to evaluate whether new molecules can either prevent infection or arrest parasite replication, reducing the number of amastigotes. Such assays offer valuable insights into the potential effectiveness of compounds in combating the disease at a cellular level, laying a foundation for further preclinical and clinical investigations.

Another approach for discovering new compounds involves enzymatic assays targeting cruzain (Cz), an important enzyme required for parasite survival at all life-stages and host cell invasion.^7–9^ Cz is a well characterized enzyme^10,11^ with a thoroughly studied active site^12,13^ that belongs to the group of the papain-like cysteine proteases. The human homologue of Cz is cathepsin L (*h*CatL)^14^ and these enzymes share 36% amino acid sequence identity.^15^ This resemblance emphasizes the importance of understanding the interactions of potential inhibitors to both enzymes and guides the development of effective therapeutics with high specificity for Cz and minimal off-target effects by targeting *h*CatL. Therefore, after reviewing the active compounds against Cz and reported in the literature, we identified several thiosemicarbazones^16^ and selenosemicarbazones^17,18^ with promising results (Figure 1). This fact, combined with the previous background of the group on the role of *Se* in CD,^19^ prompted us to plan an isosteric replacement as a strategy for the design of new compounds.^20^

**Figure 1:**
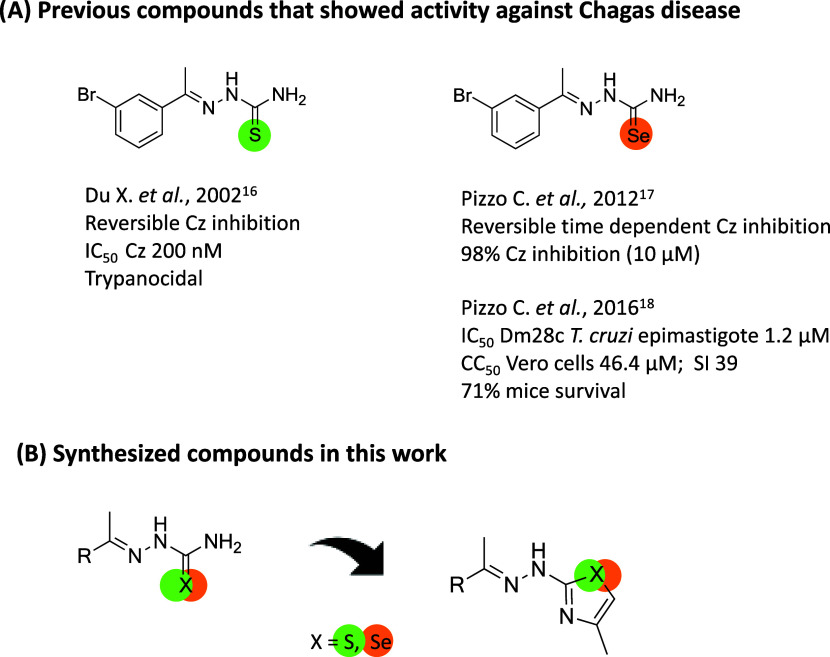
Previous compounds with activity against Chagas disease found in the literature (A) and the scaffold of the synthesized compounds in this study (B).

Isosteric replacement of sulfur (*S*) with selenium (*Se*), has emerged as a strategic approach in the development of novel and potent compounds. This idea has been used previously for cancer therapeutics,^21,22^ adenosine A2A receptor agonists^23^ and antifungal agents.^24^ Our group has previously reviewed the literature and highlighted numerous *S*-containing compounds exhibiting promising activity against CD.^20,25^ Recently, *Se* has emerged as a potential alternative for combating NTDs. Defined as a hormetic element, *Se* is essential for life, though its overdose can lead to adverse effects. *Se* plays a crucial role in the immune system and in the synthesis of selenoproteins, while its deficiency can lead to diseases.^20^

The concept of isosteric replacement relies on the idea that *S* and *Se* are bioisosters, sharing significant similarities, while differing in some properties that influence reactivity and biological outcomes. Both elements belong to the chalcogen group, however, *Se* is heavier, thus more polarizable due to its loosely bonded electrons and weak bindings. This impacts on the redox properties, which is the main difference between both elements. Consequently, *Se* exhibits greater reactivity than *S* and this heightened reactivity impacts the stability of *Se*-compounds.^26–28^

Among the *S*-compounds mentioned in the literature, thiosemicarbazones (TSCs) were chosen as a starting point for the replacement of *S* by *Se*. This choice is motivated by the existing evidence of their activity against , including *in vivo* assays.^20^ TSCs are also known as reversible covalent Cz inhibitors. Docking studies suggest that the TSCs scaffold is oriented to the catalytic Cys25 and His^162^, binding to S1′ and S2 pockets of Cz.^29–32^ TSCs emerge as promising candidates for CD treatment because they have several advantageous characteristics. First, they possess a low molecular weight, facilitating their penetration into biological systems and potentially enhancing their bioavailability. Second, TSC synthesis is cost-effective, making them economically viable for large-scale production. Additionally, TSCs are nonpeptidic molecules, avoiding liability problems of peptidic compounds when administered orally. Therefore, we consider TSCs a good starting point to obtain selenosemicarbazones (*Se*SCs), their selenated counterparts. We will also study thiazoles (TZs) and selenazoles (*Se*Zs) as they are the cyclic bioisosters of TSCs and *Se*SCs, respectively. Of note, this study reports the first evaluation of *Se*Zs against . The main goal of this work is to obtain an active compound with improved antiparasitic activity while also conducting a comparative analysis between structures containing *S* *versus* *Se*.

## 2 Results

### 2.1 Chemistry Results

In this work, we synthesized 32 compounds containing *S* and 25 compounds containing *Se*. *S*-*Se* isosteric replacement was used for the design of the new compounds and TSCs were used as a starting point because they are known Cz inhibitors. The synthesis pathway for **Series 1** (***S*** **1** and ***Se*** **1**) derivatives and their cyclic counterparts **Series 2** (***S*** **2** and ***Se*** **2**) is simple and cost-effective, being a suitable route for a NTD like CD.

The synthesis of the intermediate **I3** is shown in Scheme 1. First, commercially available **I1** was methylated with methyl iodide. Then, elemental *Se* was reduced and incorporated into **I2** to obtain the intermediate **I3**.

**Scheme 1:**
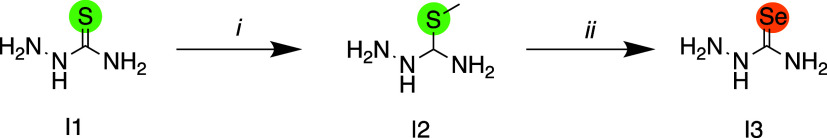
Reagents and Conditions for the Synthesis of Intermediate Hydrazinecarboselenoamide (**I3**)­^a^

a (i): EtOH, Methyl iodide. Reflux (2h). (ii): Anhydrous EtOH, N_2_, *Se*, NaBH_4_, 0°C, RT (overnight).

The final compounds presented in this work were obtained through Hantzsch reaction. Of note, synthesis and purification of *S* derivatives (17 TSCs and 15 TZs) was easier than *Se* derivatives (12 *Se*SCs and 13 *Se*Zs). Indeed, compounds from series ***Se*** **1** had the lowest yield (24%). As shown in Scheme 2 (step i), compounds from **Series 1** were obtained after condensation between the corresponding methyl ketone (**a-q**) and commercial **I1** (***S*** **1** derivatives) or **I3** (***Se*** **1** derivatives). Derivatives from **Series 1** (***S*** **1** and ***Se*** **1**) were purified if they were considered as final products. If not, the cyclation was continued in the same batch. Posterior condensation of compounds from **Series 1** with chloroacetone led to the obtention of the corresponding chalcogen cyclic derivatives from **Series 2** (***S*** **2** and ***Se*** **2** derivatives) (Scheme 2, step ii). The different substituents (R) used in each methyl ketone (**a-q**) are described in “starting methyl ketones” (Scheme 2).

**Scheme 2:**
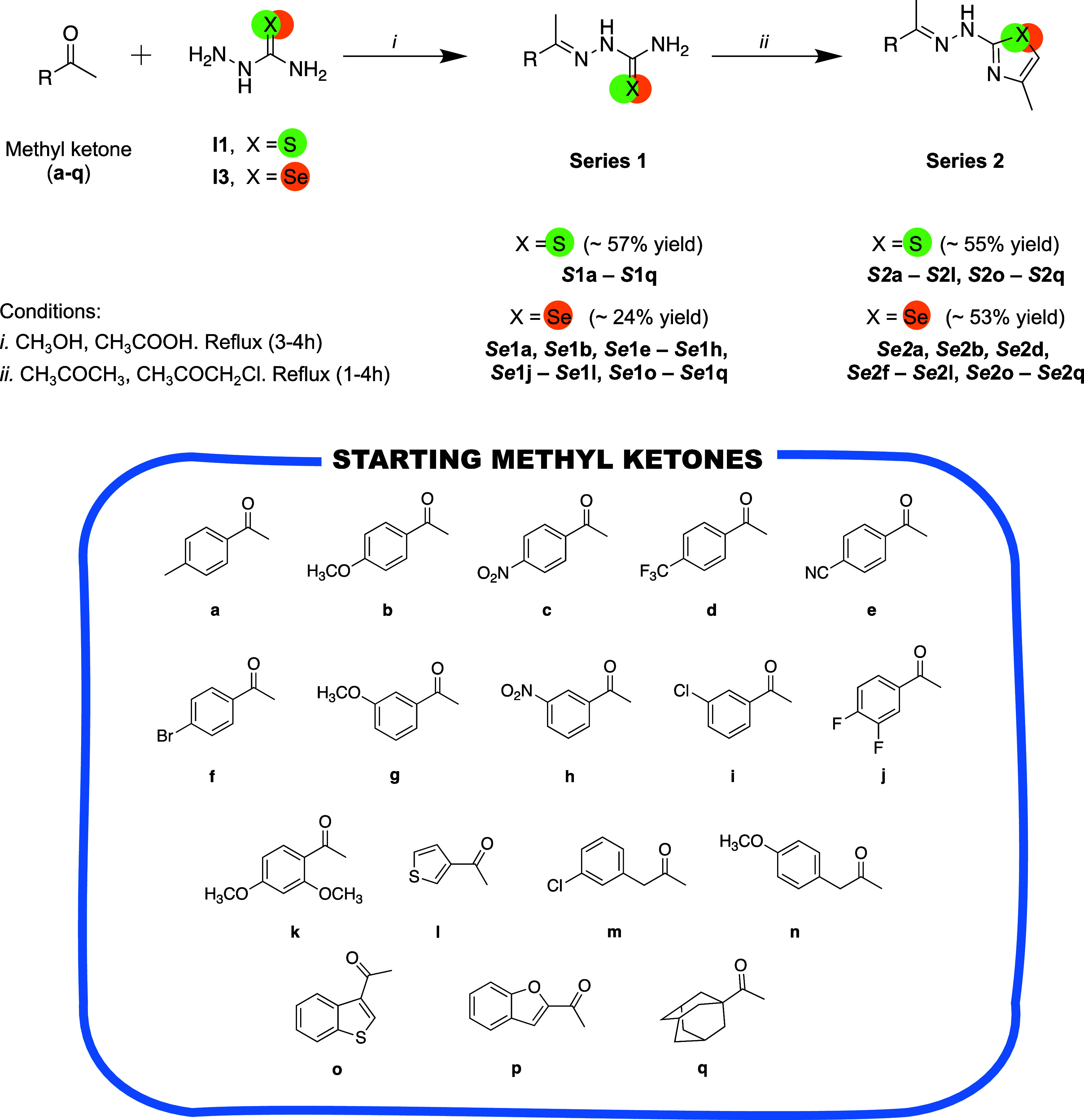
General Synthesis Route of **Series 1** and **2^a^**

a R relates to the corresponding substituent of the starting methyl ketone (**a-q**) used for each compound.

After compound characterization, derivatives ***S*** **1k**, ***Se*** **1k**, ***Se*** **2k** showed isomers in ^1^H and ^13^C NMR spectra (Supporting Figures S23, S24, S55, S113 and S114). It is already described in the literature TSCs and TZs have *E*, *Z* isomers.^13,33,34^ So, we confirmed *E* isomer predominates over *Z* isomer with bidimensional spectra (Supporting Figures S123 and S124).

### 2.2 Cell-Based Assays

A total of 57 compounds were tested at 10 μM against the trypomastigote and amastigote forms of CAI/72 strain (DTU TcI^35^). Antiparasitic activity (Anti-*Tc* CAI/72), and toxicity (C2C12 viability), were assayed at the same time (See Supporting Table S1). As a result, 48 compounds out of 57 were not cytotoxic to C2C12 cells based on a cytotoxic concentration 50 (CC_50_) of >10 μM. Seven compounds showed antiparasitic activity greater than 50% at 10 μM; with five of them containing *Se*. The half maximal effective concentration (EC_50_) of these 7 compounds was calculated and all but one (***S*** **1i**) exhibited more potent activity (EC_50_ ranging from 0.308 to 3.73 μM) against the parasite compared to the reference drug **BZ** (EC_50_ 5.79 μM) (Table 1). Compound ***Se*** **1e**, which fulfilled EC_50_ requirements, was discontinued because of stability problems.

**Table 1:**
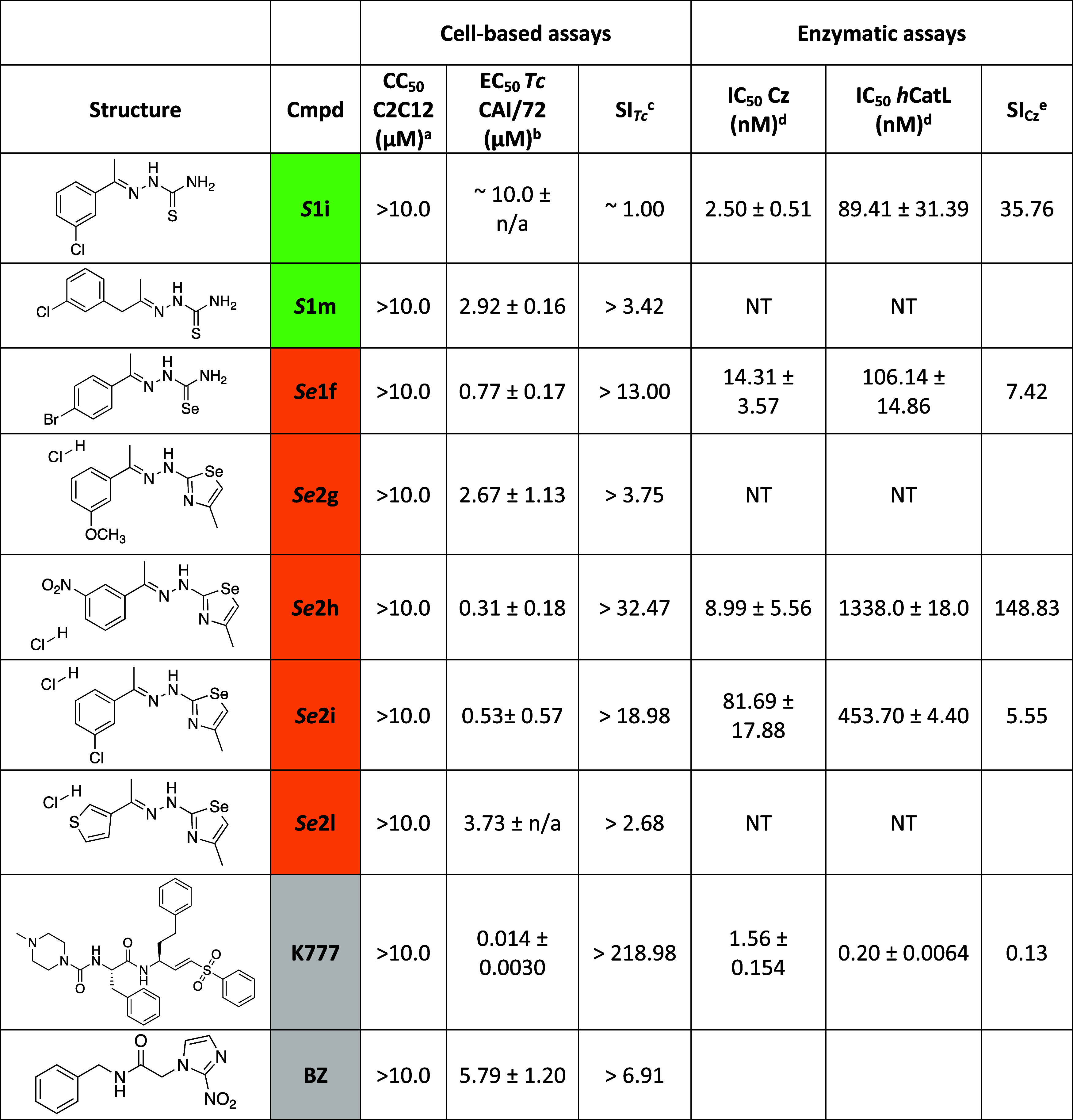
Results of Active Compounds in Cell-Based Assays and Enzymatic Assays^f^

a CC_50_ C2C12 (μM) is the concentration in which the viability is calculated at 10 μM.

b EC_50_ (μM) is expressed as the average of three independent experiments in triplicates (*n* = 9) ± standard deviation (SD) against intracellular amastigote parasites.

c SI_Tc_ shows the selectivity for the parasite, and it is the ratio between CC_50_ C2C12 and EC_50_ Tc CAI/72.

d IC_50_ (nM) is shown as a mean of two different experiments against Cz and hCatL, each of them performed in triplicates (*n* = 6) ± standard error of the mean (SEM). Error is given by the ratio of the standard deviation to the square root of the number of measurements.

e SI_Cz_ shows the selectivity for Cz and it is calculated as the ratio between IC_50_ hCatL and IC_50_ Cz.

f NT: no tested. n/a: no aplicable.

Compounds ***Se*** **1f**, ***Se*** **2h**, ***Se*** **2i** were more potent than **BZ** (EC_50_ 5.79 μM, SI > 6.91), with EC_50_ under 1 μM and selectivity index (SI) higher than 10 (Table 1).^36^ In Figure 2 is shown the effect of ***Se*** **2h** against , compared to DMSO and **BZ**, the reference drug. Of note, **BZ** and ***Se*** **2h** look very similar, but we have to consider the maxima concentration we used for ***Se*** **2h** is 10 μM *versus* 40 μM of **BZ**.

**Figure 2:**
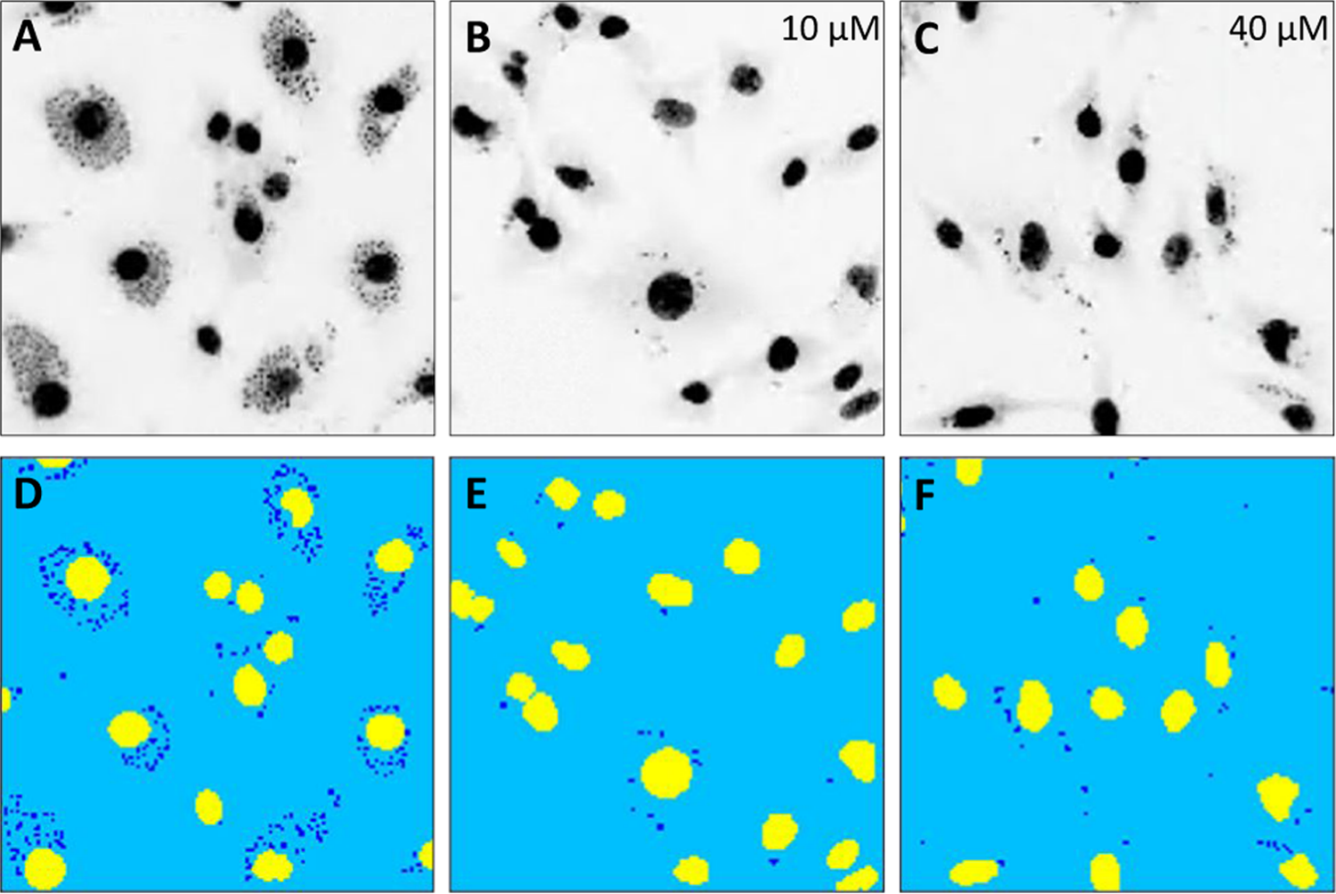
C2C12 cells infected by CAI/72. Pictures of wells treated with **DMSO** (A, D); ***Se*** **2h** at 10 μM (B, E); **BZ** at 40 μM (C, F). Charts A, B and C correspond to the real picture of the well; Charts D, E, F correspond to the analysis pictures (DAPI staining).

Therefore, we performed dose–response curves (DRC) of antiparasitic activity of the previously mentioned derivatives (***Se*** **1f**, ***Se*** **2h**, ***Se*** **2i**), using **BZ** and **K777** as positive controls (Supporting Figure S1). All curves were normalized by DMSO controls and **BZ** EC_100_.

It is noteworthy that the three selected compounds all contain *Se* (***Se*** **1f**, ***Se*** **2h**, ***Se*** **2i**). This, in agreement with our main hypothesis suggesting that *Se-*containing molecules improve activity against .

Based on antiparasitic results, there was no significant difference in activity between **Series 1** and **2**. Interestingly, for anti- activity, in general electron withdrawing groups, like halogens, nitrile, and nitro group, are preferred in the aromatic ring (Supporting Table S1).

### 2.3 Enzymatic Evaluation of Prioritized Hits

In parallel to cell-based assays, the inhibitory activity against recombinant Cz at 10 μM was assessed for all synthesized compounds. A total of 33 compounds showed inhibition of Cz greater than 85% at 10 μM (Supporting Table S1). Moreover, all ***Se*** **1** derivatives presented more than 80% enzyme inhibition. Among the 33 active compounds against enzyme, we prioritized the inhibitors that reduced parasite replication by ≥50%. Compounds ***Se*** **1f** (4Br-Ph *Se*SC), ***Se*** **2h** (3NO_2_–Ph *Se*Z), ***Se*** **2i** (3Cl-Ph *Se*Z), and ***S*** **1i** (3Cl-Ph TSC) were selected to perform DRC against Cz. Compound ***Se*** **1e** was eliminated from enzymatic assays due to a lack of stability. The potency of the top four compounds were in the nanomolar range with the best compounds being ***S*** **1i** (IC_50_ = 2.5 nM) and ***Se*** **2h** (IC_50_ = 8.99 nM) (Figure 3 and Table 1). As a comparison, the potency was similar to **K777** (IC_50_ = 1.56 nM), a well characterized Cz inhibitor.^37^ In order to complete SAR studies, we added compounds ***Se*** **1h** (3NO_2_–Ph *Se*SC), ***S*** **1h** (3NO_2_–Ph TSC), ***S*** **2h** (3NO_2_–Ph TZ), and ***S*** **2i** (3Cl-Ph TZ) for analysis and comparison with ***Se*** **2h** (3NO_2_–Ph *Se*Z) and ***S*** **1i** (3Cl-Ph TSC). The additional compounds demonstrated activity against Cz but did not exhibit efficacy against the parasite (Table S1).

**Figure 3:**
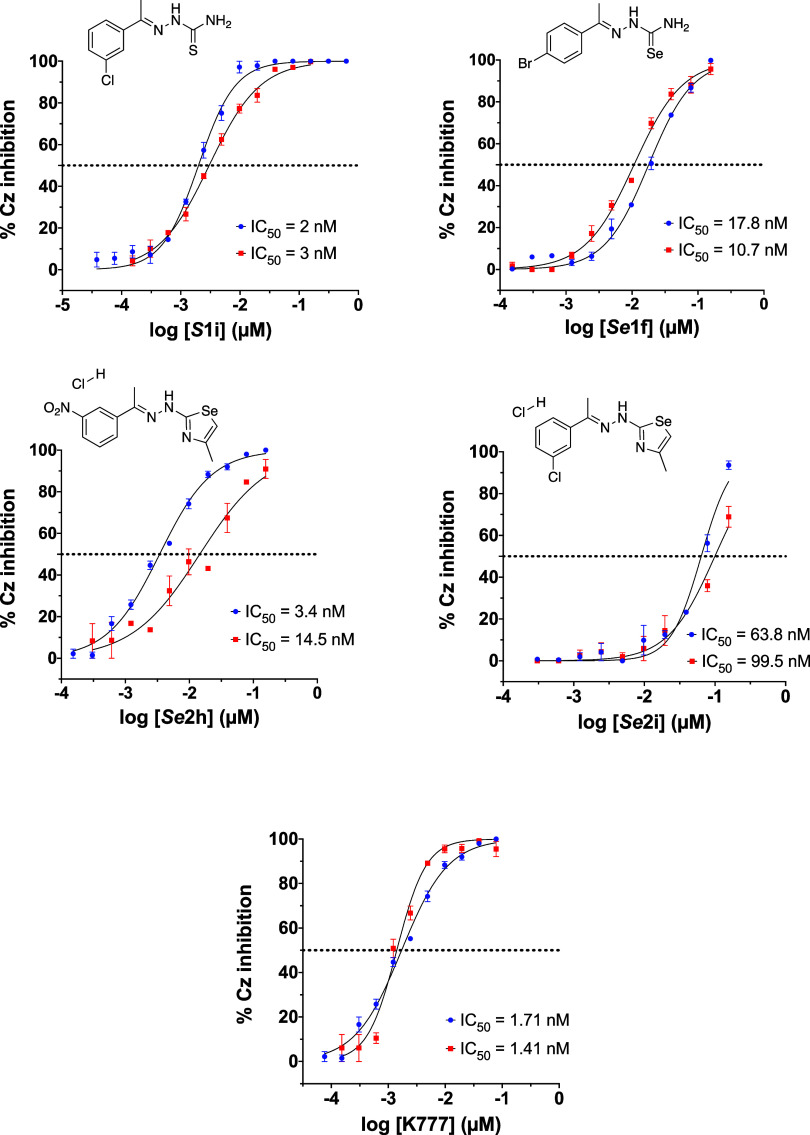
DRC for compounds ***Se*** **1f**, ***Se*** **2h**, ***Se*** **2i**, ***S*** **1i** and **K777** *versus* Cz. Each curve has two biological replicates performed in triplicates (*n* = 6). The error bars in the graphs correspond to SEM, which is calculated as the ratio of the standard deviation to the square root of the number of measurements. **K777** is a cysteine protease inhibitor used as positive control.

As previously stated, *h*CatL and Cz are homologues and they share high similarity mainly in the substrate binding site.^15^ Therefore, we performed assays with *h*CatL in order to study the selectivity of our compounds. For the screening against *h*CatL, we focused on the compounds that inhibited parasite growth more than 50% and also were active against Cz (Table 1). Compounds that fulfill these criteria and inhibited *h*CatL by more than 90% at 10 μM were further characterized. The potency of ***Se*** **1f** (4Br-Ph *Se*SC), ***Se*** **2h** (3NO_2_–Ph *Se*Z), ***Se*** **2i** (3Cl-Ph *Se*Z), and ***S*** **1i** (3Cl-Ph TSC) were calculated (Figure 4, Tables 1 and S1).

**Figure 4:**
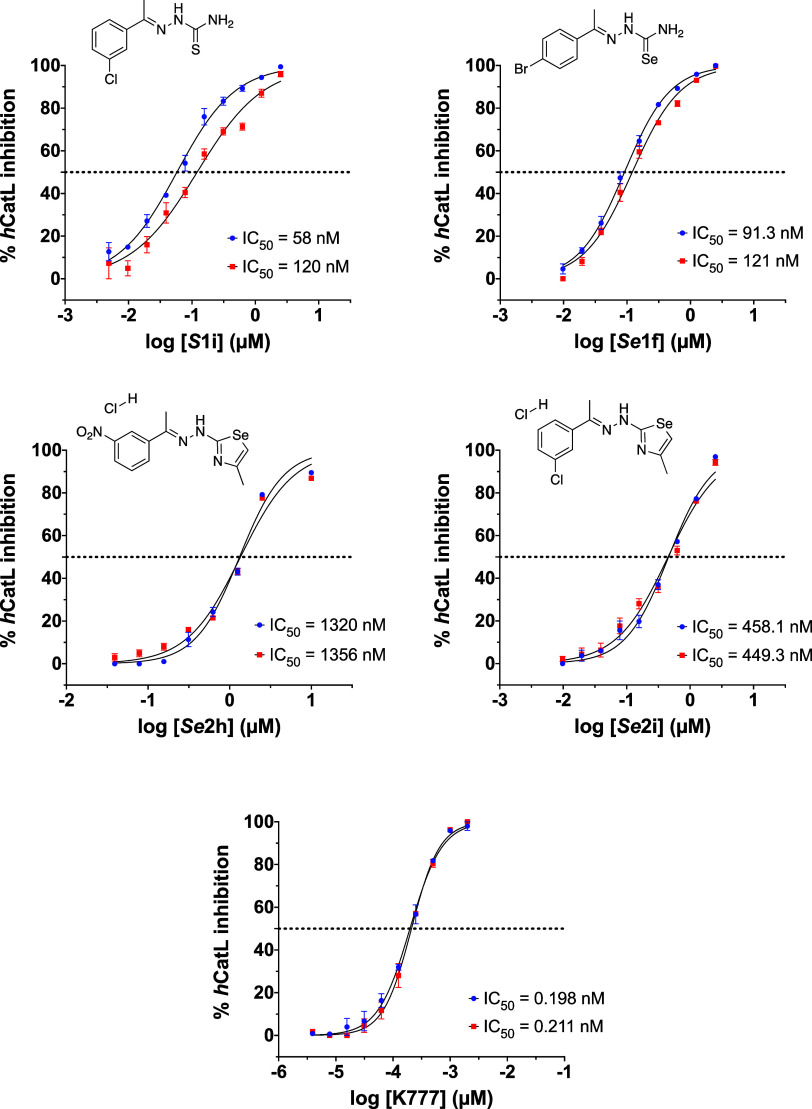
DRC of compounds ***Se*** **1f**, ***Se*** **2h**, ***Se*** **2i**, ***S*** **1i** and **K777** in *h*CatL. Each curve has two biological replicates performed in triplicates (*n* = 6). The error bars in the graphs correspond to SEM, which is calculated as the ratio of the standard deviation to the square root of the number of measurements. **K777** was used as reference inhibitor of cysteine proteases.

Furthermore, DRC of compounds ***Se*** **1h** (3NO_2_–Ph *Se*SC) and ***S*** **1h** (3NO_2_–Ph TSC) were also generated using *h*CatL to perform a SAR study. IC_50_ values are shown in Table S1.

We observed that our best compounds, ***S*** **1i** and ***Se*** **2h**, are 36 and 149 times more selective for Cz over *h*CatL, respectively (Table 1). While **K777** was used as the positive control, its SI for Cz over *h*CatL is 0.13. Therefore, ***S*** **1i** and ***Se*** **2h** are at least 275 times more selective for Cz than **K777** making them excellent candidates for further studies.

### 2.4 Molecular Dynamics Simulations

To study the mode of interaction of ***Se*** **2h** with Cz, molecular dynamics (MD) simulations were performed. The structure deposited in the Protein Data Bank (PDB) under the ID 3KKU was used to prepare the starting structure (See Supporting Information). The ligand from the crystal structure was replaced by the selenated derivative prior to the MD simulations, and the complex stability was studied over a 500 ns trajectory.^38^ The average value of backbone RMSD from equilibrium simulations is 1.48 Å. The complex achieved structural stabilization after 90 ns (See Supporting Figure S2).

An interaction map of ***Se*** **2h** at the Cz binding site is shown in Figure 5. Carbonyl group of Leu^160^ acts as hydrogen bond acceptor interacting with ligand NH (2.8 Å), Leu^160^ also interacts through its NH with the ligand endocyclic nitrogen (2.9 Å). Additionally, an hydrogen bond is present between Gly^66^ NH and the nitro group oxygens (2.9 Å). This substituent rotates so, the hydrogen bond alternates between both oxygens of the NO_2_ group. Of note, apart from these hydrogen bonds, a π-hole interaction^39^ between the carbonyl group of Gly66 and the electron-deficient nitrogen of the ligand’s nitro aromatic group is observed (3.4 Å). Leu67, Leu^160^, and Asp^161^ also contribute to the binding through CH-π interactions with the aromatic ring of the ***Se*** **2h** ligand, with average distances of 3.4, 3.8, and 3.8 Å, respectively (See Supporting Table S2).

**Figure 5:**
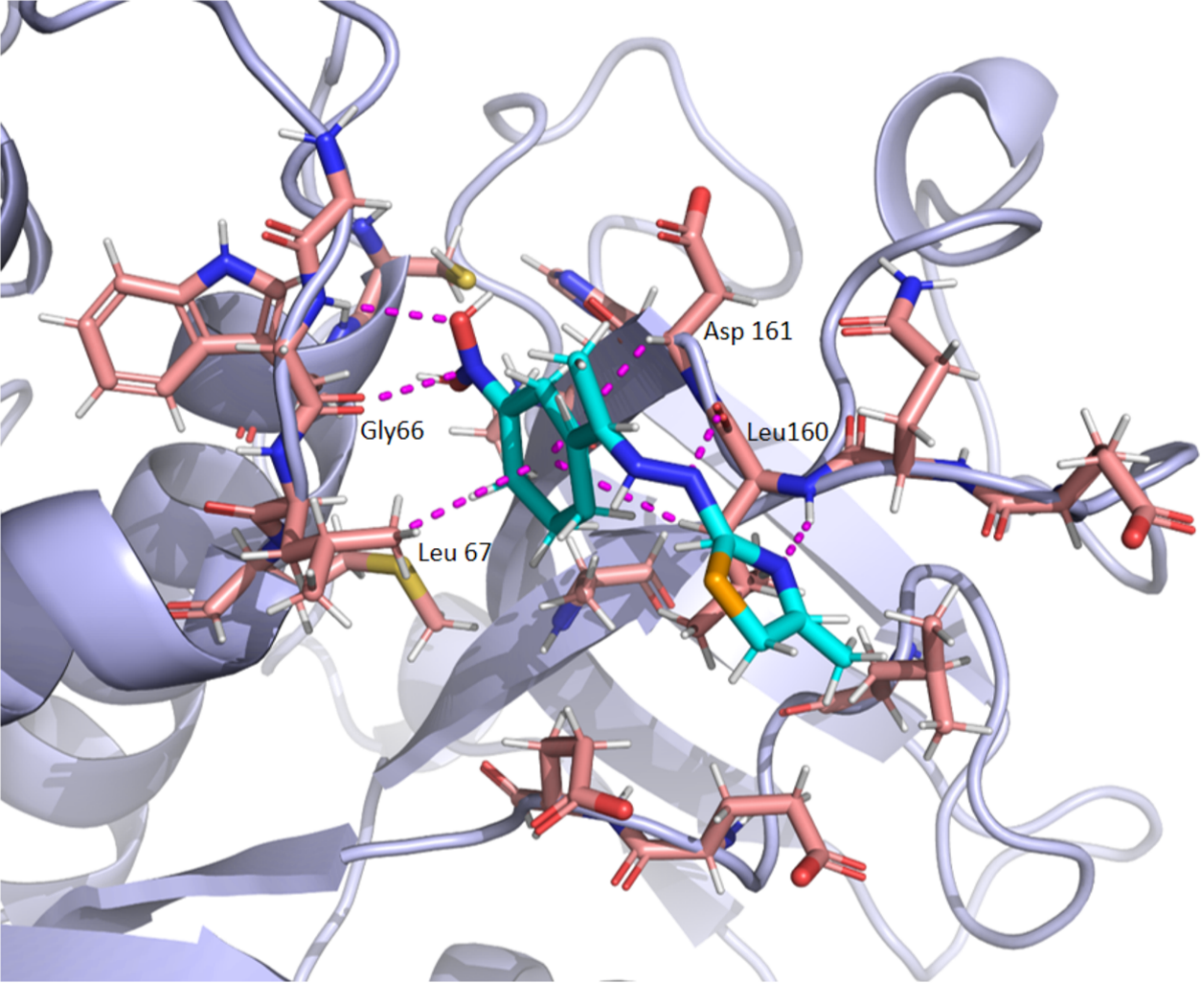
Representative frame of the binding mode of ***Se*** **2h** at Cz active site (PDB 3KKU) obtained by MD simulation. Pink dashed lines indicate all the interactions established between the ligand and the protein, including hydrogen bonds, CH-π and π-hole interactions.

### 2.5 Radical Scavenging Capacity

We evaluated the antioxidant activity of the hit compound, ***Se*** **2h**, at three different concentrations (0.06, 0.03, and 0.015 mg/mL). Data and graphical representation of the maxima antioxidant activity of ***Se*** **2h** after 2 h are shown in Figure 6 and Supporting Table S3.

**Figure 6:**
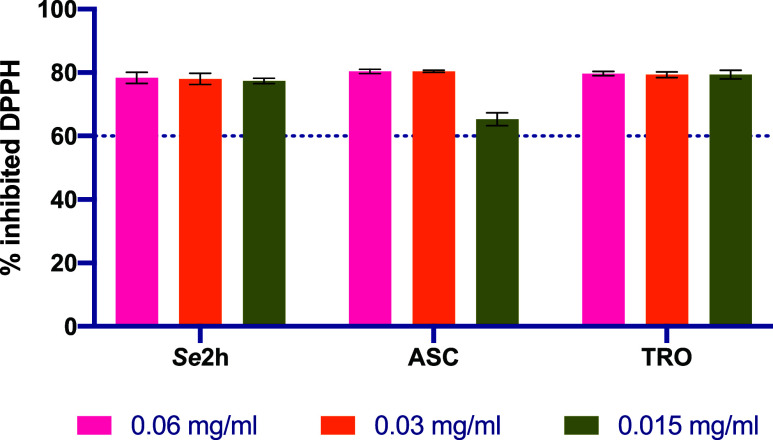
Percentage of inhibited DPPH (%) after 2 h of ***Se*** **2h** and the positive controls (ascorbic acid, ASC and Trolox, TRO). Each bar shows the mean of inhibited DPPH for each concentration and the error bars show the SEM.

***Se*** **2h** showed more than 60% inhibition of DPPH after 2 h at all the tested concentrations (0.06, 0.03, and 0.015 mg/mL). Notably, ***Se*** **2h** remains constant at all concentrations tested, so it is comparable to the positive controls used in this assay, ascorbic acid (ASC) and trolox (TRO). This led us to conclude ***Se*** **2h** is an antioxidant compound.

We also performed the kinetic curve of ***Se*** **2h** for the antioxidant activity at the lowest concentration tested (0.015 mg/mL) (Figure 7). Comparing its curve to the controls, our compound reaches the plateau later (30 min) than ASC (0 min) and TRO (5 min). Regarding the percentage of activity, it is similar to TRO (80%) and higher than ASC (60%).

**Figure 7:**
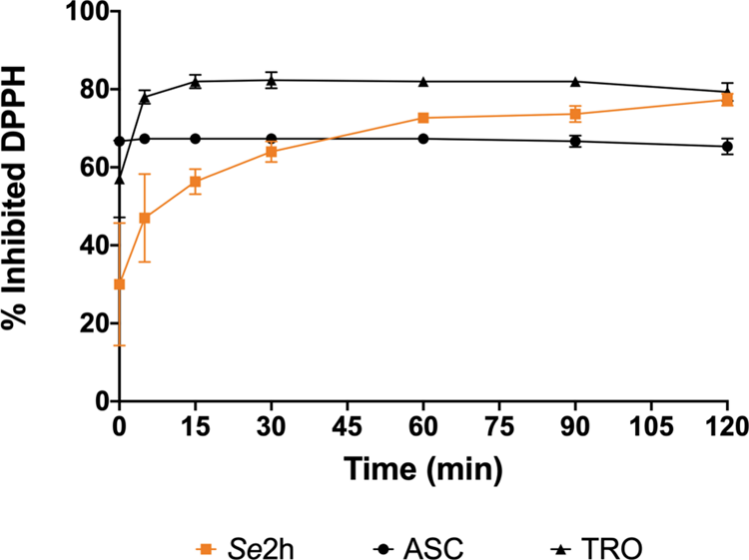
Kinetic curve of ***Se*** **2h** and positive controls, ascorbic acid (ASC) and trolox (TRO) at the lowest concentration tested 0.015 mg/mL and performed for 2 h.

### 2.6 Pharmacokinetic Study

Compound ***Se*** **2h** showed the best Cz inhibition, antiparasitic activity against and selectivity when compared to the mammalian cells. Before testing the compounds *in vivo* for proof-of-concept efficacy assessment, a snapshot pharmacokinetic (PK) study was performed. Compound ***Se*** **2h** was administered intravenous (5 mg/kg) and orally (50 mg/kg). The oral bioavailability was 82%, and the *C*_max_ with the oral dose was 2.3 μM at 30 min post bolus administration, but the half-life (*T*_1/2_) was less than 1 h, suggesting metabolic instability, high volume of distribution, or fast clearance.

## 3 Discussion

*Selenium* has emerged as an alternative for the treatment of CD following a clinical trial that showed some positive outcomes for CD patients.^40^ However, the available information comparing *S* and *Se* derivatives in CD treatment is scarce, so far.^20^ To bridge this gap, we used the isosteric replacement strategy and TSCs as a starting point to synthesize new compounds. We chose TSCs because of their extensive study and proven potency as Cz inhibitors.^13,16,29,31,41^ Our hypothesis suggests *Se*-compounds would exhibit similar inhibitory properties to *S*-compounds, but potentially with enhanced activity due to higher reactivity of *Se*. With this concept in mind, we designed two series: **Series 1**, comprising TSCs (17 molecules) and their *Se*-counterparts (*Se*Cs) (12 molecules); **Series 2**, consisting of their cyclic bioisosters, TZs (15 molecules) and *Se*Zs (13 molecules), respectively. This design allows direct comparison of the efficacy of each scaffold. Figure 8 shows the structures of the molecules synthesized in this work, highlighting the chemical space used for the SAR study.

**Figure 8:**
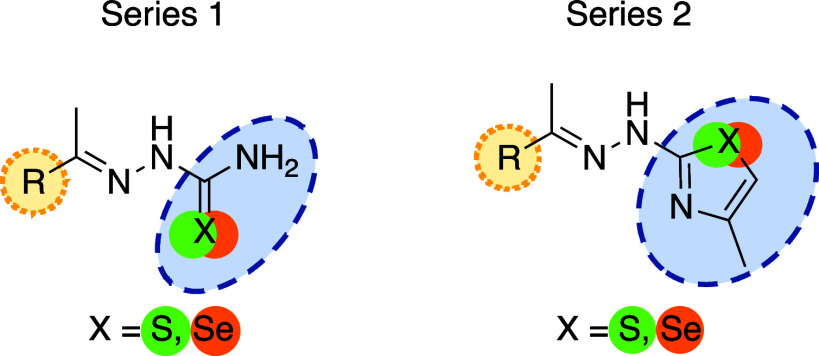
General structure of the frameworks used in this work. **Series 1** corresponds to thio- or seleno-semicarbazone scaffold and **Series 2** corresponds to thio- or selen-azole scaffold. The chemical space employed for the SAR study is highlighted in yellow and blue.

As a novel approach, *Se*Zs derivatives will undergo testing against for the first time. While this class of compounds has been previously evaluated for other pathologies such as botulism,^42^ epilepsia,^43^ cancer,^44,45^ filariasis,^44^ candidiasis,^46^ and ;^47^ including *in vivo* studies,^42–44^ their efficacy against CD has not ever been explored.

Regarding *Se* chemistry, it presents some challenges in contrast to *S* chemistry, even though both elements are bioisosters. The synthesis pathway involving *S* is straightforward, characterized by high yields, minimal stability issues and a cleaner and less odorous process compared to the synthesis of *Se* compounds. Indeed, *Se*-compounds are more prone to instability, and compound ***Se*** **1e** (4CN-Ph *Se*SCs) was discontinued for this reason. However, both synthetic routes remain relatively simple and cost-effective despite these challenges, making them suitable for a novel NTD therapeutic drug development.

In general, all tested compounds were no cytotoxic, except for seven ***Se*** **1** derivatives that belong to *Se*SC group (***Se*** **1a**, ***Se*** **1b**, ***Se*** **1g**, ***Se*** **1k**, ***Se*** **1l**, ***Se*** **1o**, ***Se*** **1p**). Nevertheless, their cyclic counterparts were no toxic in the evaluated mammal system, with 100% cell viability (*e.g.*, ***Se*** **1p** 39.4% *vs* ***Se*** **2p** > 100% and ***Se*** **1l** 38.12% *vs* ***Se*** **2l** > 100%). Compound ***Se*** **1j** (27.7% cell viability) and ***Se*** **2j** (38.28% cell viability) were discontinued due their toxicity, that can be associated with the substituent (3,4-difluorophenyl) (Table S1). These results suggest selenazole structure improves toxicity profile of *Se* derivatives, while it preserves activity. This reinforces the idea of *Se* is less toxic included in a cycle, as Wei Hou and Hongtao Xu described before.^48^

In terms of anti- activity, we found seven compounds with more than 50% activity (Table 1). Among them, ***Se*** **2h** has the lowest EC_50_ (0.31 μM), so we analyzed its effect on the well (Figure 2). Compared to **BZ**, ***Se*** **2h** demonstrated superior efficacy, achieving similar results with only one-fourth of the dose required for **BZ**.

Regarding enzymatic activity, we performed assays on Cz, which is the major cysteine protease in and known to be essential. It is present in all life stages, and plays a crucial role in both, parasitic survival and host cell invasion. From the seven compounds with antiparasitic activity (Table 1), we focused on ***Se*** **1f**, ***Se*** **2h**, ***Se*** **2i** and ***S*** **1i** because they also showed Cz inhibition (>85% at 10 μM). Furthermore, we look for selectivity against Cz, so we tested the selected compounds (***Se*** **1f**, ***Se*** **2h**, ***Se*** **2i** and ***S*** **1i**) against its homologue, *h*CatL. Both enzymes share a high amino acid sequence identity, mainly at the active site. They belong to the cysteine protease group and share 36% similarity.^15^ Our interest in selectivity is because we are aware of the selectivity issues of **K777**. This compound is used as a reference inhibitor that binds covalent and irreversibly to cysteine proteases.^37,49^ It was originally developed as a cathepsin S inhibitor, but then it showed its potential against .^49,50^ Despite its lack of selectivity for Cz (SI 0.13), **K777** completed a phase I clinical trial. However, it was stopped due to tolerability issues.^51–53^

Summarizing and enzymatic data, compounds ***Se*** **1f**, ***Se*** **2h**, ***Se*** **2i** and ***S*** **1i** are eligible for further studies due to their promising profile. To deeply analyze the selected derivatives and choose the best one, we generated a graphical representation by plotting EC_50_ against IC_50_ Cz, as illustrated in Figure 9. Our aim was to identify compounds with activity against both (EC_50_ < 1 μM, SI > 10) and Cz (IC_50_ < 100 nM).

**Figure 9:**
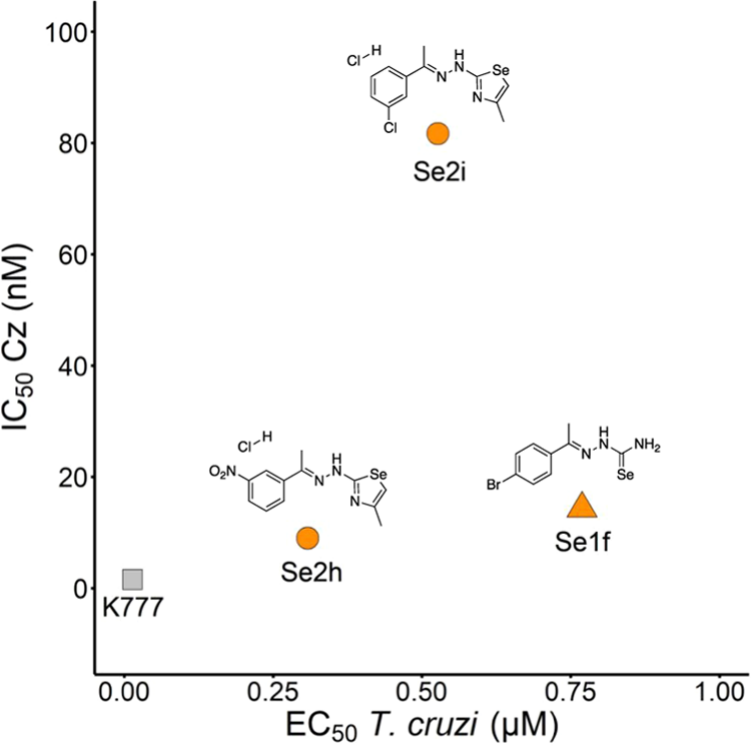
Active compounds against (EC_50_ < 1 μM) and Cz (IC_50_ < 0.1 μM) selected for further studies. **K777** (shadow in gray) is used as a reference compound. ***S*** **1i** does not appear in the graph because its EC_50_ is far from the limit (EC_50_ ∼ 10 μM).

Among the synthesized derivatives, none of them improved the **K777** activity profile, but they did show higher selectivity (Table 1). Despite ***S*** **1i** was the most active against Cz (IC_50_ = 2.50 nM) and 275 times more selective than **K777** (SI 35.76), it is not shown in the graph because its activity against is higher than 1 μM (EC_50_ ∼ 10 μM). This compound has previously been described as a tyrosinase^34^ and Cz inhibitor (IC_50_ Cz = 29.9 μM).^13^ The difference in IC_50_ values against Cz could be attributed to the different assay conditions (as temperature, DMSO and DTT concentration) and mainly, the origin of the enzyme used for the assay. The Cz used in this work comes from a designed plasmid, as the one used in Blau et al.^54^ The Cz used in Jasinski et al.^13^ is isolated and purified from Tulahuen 2 strain epimastigotes. Additionally, compound ***Se*** **2h** (IC_50_ Cz = 8.99 nM, SI 148.83) demonstrated being 1000 times more selective than **K777**, what could allow great therapeutic dosing without toxicity. Therefore, we consider ***Se*** **2h** a good candidate to go further into *in vivo* assays. It demonstrated the lowest EC_50_ and the highest SI against the parasite, coupled with superior activity and selectivity against Cz.

Before moving to *in vivo* assays, we performed molecular dynamic (MD) simulations on the compound ***Se*** **2h** to elucidate the potential interactions that govern its molecular recognition by Cz. According to the literature, TSCs bind to Cz in a time-dependent, covalent, reversible, and competitive way.^13,16,30^ Therefore, we hypothesized that our synthesized compounds, would also target Cz due to their bioisosteric nature.^25^ We proved this statement experimentally, performing the enzymatic assays against Cz. But we decided to study the interactions between ***Se*** **2h** and Cz in depth, as it is the first time *Se*Zs are tested in CD. After analyzing the interactions, we realized ***Se*** **2h** binds to Cz in a noncovalent way, contrary to TSCs and **K777**. The different type of binding between TSCs and TZs is already described by de Barros Dias et al.^33^ In our case, ***Se*** **2h** interacts at the binding site through specific hydrogen bonding with Leu^160^ and Gly^66^. These contacts, observed intermittently along the trajectory (See Supporting Table S2), have been previously described for the Cz-ligand system (X-ray studies).^32,38,55^ In addition to the hydrogen bond network, a π-hole interaction between the carbonyl group of Gly^66^ and the nitro aromatic group of ***Se*** **2h** is present during 70% of the trajectory, enhancing complex stability and the binding process. The phenyl group also engages through CH-π interactions with multiple residues (Leu^67^, Leu^160^, and Asp^161^), contributing to a conformation in which the *Se* atom of the selenazole moiety does not participate in the interactions. This presents a different binding mode compared to semicarbazones, which as previously mentioned, are disposed toward the catalytic residues Cys^25^ and His^162^.^33^ These results indicate that the cyclization of the thiosemicarbazone moiety could modify the binding mode of the ligands. In this context, complex between Cz and several TZ-derived compounds with trypanocidal activity have been studied, revealing a different binding mode from noncyclic semicarbazones. These findings align with our results, indicating that in the case of the *Se*Z derivative ***Se*** **2h**, both the π-hole interaction and the hydrophobic CH-π contacts play a key role in the binding mode. In addition, the above-mentioned hydrogen bond that involves the endocyclic N of the *Se*Z ring and the NH of the *Se*SC analogue validate our design, proving the impact of both moieties in the biological activity of the ligand. Nevertheless, to confirm how ***Se*** **2h** binds to Cz, more studies on X-ray structure of the complex should be performed.

We also performed DPPH assay to evaluate the antioxidant capacity of our lead compound. In Figures 6 and 7, ***Se*** **2h** exhibited antioxidant activity comparable to positive controls (ASC and TRO). While this antioxidant capacity may seem contradictory to current understanding, it may contribute to the efficacy of ***Se*** **2h** against . The parasite has an antioxidant defense system to protect itself from the immune response. During infection, host cells generate pro-inflammatory and oxidative stress to combat , so an antioxidant compound would seem to help the parasite. However, Mesías et al.^56^ and Paiva et al.,^57,58^ reported that an oxidative milieu enhances the progression of . Thus, ***Se*** **2h** may inhibit the parasite at the early stage (acute phase), preventing trypomastigotes from infection (Figure 2). Nevertheless, further studies are needed to confirm this hypothesis.

Additionally, as the oxidative environment has a dual role enhancing and combating the parasite, our compound can show both antioxidant and pro-oxidant behavior that would influence the activity. ***Se*** **2h** contains a nitro (NO_2_) group that can be reduced to an amino group (NH_2_) by nitro-reductases, inhibiting the antioxidant defense of the parasite. This is the mechanism of action described for **BZ**, the reference compound,^59–61^ and could be another potential target of ***Se*** **2h**.

Finally, a PK study was conducted with the compound ***Se*** **2h**, evaluating both intravenous and oral administration routes. The results of the PK analysis revealed no signs of acute toxicity, adequate oral bioavailability (82%) but short half-life, as **K777**.^53^ However, the compound should be optimized before *in vivo* efficacy studies.

To optimize the efficacy of ***Se*** **2h**, additional assays focusing on binding mode and PK parameters related to possible metabolic instability and distribution are imperative.

Analyzing the SAR (Figure 8), several substituents with different electronic nature were tried in the aromatic ring, but electron withdrawing groups were preferred for the biological activity, against and Cz (–Br, –OCH_3_, –NO_2_, –Cl). What is striking is five out of the seven selected compounds contained *Se* (***Se*** **1f**, ***Se*** **2g**, ***Se*** **2h**, ***Se*** **2i**, ***Se*** **2l**). Therefore, upon examination of the cyclic bioisosters, we can confirm that *Se* enhances the activity of the compounds. Specifically, four of the selected compounds belong to ***Se*** **2 series** whereas no one of the compounds from the ***S*** **2 series** showed activity (Table S1). Thus, far, with the experiments performed we can conclude *Se*Z structure decreases *Se* toxicity while it preserves antiparasitic activity.

## 4 Conclusions

Among the 57 synthesized compounds, ***Se*** **2h** emerges as a promising candidate that combines activity and selectivity against and its main target, Cz. This compound improves **BZ** antiparasitic activity and has encouraging outcomes when administered orally.

Despite it was the first time *Se*Zs were tested against CD, we found this cyclic structure decreases *Se* toxicity while increasing anti activity, compared to *Se*SCs and TZs, respectively. Moreover, the selenazole ring changes the mode of interaction within the pocket of Cz. According to MD, ***Se*** **2h** binds noncovalently to Cz, unlike TSCs and **K777**, which bind covalently to Cz. To verify these findings, we plan to conduct X-ray crystallography experiments.

In summary, ***Se*** **2h** represents a good starting point for developing potent and selective derivatives against CD with minimal host toxicity.

## 5 Experimental Section

### 5.1 Chemistry

Chemical reagents used for compound synthesis were purchased from E. Merck (Darmstadt, Germany); Sigma-Aldrich (Saint Louis, MO); Fluorochem Ltd. (Hadfield, Derbyshire, United Kingdom); Thermo Fisher Scientific (Waltham, MA). The progress of all reactions was monitored by thin layer chromatography (TLC) using SIL G/UV254, 0.2 mm thickness (ALUGRAM Xtra SIL G, Macherey-Nagel GmbH & Co. KG) as the stationary phase, and solvent mixtures (hexane/ethyl acetate) as the mobile phase; UV lamps (254 nm) were employed to spots detection.

Infrared spectra (IR) were recorded on an Agilent Cary 630 FTIR spectrophotometer (Agilent Technologies) using ATR technique for solid samples. Proton (^1^H) and carbon (^13^C) NMR spectra of every compound were recorded on a Bruker Advance Neo 400 Ultrashield spectrometer (Rheinstetten, Germany) operating at 400/100 MHz (^1^H/^13^C) and using DMSO-*d*_6_, (CD_3_)­OD or CDCl_3_, as solvent and tetramethylsilane (TMS) as reference. Chemical shifts (∂) were reported in parts per million (ppm), coupling constants (*J*) were given in hertz (Hz) and multiplicities detected in ^1^H-RMN were expressed as singlet (s), broad singlet (bs), doublet (d), doublet of doublets (dd), triplet (t), doublet of triplets (dt), quadruplet (q), and multiplet (m). The purity of compounds was confirmed by qNMR (purity ≥95%).^62^ Melting points (Mp) were determined on a Mettler FP82 P FP80 apparatus (Greifensee, Switzerland).

### 5.2 General Procedure for the Synthesis of Compounds

#### 5.2.1 Synthesis of Intermediate Hydrazinecarboselenoamide (**I3**)

The synthesis of the intermediate **I3**, was performed in two steps as described by Chen et al.^63^ with some modifications. First, commercially available hydrazinecarbotioamide (**I1**) was methylated using excess of methyl iodide. In a second step, anhydrous EtOH (20 mL) and *Se* (11 mmol) were mixed on ice under nitrogen atmosphere. Sodium borohydride (22 mmol) was then added portion wise until reaction turned colorless. Finally, freshly synthesized hydrazineyl­(methylthio)­methanamine (**I2**) (11 mmol) was added and reaction was stirred at room temperature overnight. The intermediate **I3** was isolated by filtration, dried, and used without further purification.

#### 5.2.2 Synthesis of Arylidene Thiosemicarbazone Series 1 (***S*** **1 Series**)

The synthesis of ***S*** **1** derivatives, was modified from the method proposed by Matsa et al.^64^ Commercial **I1** (11 mmol) was dissolved in EtOH (40 mL). Then, the corresponding methyl ketone (11 mmol) and glacial acetic acid as a catalyst (0.5 mL), were added. The mixture was heated at reflux for 3 h and cooled down to room temperature. The reaction was monitored by TLC, using Hex/AcOEt [6:4] as a mobile phase. The resulting precipitate was filtered and washed with hexane and diethyl ether. When needed, the obtained solid was purified by recrystallization.

#### 5.2.3 Synthesis of Arylidene Selenosemicarbazone Series 1 (***Se*** **1 Series**)

The synthesis of ***Se*** **1** derivatives, was modified from the method proposed by Zaharia et al.^65^ The synthesized **I3** (7.24 mmol) was dissolved in a mixture of EtOH and glacial acetic acid [4:3] (7 mL). The corresponding methyl ketone (7.24 mmol) was dissolved in EtOH (5 mL) and added to the reaction mixture. Then, it was heated at reflux and stirred for 4 h until the formation of the corresponding ***Se*** **1** derivative. The reaction was confirmed by TLC using Hex/AcOEt [6:4] as mobile phase and hot reaction mixture was eventually filtered to remove *Se* excess. The crude material was allowed to precipitate at room temperature. If no precipitate appeared, the solvent was removed under reduced pressure and the obtained solid was extracted in AcOEt/NaHCO_3_ (3 × 20 mL), dried over anhydrous Na_2_SO_4_, filtered off and concentrated under reduce pressure. The final compound was purified, if needed, by recrystallization.

#### 5.2.4 Synthesis of Thio- and Selenazole Series 2 (***S*** **2**, ***Se*** **2 Series**)

Synthetic route for cyclic compounds was based on Geisler et al.^66^ pathway with modifications. The corresponding ***Se*** **1** or ***S*** **1** derivative (1.06 mmol) was dissolved in dry acetone with a ratio 2 mmol:10 mL. Chloroacetone (1.26 mmol) was added to the solution and, the mixture was stirred and heated at reflux (1–4 h) until the formation of a new precipitate. The reaction was confirmed by TLC using Hex/AcOEt [6:4] as mobile phase. The reaction was cooled down to room temperature while stirring. Then, the obtained precipitate was collected by vacuum filtration and then washed with hexane. Finally, the solid was vacuum-dried to obtain the pure compound as hydrochloride.

### 5.3 Compounds Characterization

#### 5.3.1 Thiosemicarbazone Derivatives (***S*1**)

##### 5.3.1.1 (*E*)-2-(1-(4-Tolyl)­ethylidene)­hydrazine-1-carbothioamide (***S*1a**)

C_10_H_13_N_3_S. MW: 207.30. Yield: 30.26%. Mp: 150–153 °C. IR (ATR, cm^–1^): 3412, 3371, 3209, 3114 (*v*_N–H_); 1590 (*v*_C=N_); 1293 (*v*_C=S_). ^1^H NMR (DMSO-*d*_6_, 400 MHz) δ ppm: 10.09 (s, 1H, NH); 8.25 (s, 1H, NH_2_); 7.89 (s, 1H, NH_2_); 7.83 (d, 2H, H_2_+H_6_, *J*_2–6_ = *J*_6–2_ = 8.0 Hz); 7.19 (d, 2H, H_3_ + H_5_, *J*_3–5_ = *J*_5–3_ = 7.9 Hz); 2.33 (s, 3H, CH_3_); 2.28 (s, 3H, CH_3_). ^13^C NMR (DMSO-*d*_6_, 100 MHz) δ ppm: 178.82, 147.95, 138.83, 134.87, 128.84 (2C), 126.53 (2C), 20.82, 13.89. Purity (qNMR: 25 °C, DMSO-*d*_6_, *m*_s_ = 7.31 mg, m_IC_ (sulfonamide) = 2.27 mg): 95.41%.

##### 5.3.1.2 (*E*)-2-(1-(4-Methoxyphenyl)­ethylidene)­hydrazine-1-carbothioamide (***S*1b**)

C_10_H_13_N_3_OS. MW: 223.29. Yield: 51.63%. Mp: 171–174 °C. IR (ATR, cm^–1^): 3381, 3245, 3161 (*v*_N–H_); 1591, 1493 (*v*_C=N_); 1247, 1180 (*v*_C=S_). ^1^H NMR (DMSO-*d*_6_, 400 MHz) δ ppm: 10.06 (s, 1H, NH); 8.21 (s, 1H, NH_2_); 7.89 (d, 3H, NH + H_2_ + H_6_, *J*_2–6_ = *J*_6–2_ = 8.6 Hz); 6.93 (d, 2H, H_3_ + H_5_, *J*_3–5_ = *J*_5–3_ = 8.6 Hz); 3.79 (s, 3H, OCH_3_); 2.27 (s, 3H, CH_3_). ^13^C NMR (DMSO-*d*_6_, 100 MHz) δ ppm: 178.66, 160.25, 147.89, 130.08, 128.15 (2C), 113.59 (2C), 55.22, 13.87. Purity (qNMR: 25 °C, DMSO-*d*_6_, *m*_s_ = 8.95 mg, m_IC_ (sulfonamide) = 1.31 mg): 95.58%.

##### 5.3.1.3 (*E*)-2-(1-(4-Nitrophenyl)­ethylidene)­hydrazine-1-carbothioamide (***S*1c**)

C_9_H_10_N_4_O_2_S. MW: 238.27. Yield: 79.66%. Mp: 209.8–213.1 °C. IR (ATR, cm^–1^): 3480, 3357, 3193 (*v*_N–H_); 1579 (*v*_C=N_); 1500 (*v*_NO_2__) 1338 (*v*_C=S_). ^1^H NMR (DMSO-*d*_6_, 400 MHz) δ ppm: 10.26 (s, 1H, NH); 8.46 (s, 1H, NH_2_); 8.22 (d, 2H, H_3_ + H_5_, *J*_3–5_ = *J*_5–3_ = 9.1 Hz); 8.18 (d, 2H, H_2_ + H_6_, *J*_2–6_ = *J*_6–2_ = 9.0 Hz); 8.14 (s, 1H, NH_2_); 2.35 (s, 3H, CH_3_). ^13^C NMR (DMSO-*d*_6_, 100 MHz) δ ppm: 179.32, 147.41, 145.28, 143.91, 127.74 (2C), 123.25 (2C), 13.92. Purity (qNMR: 25 °C, DMSO-*d*_6_, *m*_s_ = 11.25 mg, *m*_IC_ (sulfonamide) = 2.99 mg): 95.16%. NMR data are in agreement with those reported in the literature.^13^

##### 5.3.1.4 (*E*)-2-(1-(4-(Trifluoromethyl)­phenyl)­ethylidene)­hydrazine-1-carbothioamide (***S*1d**)

C_10_H_10_F_3_N_3_S. MW: 261.27. Yield: 47.64%. Mp: 168.1–171.9 °C. IR (ATR, cm^–1^): 3442, 3263, 3155 (*v*_N–H_); 1593 (*v*_C=N_); 1313 (*v*_C=S_). ^1^H NMR (DMSO-*d*_6_, 400 MHz) δ ppm: 10.35 (s, 1H, NH); 8.39 (s, 1H, NH_2_); 8.15 (d, 2H, H_3_ + H_5_, *J*_3–5_ = *J*_5–3_ = 8.3 Hz); 8.06 (s, 1H, NH_2_); 7.71 (d, 2H, H_2_ + H_6_, *J*_2–6_ = *J*_6–2_ = 8.4 Hz); 2.33 (s, 3H, CH_3_). ^13^C NMR (DMSO-*d*_6_, 100 MHz) δ ppm: 179.22, 146.06, 141.62, 129.50–128.55 (1C, C_4_), 127.32 (2C), 125.59 + 122.88 (1C, CF_3_), 125.01, 124.97, 13.95. Purity (qNMR: 25 °C, DMSO-*d*_6_, *m*_s_ = 15.658 mg, *m*_IC_ (sulfonamide) = 5.048 mg): 97.69%.

##### 5.3.1.5 (*E*)-2-(1-(4-Cyanophenyl)­ethylidene)­hydrazine-1-carbothioamide (***S*1e**)

C_10_H_10_N_4_S. MW: 218.28. Yield: 84.5%. Mp: 217.7–220.2 °C. IR (ATR, cm^–1^): 3388, 3237, 3140 (*v*_N–H_); 2229 (*v*_C≡N_); 1580 (*v*_C=N_); 1086 (*v*_C=S_). ^1^H NMR (DMSO-*d*_6_, 400 MHz) δ ppm: 10.34 (s, 1H, NH); 8.40 (s, 1H, NH_2_); 8.14 (d, 2H, H_3_ + H_5_, *J*_3–5_ = *J*_5–3_ = 8.4 Hz); 8.12 (s, 1H, NH_2_); 7.82 (d, 2H, H_2_ + H_6_, *J*_2–6_ = *J*_6–2_ = 8.4 Hz); 2.31 (s, 3H, CH_3_). ^13^C NMR (DMSO-*d*_6_, 100 MHz) δ ppm: 179.23, 145.67, 141.99, 132.09 (2C), 127.35 (2C), 118.82, 111.22, 13.76. Purity (qNMR: 25 °C, DMSO-*d*_6_, *m*_s_ = 6.64 mg, *m*_IC_ (sulfonamide) = 9.71 mg): 100%.

##### 5.3.1.6 (*E*)-2-(1-(4-Bromophenyl)­ethylidene)­hydrazine-1-carbothioamide (***S*1f**)

C_9_H_10_BrN_3_S. MW: 272.16. Yield: 70.11%. Mp: 188.5–190.1 °C. IR (ATR, cm^–1^): 3409, 3193, 3137 (*v*_N–H_); 1586 (*v*_C=N_); 1392 (*v*_C=S_). ^1^H NMR (DMSO-*d*_6_, 400 MHz) δ ppm: 10.18 (s, 1H, NH); 8.31 (s, 1H, NH_2_); 7.99 (s, 1H, NH_2_); 7.90 (d, 2H, H_3_ + H_5_, *J*_3–5_ = *J*_5–3_ = 8.5 Hz); 7.55 (d, 2H, H_2_ + H_6_, *J*_2–6_ = *J*_6–2_ = 8.5 Hz); 2.28 (s, 3H, CH_3_). ^13^C NMR (DMSO-*d*_6_, 100 MHz) δ ppm: 179.01, 146.61, 136.88, 131.10 (2C), 128.68 (2C), 122.69, 13.78. Purity (qNMR: 25 °C, DMSO-*d*_6_, *m*_s_ = 11.57 mg, *m*_IC_ (sulfonamide) = 1.45 mg): 99.89%. NMR data are in agreement with those reported in the literature.^34^

##### 5.3.1.7 (*E*)-2-(1-(3-Methoxyphenyl)­ethylidene)­hydrazine-1-carbothioamide (***S*1g**)

C_10_H_13_N_3_OS. MW: 223.29. Yield: 86.74%. Mp: 186.8–188.7 °C. IR (ATR, cm^–1^): 3362, 3233, 3153 (*v*_N–H_); 1584 (*v*_C=N_); 1230 (*v*_C=S_). ^1^H NMR (DMSO-*d*_6_, 400 MHz) δ ppm: 10.11 (s, 1H, NH); 8.28 (s, 1H, NH_2_); 7.93 (s, 1H, NH_2_); 7.45 (d, 1H, H_6_, *J*_6–5_ = 8.0 Hz); 7.45 (s, 1H, H_2_); 7.29 (t, 1H, H_5_, *J*_5–6_ = 7.9 Hz); 6.96 (dd, 1H, H_4_, *J*_4–5_ = 8.8 Hz, *J*_4–2_ = 1.8 Hz); 3.80 (s, 3H, OCH_3_); 2.29 (s, 3H, CH_3_). ^13^C NMR (DMSO-*d*_6_, 100 MHz) δ ppm: 178.93, 159.28, 147.88, 139.16, 129.27, 119.15, 114.98, 111.85, 55.20, 14.23. Purity (qNMR: 25 °C, DMSO-*d*_6_, *m*_s_ = 9.96 mg, *m*_IC_ (sulfonamide) = 2.37 mg): 101.41%.

##### 5.3.1.8 (*E*)-2-(1-(3-Nitrophenyl)­ethylidene)­hydrazine-1-carbothioamide (***S*1h**)

C_9_H_10_N_4_O_2_S. MW: 238.27. Yield: 80.35%. Mp: 197.3–200.5 °C. IR (ATR, cm^–1^): 3401, 3196, 3140 (*v*_N–H_); 1593 (*v*_C=N_); 1500 (*v*_NO2_); 1345 (*v*_C=S_). ^1^H NMR (DMSO-*d*_6_, 400 MHz) δ ppm: 10.36 (s, 1H, NH); 8.60 (s, 1H, NH_2_); 8.42 (s, 1H, H_4_, *J*_4–5_ = *J*_4–6_ = *J*_5–6_ = 8.0 Hz); 8.39 (s, 1H, NH_2_); 8.20 (s, 1H, H_6_, *J*_4–5_ = *J*_4–6_ = *J*_5–6_ = 8.0 Hz); 8.15 (s, 1H, NH_2_); 7.66 (s, 1H, H_5_, *J*_4–5_ = *J*_4–6_ = *J*_5–6_ = 8.0 Hz); 2.36 (s, 3H, CH_3_). ^13^C NMR (DMSO-*d*_6_, 100 MHz) δ ppm: 179.23, 148.20, 145.61, 139.49, 132.95, 129.71, 123.59, 121.01, 14.06. Purity (qNMR: 25 °C, DMSO-*d*_6_, *m*_s_ = 10.12 mg, *m*_IC_ (sulfonamide) = 1.11 mg): 97.18%. NMR data literature.^54,67^

##### 5.3.1.9 (*E*)-2-(1-(3-Chlorophenyl)­ethylidene)­hydrazine-1-carbothioamide (***S*1i**)

C_9_H_10_ClN_3_S. MW: 227.71. Yield: 28.35%. Mp: 152–154.9 °C. IR (ATR, cm^–1^): 3392, 3194, 3137 (*v*_N–H_); 1589 (*v*_C=N_); 1292 (*v*_C=S_). ^1^H NMR (DMSO-*d*_6_, 400 MHz) δ ppm: 10.25 (s, 1H, NH); 8.32 (s, 1H, NH_2_); 8.11 (s, 1H, NH_2_); 8.07 (s, 1H, H_2_); 7.84 (d, 1H, H_6_, *J*_6–5_ = 7.1 Hz); 7.41 (q, 2H, H_4_ + H_5_, *J*_4–5_ = 8.7 Hz, *J*_5–6_ = 7.9 Hz); 2.29 (s, 3H, CH_3_). ^13^C NMR (DMSO-*d*_6_, 100 MHz) δ ppm: 179.05, 146.31, 139.81, 133.41, 130.00, 128.91, 126.14, 125.35, 13.95. Purity (qNMR: 25 °C, DMSO-*d*_6_, *m*_s_ = 10.84 mg, *m*_IC_ (sulfonamide) = 3.66 mg): 97.24%. NMR data are in agreement with those reported in the literature.^13,34^

##### 5.3.1.10 (*E*)-2-(1-(3,4-Difluorophenyl)­ethylidene)­hydrazine-1-carbothioamide (***S*1j**)

C_9_H_9_F_2_N_3_S. MW: 229.05. Yield: 44.62%. Mp: 176.3–179 °C. IR (ATR, cm^–1^): 3414, 3200, 3150 (*v*_N–H_); 1595 (*v*_C=N_); 1183 (*v*_C=S_). ^1^H NMR (DMSO-*d*_6_, 400 MHz) δ ppm: 10.24 (s, 1H, NH); 8.31 (s, 1H, NH_2_); 8.18 (dd, 1H, H_6_, *J*_6‑F_ = 12.9 Hz, *J*_6‑F_ = 8.2 Hz); 8.14 (s, 1H, NH_2_); 7.71 (d, 1H, H_2_, *J*_2‑F_ = 10.7 Hz); 7.41 (m, 1H, H_5_); 2.28 (s, 3H, CH_3_). ^13^C NMR (DMSO-*d*_6_, 100 MHz) δ ppm: 179.03, 151.37–150.67 (1C, C_4_), 148.90–148.24 (1C, C_3_), 145.54, 135.31, 123.69, 117.12, 115.47, 13.80. Purity (qNMR: 25 °C, DMSO-*d*_6_, *m*_s_ = 12.05 mg, *m*_IC_ (sulfonamide) = 2.96 mg): 95.22%.

##### 5.3.1.11 (*E*,*Z*)-2-(1-(2,4-Dimethoxyphenyl)­ethylidene)­hydrazine-1-carbothioamide (***S*1k**)

C_11_H_15_N_3_O_2_S. MW: 253.32. Yield: 72.07%. Mp: 157.2–158.3 °C. IR (ATR, cm^–1^): 3375, 3226, 3148 (*v*_N–H_); 1597 (*v*_C=N_); 1206 (*v*_C=S_). *E* isomer (73%): ^1^H NMR (DMSO-*d*_6_, 400 MHz) δ ppm: 10.08 (s, 1H, NH); 8.14 (s, 1H, NH_2_); 7.60 (s, 1H, NH_2_); 7.40 (d, 1H, H_6_, *J*_6–5_ = 8.5 Hz), 6.60 (d, 1H, H_3_, *J*_3–5_ = 2.3 Hz), 6.52 (dd, 1H, H_5_, *J*_5–6_ = 8.5, *J*_3–5_ = 2.3 Hz), 3.80 (s, 1H, OCH_3_); 3.79 (s, 1H, OCH_3_); 2.20 (s, 1H, CH_3_). *Z* isomer (27%): ^1^H NMR (DMSO-*d*_6_, 400 MHz) δ ppm: 8.31 (s, 1H, NH); 8.26 (s, 1H, NH_2_); 7.83 (s, 1H, NH_2_); 7.14 (d, 1H, H_6_, *J*_5–6_ = 8.4 Hz), 6.74 (d, 1H, H_3_, *J*_3–5_ = 2.2 Hz), 6.68 (dd, 1H, H_5_, *J*_5–6_ = 8.4, *J*_3–5_ = 2.2 Hz), 3.83 (s, 1H, OCH_3_); 3.81 (s, 1H, OCH_3_); 2.16 (s, 1H, CH_3_). *E* isomer: ^13^C NMR (DMSO-*d*_6_, 100 MHz) δ ppm: 179.24, 161.82, 159.04, 150.83, 130.96, 121.72, 105.58, 99.09, 56.11, 55.84, 18.58. *Z* isomer: ^13^C NMR (DMSO-*d*_6_, 100 MHz) δ ppm: 178.03, 162.28, 157.07, 148.66, 129.41, 115.02, 106.70, 99.69, 56.34, 55.94, 24.78. Purity (qNMR: 25 °C, DMSO-*d*_6_, *m*_s_ = 8.27 mg, *m*_IC_ (sulfonamide) = 2.70 mg): 95.86%.

##### 5.3.1.12 (*E*)-2-(1-(Thiophen-3-yl)­ethylidene)­hydrazine-1-carbothioamide (***S*1l**)

C_7_H_9_N_3_S_2_. MW: 199.29. Yield: 35.04%. Mp: 100.3–102.6 °C. IR (ATR, cm^–1^): 3373, 3265, 3166 (*v*_N–H_); 1612, 1593 (*v*_C=N_); 1088 (*v*_C=S_). ^1^H NMR (DMSO-*d*_6_, 400 MHz) δ ppm: 10.08 (s, 1H, NH); 8.22 (s, 1H, NH_2_); 7.94 (d, 1H, H_5_, J_5–2_ = 2.6 Hz), 7.93 (s, 1H, NH_2_); 7.83 (d, 1H, H_4_, *J*_4–2_ = 5.1 Hz); 7.51 (dd, 1H, H_2_, *J*_2–4_ = 5.1, J_2–5_ = 2.8 Hz); 2.27 (s, 1H, CH_3_). ^13^C NMR (DMSO-*d*_6_, 100 MHz) δ ppm: 178.65, 144.70, 141.07, 126.52, 126.31, 125.73, 14.55. Purity (qNMR: 25 °C, MeOD, *m*_s_ = 8.44 mg, *m*_IC_ (sulfonamide) = 2.15 mg): 97.29%.

##### 5.3.1.13 (*E*)-2-(1-(3-Chlorophenyl)­propan-2-ylidene)­hydrazine-1-carbothioamide (***S*1m**)

C_10_H_12_ClN_3_S. MW: 241.74. Yield: 32.00%. Mp: 119.6–121.6 °C. IR (ATR, cm^–1^): 3388, 3280, 3189 (*v*_N–H_); 1599 (*v*_C=N_); 1183 (*v*_C=S_). ^1^H NMR (DMSO-*d*_6_, 400 MHz) δ ppm: 10.01 (s, 1H, NH); 8.12 (s, 1H, NH_2_); 7.63 (s, 1H, NH_2_); 7.34 (m, 2H, H_4_ + H_5_); 7.31 (s, 1H, H_2_); 7.23 (d, 1H, H_6_, *J*_6–5_ = 7.4 Hz); 3.53 (s, 2H, CH_2_); 1.83 (s, 3H, CH_3_). ^13^C NMR (DMSO-*d*_6_, 100 MHz) δ ppm: 178.89, 152.11, 139.83, 133.08, 130.32, 128.77, 127.75, 126.63, 43.87, 15.98. Purity (qNMR: 25 °C, DMSO-*d*_6_, *m*_s_ = 11.22 mg, *m*_IC_ (sulfonamide) = 1.49 mg): 95.73%.

##### 5.3.1.14 (*E*)-2-(1-(4-Methoxyphenyl)­propan-2-ylidene)­hydrazine-1-carbothioamide (***S*1n**)

C_11_H_15_N_3_OS. MW: 237.32. Yield: 47.41%. Mp: 123.8–124.9 °C. IR (ATR, cm^–1^): 3399, 3204, 3144 (*v*_N–H_); 1584 (*v*_C=N_); 1243 (*v*_C=S_). ^1^H NMR (DMSO-*d*_6_, 400 MHz) δ ppm: 9.99 (s, 1H, NH); 8.08 (s, 1H, NH_2_); 7.61 (s, 1H, NH_2_); 7.16 (d, 2H, H_3_ + H_5_, *J*_3–5_ = *J*_5–3_ = 8.5 Hz); 6.87 (d, 2H, H_2_ + H_6_, *J*_2–6_ = *J*_6–2_ = 8.4 Hz); 3.72 (s, 3H, OCH_3_); 3.44 (s, 2H, CH_2_); 1.80 (s, 3H, CH_3_). ^13^C NMR (DMSO-*d*_6_, 100 MHz) δ ppm: 178.78, 158.03, 153.14, 129.96 (2C), 128.97, 113.92 (2C), 55.00, 43.58, 15.77. Recrystallization from H_2_O:EtOH. Purity (qNMR: 25 °C, DMSO-*d*_6_, *m*_s_ = 10.61 mg, *m*_IC_ (sulfonamide) = 3.15 mg): 96.91%.

##### 5.3.1.15 (*E*)-2-(1-(Benzo­[*b*]­thiophen-3-yl)­ethylidene)­hydrazine-1-carbothioamide (***S*1o**)

C_11_H_11_N_3_S_2_. MW: 249.35. Yield: 61.89%. Mp: 180–182.3 °C. IR (ATR, cm^–1^): 3439, 3105 (*v*_N–H_); 1579, 1515 (*v*_C=N_); 1258 (*v*_C=S_). ^1^H NMR (DMSO-*d*_6_, 400 MHz) δ ppm: 10.33 (s, 1H, NH); 8.54 (s, 1H, NH_2_); 8.37 (s, 1H, NH_2_); 8.25 (s, 1H, H_2_); 8.02 (d, 1H, H_7_, *J*_7–6_ = 7.7 Hz); 7.44 (m, 3H, H_4_ + H_5_ + H_6_); 2.44 (s, 3H, CH_3_). ^13^C NMR (DMSO-*d*_6_, 100 MHz) δ ppm: 179.05, 147.16, 139.88, 135.96, 134.13, 130.37, 125.38, 125.11, 124.84, 122.87, 16.38. Purity (qNMR: 25 °C, DMSO-*d*_6_, *m*_s_ = 6.60 mg, *m*_IC_ (sulfonamide) = 1.98 mg): 95.39%.

##### 5.3.1.16 (*E*)-2-(1-(Benzofuran-2-yl)­ethylidene)­hydrazine-1-carbothioamide (***S*1p**)

C_11_H_11_N_3_OS. MW: 233.29. Yield: 55.36%. Mp: 183–184 °C. IR (ATR, cm^–1^): 3308, 3230, 3138 (*v*_N–H_); 1590, 1564 (*v*_C=N_); 1243, 1176 (*v*_C=S_). ^1^H NMR (DMSO-*d*_6_, 400 MHz) δ ppm: 10.48 (s, 1H, NH); 8.43 (s, 1H, NH_2_); 7.85 (s, 1H, NH_2_); 7.66 (d, 1H, H_7_, *J*_7–6_ = *J*_6–7_ = 7.6 Hz,), 7.60 (d, 1H, H_4_, *J*_4–5_ = 8.2 Hz); 7.56 (s, 1H, H_3_); 7.35 (t, 1H, H_6_, *J*_6–7_ = *J*_7–6_ = 7.6 Hz); 7.27 (t, 1H, H_5_, *J*_5–6_ = 7.4 Hz); 2.34 (s, 1H, CH_3_). ^13^C NMR (DMSO-*d*_6_, 100 MHz) δ ppm: 179.00, 154.50, 153.66, 139.52, 128.12, 125.56, 123.39, 121.62, 111.33, 106.73, 13.46. Purity (qNMR: 25 °C, DMSO-*d*_6_, *m*_s_ = 9.66 mg, *m*_IC_ (sulfonamide) = 1.98 mg): 95.65%.

##### 5.3.1.17 (*E*)-2-(1-((3*r*,5*r*,7*r*)-Adamantan-1-yl)­ethylidene)­hydrazine-1-carbothioamide (**S1q**)

C_13_H_21_N_3_S. MW: 251.39. Yield: 59.80%. Mp: 185.5–189.1 °C. IR (ATR, cm^–1^): 3412, 3245, 3215, 3138 (*v*_N–H_); 2898, 2846 (*v*_C–H_); 1591 (*v*_C=N_); 1083 (*v*_C=S_). ^1^H NMR (DMSO-*d*_6_, 400 MHz) δ ppm: 9.82 (s, 1H, NH); 8.09 (s, 1H, NH_2_); 7.30 (s, 1H, NH_2_); 1.98 (bs, 3H, CH_adamantane_); 1.83 (s, 3H, CH_3_); 1.68 (m, 12H, CH_2adamantane_). ^13^C NMR (DMSO-*d*_6_, 100 MHz) δ ppm: 178.84, 159.51, 39.97, 38.87 (3C), 36.19 (3C), 27.63 (3C), 11.58. Purity (qNMR: 25 °C, DMSO-*d*_6_, *m*_s_ = 7.50 mg, *m*_IC_ (sulfonamide) = 2.37 mg): 99.02%.

#### 5.3.2 Selenosemicarbazone Derivatives (***Se*** 1)

##### 5.3.2.1 (*E*)-2-(1-(4-Tolyl)­ethylidene)­hydrazine-1-carboselenoamide (***Se*1a**)

C_10_H_13_N_3_Se. MW: 254.20. Yield: 21.27%. Mp: 153.4–155.7 °C. IR (ATR, cm^–1^): 3325, 3231, 3148 (*v*_N–H_); 1582 (*v*_C=N_); 1244 (*v*_C=Se_). ^1^H NMR (DMSO-*d*_6_, 400 MHz) δ ppm: 10.41 (s, 1H, NH); 8.68 (s, 1H, NH_2_); 8.38 (s, 1H, NH_2_); 7.85 (d, 2H, H_2_ + H_6_, *J*_2–6_ = *J*_6–2_ = 8.2 Hz); 7.19 (d, 2H, H_3_ + H_5_, *J*_3–5_ = *J*_5–3_ = 8.2 Hz); 2.32 (s, 3H, CH_3_); 2.27 (s, 3H, CH_3_). ^13^C NMR (DMSO-*d*_6_, 100 MHz) δ ppm: 174.55; 149.74; 139.15; 134.70; 128.87 (2C); 126.74 (2C); 20.86; 14.22. ^77^Se NMR (DMSO-*d*_6_, 76 MHz) δ ppm: 201.59. Recrystallization from EtOH:H_2_O. Purity (qNMR: 25 °C, DMSO-*d*_6_, *m*_s_ = 3.20 mg, *m*_IC_ (sulfonamide) = 2.84 mg): 95.27%.

##### 5.3.2.2 (*E*)-2-(1-(4-Methoxyphenyl)­ethylidene)­hydrazine-1-carboselenoamide (***Se*1b**)

C_10_H_13_N_3_Ose. MW: 270.19. Yield: 39.41%. Mp: 170.4–171.3 °C. IR (ATR, cm^–1^): 3364, 3237, 3140 (*v*_N–H_); 1587 (*v*_C=N_); 1246 (*v*_C=Se_). ^1^H NMR (DMSO-*d*_6_, 400 MHz) δ ppm: 10.37 (s, 1H, NH); 8.64 (s, 1H, NH_2_); 8.36 (s, 1H, NH_2_); 7.91 (d, 2H, H_2_ + H_6_, *J*_2–6_ = *J*_6–2_ = 8.9 Hz); 6.92 (d, 2H, H_3_ + H_5_, *J*_3–5_ = *J*_5–3_ = 8.9 Hz); 3.79 (s, 3H, OCH_3_); 2.27 (s, 3H, CH_3_). ^13^C NMR (DMSO-*d*_6_, 100 MHz) δ ppm: 174.25; 160.44; 149.64; 129.86; 128.37 (2C); 113.59 (2C); 55.23; 13.15. ^77^Se NMR (DMSO-*d*_6_, 76 MHz) δ ppm: 198.57. Purity (qNMR: 25 °C, DMSO-*d*_6_, *m*_s_ = 8.51 mg, *m*_IC_ (sulfonamide) = 2.84 mg): 95.52%.

##### 5.3.2.3 (*E*)-2-(1-(4-Cyanophenyl)­ethylidene)­hydrazine-1-carboselenoamide (***Se*1e**)

C_10_H_10_N_4_Se. MW: 265.18. Yield: 8.2%. Mp: 219.4–220.4 °C. IR (ATR, cm^–1^): 3364, 3219, 3133 (*v*_N–H_); 2233 (*v*_C≡N_); 1580 (*v*_C=N_); 1085 (*v*_C=Se_). ^1^H NMR (DMSO-*d*_6_, 400 MHz) δ ppm: 10.62 (s, 1H, NH); 8.87 (s, 1H, NH_2_); 8.63 (s, 1H, NH_2_); 8.17 (d, 2H, H_3_ + H_5_, *J*_3–5_ = *J*_5–3_ = 8.5 Hz); 7.83 (d, 2H, H_2_ + H_6_, *J*_2–6_ = *J*_6–2_ = 8.5 Hz); 2.32 (s, 3H, CH_3_). ^13^C NMR (DMSO-*d*_6_, 100 MHz) δ ppm: 175.42, 147.39, 141.86, 132.12 (2C), 127.55 (2C), 118.80, 111.47, 14.11. ^77^Se NMR (DMSO-*d*_6_, 76 MHz) δ ppm: 217.04. Purity (qNMR: 25 °C, DMSO-*d*_6_, *m*_s_ = 8.91 mg, *m*_IC_ (sulfonamide) = 1.02 mg): 95.66%.

##### 5.3.2.4 (*E*)-2-(1-(4-Bromophenyl)­ethylidene)­hydrazine-1-carboselenoamide (***Se*1f**)

C_9_H_10_BrN_3_Se. MW: 319.06. Yield: 17.01%. Mp: 183.1–183.2 °C. IR (ATR, cm^–1^): 3406, 3254, 3152 (*v*_N–H_); 1587 (*v*_C=N_); 1265 (*v*_C=Se_). ^1^H NMR (DMSO-*d*_6_, 400 MHz) δ ppm: 10.51 (s, 1H, NH); 8.76 (s, 1H, NH_2_); 8.50 (s, 1H, NH_2_); 7.93 (d, 2H, H_3_ + H_5_, *J*_3–5_ = *J*_5–3_ = 8.7 Hz); 7.56 (d, 2H, H_2_ + H_6_, *J*_2–6_ = *J*_6–2_ = 8.7 Hz); 2.28 (s, 3H, CH_3_). ^13^C NMR (DMSO-*d*_6_, 100 MHz) δ ppm: 174.94, 148.38, 136.72, 131.13 (2C), 128.87 (2C), 122.99, 14.11. ^77^Se NMR (DMSO-*d*_6_, 76 MHz) δ ppm: 207.67. Purity (qNMR: 25 °C, DMSO-*d*_6_, *m*_s_ = 3.51 mg, *m*_IC_ (sulfonamide) = 0.49 mg): 97.67%.

##### 5.3.2.5 (*E*)-2-(1-(3-Methoxyphenyl)­ethylidene)­hydrazine-1-carboselenoamide (***Se*1g**)

C_10_H_13_N_3_Ose. MW: 270.19. Yield: 25.1%. Mp: 181–183.5 °C. IR (ATR, cm^–1^): 3351, 3237, 3148 (*v*_N–H_); 1582 (*v*_C=N_); 1228 (*v*_C=Se_). ^1^H NMR (DMSO-*d*_6_, 400 MHz) δ ppm: 10.43 (s, 1H, NH); 8.73 (s, 1H, NH_2_); 8.43 (s, 1H, NH_2_); 7.49–7.47 (m, 2H, H_2_ + H_6_); 7.30 (t, 1H, H_5_, *J*_5–4_ = 8.1 Hz, *J*_5–6_ = 7.3 Hz) 6.98 (d, 1H, H_4_, *J*_4–5_ = 6.4 Hz); 3.80 (s, 3H, CH_3_); 2.29 (s, 3H, CH_3_). ^13^C NMR (DMSO-*d*_6_, 100 MHz) δ ppm: 174.75, 159.29, 149.69, 138.98, 129.30, 119.35, 115.27, 111.99, 55.23, 14.56. ^77^Se NMR (DMSO-*d*_6_, 76 MHz) δ ppm: 204.79. Purity (qNMR: 25 °C, DMSO-*d*_6_, *m*_s_ = 6.66 mg, *m*_IC_ (sulfonamide) = 0.48 mg): 104.38%.

##### 5.3.2.6 (*E*)-2-(1-(3-Nitrophenyl)­ethylidene)­hydrazine-1-carboselenoamide (***Se*1h**)

C_9_H_10_N_4_O_2_Se. MW: 285.17. Yield: 51.74%. Mp: 182.8–184.8 °C. IR (ATR, cm^–1^): 3375, 3185, 3127 (*v*_N–H_); 1593 (*v*_C=N_); 1500 (*v*_NO_2__); 1273 (*v*_C=Se_). ^1^H NMR (DMSO-*d*_6_, 400 MHz) δ ppm: 10.64 (s, 1H, NH); 8.85 (s, 1H, NH_2_); 8.67 (s, 1H, NH_2_); 8.64 (t, 1H, H_2_, *J*_2–6_ = 1.8 Hz); 8.44 (d, 1H, H_4_, *J*_4–5_ = *J*_4–6_ = *J*_5–6_= 8.0 Hz); 8.23 (dd, 1H, H_6_, *J*_4–5_ = *J*_4–6_ = *J*_5–6_ = 8.0, *J*_6–2_ = 1.8 Hz); 7.67 (t, 1H, H_5_, *J*_4–5_ = *J*_4–6_ = *J*_5–6_ = 8.0 Hz); 2.37 (s, 3H, CH_3_). ^13^C NMR (DMSO-*d*_6_, 100 MHz) δ ppm: 175.31, 148.22, 147.38, 139.34, 133.14, 129.74, 123.84, 121.22, 14.42. ^77^Se NMR (DMSO-*d*_6_, 76 MHz) δ ppm: 213.09. Purity (qNMR: 25 °C, DMSO-*d*_6_, *m*_s_ = 9.05 mg, *m*_IC_ (sulfonamide) = 0.68 mg): 95.03%.

##### 5.3.2.7 (*E*)-2-(1-(3,4-Difluorophenyl)­ethylidene)­hydrazine-1-carboselenoamide (***Se*1j**)

C_9_H_9_F_2_N_3_Se. MW: 273.15. Yield: 30.17%. Mp: 176.8–179 °C. IR (ATR, cm^–1^): 3405, 3241, 3148 (*v*_N–H_); 1593 (*v*_C=N_); 1185 (*v*_C=Se_). ^1^H NMR (DMSO-*d*_6_, 400 MHz) δ ppm: 10.50 (s, 1H, NH); 8.78 (s, 1H, NH_2_); 8.63 (s, 1H, NH_2_); 8.23 (m, 1H, H_6_); 7.74 (m, 1H, H_2_); 7.43 (m, 1H, H_5_); 2.28 (s, 3H, CH_3_). ^13^C NMR (DMSO-*d*_6_, 100 MHz) δ ppm: 174.99, 151.53–150.68 (1C), 149.06–148.26 (1C), 147.29, 135.24, 123.97, 117.17, 115.66, 14.13. ^77^Se NMR (DMSO-*d*_6_, 76 MHz) δ ppm: 208.17. Recrystallization from EtOH:H_2_O. Purity (qNMR: 25 °C, DMSO-*d*_6_, *m*_s_ = 7.29 mg, *m*_IC_ (sulfonamide) = 0.70 mg): 101.91%.

##### 5.3.2.8 (*E*,*Z*)-2-(1-(2,4-Dimethoxyphenyl)­ethylidene)­hydrazine-1-carboselenoamide (***Se*1k**)

C_11_H_15_N_3_O_2_Se. MW: 300.22. Yield: 24.86%. Mp: 142.8–143.5 °C. IR (ATR, cm^–1^): 3406, 3339, 3235 (*v*_N–H_); 1599 (*v*_C=N_); 1275 (*v*_C=Se_). *E* isomer (65%): ^1^H NMR (DMSO-*d*_6_, 400 MHz) δ ppm: 10.34 (s, 1H, NH); 8.58 (s, 1H, NH_2_); 8.09 (s, 1H, NH_2_); 7.43 (d, 1H, H_6_, *J*_5–6_ = 8.5 Hz), 6.60 (d, 1H, H_3_, *J*_5–3_ = 2.3 Hz), 6.51 (dd, 1H, H_5_, *J*_5–6_ = 8.5 Hz, *J*_5–3_ = 2.4 Hz), 3.80 (s, 3H, OCH_3_); 3.78 (s, 3H, OCH_3_); 2.20 (s, 3H, CH_3_). *Z* isomer (35%): ^1^H NMR (DMSO-*d*_6_, 400 MHz) δ ppm: 8.77 (s, 1H, NH); 8.54 (s, 1H, NH_2_); 8.35 (s, 1H, NH_2_); 7.15 (d, 1H, H_6_, *J*_5–6_ = 8.4 Hz), 6.74 (d, 1H, H_3_, *J*_5–3_ = 2.3 Hz), 6.68 (dd, 1H, H_5_, *J*_5–6_ = 8.4 Hz, *J*_5–3_ = 2.3 Hz), 3.83 (s, 3H, OCH_3_); 3.81 (s, 3H, OCH_3_); 2.15 (s, 3H, CH_3_). *E* isomer: ^13^C NMR (DMSO-*d*_6_, 100 MHz) δ ppm: 174.37, 161.50, 158.62, 152.30, 130.59, 121.00, 105.13, 98.61, 55.66, 55.38, 18.38. *Z* isomer: ^13^C NMR (DMSO-*d*6, 100 MHz) δ ppm: 173.44, 161.91, 156.52, 149.73, 128.97, 114.45, 106.28, 99.20, 55.94, 55.48, 24.33. ^77^Se NMR (DMSO-*d*_6_, 76 MHz) δ ppm: 197.10; 195.55. Purity (qNMR: 25 °C, DMSO-*d*_6_, *m*_s_ = 9.303 mg, *m*_IC_ (sulfonamide) = 2.076 mg): 101.97%.

##### 5.3.2.9 (*E*)-2-(1-(Thiophen-3-yl)­ethylidene)­hydrazine-1-carboselenoamide (***Se*1l**)

C_7_H_9_N_3_SSe. MW: 246.19. Yield: 12.11%. Mp: 137.5–138.4 °C. IR (ATR, cm^–1^): 3200, 3125 (*v*_N–H_); 1579 (*v*_C=N_); 1246 (*v*_C=Se_). ^1^H NMR (DMSO-*d*_6_, 400 MHz) δ ppm: 10.34 (s, 1H, NH); 8.67 (s, 1H, NH_2_); 8.42 (s, 1H, NH_2_); 8.01 (s, 1H, H_2_); 7.85 (d, 1H, H_5_, *J*_2–5_ = 4.7 Hz); 7.53 (bs, 1H, H_4_); 2.27 (s, 3H, CH_3_). ^13^C NMR (DMSO-*d*_6_, 100 MHz) δ ppm: 174.39; 146.32; 140.91; 126.61; 126.37 (2C); 14.83. ^77^Se NMR (DMSO-*d*_6_, 76 MHz) δ ppm: 201.01. Recrystallization from MeOH:H_2_O. Purity (qNMR: 25 °C, DMSO-*d*_6_, *m*_s_ = 2,522 mg, *m*_IC_ (sulfonamide) = 0.667 mg): 97.52%.

##### 5.3.2.10 (*E*)-2-(1-(Benzo­[*b*]­thiophen-3-yl)­ethylidene)­hydrazine-1-carboselenoamide (***Se*1o**)

C_11_H_11_N_3_SSe. MW: 296.25. Yield: 4.20%. Mp: 175.8 °C. IR (ATR, cm^–1^): 3364, 3206, 3131 (*v*_N–H_); 1579 (*v*_C=N_); 1258 (*v*_C=Se_).^1^H NMR (DMSO-*d*_6_, 400 MHz) δ ppm: 10.58 (s, 1H, NH); 8.80 (s, 1H, NH_2_); 8.53 (s, 1H, NH_2_); 8.32 (s, 1H, H_2_); 8.03 (d, 1H, H_7_, *J*_7–6_ = 7.7 Hz); 7.93 (s, 1H, H_4_); 7.48–7.40 (m, 2H, H_6_ + H_5_); 2.45 (s, 3H, CH_3_). ^13^C NMR (DMSO-*d*_6_, 100 MHz) δ ppm: 174.79; 149.24; 139.85; 135.89; 133.95; 131.11; 125.45; 125.14; 124.88; 122.86; 16.72. ^77^Se NMR (DMSO-*d*_6_, 76 MHz) δ ppm: 207.42. Recrystallization from MeOH:H_2_O. Purity (qNMR: 25 °C, DMSO-*d*_6_, *m*_s_ = 9.27 mg, *m*_IC_ (sulfonamide) = 3.89 mg): 95.41%.

##### 5.3.2.11 (*E*)-2-(1-(Benzofuran-2-yl)­ethylidene)­hydrazine-1-carboselenoamide (***Se* 1p**)

C_11_H_11_N_3_Ose. MW: 280.19. Yield: 14.55%. Mp: 182.9–183.0 °C. IR (ATR, cm^–1^): 3296, 3228, 3119 (*v*_N–H_); 1591 (*v*_C=N_); 1091 (*v*_C=Se_). ^1^H NMR (DMSO-*d*_6_, 400 MHz) δ ppm: 10.76 (s, 1H, NH); 8.89 (s, 1H, NH_2_); 8.36 (s, 1H, NH_2_); 7.68 (d, 1H, H_7_, *J*_7–4_ = 7.6 Hz); 7.63 (s, 1H, H_3_); 7.61 (d, 1H, H_4_, *J*_4–5_ = 8.4 Hz); 7.37 (t, 1H, H_6_, *J*_6–5_ = 7.1 Hz); 7.28 (t, 1H, H_5_, *J*_5–6_ = 7.3 Hz); 2.35 (s, 1H, CH_3_). ^13^C NMR (DMSO-*d*_6_, 100 MHz) δ ppm: 175.19; 154.57; 153.54; 141.14; 128.09; 125.71; 123.45; 121.71; 111.71; 107.25; 13.73. ^77^Se NMR (DMSO-*d*_6_, 76 MHz) δ ppm: 215.23. Recrystallization from MeOH:H_2_O. Purity (qNMR: 25 °C, DMSO-*d*_6_, *m*_s_ = 7.56 mg, *m*_IC_ (sulfonamide) = 1.99 mg): 96.37%.

##### 5.3.2.12 (*E*)-2-(1-((3*r*,5*r*,7*r*)-Adamantan-1-yl)­ethylidene)­hydrazine-1-carboselenoamide (***Se*1q**)

C_13_H_21_N_3_Se. MW: 298.29. Yield: 42.12%. Mp: 177.3–177.9 °C. IR (ATR, cm^–1^): 3468, 3402, 3331 (*v*_N–H_); 2897, 2884, 2843 (*v*_C–H_); 1584 (*v*_C=N_); 1246 (*v*_C=Se_). ^1^H NMR (DMSO-*d*_6_, 400 MHz) δ ppm: 10.12 (s, 1H, NH); 8.54 (s, 1H, NH_2_); 7.78 (s, 1H, NH_2_); 1.98 (bs, 3H, CH_adamantane_); 1.85 (s, 3H, CH_3_); 1.68 (m, 12H, CH_2adamantane_). ^13^C NMR (DMSO-*d*_6_, 100 MHz) δ ppm: 174.32, 161.67, 38.75 (3C), 36.17­(3C), 36.11, 27.61 (3C), 11.91. ^77^Se NMR (DMSO-*d*_6_, 76 MHz) δ ppm: 187.26. Recrystallization from MeOH:H_2_O. Purity (qNMR: 25 °C, DMSO-*d*_6_, *m*_s_ = 3.64 mg, *m*_IC_ (sulfonamide) = 0.88 mg): 100.55%.

#### 5.3.3 Thiazole Derivatives (***S*** 2)

##### 5.3.3.1 (*E*)-4-Methyl-2-(2-(1-(4-tolyl)­ethylidene)­hydrazineyl)­thiazole Hydrochloride (***S*2a**)

C_13_H_15_N_3_S.HCl. MW: 281.80. Yield: 26.34%. Mp: 96.4–97.4 °C. IR (ATR, cm^–1^): 3334 (*v*_N–H_), 2706 (*v*_HCl_), 1605 (*v*_C=N_). ^1^H NMR (MeOD, 400 MHz) δ ppm: 7.84 (d, 2H, H_2_ + H_6_); 7.27 (d, 2H, H_3_ + H_5_); 6.69 (s, 1H, H_thiazole_); 2.44 (s, 3H, OCH_3_); 2.39 (s, 3H, CH_3_); 2.36 (s, 3H, CH_3_). ^13^C NMR (MeOD, 100 MHz) δ ppm: 157.57, 147.92, 142.14, 139.14, 135.17, 130.26 (2C), 128.06 (2C), 105.13, 21.35, 14.98, 13.84. Purity (qNMR: 25 °C, MeOD, *m*_s_ = 4.92 mg, *m*_IC_ (sulfonamide) = 3.04 mg): 95.20%.

##### 5.3.3.2 (*E*)-2-(2-(1-(4-Methoxyphenyl)­ethylidene)­hydrazineyl)-4-methylthiazole Hydrochloride (***S*2b**)

C_13_H_15_N_3_OS.HCl. MW: 297.80. Yield: 62.44%. Mp: 91.8–92.9 °C. IR (ATR, cm^–1^): 3336 (*v*_N–H_), 2790 (*v*_HCl_), 1597 (*v*_C=N_). ^1^H NMR (MeOD, 400 MHz) δ ppm: 7.92 (d, 2H, H_3_ + H_5_, *J*_3–5_ = *J*_5–3_ = 8.8 Hz), 6.99 (d, 2H, H_2_ + H_6_, *J*_2–6_ = *J*_6–2_ = 8.8 Hz), 6.68 (s, 1H, H_thiazole_); 3.85 (s, 3H, OCH_3_); 2.43 (s, 3H, CH_3_); 2.36 (s, 3H, CH_3_). ^13^C NMR (MeOD, 100 MHz) δ ppm: 163.26, 157.45, 138.91, 138.79, 130.20, 129.76 (2C), 114.92 (2C), 104.96, 55.90, 14.86, 13.79. Purity (qNMR: 25 °C, MeOD, *m*_s_ = 10.62 mg, *m*_IC_ (sulfonamide) = 1.48 mg): 97.12%.

##### 5.3.3.3 (*E*)-4-Methyl-2-(2-(1-(4-nitrophenyl)­ethylidene)­hydrazineyl)­thiazole Hydrochloride (***S*2c**)

C_12_H_12_N_4_O_2_S.HCl. MW: 312.77. Yield: 2.44%. Mp: 176.2–178.8 °C. IR (ATR, cm^–1^): 3101 (*v*_N–H_), 2851 (*v*_HCl_), 1575 (*v*_C=N_). ^1^H NMR (DMSO-*d*_6_, 400 MHz) δ ppm: 11.42 (bs, 1H, HCl); 8.24 (d, 2H, H_3_ + H_5_, *J*_3–5_ = *J*_5–3_ = 8.9 Hz); 7.98 (d, 2H, H_2_ + H_6_, *J*_2–6_ = *J*_6–2_ = 8.9 Hz); 6.35 (s, 1H, H_thiazole_); 2.33 (s, 3H, CH_3_); 2.17 (s, 3H, CH_3_). ^13^C NMR (DMSO-*d*_6_, 100 MHz) δ ppm: 169.72, 146.81, 144.38, 130.92, 127.74, 126.37 (2C), 123.64 (2C), 123.24, 16.29, 13.70. Purity (qNMR: 25 °C, DMSO-*d*_6_, *m*_s_ = 5.08 mg, *m*_IC_ (sulfonamide) = 2.25 mg): 95.57%.

##### 5.3.3.4 (*E*)-4-Methyl-2-(2-(1-(4-(trifluoromethyl)­phenyl)­ethylidene)­hydrazineyl)­thiazole Hydrochloride (***S*2d**)

C_13_H_12_F_3_N_3_S.HCl. MW: 335.77. Yield: 77.29%. Mp: 86.7–89.2 °C. IR (ATR, cm^–1^): 3342 (*v*_N–H_), 2691 (*v*_HCl_), 1599 (*v*_C=N_). ^1^H NMR (MeOD, 400 MHz) δ ppm: 8.15 (d, 2H, H_3_ + H_5_, *J*_3–5_ = *J*_5–3_ = 8.2 Hz); 7.76 (d, 2H, H_2_ + H_6_, *J*_2–6_ = *J*_6–2_= 8.3 Hz); 6.76 (s, 1H, H_thiazole_); 2.51 (s, 3H, CH_3_); 2.39 (s, 3H, CH_3_). ^13^C NMR (MeOD, 100 MHz) δ ppm: 155.87, 141.70, 139.26, 139.14, 133.33–132.36 (1C), 128.75 (2C), 126.53–126.41 (2C), 124.15, 105.75, 15.10, 13.80. Purity (qNMR: 25 °C, MeOD, *m*_s_ = 10.73 mg, *m*_IC_ (sulfonamide) = 5.65 mg): 99.81%.

##### 5.3.3.5 (*E*)-4-(1-(2-(4-Methylthiazol-2-yl)­hydrazineylidene)­ethyl)­benzonitrile Hydrochloride (***S*2e**)

C_13_H_12_N_4_S.HCl. MW: 292.79. Yield: 4.80%. Mp: 171.9–174.3 °C. IR (ATR, cm^–1^): 3129 (*v*_N–H_), 2723 (*v*_HCl_), 1584 (*v*_C=N_). ^1^H NMR (MeOD, 400 MHz) δ ppm: 8.14 (d, 2H, H _3_ + H_5_, *J*_3–5_ = *J*_5–3_ = 8.7 Hz); 7.82 (d, 2H, H_2_ + H_6_, *J*_2–6_ = *J*_6–2_ = 8.8 Hz); 6.77 (s, 1H, H_thiazole_); 2.49 (s, 3H, CH_3_); 2.39 (s, 3H, CH_3_). ^13^C NMR (MeOD, 100 MHz) δ ppm: 155.27, 142.29 (2C), 139.46, 133.44 (2C), 128.86 (2C), 119.35, 114.63, 105.86, 14.85, 13.82. Purity (qNMR: 25 °C, DMSO-*d*_6_, *m*_s_ = 5.19 mg, *m*_IC_ (sulfonamide) = 0.84 mg): 95.75%.

##### 5.3.3.6 (*E*)-2-(2-(1-(4-Bromophenyl)­ethylidene)­hydrazineyl)-4-methylthiazole hydrochloride (***S*2f**)

C_12_H_12_BrN_3_O_2_S.HCl. MW: 346.67. Yield: 48.12%. Mp: 169.9–172 °C. IR (ATR, cm^–1^): 3056 (*v*_N–H_), 2758 (*v*_HCl_), 1617 (*v*_C=N_). ^1^H NMR (MeOD, 400 MHz) δ ppm: 7.88 (d, 2H, H_3_ + H_5_, *J*_3–5_ = *J*_5–3_ = 8.5 Hz); 7.61 (d, 2H, H_2_ + H_6_, *J*_2–6_ = *J*_6–2_ = 8.5 Hz, 1H); 6.73 (s, 1H, H_thiazole_); 2.45 (s, 3H, CH_3_); 2.37 (s, 3H, CH_3_). ^13^C NMR (MeOD, 100 MHz) δ ppm: 156.34, 139.14, 139.02, 137.04, 132.79 (2C), 129.86 (2C), 125.86, 105.49, 14.92, 13.77. Purity (qNMR: 25 °C, MeOD, m_s_ = 9.26 mg, m_IC_ (sulfonamide) = 4.06 mg): 98.03%.

##### 5.3.3.7 (*E*)-2-(2-(1-(3-Methoxyphenyl)­ethylidene)­hydrazineyl)-4-methylthiazole hydrochloride (***S*2g**)

C_13_H_15_N_3_OS.HCl. MW: 297.80. Yield: 89.00%. Mp: 179–181 °C. IR (ATR, cm^–1^): 3071 (*v*_N–H_), 2833 (*v*_HCl_), 1612 (*v*_C=N_). ^1^H NMR (CDCl_3_, 400 MHz) δ ppm: 7.34 (m, 3H, H_2_ + H_5_ + H_6_); 6.98 (m, 1H, H_4_); 6.23 (s, 1H, H_thiazole_); 3.85 (s, 3H, OCH_3_); 2.52 (s, 3H, CH_3_); 2.37 (s, 3H, CH_3_). ^13^C NMR (CDCl_3_, 100 MHz) δ ppm: 170.16, 159.86, 155.86, 137.78, 137.25, 129.79, 119.38, 116.04, 112.41, 102.43, 55.50, 16.00, 14.33. Purity (qNMR: 25 °C, CDCl_3_, *m*_s_ = 10.03 mg, *m*_IC_ (sulfonamide) = 2.58 mg): 98.10%.

##### 5.3.3.8 (*E*)-4-Methyl-2-(2-(1-(3-nitrophenyl)­ethylidene)­hydrazineyl)­thiazole Hydrochloride (***S*2h**)

C_12_H_12_N_4_O_2_S.HCl. MW: 312.77. Yield: 88.50%. Mp: 170–173 °C. IR (ATR, cm^–1^): 3131 (*v*_N–H_), 2827 (*v*_HCl_), 1608 (*v*_C=N_). ^1^H NMR (MeOD, 400 MHz) δ ppm: 8.81 (s, 1H, H_2_); 8.33 (t, 2H, H_4_ + H_6_, *J*_5–6_ = *J*_4–5_ = 7.9 Hz); 7.73 (t, 1H, H_5_, *J*_5–6_ = 8.0 Hz); 6.77 (s, 1H, H_thiazole_); 2.54 (s, 3H, CH_3_); 2.40 (s, 3H, CH_3_). ^13^C NMR (MeOD, 100 MHz) δ ppm: 169.64, 155.16, 150.03, 139.81, 139.21, 134.03, 131.02, 125.73, 122.69, 105.84, 15.15, 13.78. Purity (qNMR: 25 °C, MeOD, *m*_s_ = 8.28 mg, *m*_IC_ (sulfonamide) = 2.18 mg): 95.52%.

##### 5.3.3.9 (*E*)-2-(2-(1-(3-Chlorophenyl)­ethylidene)­hydrazineyl)-4-methylthiazole (***S*2i**)

C_12_H_12_ClN_3_S.HCl. MW: 302.22. Yield: 74.08%. Mp: 100.3–104.4 °C. IR (ATR, cm^–1^): 3427 (*v*_N–H_), 2928 (*v*_HCl_), 1575 (*v*_C=N_). ^1^H NMR (MeOD, 400 MHz) δ ppm: 8.08 (s, 1H, H_2_); 7.84 (d, 1H, H_6_, *J*_6–5_ = 7.1 Hz); 7.46 (q, 2H, H_4_ + H_5_, *J*_5–4_ = 8.7 Hz, *J*_5–6_ = 7.9 Hz); 6.74 (s, 1H, H_thiazole_); 2.46 (s, 3H, CH_3_); 2.38 (s, 3H, CH_3_). ^13^C NMR (MeOD, 100 MHz) δ ppm: 155.94, 139.98 (2C), 135.79 (2C), 131.36, 131.18, 127.89, 126.59, 105.50, 14.93, 13.76. Purity (qNMR: 25 °C, MeOD, *m*_s_ = 9.90 mg, *m*_IC_ (sulfonamide) = 3.52 mg): 96.77%.

##### 5.3.3.10 (*E*)-2-(2-(1-(3,4-Difluorophenyl)­ethylidene)­hydrazineyl)-4-methylthiazole Hydrochloride (***S*2j**)

C_12_H_11_F_2_N_3_S.HCl. MW: 303.76. Yield: 47.22%. Mp: 110.1–113 °C. IR (ATR, cm^–1^): 3342 (*v*_N–H_), 2833 (*v*_HCl_), 1588 (*v*_C=N_). ^1^H NMR (MeOD, 400 MHz) δ ppm: 8.04 (m, 1H, H_6_); 7.75 (d, 1H, H_2,_ *J*_2‑F_ = 12.7 Hz); 7.36 (q, 1H, H_5_, *J*_5–6_ = 8.6 Hz); 6.74 (s, 1H, H_thiazole_); 2.45 (s, 3H, CH_3_); 2.39 (s, 3H, CH_3_). ^13^C NMR (MeOD, 100 MHz) δ ppm: 155.15, 154.33–152.76 (1C), 151.83 + 151.70 (1C), 150.44 + 150.31 (1C), 139.18 + 139.06 (1C), 135.42, 125.17, 118.50 + 118.32 (1C), 117.17 + 116.98 (1C), 105.52, 14.84, 13.75. Purity (qNMR: 25 °C, MeOD, *m*_s_ = 11.54 mg, *m*_IC_ (sulfonamide) = 1.71 mg): 98.27%.

##### 5.3.3.11 (*E*)-2-(2-(1-(2,4-Dimethoxyphenyl)­ethylidene)­hydrazineyl)-4-methylthiazole Hydrochloride (***S*2k**)

C_14_H_17_N_3_O_2_S.HCl. MW: 327.83. Yield: 56.26%. Mp: 150–153.7 °C. IR (ATR, cm^–1^): 3334 (*v*_N–H_), 2838 (*v*_HCl_), 1593 (*v*_C=N_). ^1^H NMR (DMSO-*d*_6_, 400 MHz) δ ppm: 7.37 (d, 1 H, H_6_, *J*_5–6_ = 8.3 Hz); 6.65 (m, 2H, H_3_ + H_thiazole_); 6.58 (dd, 1H, H_5_, *J*_5–6_ = 8.5 Hz, *J*_5–3_ = 1.8 Hz); 3.83 (s, 3H, OCH_3_); 3.80 (s, 3H, OCH_3_); 2.34 (s, 3H, CH_3_); 2.23 (s, 3H, CH_3_). ^13^C NMR (DMSO-*d*_6_, 100 MHz) δ ppm: 161.88 (2C), 158.59 (2C), 130.55 (2C), 120.22, 105.25, 104.01, 98.72, 55.75, 55.47, 19.37, 14.15. Purity (qNMR: 25 °C, DMSO-*d*_6_, *m*_s_ = 16.42 mg, *m*_IC_ (sulfonamide) = 2.77 mg): 102.56%.

##### 5.3.3.12 (*E*)-4-Methyl-2-(2-(1-(thiophen-3-yl)­ethylidene)­hydrazineyl)­thiazole Hydrochloride (***S*2l**)

C_10_H_11_N_3_S_2_.HCl. MW: 273.80. Yield: 59.37%. Mp: 164.7–168.1 °C. IR (ATR, cm^–1^): 3064 (*v*_N–H_), 2818 (*v*_HCl_), 1582 (*v*_C=N_). ^1^H NMR (MeOD, 400 MHz) δ ppm: 7.99 (d, 1H, H_2_, *J*_2–5_ = 2.5 Hz); 7.78 (d, 1H, H_4_, *J*_4–5_ = 4.9 Hz); 7.48 (dd, 1H, H_5_, *J*_5–4_ = 5.1, *J*_5–2_ = 2.9 Hz); 6.69 (s, 1H, H_thiazole_); 2.45 (s, 3H, CH_3_); 2.37 (s, 3H, CH_3_). ^13^C NMR (MeOD, 100 MHz) δ ppm: 153.78, 140.88, 138.97, 138.85, 128.49, 127.53, 127.02, 105.10, 15.46, 13.74. Purity (qNMR: 25 °C, MeOD, *m*_s_ = 10.29 mg, *m*_IC_ (sulfonamide) = 3.59 mg): 97.91%.

##### 5.3.3.13 (*E*)-2-(2-(1-(Benzo­[*b*]­thiophen-3-yl)­ethylidene)­hydrazineyl)-4-methylthiazole Hydrochloride (***S*2o**)

C_14_H_13_N_3_S_2_.HCl. MW: 323.86. Yield: 66.72%. Mp: 165–168.8 °C. IR (ATR, cm^–1^): 3055 (*v*_N–H_), 2697 (*v*_HCl_), 1605 (*v*_C=N_). ^1^H NMR (MeOD, 400 MHz) δ ppm: 8.69 (d, 1H, H_7_, *J*_7–6_ = 8.1 Hz); 8.23 (s, 1H, H_2_); 7.94 (d, 1H, H_4_, *J*_4–5_ = 7.8 Hz); 7.46 (dt, 2H, H_6_ + H_5_, *J*_6–5_ = 7.2 Hz); 7.41 (s, 1H, H_thiazole_); 2.59 (s, 3H, CH_3_); 2.37 (s, 3H, CH_3_). ^13^C NMR (MeOD, 100 MHz) δ ppm: 155.47, 141.96 (2C), 137.21, 134.25, 132.96, 126.70, 126.21 (2C), 123.75 (2C), 105.53, 16.90, 13.86. Purity (qNMR: 25 °C, MeOD, *m*_s_ = 7.30 mg, *m*_IC_ (sulfonamide) = 1.53 mg): 95.92%.

##### 5.3.3.14 (*E*)-2-(2-(1-(Benzofuran-2-yl)­ethylidene)­hydrazineyl)-4-methylthiazole Hydrochloride (***S*2p**)

C_14_H_13_N_3_OS.HCl. MW: 307.80. Yield: 69.27%. Mp: 165.7–167.6 °C. IR (ATR, cm^–1^): 3571 (*v*_N–H_), 2768 (*v*_HCl_), 1595 (*v*_C=N_conj__). ^1^H NMR (MeOD, 400 MHz) δ ppm: 7.69 (d, 1H, H_4_, *J*_4–5_ = 7.7 Hz); 7.55 (d, 1H, H_7_, *J*_7–6_ = 8.3 Hz); 7.50 (s, 1H, H_3_); 7.41 (t, 1H, H_5_, *J*_5–4_ = 7.7 Hz), 7.29 (t, 1H, H_6_, *J*_6–5_ = 7.5 Hz), 6.75 (s, 1H, H_thiazole_); 2.47 (s, 3H, CH_3_); 2.39 (s, 3H, CH_3_). ^13^C NMR (MeOD, 100 MHz) δ ppm: 169.06, 156.93, 153.44, 148.18, 139.03, 129.43, 127.79, 124.79, 123.22, 112.32, 110.75, 105.72, 14.33, 13.72. Purity (qNMR: 25 °C, MeOD, *m*_s_ = 7.33 mg, *m*_IC_ (sulfonamide) = 2.30 mg): 96.52%.

##### 5.3.3.15 2-(2-((*E*)-1-((3*s*)-Adamantan-1-yl)­ethylidene)­hydrazineyl)-4-methylthiazole Hydrochloride (***S*2q**)

C_16_H_23_N_3_S.HCl. MW: 325.90. Yield: 52.35%. Mp: 90–92.2 °C. IR (ATR, cm^–1^): 3353 (*v*_N–H_), 2678 (*v*_HCl_), 1601 (*v*_C=N_conj__). ^1^H NMR (MeOD, 400 MHz) δ ppm: 6.64 (s, 1H, H_thiazole_); 2.34 (s, 3H, CH_3_); 2.07 (bs, 3H, CH_adamantane_); 2.01 (s, 3H, CH_3_); 2.07–1.74 (m, 12H, CH_2adamantane_). ^13^C NMR (MeOD, 100 MHz) δ ppm: 169.34, 138.83, 138.71, 105.07, 42.37, 40.39 (3C), 37.69 (3C), 29.64 (3C), 13.79, 12.67. Purity (qNMR: 25 °C, MeOD, m_s_ = 7.68 mg, m_IC_ (sulfonamide) = 2.61 mg): 95.65%.

#### 5.3.4 Selenazole Derivatives (***Se*2**)

##### 5.3.4.1 (*E*)-4-Methyl-2-(2-(1-(4-tolyl)­ethylidene)­hydrazineyl)-1,3-selenazole Hydrochloride (***Se*2a**)

C_13_H_15_N_3_Se.HCl. MW: 328.7. Yield: 59.06%. Mp: 152.0 °C. IR (ATR, cm^–1^): 3335 (*v*_N–H_), 2833 (*v*_HCl_), 1570 (*v*_C=N_). ^1^H NMR (MeOD, 400 MHz) δ ppm: 7.84 (d, 2H, H_2_ + H_6_, *J*_2–6_ = *J*_6–2_ = 7.7 Hz); 7.27 (d, 2H, H_3_ + H_5_, *J*_3–5_ = *J*_5–3_ = 8.1 Hz), 6.97 (s, 1H, H_selenazole_); 2.45 (s, 3H, OCH_3_); 2.39 (s, 3H, CH_3_); 2.32 (s, 3H, CH_3_). ^13^C NMR (MeOD, 100 MHz) δ ppm: 161.92, 158.15, 142.30, 138.47, 135.03, 130.30 (2C), 128.10 (2C), 107.27, 21.36, 15.22, 15.15. Purity (qNMR: 25 °C, MeOD, *m*_s_ = 3.414 mg, *m*_IC_ (sulfonamide) = 0.438 mg): 103.62%.

##### 5.3.4.2 (*E*)-2-(2-(1-(4-Methoxyphenyl)­ethylidene)­hydrazineyl)-4-methyl-1,3-selenazole Hydrochloride (***Se*2b**)

C_13_H_15_N_3_OSe.HCl. MW: 344.70. Yield: 78.54%. Mp: 95.3–97 °C. IR (ATR, cm^–1^): 3364 (*v*_N–H_), 2793 (*v*_HCl_), 1593 (*v*_C=N_). ^1^H NMR (CDCl_3_, 400 MHz) δ ppm: 7.72 (d, 2H, H_3_ + H_5_, *J*_3–5_ = *J*_5–3_ = 8.8 Hz), 6.92 (d, 2H, H_2_ + H_6_, *J*_2–6_ = *J*_6–2_ = 8.8 Hz), 6.59 (s, 1H, H_selenazole_); 3.85 (s, 3H, OCH_3_); 2.51 (s, 3H, CH_3_); 2.32 (s, 3H, CH_3_). ^13^C NMR (CDCl_3_, 100 MHz) δ ppm: 174.67, 161.79, 156.54, 137.16, 128.66, 128.40 (2C), 114.12 (2C), 103.60, 55.55, 16.12, 15.65. Purity (qNMR: 25 °C, CDCl_3_, *m*_s_ = 4.13 mg, *m*_IC_ (sulfonamide) = 0.61 mg): 98.31%.

##### 5.3.4.3 (*E*)-4-Methyl-2-(2-(1-(4-(trifluoromethyl)­phenyl)­ethylidene)­hydrazineyl)-1,3-selenazole Hydrochloride (***Se*2d**)

C_13_H_12_F_3_N_3_Se.HCl. MW: 382.67. Yield: 34.24%. Mp: 122.4–125.4 °C. IR (ATR, cm^–1^): 3330 (*v*_N–H_), 2710 (*v*_HCl_), 1593 (*v*_C=N_). ^1^H NMR (MeOD, 400 MHz) δ ppm: 8.15 (d, 2H, H_3_ + H_5_, *J*_3–5_ = *J*_5–3_ = 7.9 Hz); 7.76 (d, 2H, H_2_ + H_6_, *J*_2–6_ = *J*_6–2_ = 8.3 Hz); 7.04 (s, 1H, H_selenazole_); 2.52 (s, 3H, CH_3_); 2.34 (s, 3H, CH_3_). ^13^C NMR (MeOD, 100 MHz) δ ppm: 141.62, 138.53, 133.10, 132.78, 128.79 (2C), 126.84 (2C), 126.52, 126.48, 124.14, 15.23, 15.16. Purity (qNMR: 25 °C, MeOD, *m*_s_ = 4.66 mg, *m*_IC_ (sulfonamide) = 0.51 mg): 95.58%.

##### 5.3.4.4 (*E*)-2-(2-(1-(4-Bromophenyl)­ethylidene)­hydrazineyl)-4-methyl-1,3-selenazole Hydrochloride (***Se*2f**)

C_12_H_12_BrN_3_Se.HCl. MW: 393.57. Yield: 24.16%. Mp: 112.7–113.5 °C. IR (ATR, cm^–1^): 3350 (*v*_N–H_), 2702 (*v*_HCl_), 1597 (*v*_C=N_). ^1^H NMR (MeOD, 400 MHz) δ ppm: 7.82 (d, 2H, H_3_ + H_5_, *J*_3–5_ = *J*_5–3_ = 8.6 Hz); 7.58 (d, H_2_+H_6_, *J*_2–6_ = *J*_6–2_ = 8.7 Hz); 6.72 (s, 1H, H_selenazole_); 2.40 (s, 3H, CH_3_); 2.25 (s, 3H, CH_3_). ^13^C NMR (MeOD, 100 MHz) δ ppm: 155.44, 139.29, 139.27, 137.47, 132.72 (2C), 129.59 (2C), 125.43, 105.93, 15.54, 14.78. Purity (qNMR: 25 °C, MeOD, *m*_s_ = 3.25 mg, *m*_IC_ (sulfonamide) = 0.69 mg): 97.28%.

##### 5.3.4.5 (*E*)-2-(2-(1-(3-Methoxyphenyl)­ethylidene)­hydrazineyl)-4-methyl-1,3-selenazole Hydrochloride (***Se*2g**)

C_13_H_15_N_3_OSe.HCl. MW: 344.7. Yield: 78.91%. Mp: 173.8–177.8 °C. IR (ATR, cm^–1^): 3375 (*v*_N–H_), 2833 (*v*_HCl_), 1616 (*v*_C=N_). ^1^H NMR (CDCl_3_, 400 MHz) δ ppm: 7.31 (m, 3H, H_2_ + H_4_ + H_6_); 6.99 (m, 1H, H_5_), 6.63 (s, 1H, H_selenazole_); 3.85 (s, 3H, OCH_3_); 2.53 (s, 3H, CH_3_); 2.33 (s, 3H, CH_3_). ^13^C NMR (CDCl_3_, 100 MHz) δ ppm: 175.13, 159.85, 156.75, 137.67, 137.31, 129.82, 119.38, 116.01, 112.54, 103.96, 55.50, 16.47, 15.66. ^77^Se NMR (CDCl_3_, 76 MHz) δ ppm: 533.66 Purity (qNMR: 25 °C, CDCl_3_, *m*_s_ = 8.27 mg, *m*_IC_ (duroquinone) = 1.29 mg): 97.19%.

##### 5.3.4.6 (*E*)-4-Methyl-2-(2-(1-(3-nitrophenyl)­ethylidene)­hydrazineyl)-1,3-selenazole Hydrochloride (***Se*2h**)

C_12_H_12_N_4_O_2_Se.HCl. MW: 359.67. Yield: 66.88%. Mp: 175.3–176.11 °C. IR (ATR, cm^–1^): 3366 (*v*_N–H_), 2762 (*v*_HCl_), 1591 (*v*_C=N_). ^1^H NMR (DMSO-*d*_6_, 400 MHz) δ ppm: 8.63 (s, 1H, H_2_); 8.39 (bs, 1H, H_4_); 8.27 (d, 1H, H_6_, *J*_5–6_ = 7.8 Hz); 7.74 (t, 1H, H_5_, *J*_5–4_ = 8.0 Hz); 6.81 (s, 1H, H_selenazole_); 2.53 (s, 3H, CH_3_); 2.21 (s, 3H, CH_3_). ^13^C NMR (DMSO-*d*_6_, 100 MHz) δ ppm: 152.95, 148.57 (2C), 139.21, 137.04, 133.45, 130.50 (2C), 124.58, 121.41, 16.13, 15.87. Purity (qNMR: 25 °C, DMSO-*d*_6_, *m*_s_ = 11.359 mg, *m*_IC_ (sulfonamide) = 1.788 mg): 99.87%.

##### 5.3.4.7 (*E*)-2-(2-(1-(3-Chlorophenyl)­ethylidene)­hydrazineyl)-4-methyl-1,3-selenazole Hydrochloride (***Se*2i**)

C_12_H_12_ClN_3_Se.HCl. MW: 349.12. Yield: 7.35%. Mp: 147.5–150.2 °C. IR (ATR, cm^–1^): 3070 (*v*_N–H_), 2837 (*v*_HCl_), 1573 (*v*_C=N_). ^1^H NMR (MeOD, 400 MHz) δ ppm: 8.07 (bs, 1H, H_2_), 7.84 (d, 1H, H_4_, *J*_4–5_ = 7.3 Hz), 7.47 (m, 2H, H_5_ + H_6_), 7.01 (s, 1H, H_selenazole_); 2.46 (s, 3H, CH_3_); 2.33 (s, 3H, CH_3_). ^13^C NMR (MeOD, 100 MHz) δ ppm: 182.23, 178.14, 156.42, 139.88, 138.48, 135.80, 131.46, 131.21, 127.95, 126.65, 15.16, 15.10. Purity (qNMR: 25 °C, MeOD, *m*_s_ = 3.14 mg, *m*_IC_ (sulfonamide) = 2.60 mg): 95.62%.

##### 5.3.4.8 (*E*)-2-(2-(1-(3,4-Difluorophenyl)­ethylidene)­hydrazineyl)-4-methyl-1,3-selenazole Hydrochloride (***Se*2j**)

C_12_H_11_F_2_N_3_Se.HCl. MW: 350.66. Yield: 49.08%. Mp: 128–130 °C. IR (ATR, cm^–1^): 3368 (*v*_N–H_), 2827 (*v*_HCl_), 1586 (*v*_C=N_). ^1^H NMR (MeOD, 400 MHz) δ ppm: 8.04 (bs, 1H, H_2_); 7.76 (d, 1H, H_6_, *J*_6–5_ = 8.1 Hz); 7.36 (q, 1H, H_5_, *J*_5–6_ = 8.6 Hz); 7.02 (s, 1H, H_selenazole_); 2.46 (s, 3H, CH_3_); 2.34 (s, 3H, CH_3_). ^13^C NMR (MeOD, 100 MHz) δ ppm: 155.61 + 155.50 (1C), 154.40 + 154.27 (1C), 152.91 + 152.76 (1C), 151.90 + 151.77 (1C), 150.46 + 150.33 (1C), 138.45, 135.29, 125.31, 118.53 + 118.35 (1C), 117.25 + 117.05 (1C), 15.14, 14.94. Purity (qNMR: 25 °C, MeOD, *m*_s_ = 5.72 mg, *m*_IC_ (sulfonamide) = 0.81 mg): 95.01%.

##### 5.3.4.9 (*E*,*Z*)-2-(2-(1-(2,4-Dimethoxyphenyl)­ethylidene)­hydrazineyl)-4-methyl-1,3-selenazole Hydrochloride (***Se*2k**)

C_14_H_17_N_3_O_2_Se.HCl. MW: 374.73. Yield: 47.01%. Mp: 154.4 °C. IR (ATR, cm^–1^): 3125 (*v*_N–H_), 2810 (*v*_HCl_), 1595 (*v*_C=N_). *E* isomer (77%): ^1^H NMR (MeOD, 400 MHz) δ ppm: 7.46 (d, 1H, H_6_, *J*_5–6_ = 8.5 Hz); 6.91 (bs, 1H, H_selenazole_); 6.63 (d, 1H, H_3_, *J*_5–3_ = 2.3 Hz); 6.59 (m, 1H, H_5_); 3.89 (s, 3H, OCH_3_); 3.85 (s, 3H, OCH_3_); 2.39 (s, 3H, CH_3_); 2.27 (s, 3H, CH_3_). *Z* isomer (23%): ^1^H NMR (MeOD, 400 MHz) δ ppm: 7.14 (d, 1H, H_6_, *J*_5–6_ = 8.3 Hz); 6.86 (bs, 1H, H_selenazole_); 6.70 (q, 1H, H_5_, *J*_3–5_ = 2.2 Hz); 6.67 (d, 1H, H_3_, *J*_3–5_ = 2.2 Hz); 3.86 (s, 3H, OCH_3_); 3.84 (s, 3H, OCH_3_); 2.32 (s, 3H, CH_3_); 2.26 (s, 3H, OCH_3_). ^13^C NMR (MeOD, 100 MHz) δ ppm: 176.93, 164.45 (2C), 160.59 (2C), 158.46, 138.26, 132.02, 106.32, 99.54, 56.17, 56.02, 18.89, 15.15. Purity (qNMR: 25 °C, MeOD, *m*_s_ = 3.31 mg, *m*_IC_ (sulfonamide) = 0.58 mg): 102.69%.

##### 5.3.4.10 (*E*)-4-Methyl-2-(2-(1-(thiophen-3-yl)­ethylidene)­hydrazineyl)-1,3-selenazole Hydrochloride (***S*e2l**)

C_10_H_11_N_3_SSe.HCl. MW: 320.70. Yield: 72.95%. Mp: 148.8–149.9 °C. IR (ATR, cm^–1^): 3269 (*v*_N–H_), 2810 (*v*_HCl_), 1564 (*v*_C=N_), 1524 (*v*_C=C_arom__). ^1^H NMR (MeOD, 400 MHz) δ ppm: 8.01 (dd, 1H, H_2_, *J*_2–5_ = 2.9, *J*_2–4_ = 1.3 Hz); 7.77 (bs, 1H, H_4_); 7.49 (dd, 1H, H_5_, *J*_5–4_ = 5.1, *J*_2–5_ = 2.9 Hz); 6.97 (s, 1H, H_selenazole_); 2.46 (s, 3H, CH_3_); 2.32 (s, 3H, CH_3_). ^13^C NMR (MeOD, 100 MHz) δ ppm: 154.16, 140.76, 138.41, 128.72 (2C), 127.60, 126.98, 107.21, 15.61, 15.21. Purity (qNMR: 25 °C, MeOD, *m*_s_ = 4.67 mg, *m*_IC_ (sulfonamide) = 0.75 mg): 95.24%.

##### 5.3.4.11 (*E*)-2-(2-(1-(Benzo­[*b*]­thiophen-3-yl)­ethylidene)­hydrazineyl)-4-methyl-1,3-selenazole Hydrochloride (***Se*2o**)

C_14_H_13_N_3_SSe.HCl. MW: 370.76. Yield: 5.59%. Mp: 145.5–148.6 °C. IR (ATR, cm^–1^): 3066 (*v*_N–H_), 2712 (*v*_HCl_), 1607 (*v*_C=N_), 1500 (*v*_C=C_arom__). ^1^H NMR (MeOD, 400 MHz) δ ppm: 8.66 (d, 1H, H_7_, *J*_7–6_ = 8.1 Hz); 8.25 (s, 1H, H_2_); 7.95 (d, 1H, H_4_, *J*_4–5_ = 7.9 Hz), 7.52–7.42 (m, 2H, H_5_ + H_6_), 7.01 (s, 1H, H_selenazole_); 2.61 (s, 3H, CH_3_); 2.33 (s, 3H, CH_3_). ^13^C NMR (MeOD, 100 MHz) δ ppm: 156.19, 142.00 (2C), 138.67, 137.15, 134.13, 133.23, 126.54, 126.28 (2C), 126.21, 123.83, 17.19, 15.21. Purity (qNMR: 25 °C, MeOD, *m*_s_ = 5.13 mg, *m*_IC_ (sulfonamide) = 1.05 mg): 95.20%.

##### 5.3.4.12 (*E*)-2-(2-(1-(Benzofuran-2-yl)­ethylidene)­hydrazineyl)-4-methyl-1,3-selenazole Hydrochloride (***Se*2p**)

C_14_H_13_N_3_OSe.HCl. MW: 354.70. Yield: 79.75%. Mp: 155.4–157.3 °C. IR (ATR, cm^–1^): 3125 (*v*_N–H_), 2790 (*v*_HCl_), 1593 (*v*_C=N_). ^1^H NMR (MeOD, 400 MHz) δ ppm: 7.69 (d, 1H, H_4_, *J*_4–5_ = 7.8 Hz), 7.56 (d, 1H, H_7_, *J*_7–6_ = 8.4 Hz); 7.52 (s, 1H, H_3_); 7.42 (t, 1H, H_5_, *J*_5–4_ = 7.8 Hz), 7.30 (t, 1H, H_6_, *J*_6–5_ = 7.5 Hz), 7.03 (s, 1H, H_selenazole_); 2.49 (s, 3H, CH_3_); 2.34 (s, 3H, CH_3_). ^13^C NMR (MeOD, 100 MHz) δ ppm: 156.96, 153.33, 148.49, 138.49, 138.38, 129.42, 127.89, 124.83, 123.26, 112.33, 111.07, 108.06, 15.11, 14.44. Purity (qNMR: 25 °C, MeOD, *m*_s_ = 10.01 mg, *m*_IC_ (sulfonamide) = 0.84 mg): 102.20%.

##### 5.3.4.13 2-(2-((*E*)-1-((3*s*)-Adamantan-1-yl)­ethylidene)­hydrazineyl)-4-methyl-1,3-selenazole (***Se*2q**)

C_16_H_23_N_3_Se. MW: 336.34. Yield: 86.93%. Mp: 115.8–117 °C. IR (ATR, cm^–1^): 3343 (*v*_N–H_), 1597 (*v*_C=N_), 1508 (*v*_C=C_arom__). ^1^H NMR (CDCl_3_, 400 MHz) δ ppm: 6.54 (s, 1H, H_selenazole_); 2.30 (s, 3H, CH_3_); 2.06 (bs, 6H, CH_3_ + CH _adamantane_); 1.77–1.66 (m, 12H, CH_2adamantane_). ^13^C NMR (CDCl_3_, 100 MHz) δ ppm: 175.28, 168.53, 136.99, 103.32, 40.92, 39.32 (3C), 36.66 (3C), 28.11 (3C), 15.64, 13.72. Purity (qNMR: 25 °C, CDCl_3_, *m*_s_ = 3,07 mg, *m*_IC_ (sulfonamide) = 0.46 mg): 101.26%.

### 5.4 Cell-Based Assays

Antiparasitic activity and cytotoxicity of compounds were determined as described before (da Silva et al.,^68^ Boudreau et al.^69^). Mouse myoblasts cell line C2C12 (ATCCCRL-1772), were maintained in high glucose (4.5 g/L glucose) Dulbecco’s modified Eagle’s medium (DMEM Gibco), supplemented with 1% penicillin–streptomycin 10,000 U/mL (Gibco) and 5% fetal bovine serum (FBS, Sigma-Aldrich) at 37 °C with 5% CO_2_. parasites (CAI/72 strain, DTU TcI^35^) were maintained by weekly coinfection with C2C12 cells. For all activity assays, C2C12 cells were infected at a 10:1 parasite-to-cell ratio with culture-derived trypomastigotes, which were harvested from the supernatant of infected C2C12 cells. After adding compounds to 384-well black clear-bottom plates (Greiner cat. no. 781091), trypomastigotes (7000 parasites/well) and C2C12 cells (700 cells/well) were seeded in a total volume of 50 μL of DMEM per well in the 384-well plates. Compounds were first tested at 10 μM. Then, compounds with an antiparasitic activity equal to or higher than 50% were selected to be tested in 10 points, 2-fold serial dilution for thio-compounds and 3-fold serial dilution for seleno-compounds, starting at 10 μM to assess the dose–response and determine potency. After 48 h at 37 °C and 5% CO_2_, plates were fixed with 4% formaldehyde solution for at least 1 h, washed with 1× PBS, and stained with 5 μg/mL of 4′,6-diamidino-2-phenylindole (DAPI) for at least 1 h prior to reading. Plates were imaged with the automated ImageXpress MicroXL microscope (Molecular Devices) and analyzed by MetaXpress software (Molecular Devices) using a custom module optimized for this assay. Infection levels (parasites per host cell) were normalized to the positive control (40 μM BNZ) and negative control (DMSO). Antiparasitic activity (EC_50_) ± standard deviation (SD) and host cell cytotoxicity (C2C12 viability) values were determined from the DRC using Prism software (GraphPad Software, La Jolla, CA). Every assay has three biological replicates, each in triplicates (*n* = 9 data points).

### 5.5 Enzymatic Assays

#### 5.5.1 Cruzain Inhibition Assays

The screening assay was performed using 1 nM of Cz diluted in 0.1 M sodium acetate pH 5.5, 1 mM dithiothreitol (DTT), and 0.01% Triton X-100. Cz was expressed and purified as described in da Silva et al.^68^ The reaction starts after 10 min incubating the enzyme with the compounds (10 μM), when adding 2.5 μM of the substrate Z-FR-AMC final concentration (Z-Phe-Arg-amidomethylcoumarin, Sigma-Aldrich, C9521) diluted in the same assay buffer (final volume of 30 μL). The experiment was performed for 10 min. Then, IC_50_ values of compounds inhibiting more than 85% at 10 μM and active against (antiparasitic activity ≥50%) were calculated. The DRC was performed with at least nine different concentrations of the inhibitors, diluted by 2-fold serial dilution, ranging from 4 × 10^–6^ to 10 μM.

#### 5.5.2 Human Cathepsin L Inhibition Assays

Recombinant human cathepsin L (*h*CatL) was purchased from R&D Systems (952-CY) and activated according to the manufacturer’s protocol. The assay was modified from Ashhurst et al.,^70^ using 25 pM of the enzyme diluted in 40 mM sodium acetate pH 5.5, 5 mM dithiothreitol (DTT), 100 mM NaCl, 1 mM EDTA and 0.01% Triton X-100. Compounds were screened at 10 μM. The reaction starts after 15 min incubation period, when adding 25 μM of the substrate Z-FR-AMC (Z-Phe-Arg-amidomethylcoumarin, Sigma-Aldrich, C9521) diluted in the same assay buffer, reaching a final volume of 30 μL. The experiment was performed for 30 min. Later, IC_50_ values of compounds inhibiting more than 90% at 10 μM were calculated. The experiment was performed with at least nine different concentrations of the inhibitors, diluted by 2-fold serial dilution, ranging from 6 × 10^–4^ to 2.5 μM.

#### 5.5.3 General Considerations

All experiments were accomplished in a 384-well black microplate, using a Synergy HTX (Biotek) plate reader at 25 °C with absorption/emission wavelengths of 360/460 nm. All assays were performed in triplicate wells and DMSO was used as a vehicle control. Enzymatic activity was measured and normalized to DMSO controls, and the inhibitor E-64 (10 μM) was used as a positive control for all assays. All experiments were performed in triplicates, with at least two independent assays (*n* = 6 data points). IC_50_ values were determined through nonlinear regression. Obtained data were treated and analyzed using GraphPad Prism 9.0 (GraphPad Software, La Jolla, California).

### 5.6 Compound Handling

Stock solutions of synthesized compounds (100 and 10 mM), BNZ (Sigma-Aldrich) (40 mM), and K777 (20 mM), were prepared in 100% DMSO, in micro tubes and stored at −20 °C. For ***Se*** **1** and ***Se*** **2 series**, aliquots from initial stock were made to avoid freeze-and-thaw. Working stock solutions were prepared in culture media on the same day as the assay. At final concentration, all the sample wells for the antiparasitic assays contained <0.5% of DMSO.

### 5.7 Molecular Dynamics Simulations

The structure deposited in the Protein Data Bank (PDB) under the ID 3KKU^38^ was used to prepare the starting structure to run the MolecularDynamics Simulations (MD). The ligand of the 3KKU structure was replaced by the selenium derivative ***Se*** **2h** before running the MD simulation (See Supporting Information). Both the system preparation and the simulation were performed in the AMBER 18 suite software. The protocol for the system preparation and the MD simulations is detailed as follows. First, the system is neutralized by adding sodium ions and later immersed in a cubic box of 10 Å length, in each direction from the end of the protein, using TIP3P water parameters. The force fields used to obtain topography and coordinates files were ff14SB^71^ and GAFF.^72^ The first step of the simulation protocol followed to run the MD simulations is a minimization of the solvent molecules position only, keeping the solute atom positions restrained, and the second stage minimizes all the atoms in the simulation cell. Heating the system is the third step, which gradually raises the temperature 0 to 300 K under a constant volume (ntp = 0) and periodic boundary conditions. In addition, Harmonic restraints of 10 kcal/mol^–1^ were applied to the solute, and the Berendsen temperature coupling scheme^73^ was used to control and equalize the temperature. The time step was kept at 2 fs during the heating phase. Long-range electrostatic effects were modeled using the particle-mesh-Ewald method.^74^ The Lennard-Jones interactions cutoff was set at 8 Å. An equilibration step for 100 ps with a 2 fs time step at a constant pressure and temperature of 300 K was performed prior to the production stage. The trajectory production stage kept the equilibration step conditions and was prolonged for 500 ns longer at the 1 fs time step. In addition, the selenium derivative required a previous preparation step where the parameters and charges were generated by using the antechamber module of AMBER, using the GAFF force field and AM1-BCC method for charges.^75^

### 5.8 Evaluation of Antioxidant Activity

The antioxidant capacity of the selected *Se* compound was tested using the DPPH assay described by Astrain-Redin.^76^ The compound was tested at three different concentrations (0.06, 0.03, and 0.015 mg/mL) and we used two positive controls: ascorbic acid (ASC) and trolox (TRO). A solution of DPPH (2,2-diphenyl-1-picrylhydrazyl) in methanol (0.04 mg/mL, preserved in the dark) was prepared and 100 μL of this stock solution was added to 100 μL of tested compound solution. The color change, from purple (radical) to yellow (reduced), was measured at 517 nm at different time points (0, 5, 15, 30, 60, 90, and 120 min). All measurements were carried out in triplicate and recorded on a BioTek PowerWave XS spectrophotometer (Biotek). Data were collected using BioTek Gen5Microplate reader and Imager software (Agilent, version 3.12). Results are expressed as a percentage of inhibited DPPH ± standard error of the mean (SEM), calculated using the following formula

$$\%i\mathrm{nhibited}\;\mathrm{DPPH}=\frac{A_{control}-A_{sample}}{A_{control}}\times 100$$

where *A*_control_ refers to the absorbance of the negative control and *A*_sample_ refers to the absorbance of the tested compound.

### 5.9 Pharmacokinetic Study

Compound ***Se*** **2h** was administered intravenously (5 mg/kg in 10% dimethyl sulfoxide, 30% propylene glycol, 30% PEG 400, and 30% of 10.0% Tween 80 in DI water) and by oral gavage (50 mg/kg in PEG 400 with 5% 1-methyl-2-pyrrolidone) in two female Balb/c mice (6 weeks old) for each group. Blood samples (∼25 μL) were collected with heparinized capillary tubes *via* the eye vein on mice anesthetized with isoflurane precompound administration and in the following times post administration: 30 min, 1 h, 2 h, 4 h. Samples were immediately centrifuged for plasma collection (supernatant). The animal experiment was approved by the UC San Diego IACUC committee under the protocol S14187 and follow all the ethical standards. The material was submitted for mass spectrometry quantification with provided known amounts spiked in naïve plasma for reference curve.

## Supplementary Material







## Notes

Biological data; DRC against ; molecular dynamic data; antioxidant activity; IR, ^1^H NMR, ^13^C NMR, ^77^Se-NMR and qNMR spectra of final compounds; and bidimensional spectra of compounds ***S*** **1k** and ***Se*** **1k** (PDF)
PDB 3KKU starting crystal structure for molecular modeling (PDB)
Molecular formula strings (CSV)

## Abbreviations Used

BZ: Benznidazole
CC_50_: cytotoxic concentration 50
CD: Chagas disease
Cz: cruzain
DRC: dose–response curve
EC_50_: half maximal effective concentration
*h*CatL: human cathepsine L
IC_50_: half-maximal inhibitory concentration
MD: molecular dynamics
NTD: neglected tropical disease
*S*: sulfur
SD: standard deviation
*Se*: selenium
*Se*SC: selenosemicarbazone
*Se*Z: selenazole
SEM: standard error of the mean
SI: selectivity index
TSC: thiosemicarbazone
TZ: thiazole
