# Supplementary material for: Synthesis and Biological Evaluation of New Chalcogen Semicarbazone (S, Se) and Their Azole Derivatives against Chagas Disease
Source: J Med Chem. 2024 Nov 1;67(21):19038–56. doi: 10.1021/acs.jmedchem.4c01535 (PMC12320831; doi:10.1021/acs.jmedchem.4c01535)

SUPPLEMENTARY MATERIAL

Synthesis and biological evaluation of new chalcogen semicarbazone (*S*, *Se*) and their azole derivatives against Chagas Disease.

Mercedes Rubio-Hernández[1], Verónica Alcolea[1], Elany Barbosa da Silva[2], Miriam A. Giardini[2], Thaís H.M. Fernandes[2], Nuria Martínez-Sáez[3], Anthony J. O'Donoghue[2], Jair L. Siqueira-Neto[2]*, Silvia Pérez-Silanes[1]*.

[1] ISTUN Institute of Tropical Health, Department of Pharmaceutical Sciences, Universidad de Navarra, 31008 Pamplona, Spain.

[2] Skaggs School of Pharmacy and Pharmaceutical Sciences and Center for Discovery and Innovation in Parasitic Diseases, University of California, San Diego, La Jolla, 9500 Gilman Drive, California 92093, United States.

[3] Department of Pharmaceutical Sciences, Universidad de Navarra, 31008 Pamplona, Spain.

*sperez@unav.es and jairlage@health.ucsd.edu

Contents:

1. Biological data
    a. Table S1.. S2
    b. DRC against *T. cruzi*.. S9
2. Molecular Dynamic data S10
3. Radical scavenging capacity S11
4. IR, ^{1}H-NMR, ^{13}C-NMR, ^{77}Se-NMR and qNMR spectra of final compounds
    a. *S*1 series S12
    b. *Se*1 series S46
    c. *S*2 series S76
    d. *Se*2 series S106
5. Bidimensional spectra of compounds ***S*1k** and ***Se*1k** S132

1. Biological data

a. Table S1

Table S1. Results from the 57 compounds screened against the trypomastigote and amastigote form of *T. cruzi* CAI/72 strain, Cz and *h*CatL.

		Cell-based assays				Enzymatic assays				
**Structure**	**Compound**	**Anti-*Tc* CAI/72 activity (%)^**[a]	**C2C12 viability (%)^**[b]	**EC_{50} *Tc* CAI/72 (μM)**[c]	**SI_{Tc}**[d]	**Cz inhibition (%)^**[e]	**IC_{50} Cz (nM)**[f]	***h*CatL inhibition (%)^**[g]	**IC_{50} *h*CatL (nM)**[h]	**SI_{Cz}**[i]
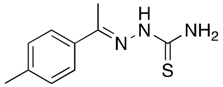	***S*1a**	42.28	>100	NT		74.84				
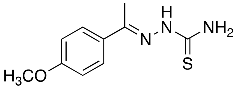	***S*1b**	23.19	>100	NT		87.12				
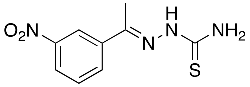	***S*1c**	20.61	>100	NT		97.59				
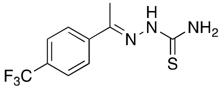	***S*1d**	43.82	93.11	NT		85.81				
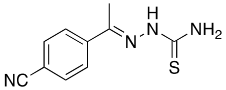	***S*1e**	31.58	>100	NT		98.77				
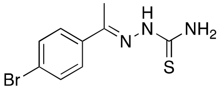	***S*1f**	45.44	>100	NT		97.07				
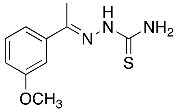	***S*1g**	22.29	>100	NT		95.31				
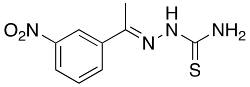	***S*1h**	24.80	>100	NT		100.00	1.15 ± 0.74	94.51	189.55 ± 18.35	164.83

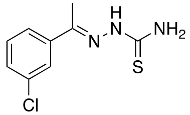	***S*1i**	**56.20**	**>100**	**~ 10.0 ± n/a**	**~ 1.00**	**100.00**	**2.50 ± 0.51**	**97.25**	**89.41 ± 31.39**	**35.76**
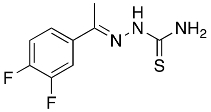	***S*1j**	49.89	>100	NT		99.79				
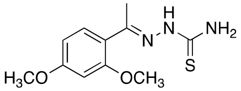	***S*1k**	0.70	>100	NT		39.26				
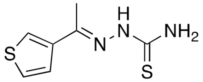	***S*1l**	10.89	>100	NT		62.83				
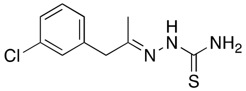	***S*1m**	**69.39**	**>100**	**2.92 ± 0.156**	**> 3.42**	**72.30**				
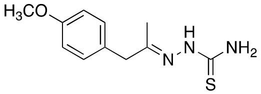	***S*1n**	34.47	>100	NT		56.20				
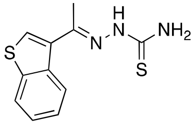	***S*1o**	23.14	>100	NT		100.00				
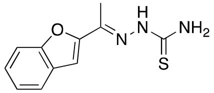	***S*1p**	49.72	>100	NT		99.30				
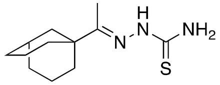	***S*1q**	38.73	>100	NT		5.06				
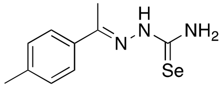	***Se*1a**	21,64	34.06	NT		98.25				

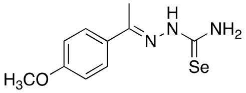	***Se*1b**	29.98	40.87	NT		98.92				
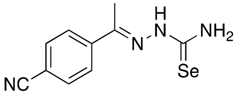	***Se*1e**	**57.10**	**>100**	NT		**104.45**				
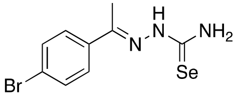	***Se*1f**	**69.53**	**>100**	**0.77 ± 0.17**	**> 13.00**	**101.08**	**14.31 ± 3.57**	**97.30**	**106.14 ± 14.86**	**7.42**
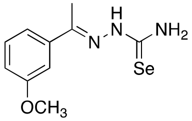	***Se*1g**	7.03	31.06	NT		101.39				
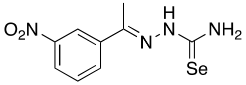	***Se*1h**	40.95	97.30	NT		104.46	0.91 ± 0.18	98.65	9.32 ± 0.38	10.22
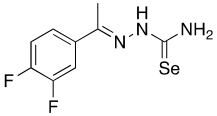	***Se*1j**	NA	27.70	NT		101.82				
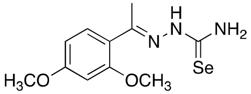	***Se*1k**	NA	43.74	NT		92.47				
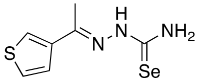	***Se*1l**	30.50	38.12	NT		90.07				
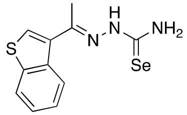	***Se*1o**	4.32	31.44	NT		93.73				
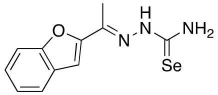	***Se*1p**	28.77	39.40	NT		102.35				

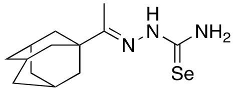	***Se*1q**	16.20	78.62	NT		83.80				
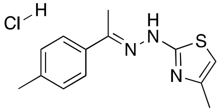	***S2*a**	12.53	>100	NT		36.82				
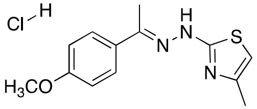	***S2*b**	23.54	>100	NT		37.51				
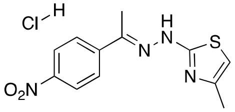	***S2*c**	NA	99.21	NT		96.73				
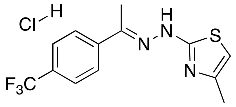	***S2*d**	NA	>100	NT		41.62				
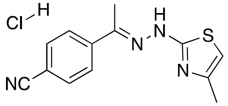	***S2*e**	22.41	>100	NT		89.85				
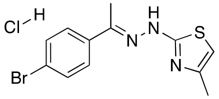	***S2*f**	1.10	>100	NT		53.03				
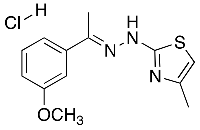	***S2*g**	40.57	>100	NT		45.14				
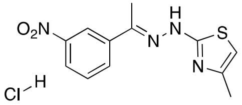	***S2*h**	28.80	>100	NT		96.89	1284.50 ± 1115.50	12.12	NT; IC50 > 10µM	

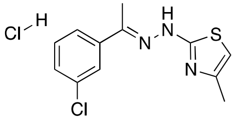	***S*2i**	49.31	>100	NT		97.55	142.17 ± 79.24	24.67	NT; IC50 > 10µM	
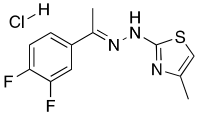	***S*2j**	28.30	>100	NT		79.38				
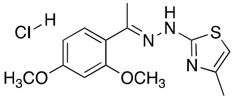	***S*2k**	45.97	>100	NT		34.18				
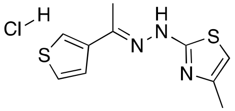	***S*2l**	19.54	>100	NT		31.57				
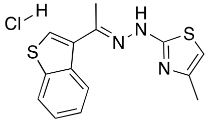	***S*2o**	45.73	>100	NT		27.85				
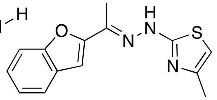	***S*2p**	31.35	>100	NT		74.29				
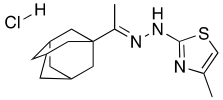	***S*2q**	40.47	>100	NT		30.49				
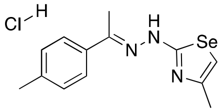	***Se*2a**	11.12	91.87	NT		28.64				
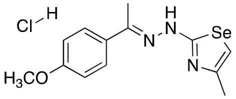	***Se*2b**	12.14	94.05	NT		21.62				

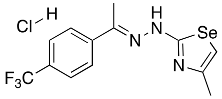	***Se*2d**	27.91	96.09	NT		95.20				
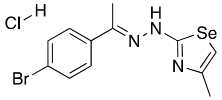	***Se*2f**	18.29	87.65	NT		98.63				
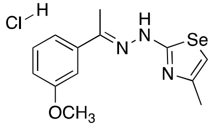	***Se*2g**	**55.07**	**>100**	**2.67 ± 1.13**	**> 3.75**	62.93				
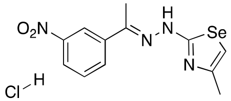	***Se*2h**	**57.70**	**>100**	**0.31 ± 0.18**	**> 32.47**	**100.09**	**8.99 ± 5.56**	**92.16**	**1338.0 ± 18.0**	**148.83**
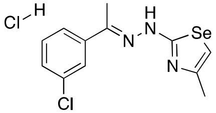	***Se*2i**	**65.03**	**>100**	**0.53 ± 0.57**	**> 18.98**	**102.55**	**81.69 ± 17.88**	**97.97**	**453.70 ± 4.40**	**5.55**
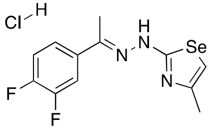	***Se*2j**	18.69	38.28	NT		102.31				
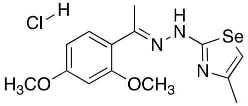	***Se*2k**	38.11	>100	NT		NA				
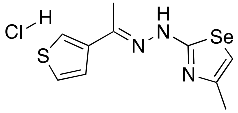	***Se*2l**	**50.37**	**>100**	**3.73 ± n/a**	**> 2.68**	**NA**				
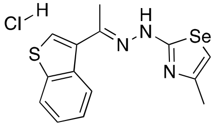	***Se*2o**	42.26	>100	NT		101.23				

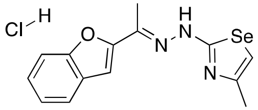	***Se*2p**	26.61	>100	NT		98.51				
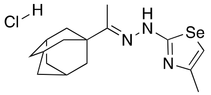	***Se*2q**	40.64	>100	NT		NA				
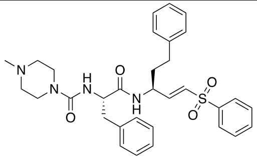	**K777**	60.98*	>100	0.014 ± 0.0030	> 218.98		1.56 ± 0.15*		0.20 ± 0.0064*	0.13
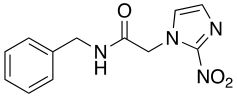	**BZ**	81.71*	>100	5.79 ± 1.20	> 6.91					

^ Assays performed at 10 µM

* Assays performed at 3 µM for **K777** and 40 µM for **BZ**

[a]Anti-*Tc* CAI/72-C2C12 is showed as percentage of anti *T.cruzi* CAI/72 activity, calculated at 10 µM in C2C12 infected cells. We established > 50% activity as threshold to move forward to other assays.

[b]C2C12 viability is calculated at 10 µM and showed as percentage. Compounds with < 50% viability are considered cytotoxic.

[c]EC_{50} (µM) is expressed as the average of three independent experiments in triplicates (n=9) ± standard deviation (SD) against intracellular amastigote parasites.

[d]SI_{Tc} shows the selectivity for the parasite and it is the ratio between EC_{50} C2C12 and EC_{50} *Tc* CAI/72. EC_{50} C2C12 is calculated as an estimation (>10 µM).

[e]Cz inhibition is showed as percentage. It is calculated at 10 µM. We established > 85% Cz inhibition as threshold to move forward to other assays.

[f]IC_{50} Cz (nM) is expressed as the mean of two different experiments, each of them performed in triplicates (n=6) ± standard error of the mean (SEM). Error is given by the ratio of the standard deviation to the square root of the number of measurements.

[g]*h*CatL inhibition is showed as percentage. It is calculated at 10 µM. We established > 90% *h*CatL inhibition as threshold.

[h]IC_{50} *h*CatL (nM) is expressed as the mean of two different experiments, each of them performed in triplicates (n=6) ± standard error of the mean (SEM). Error is given by the ratio of the standard deviation to the square root of the number of measurements.

[i]SI_{Cz} shows the selectivity for Cz and it is calculated as the ratio between IC_{50} *h*CatL and IC_{50} Cz.

NT: not tested. NA: not active. n/a: not applicable.

b. DRC against *T. cruzi*

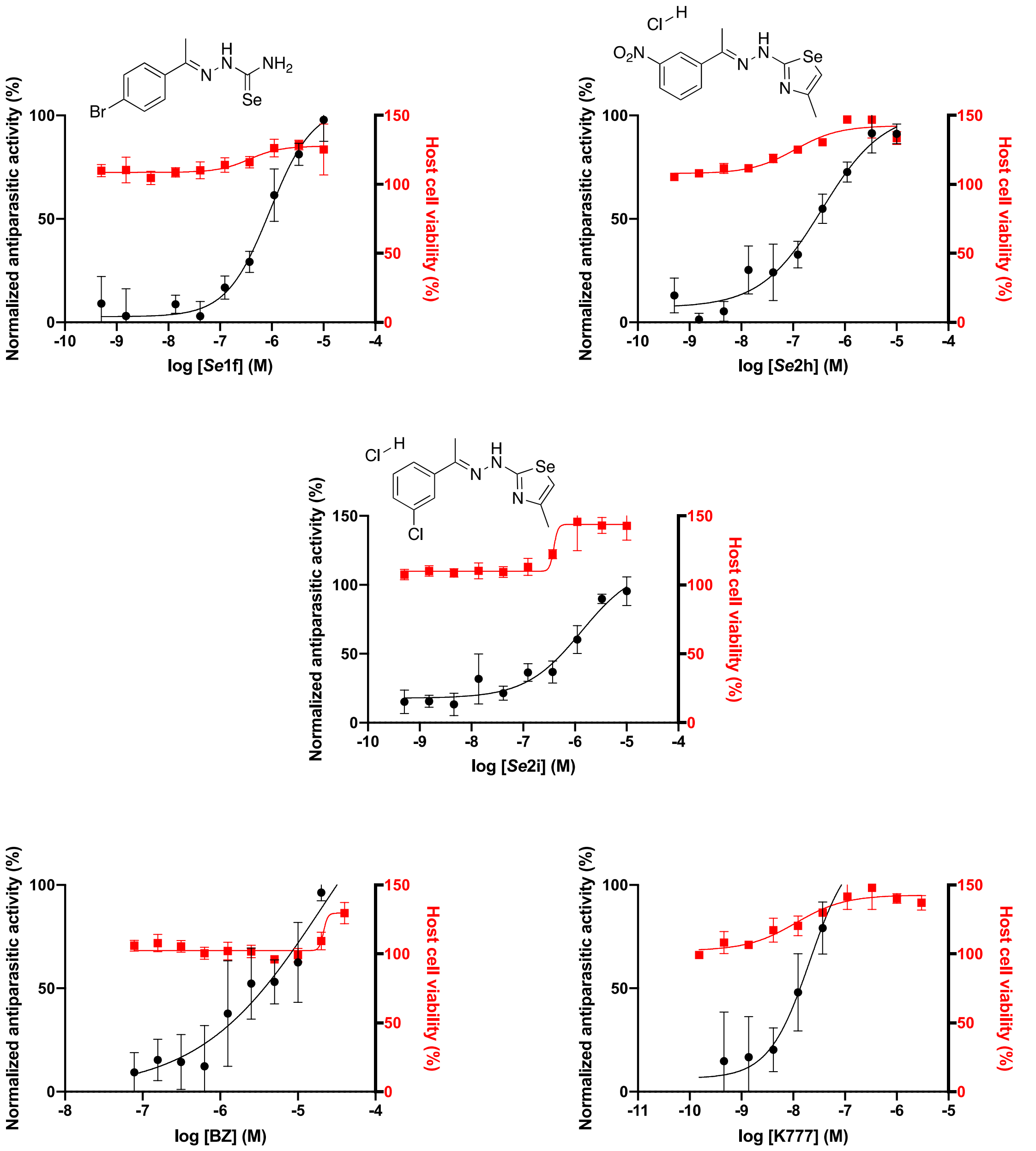

Figure S1. DRC of the three selected compounds with EC_{50} < 1 µM and SI > 10 (***Se*1f**, ***Se*2h**, ***Se*2i**), and the reference compounds (**BZ**, **K777**). Each graph shows the compiled curve of the 3 experiments performed in triplicate. Error bars are expressed as the standard deviation (SD). The final EC_{50} is expressed as the average of three independent experiments ± SD. Black curve: Normalized antiparasitic activity (% *T. cruzi* CAI/72 reduction). Red curve: Host cell viability (% C2C12 cells).

2. Molecular Dynamic Data

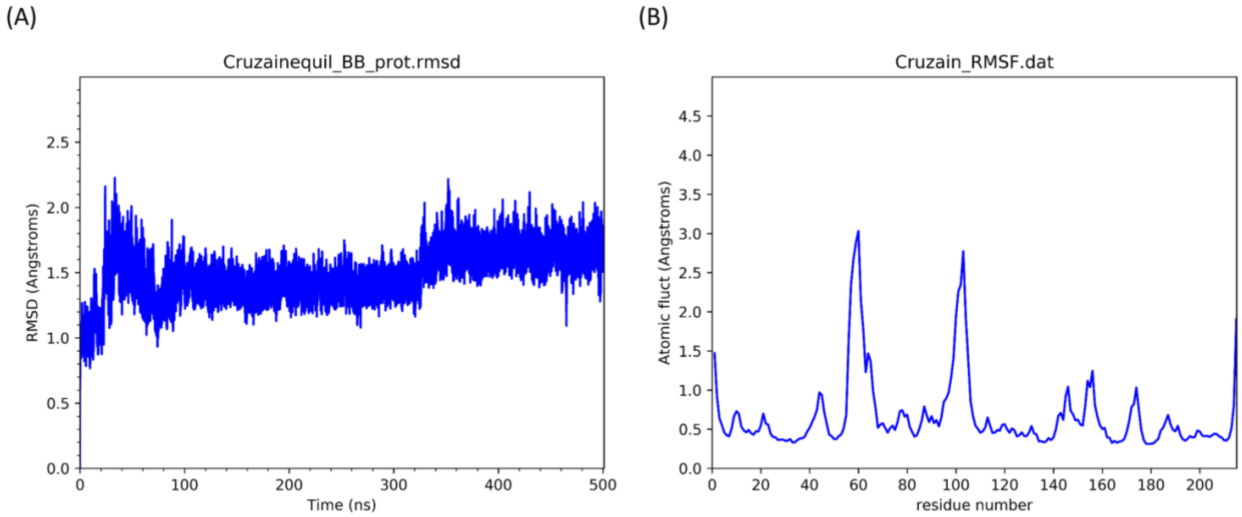

Figure S2. (A) Plot of protein *backbone* RMSD from simulations. (B) Protein residues RMSF plot.

Table S2. Average distances obtained for the main interactions and the trajectory percentage. They are present throughout the 500 ns of the MD simulation.

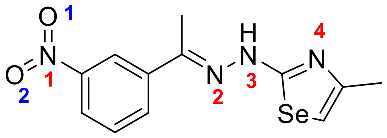

Se2h

Type of interaction	Involved Atoms	Distance (Å)	Occurrence (%)
H bond	Leu160 (O)- Ligand N3	2.8	47.9
H bond	Leu160 (N)- Ligand N4	2.9	17.5
H bond	Gly66 (N)- Ligand (O1)	2.9	2.56
H bond	Gly66 (N)- Ligand (O2)	2.9	2.23
CH-π	Leu160 (Cβ)-Ligand (Ph)	3.9	1.8
CH-π	Asn161 (Cα)-Ligand (Ph)	3.8	7.23
CH-π	Leu67 (Cσ)-Ligand (Ph)	3.8	15.1
Π-Hole	Gly66 (O)-Ligand N1	3.4	69.5

3. Radical scavenging capacity

Table S3. Radical scavenging capacity

Percentage of inhibited DPPH (2 h)			
	**0,06 mg/ml**	**0,03 mg/ml**	**0,015 mg/ml**
***Se*2h**	78,33 ± 1,76	78,00 ± 1,73	77,33 ± 0,88
**ASC**	80,33 ± 0,67	80,33 ± 0,33	65,33 ± 2,03
**TRO**	79,67 ± 0,67	79,33 ± 0,88	79,33 ± 1,33

Percentage of inhibited DPPH after 2 hours is expressed as the mean (%) ± SEM, at the three different concentrations tested. Ascorbic acid (ASC) and trolox (TRO) are used as positive controls.

4. IR, ^{1}H-NMR, ^{13}C-NMR, ^{77}Se-NMR and qNMR spectra of final compounds

a. *S*1 series

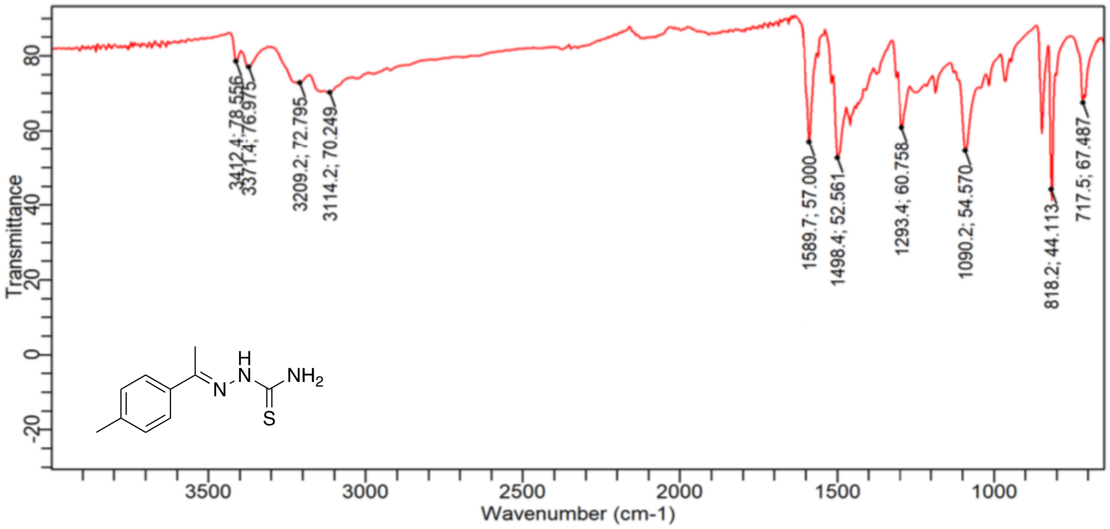

Figure S3. IR (up) and ^{1}H-NMR (down) of compound ***S*1a**.

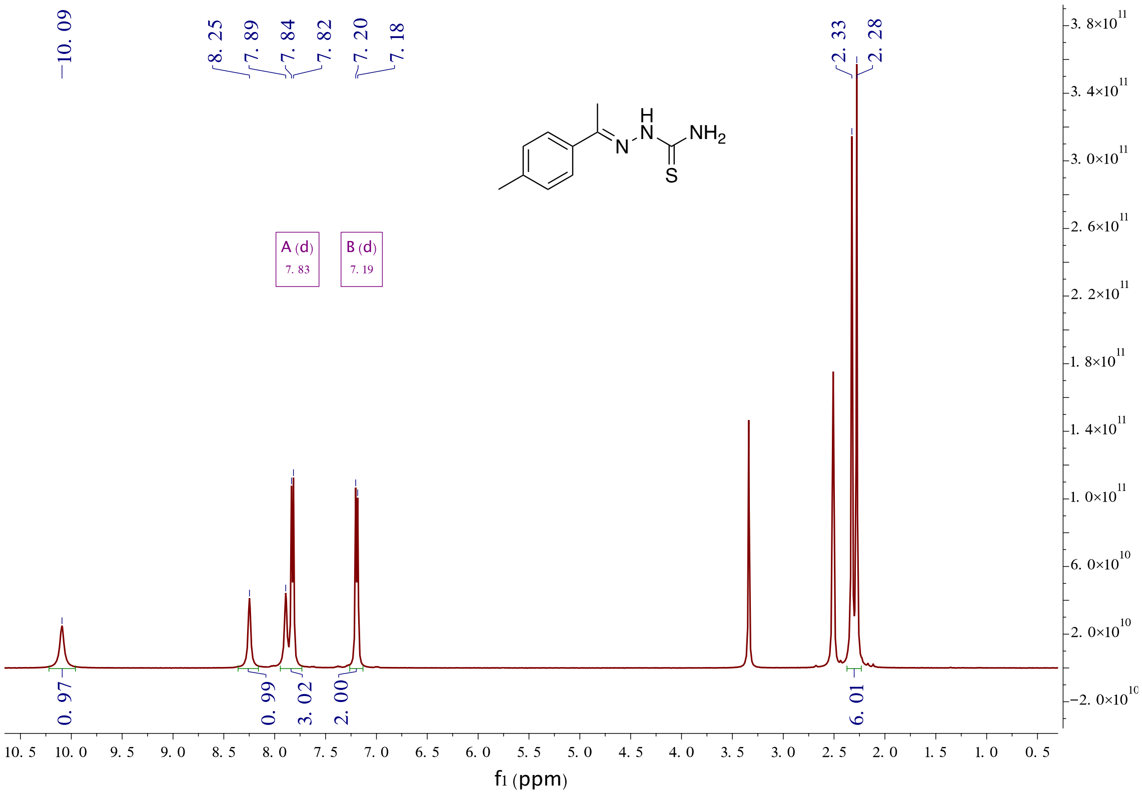

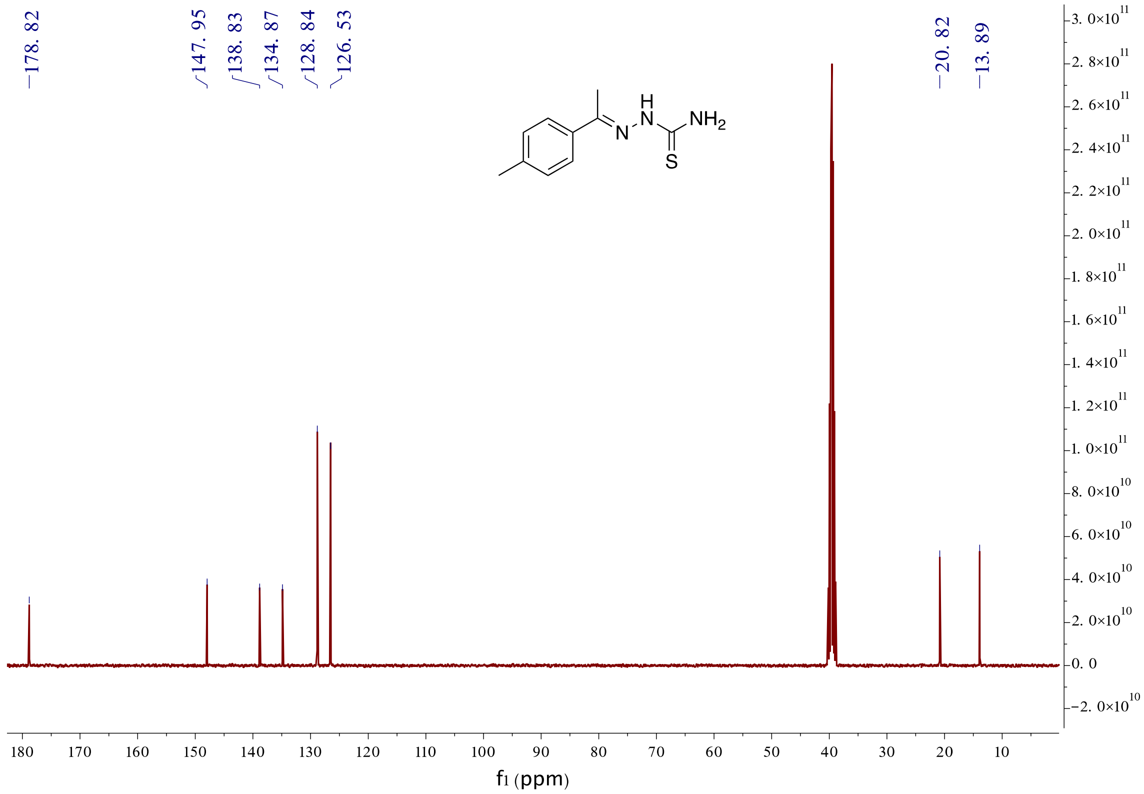

Figure S4. ^{13}C-NMR (up) and qNMR (down) of compound ***S*1a**.

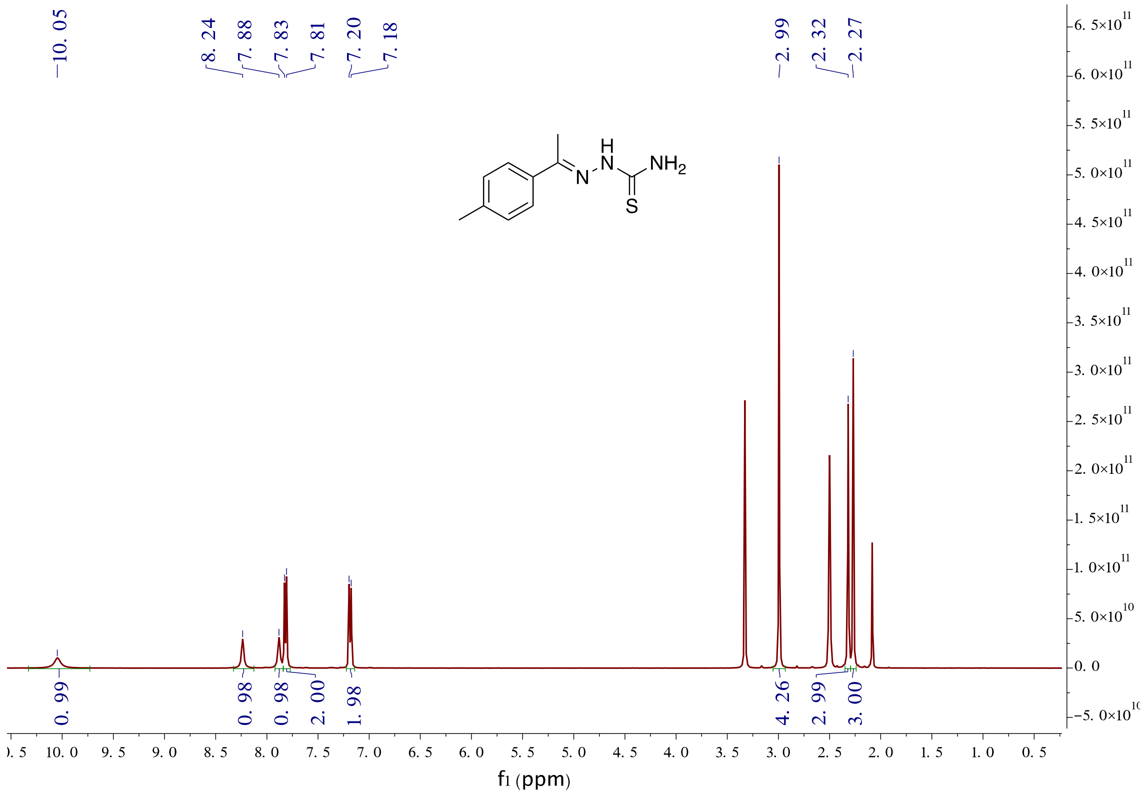

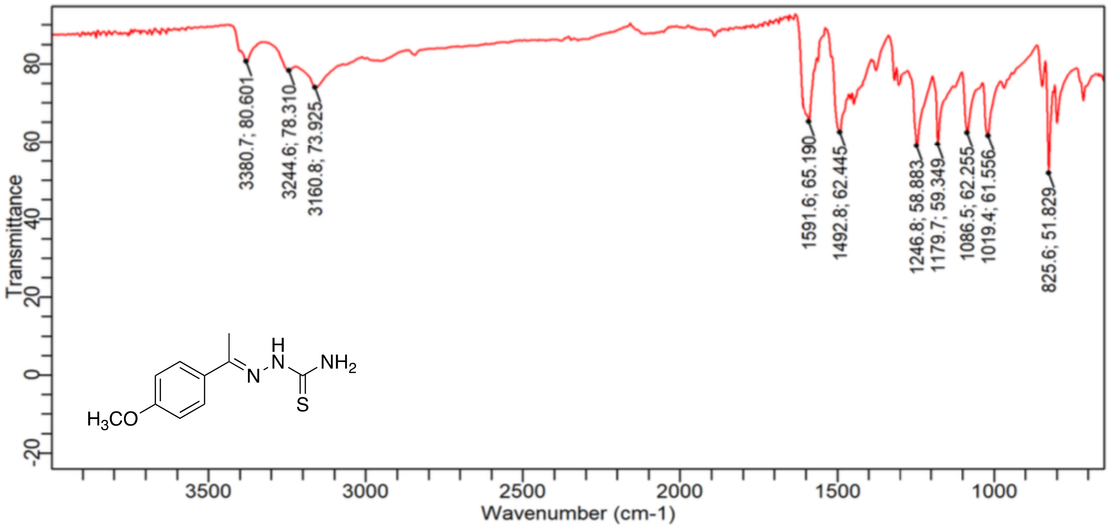

Figure S5. IR (up) and ^{1}H-NMR (down) of compound ***S*1b**.

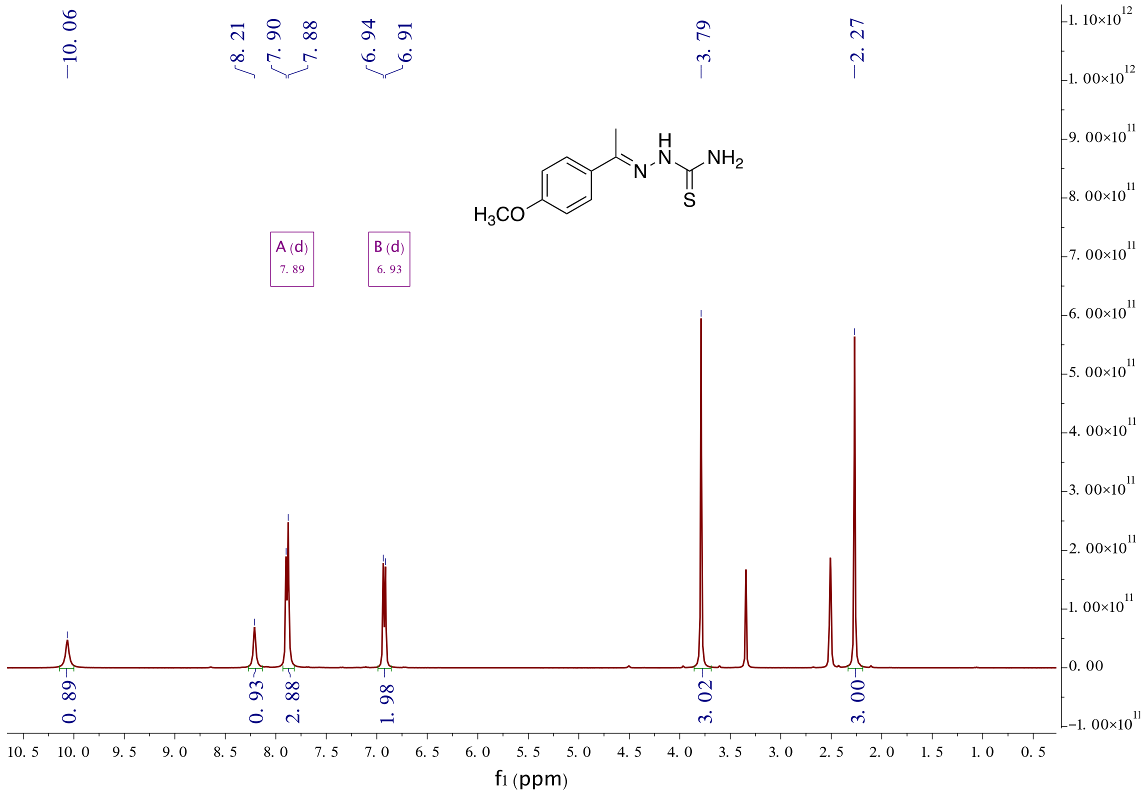

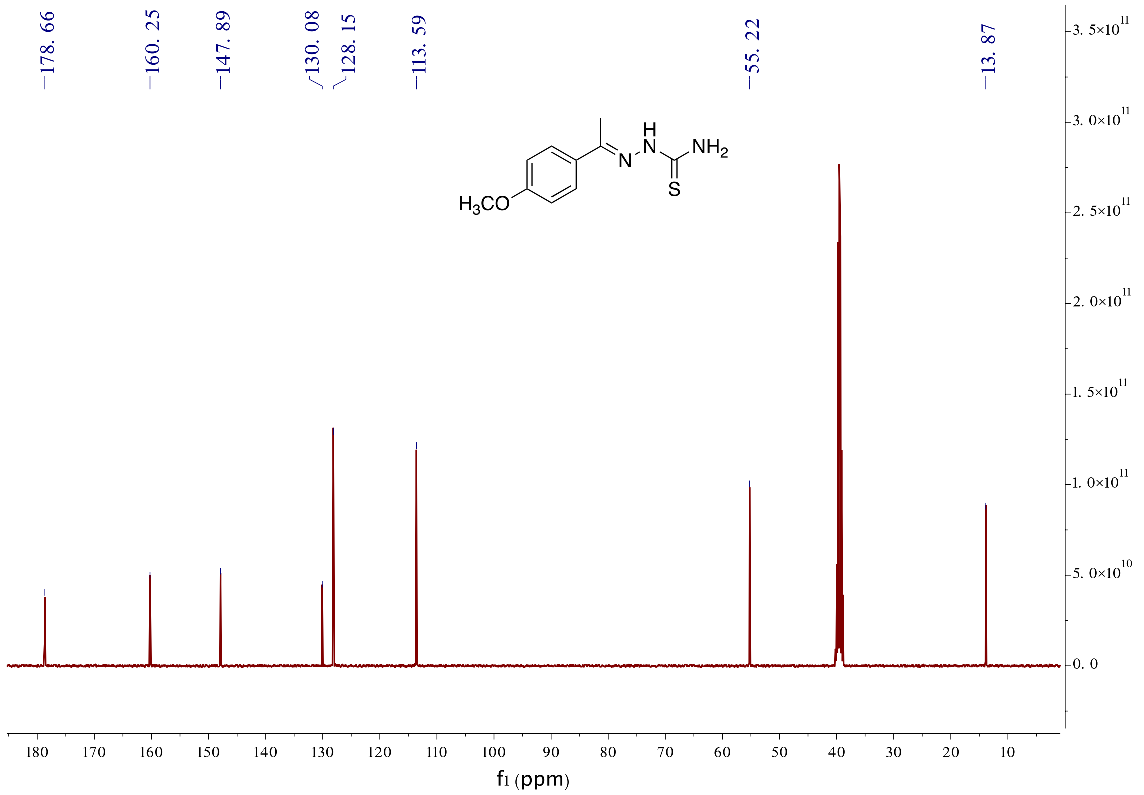

Figure S6. ^{13}C-NMR (up) and qNMR (down) of compound ***S*1b**.

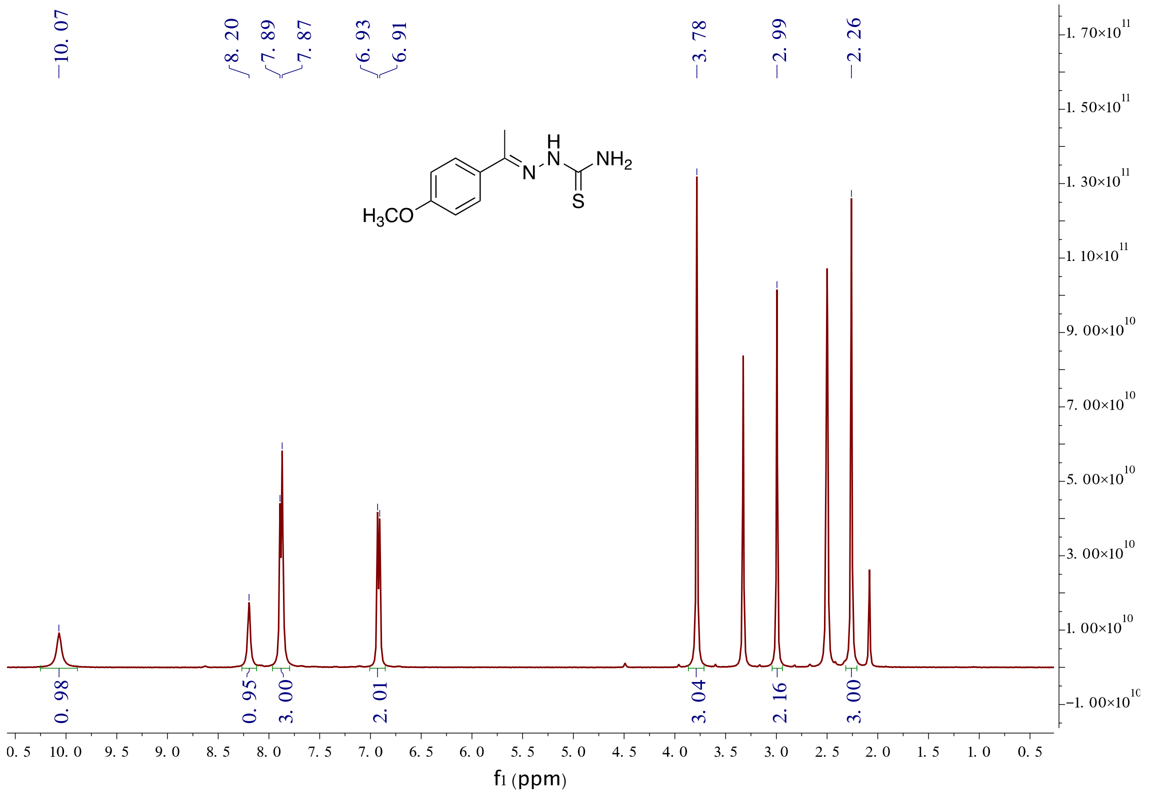

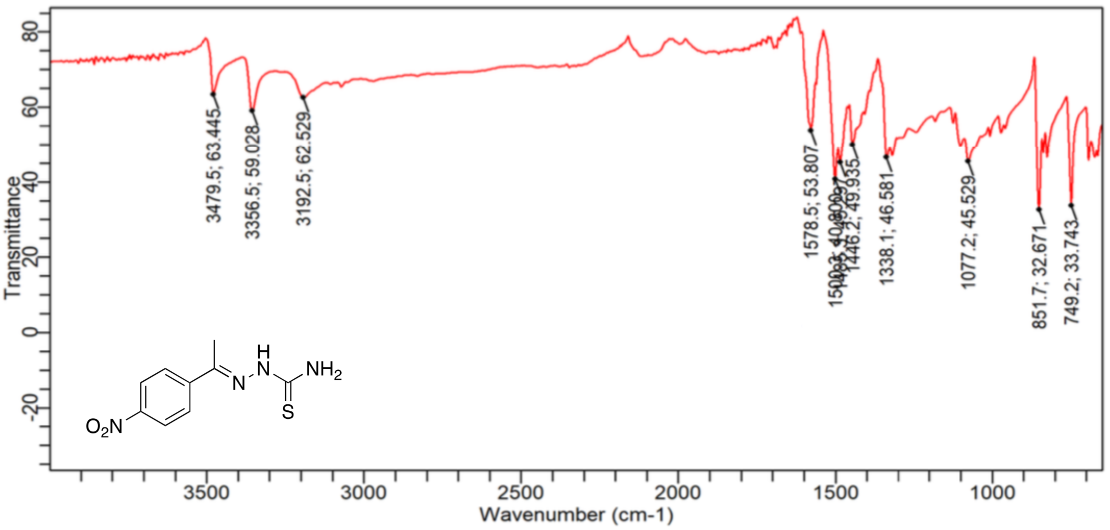

Figure S7. IR (up) and ^{1}H-NMR (down) of compound ***S*1c**.

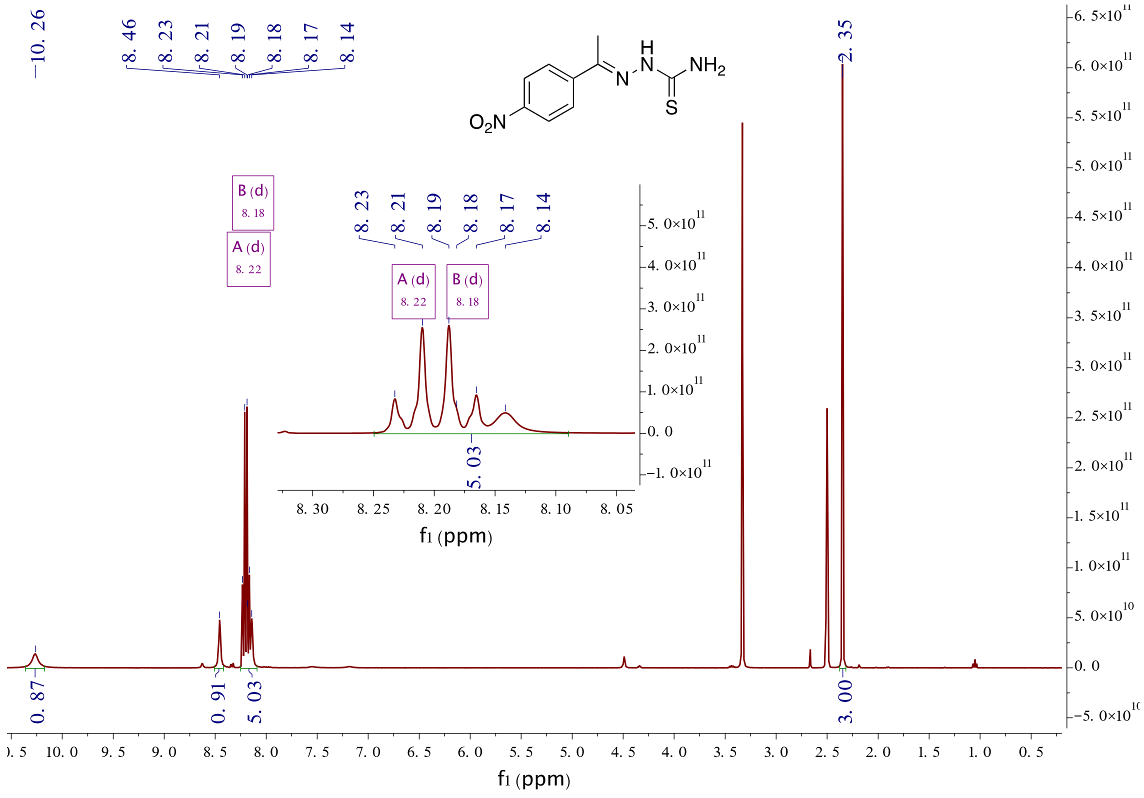

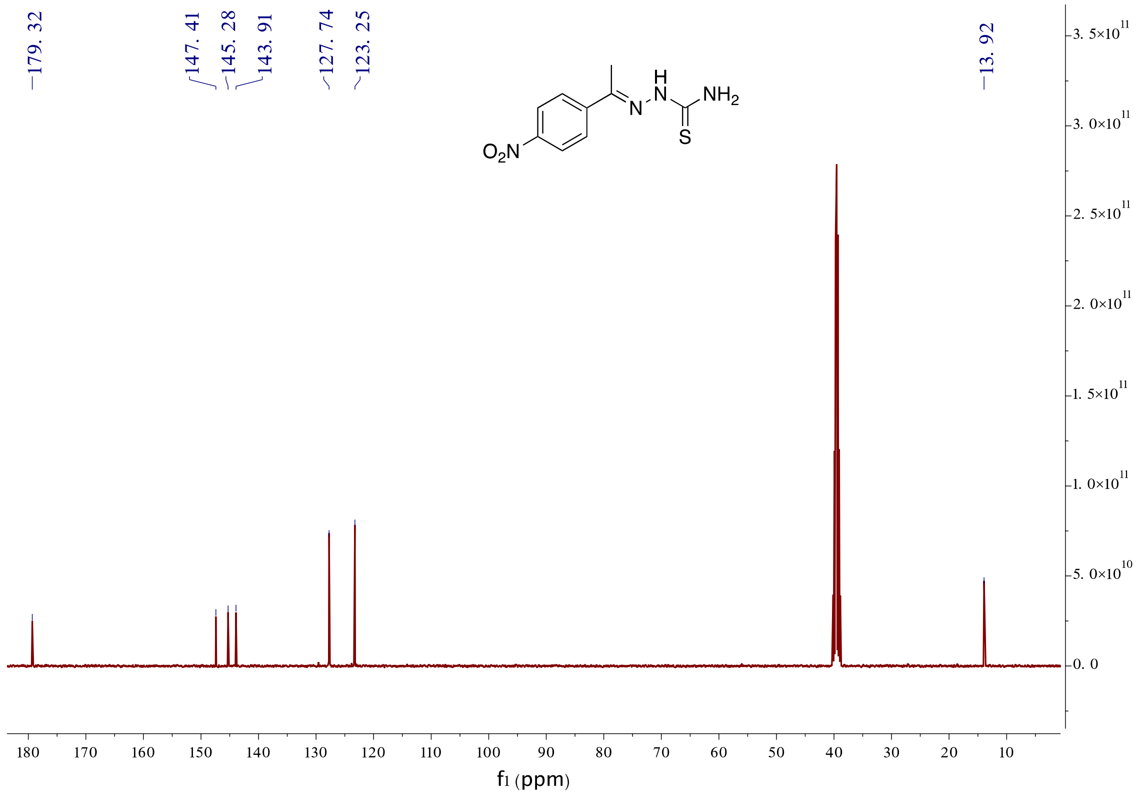

Figure S8. ^{13}C-NMR (up) and qNMR (down) of compound ***S*1c**.

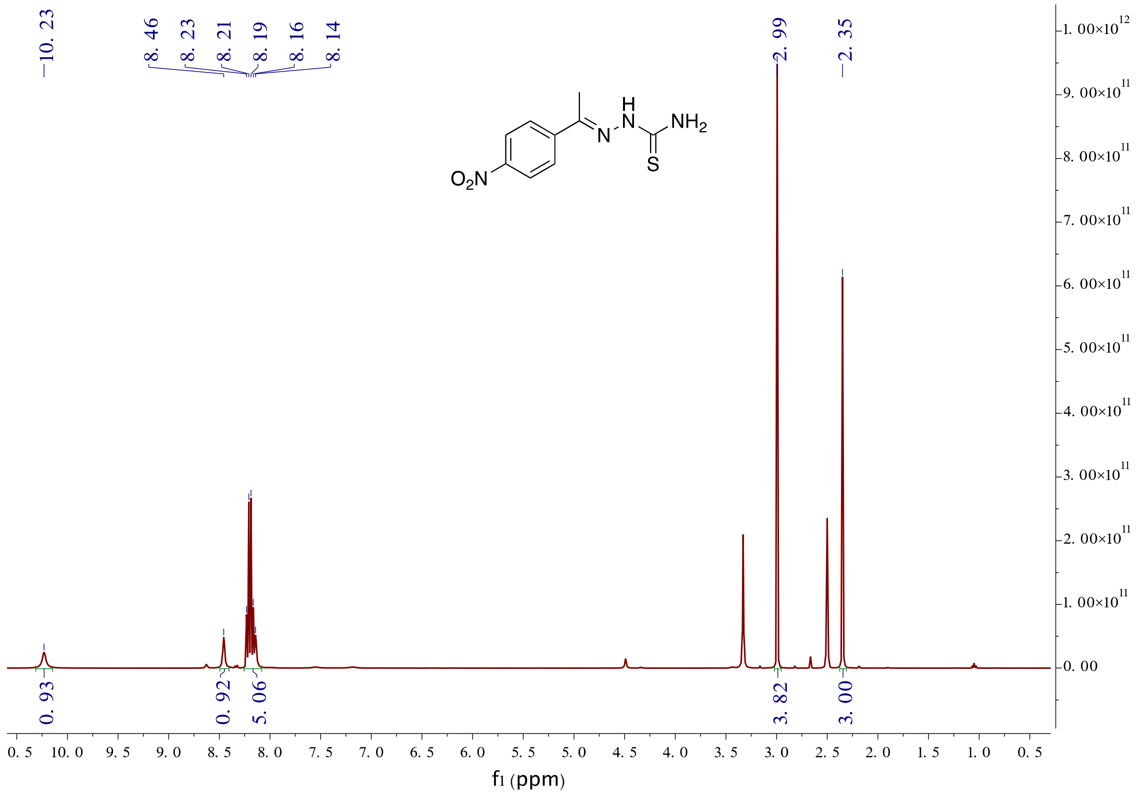

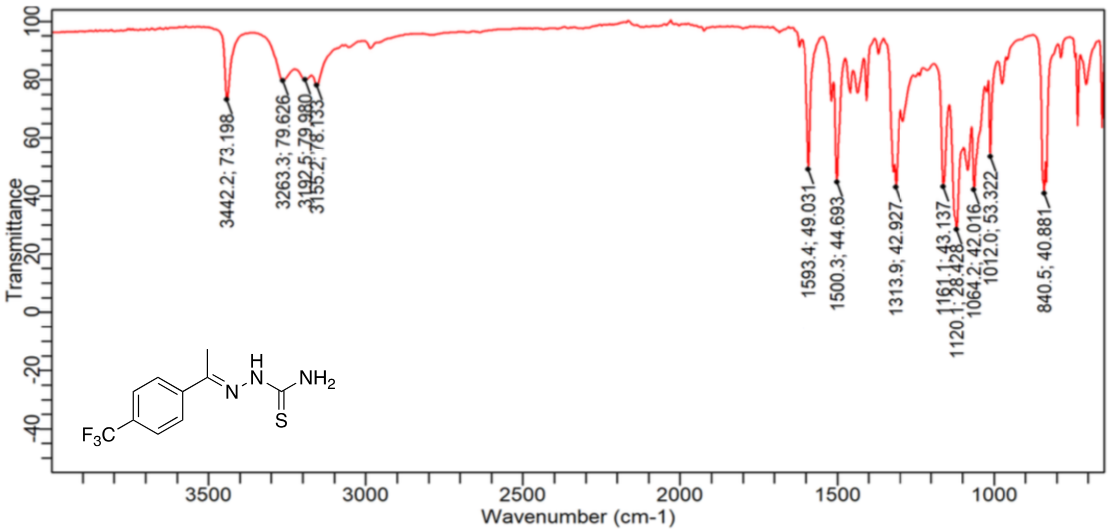

Figure S9. IR (up) and ^{1}H-NMR (down) of compound ***S*1d**.

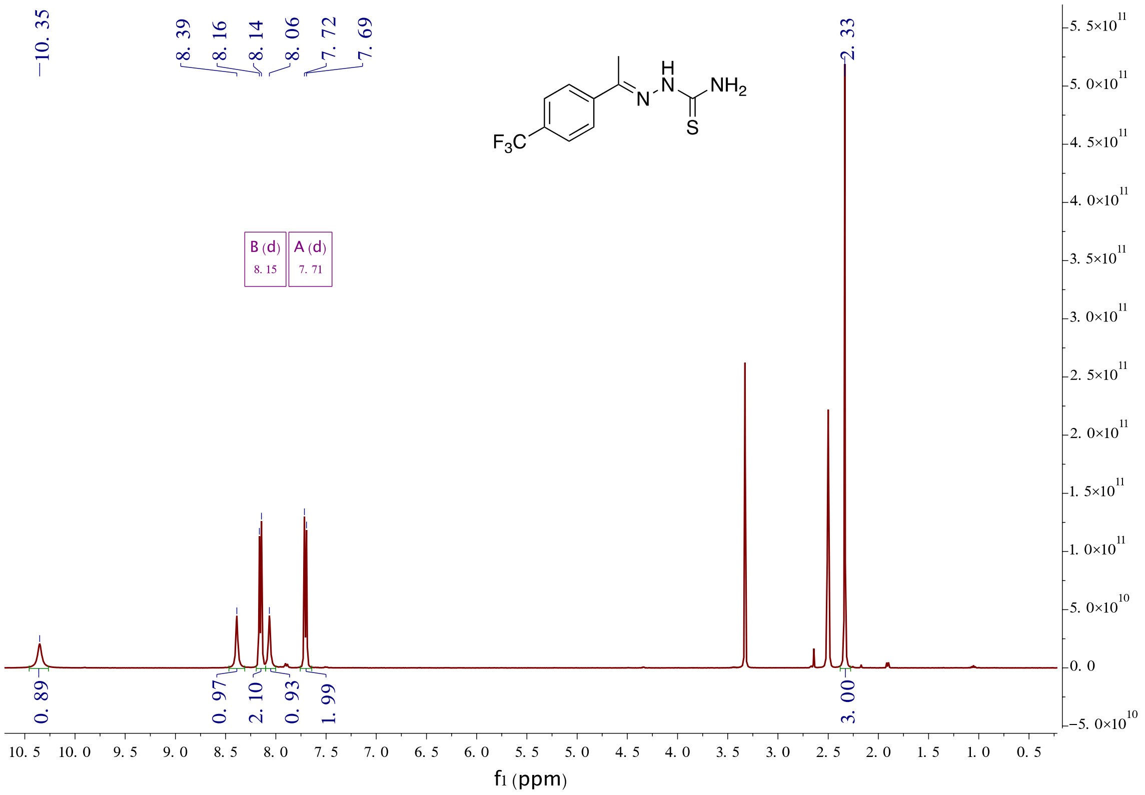

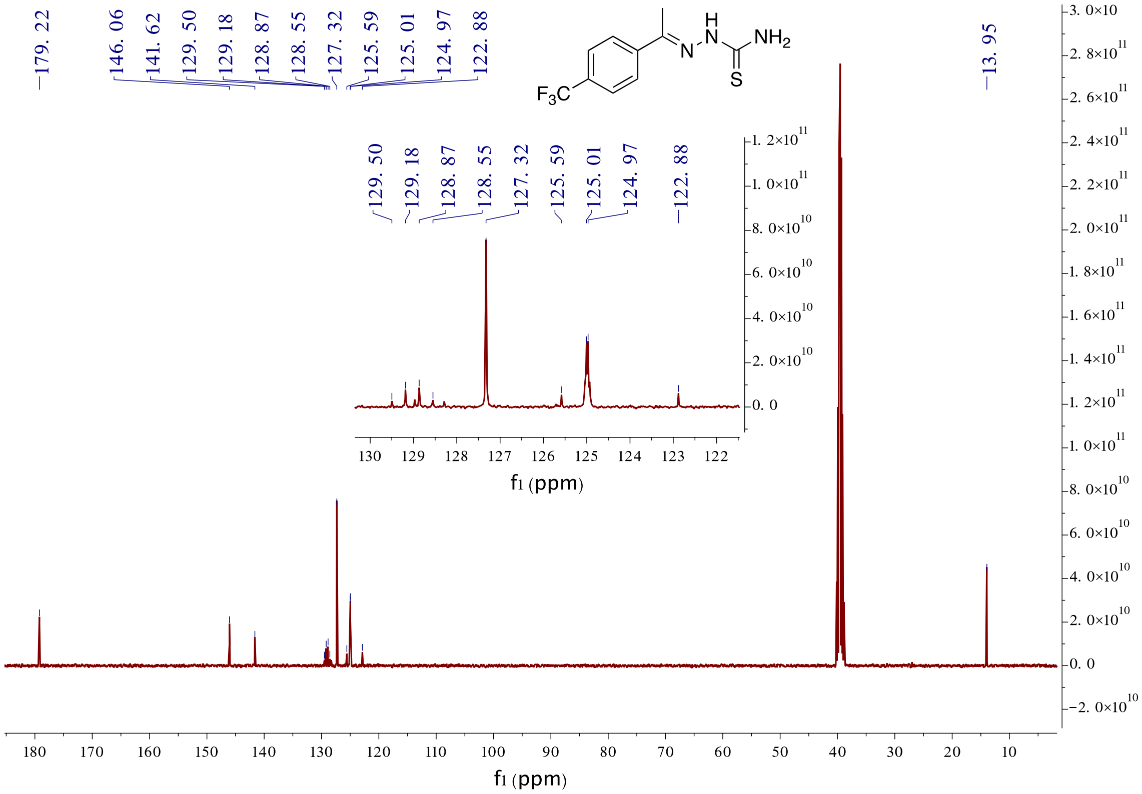

Figure S10. ^{13}C-NMR (up) and qNMR (down) of compound ***S*1d**.

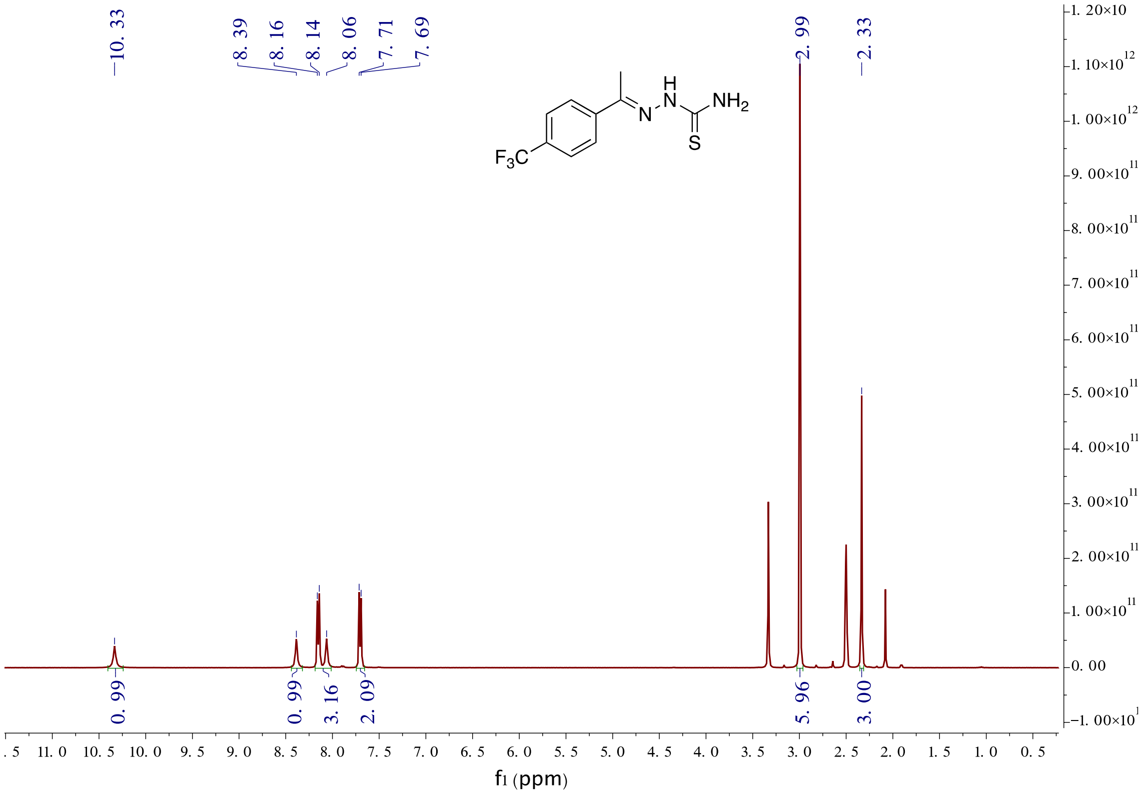

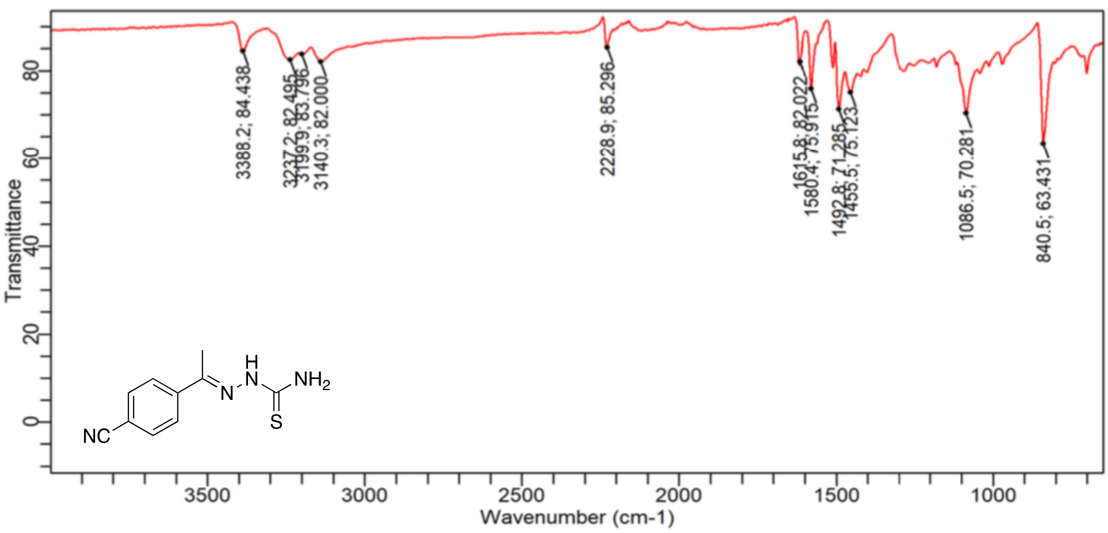

Figure S11. IR (up) and ¹H-NMR (down) of compound ***S*1e**.

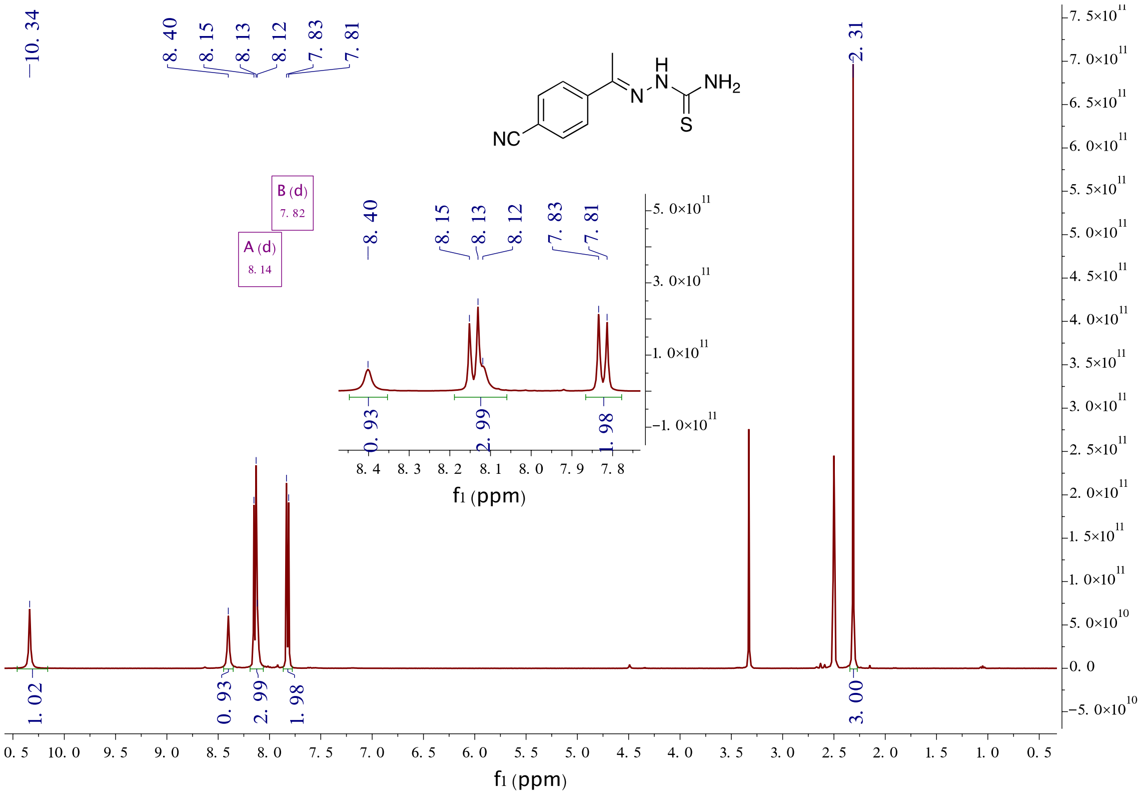

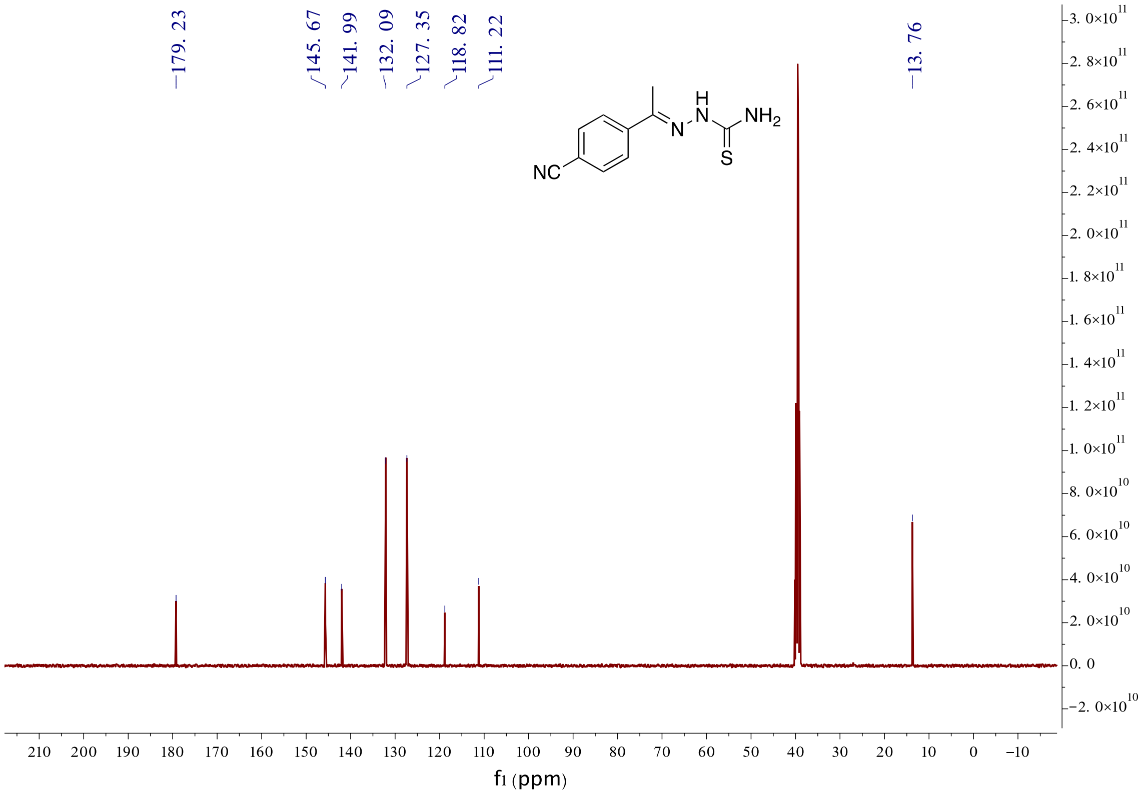

Figure S12. ^{13}C-NMR (up) and qNMR (down) of compound ***S*1e**.

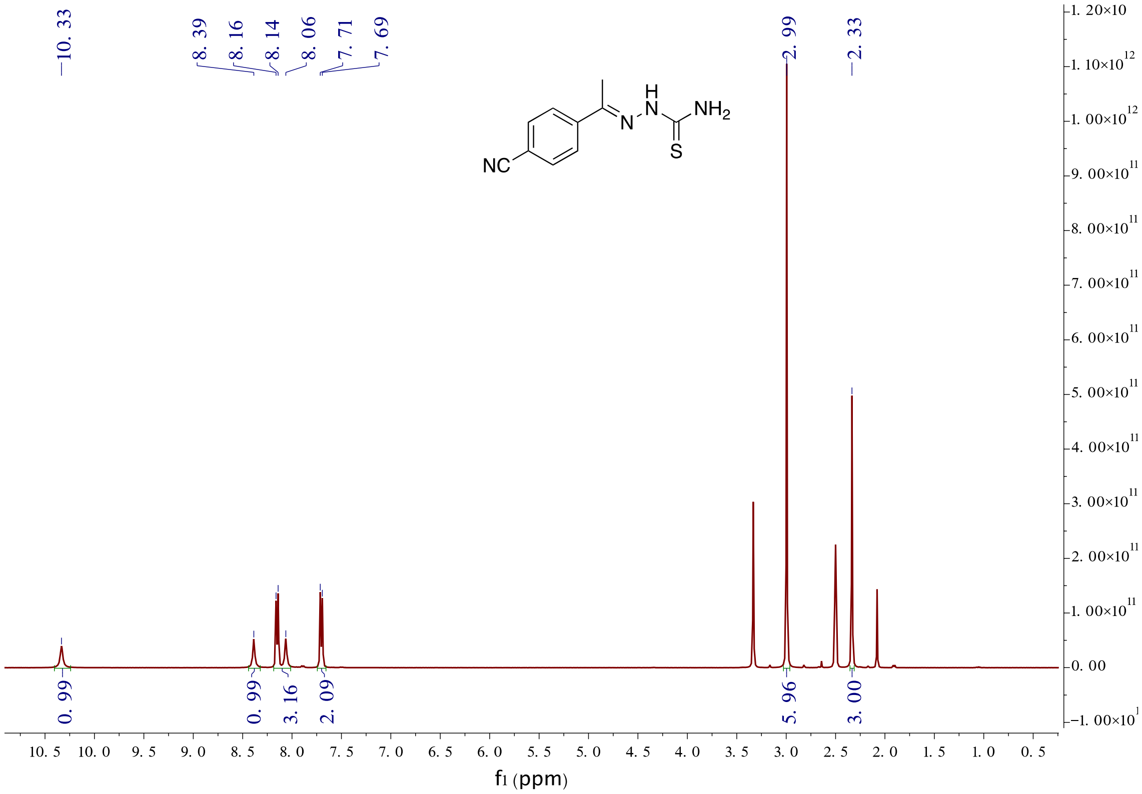

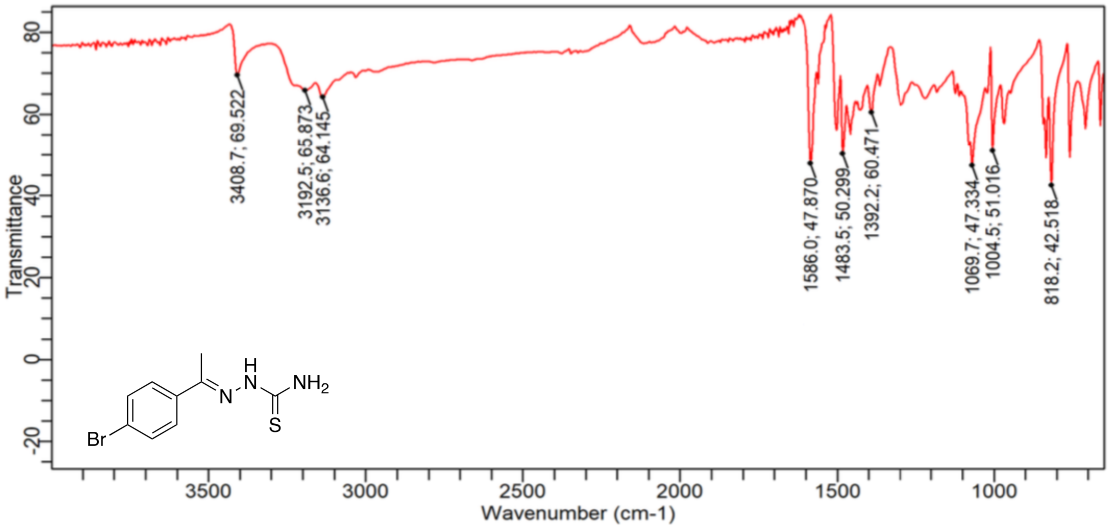

Figure S13. IR (up) and ^{1}H-NMR (down) of compound ***S*1f**.

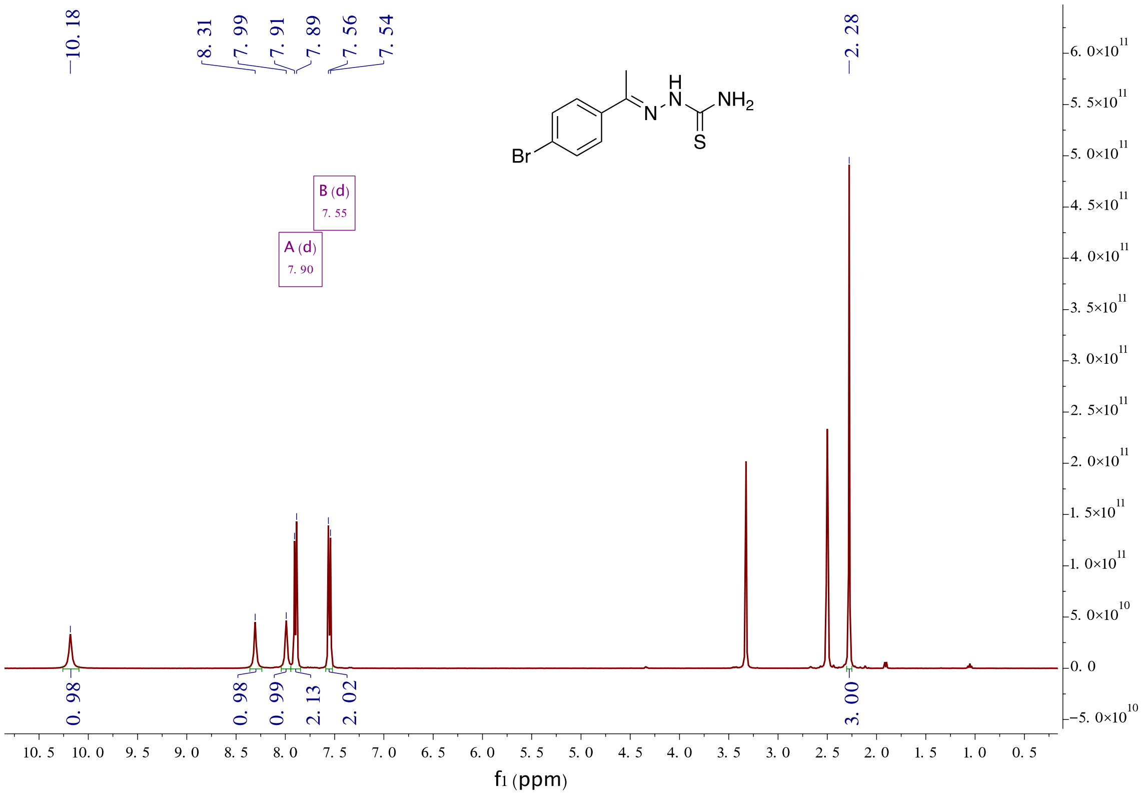

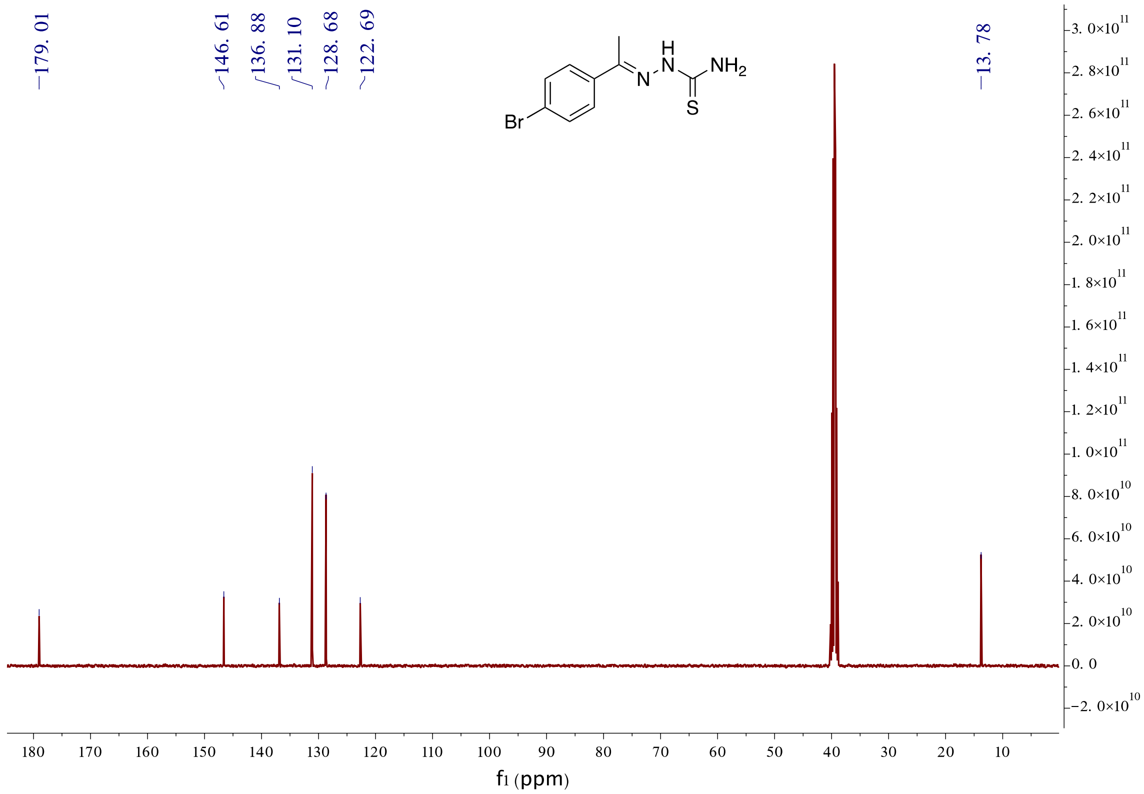

Figure S14. ^{13}C-NMR (up) and qNMR (down) of compound ***S*1f**.

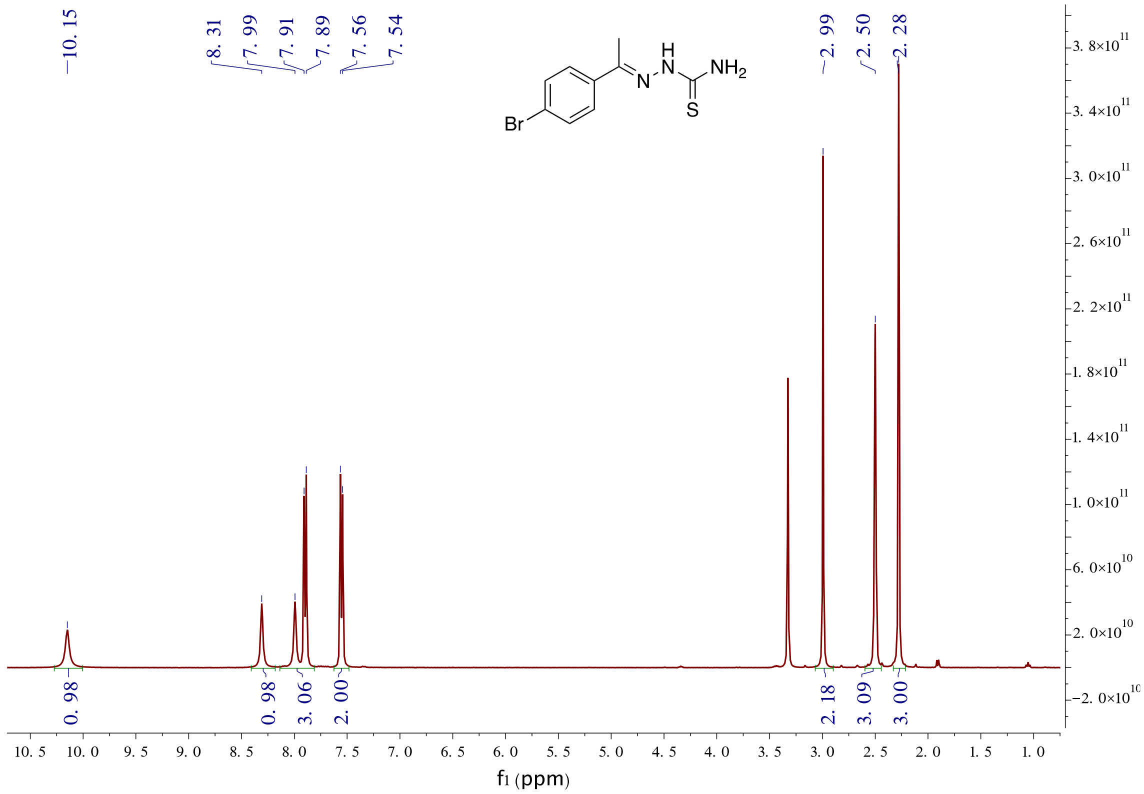

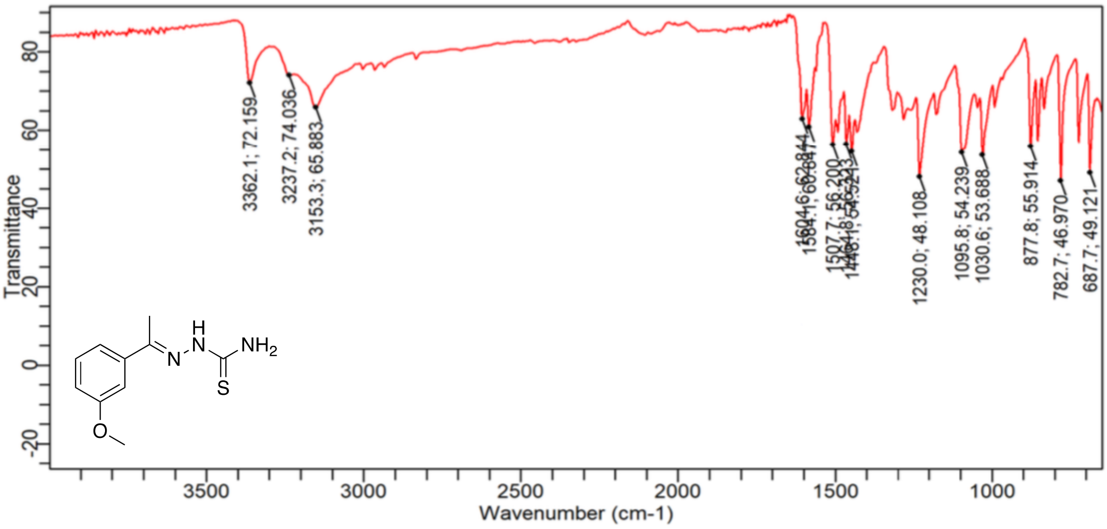

Figure S15. IR (up) and ^{1}H-NMR (down) of compound ***S*1g**.

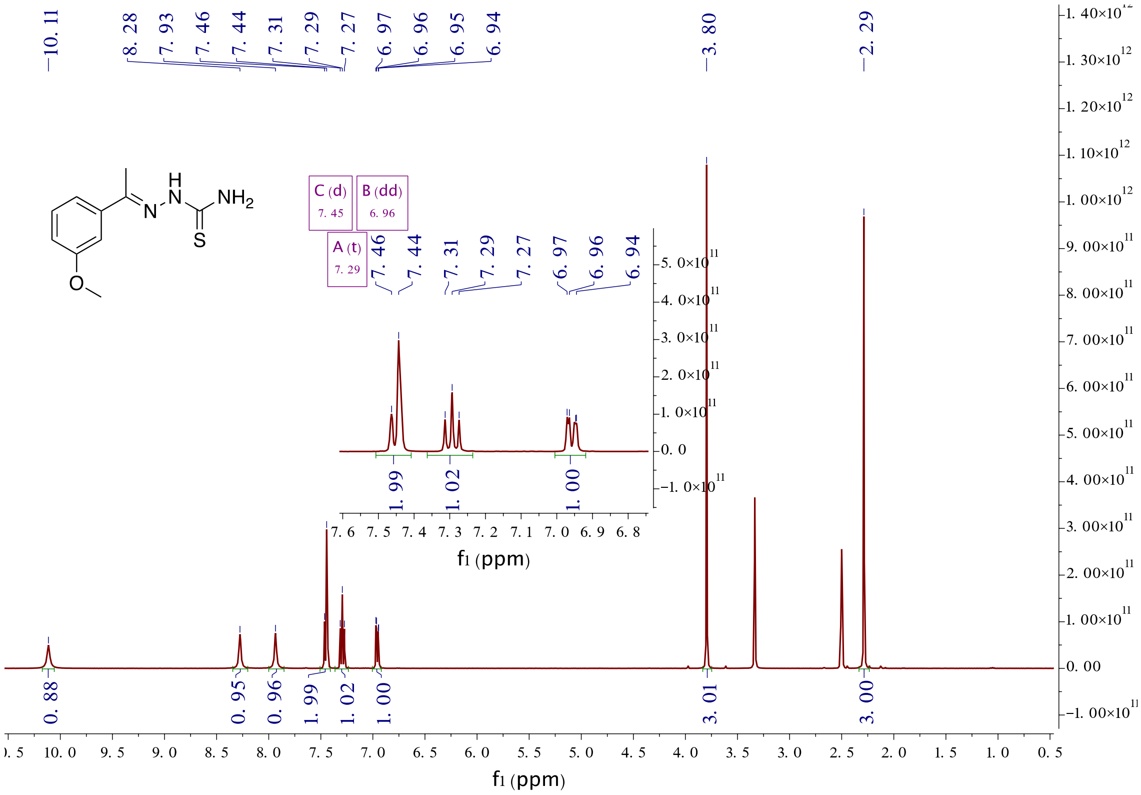

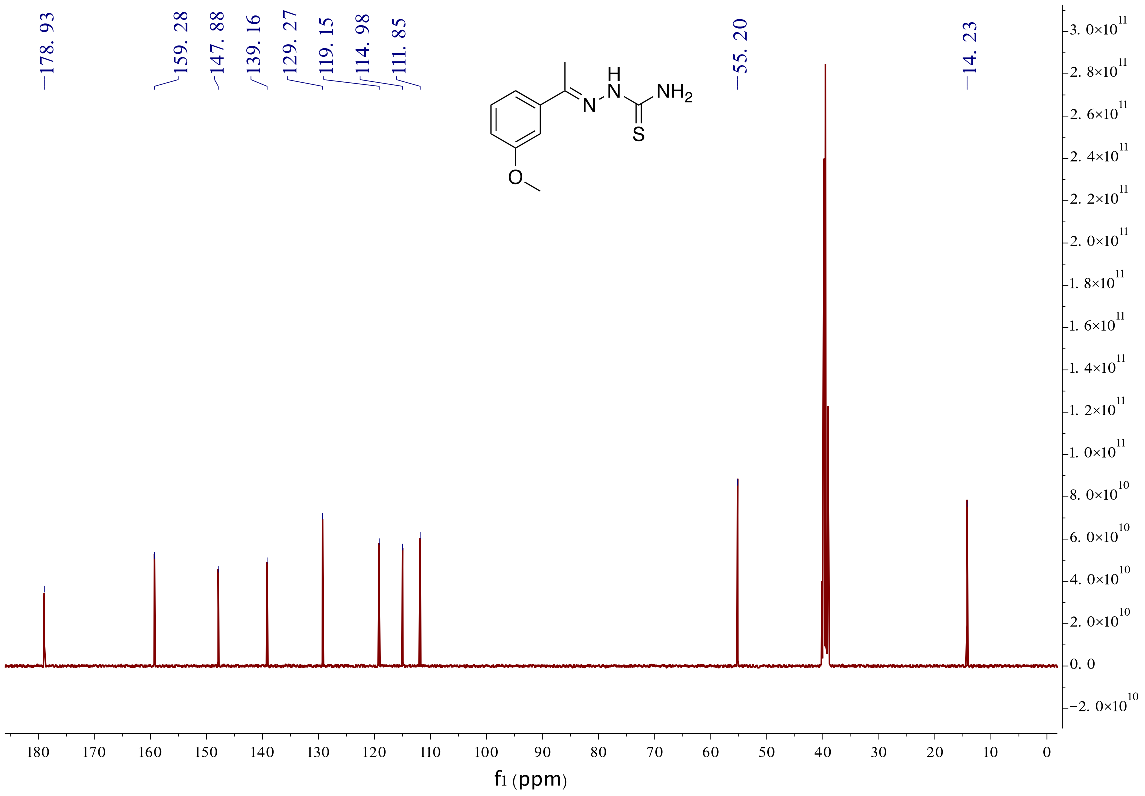

Figure S16. ^{13}C-NMR (up) and qNMR (down) of compound ***S*1g**.

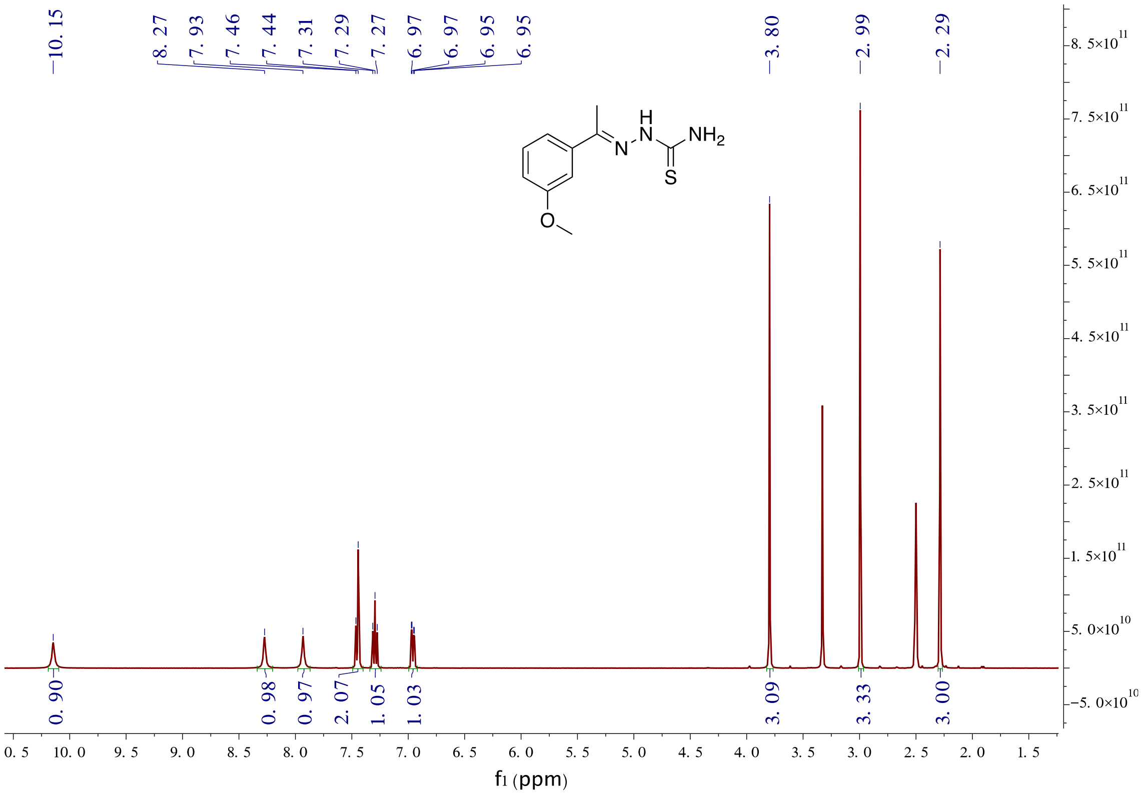

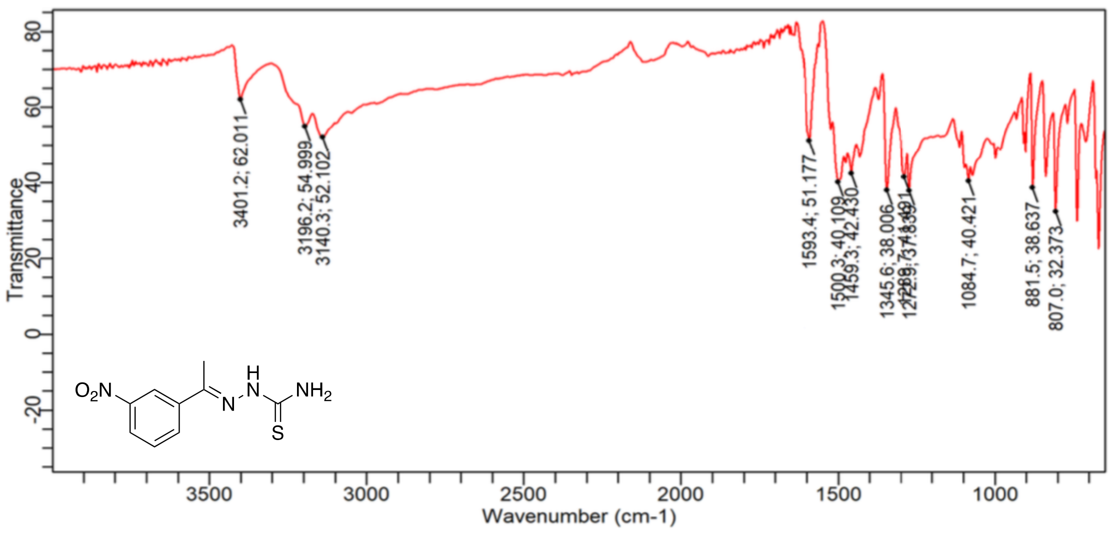

Figure S17. IR (up) and ^{1}H-NMR (down) of compound ***S*1h**.

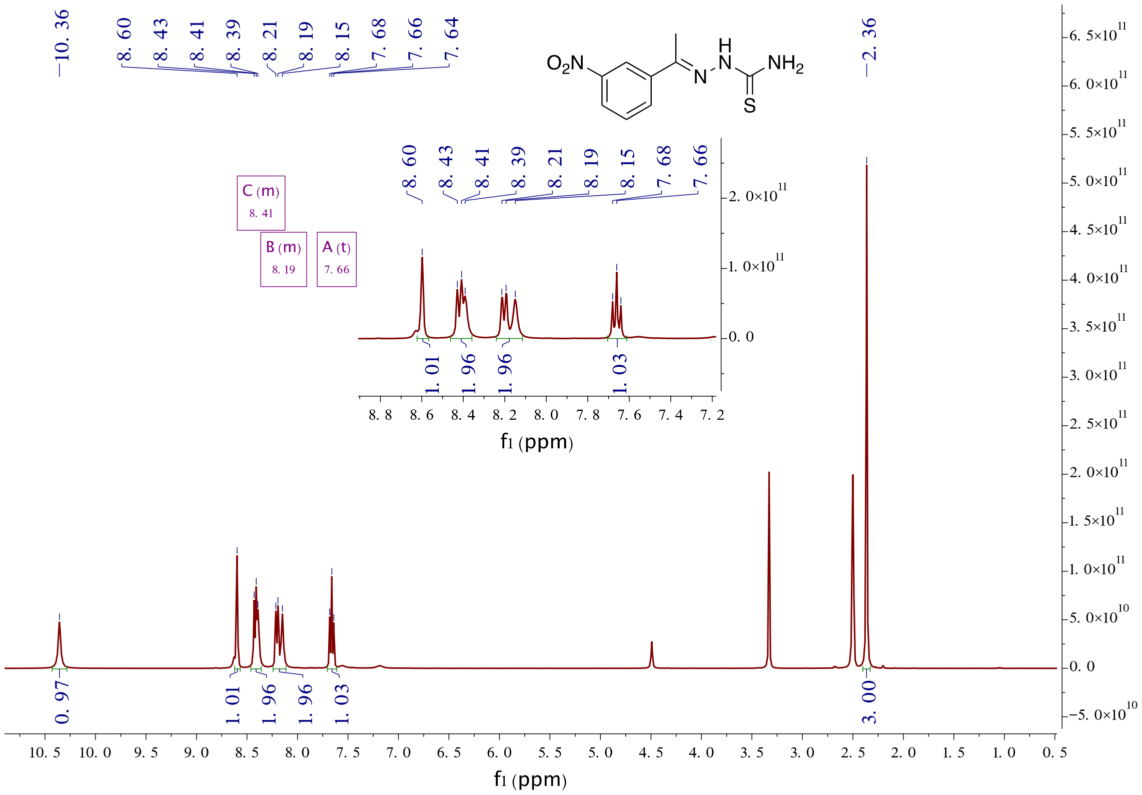

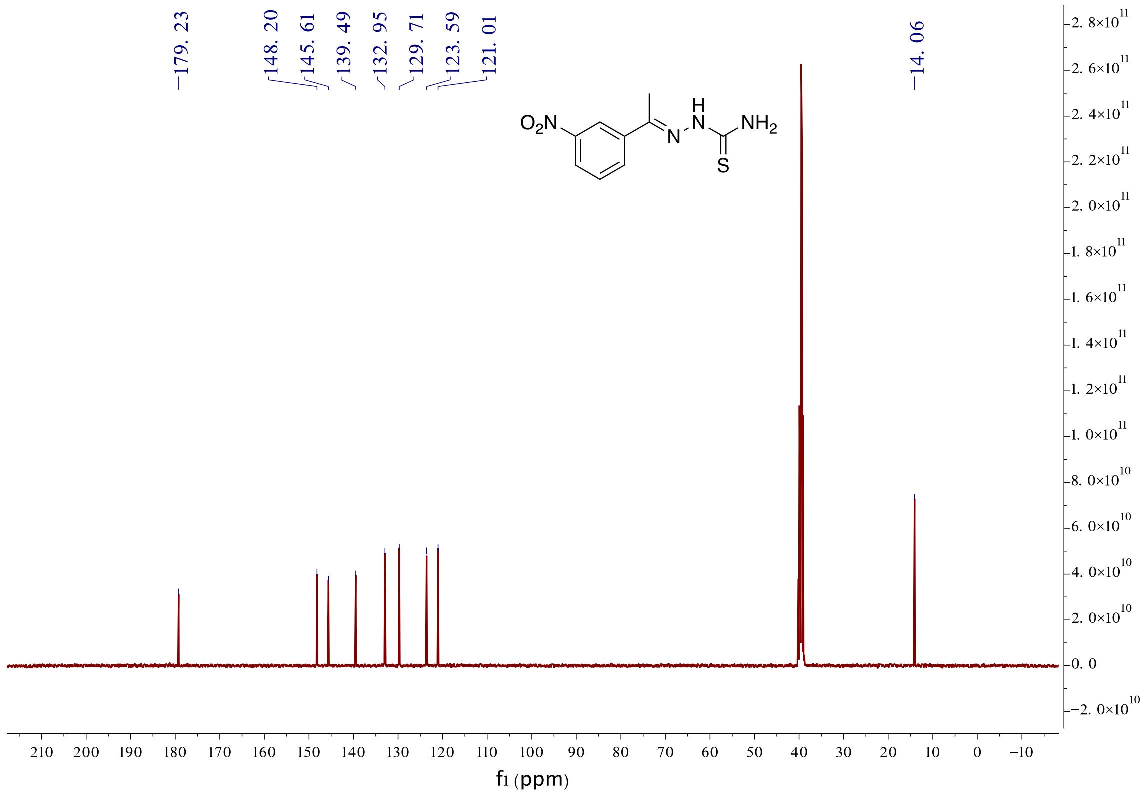

Figure S18. ^{13}C-NMR (up) and qNMR (down) of compound ***S*1h**.

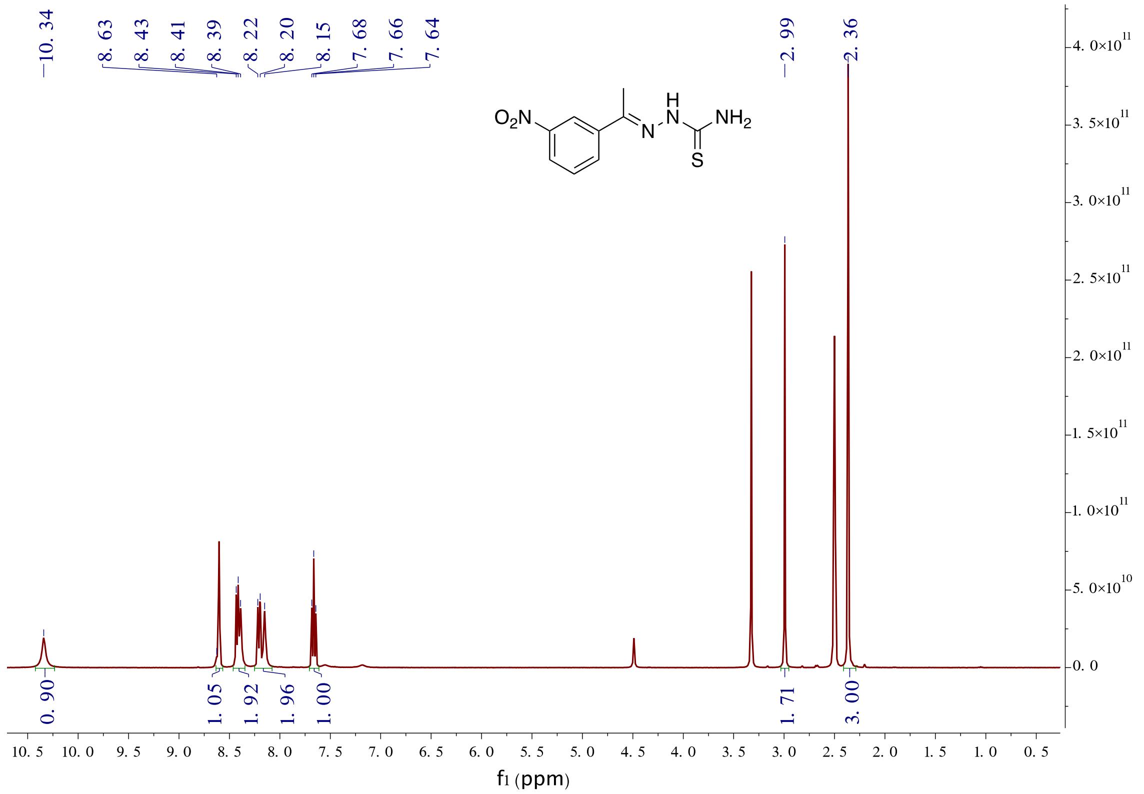

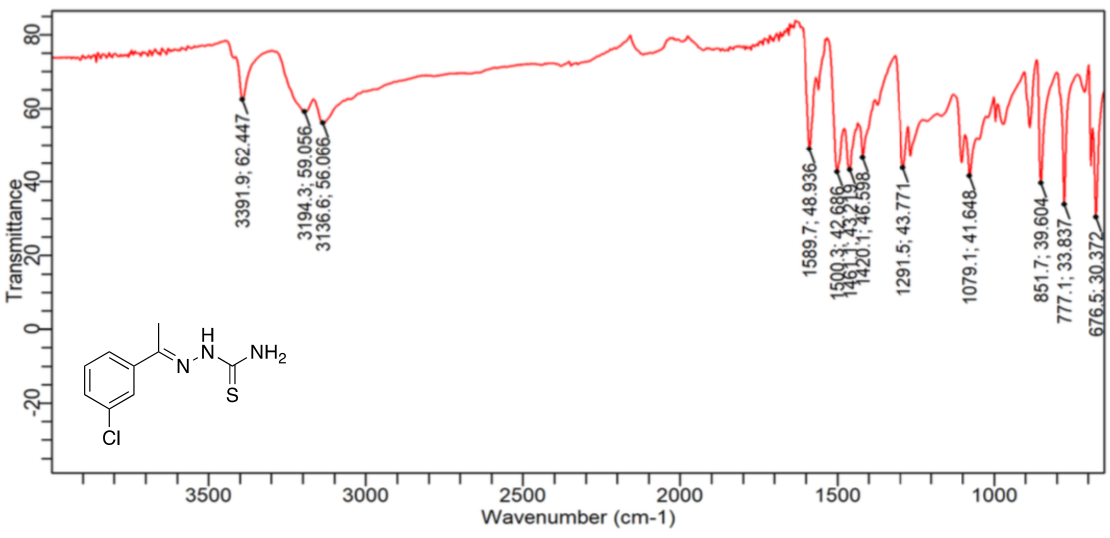

Figure S19. IR (up) and ¹H-NMR (down) of compound ***S1i***.

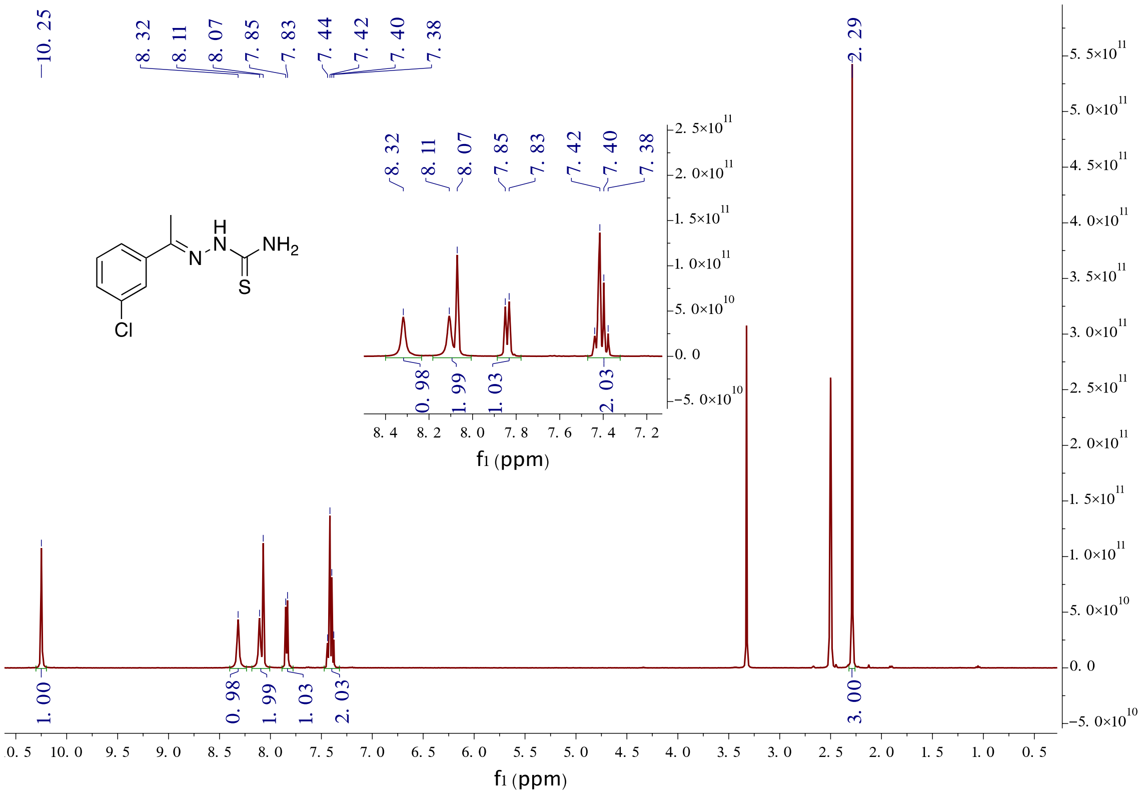

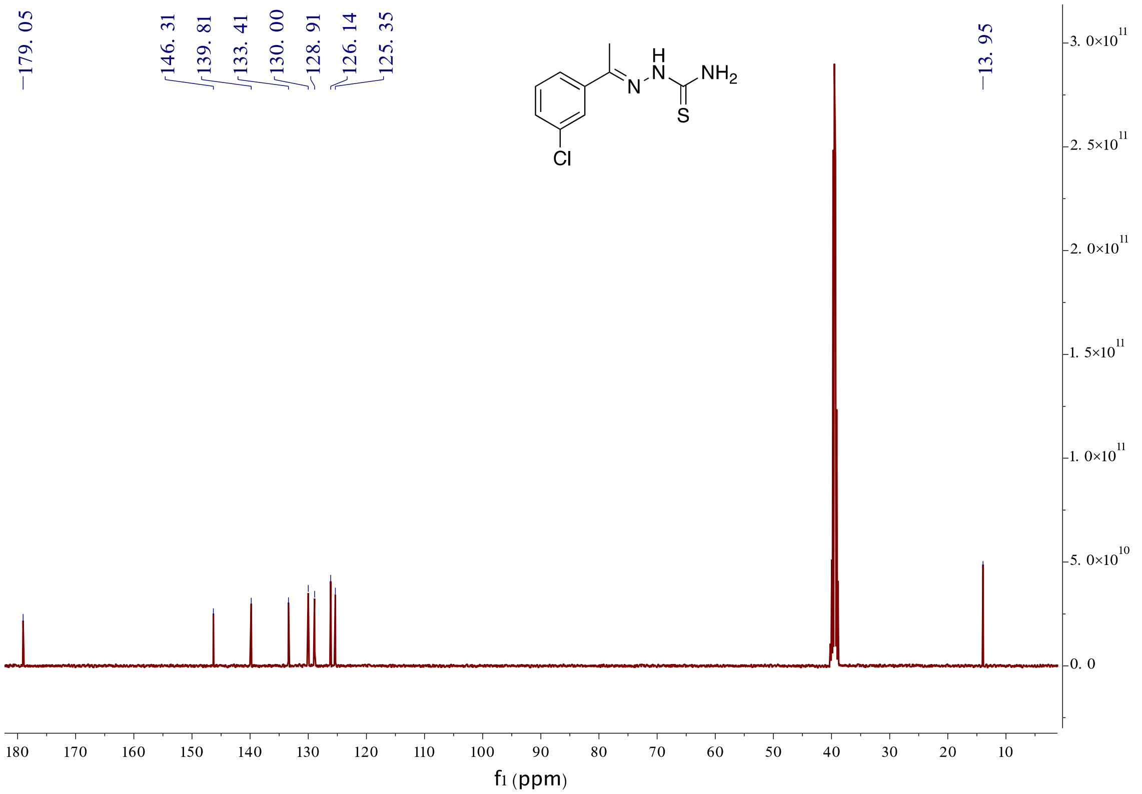

Figure S20. ^{13}C-NMR (up) and qNMR (down) of compound ***S1i***.

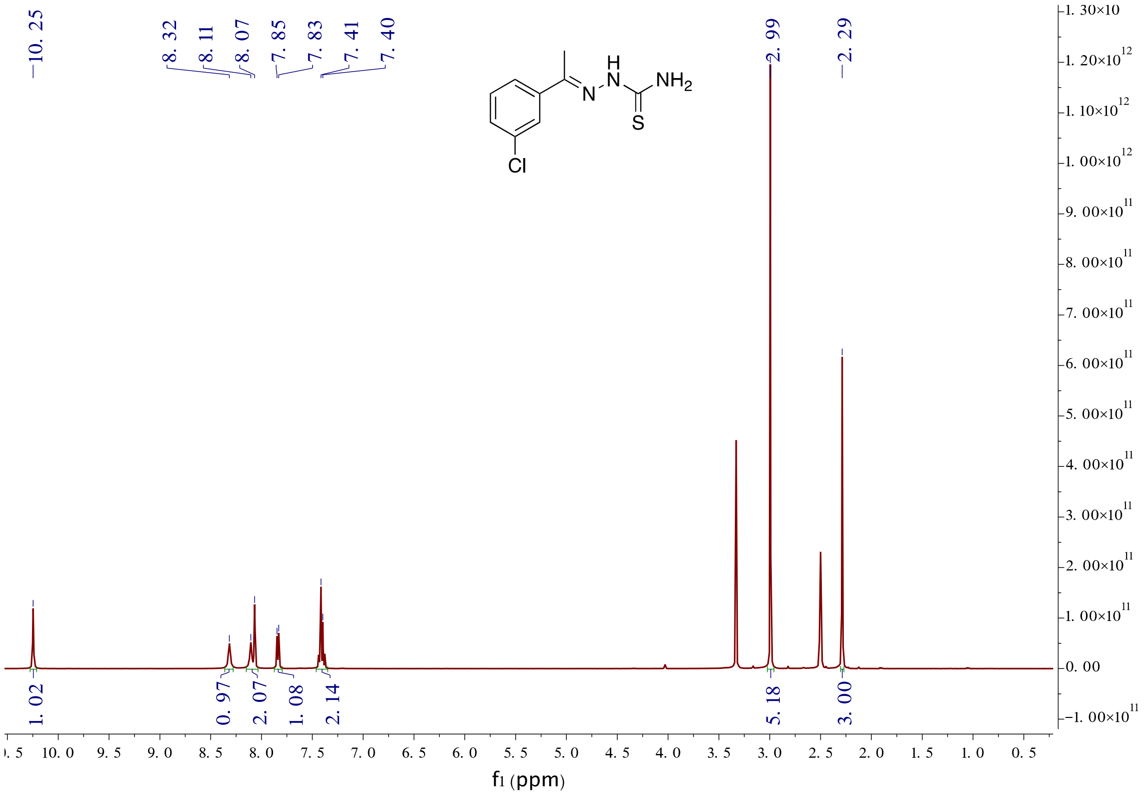

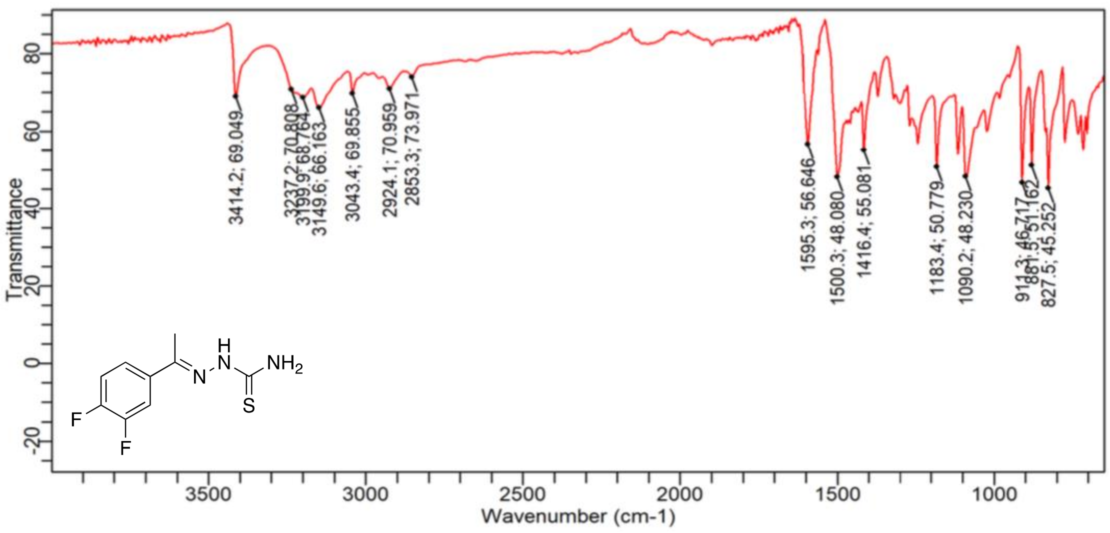

Figure S21. IR (up) and ^{1}H-NMR (down) of compound ***S*1j**.

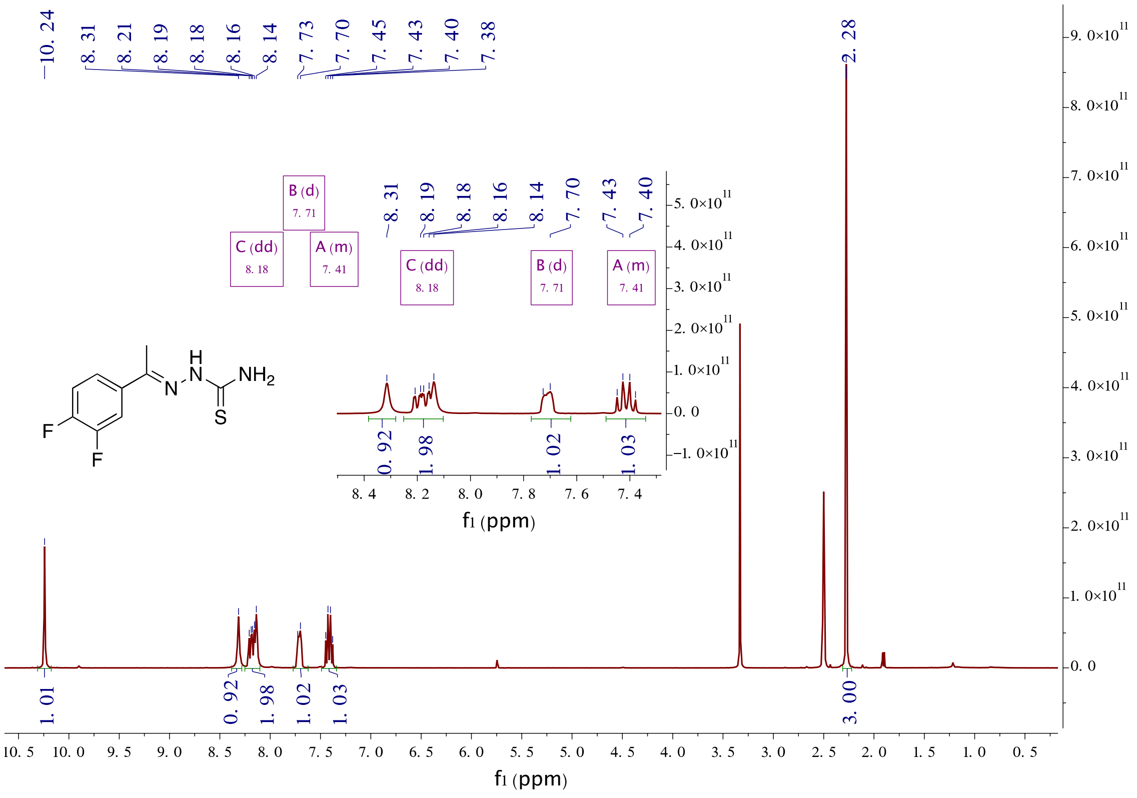

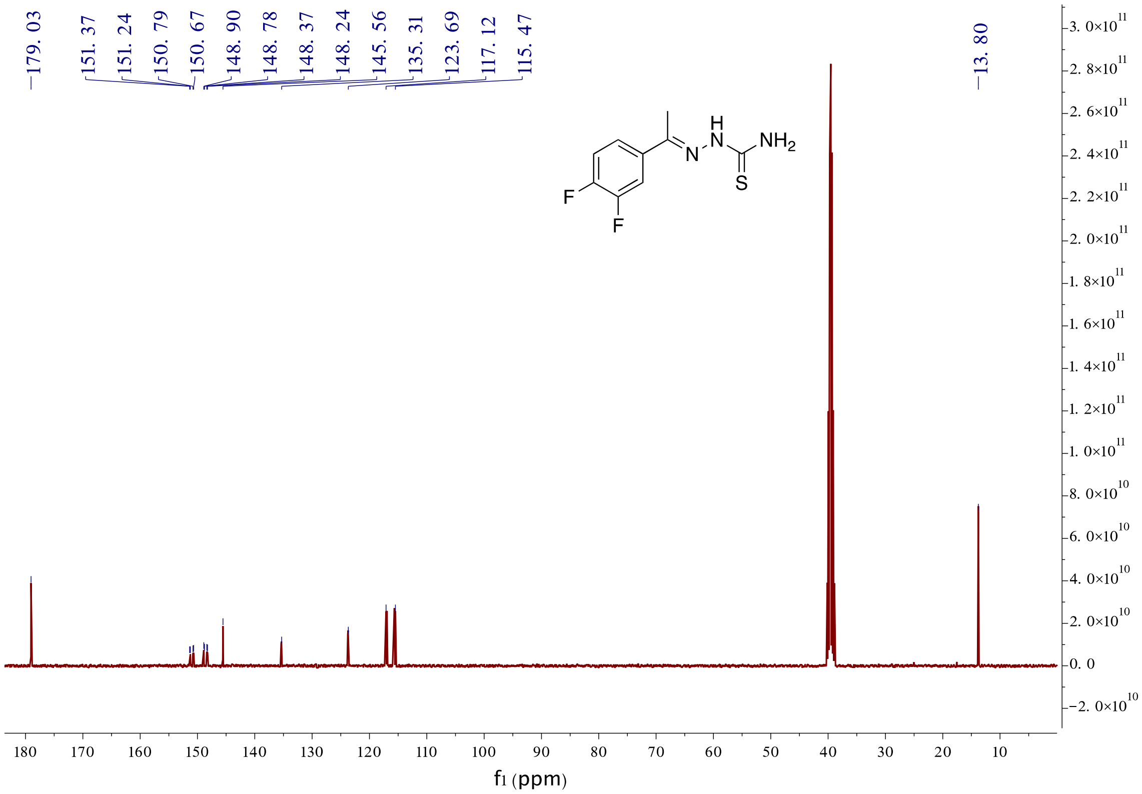

Figure S22. ^{13}C-NMR (up) and qNMR (down) of compound ***S*1j**.

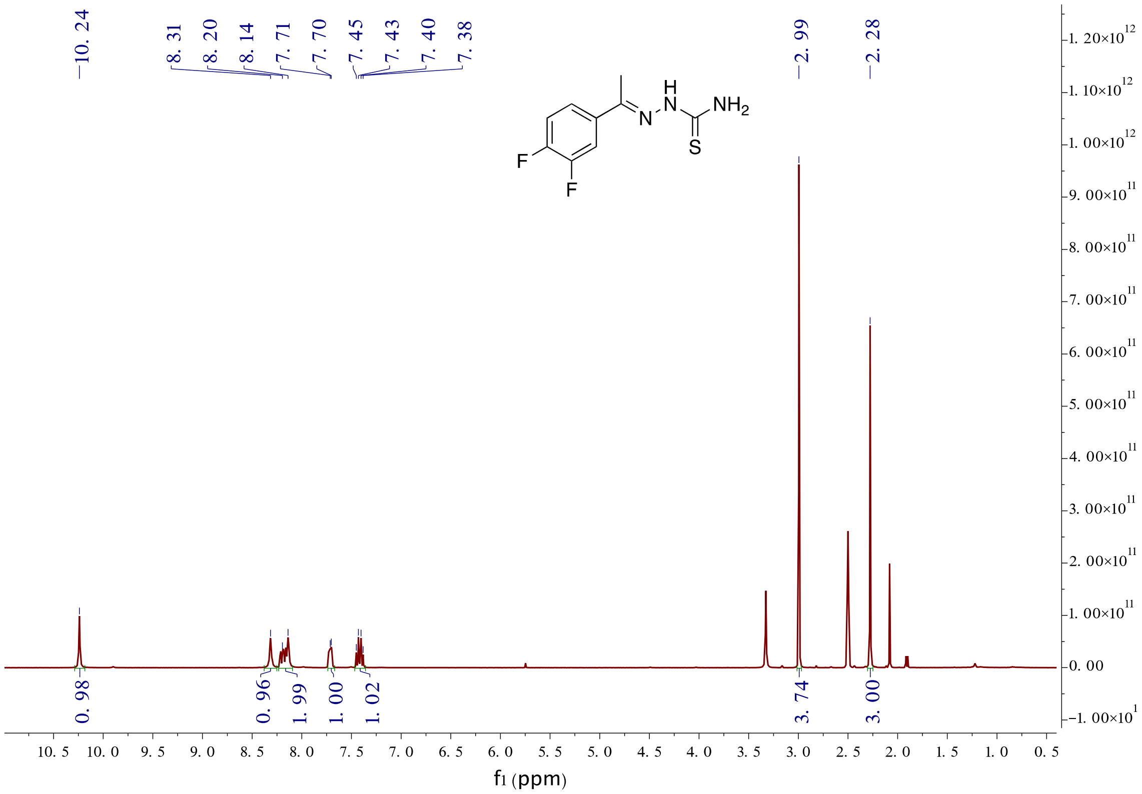

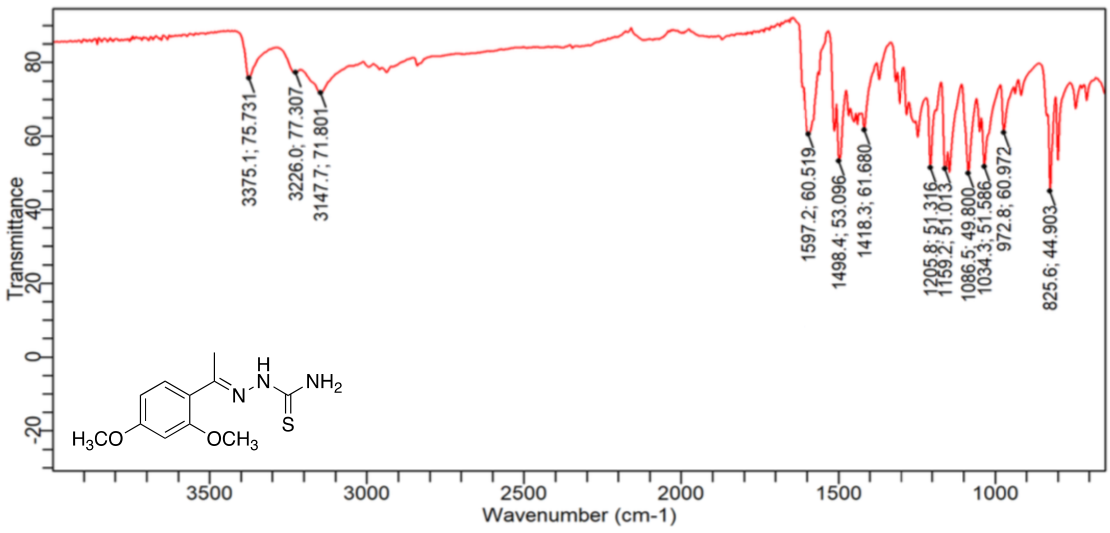

Figure S23. IR (up) and ^{1}H-NMR (down) of compound ***S*1k**.

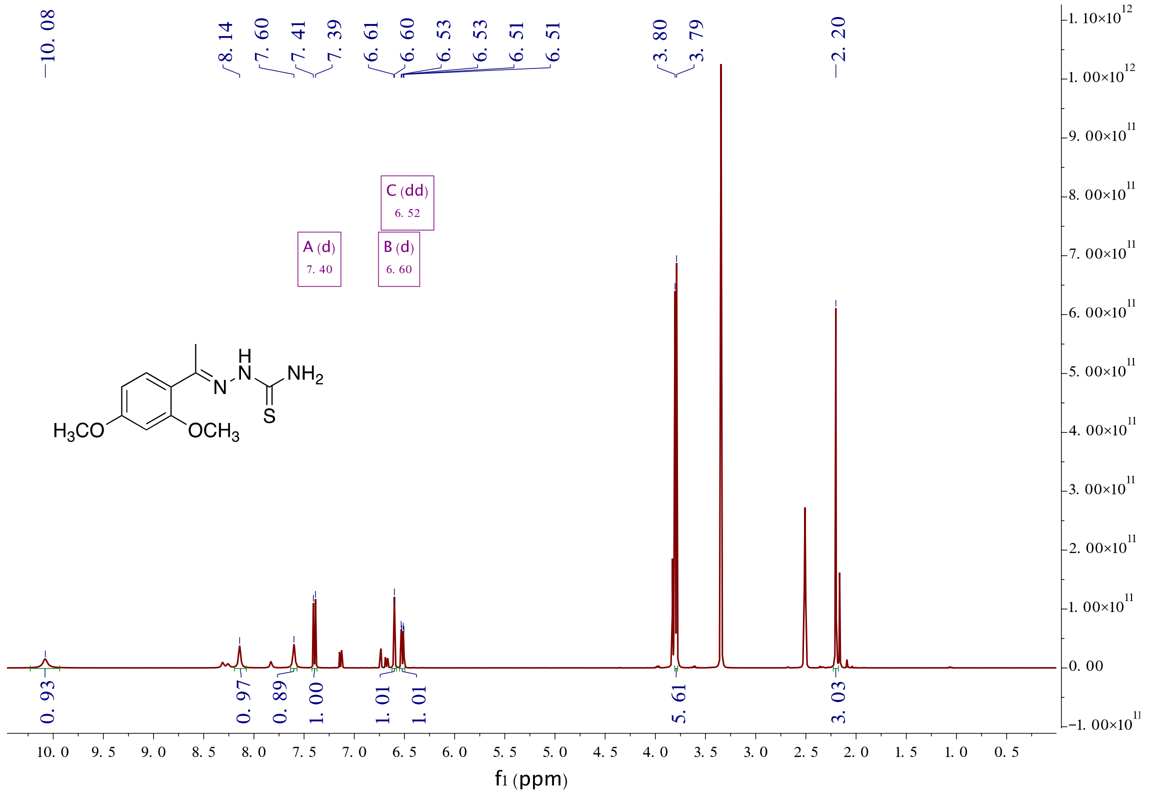

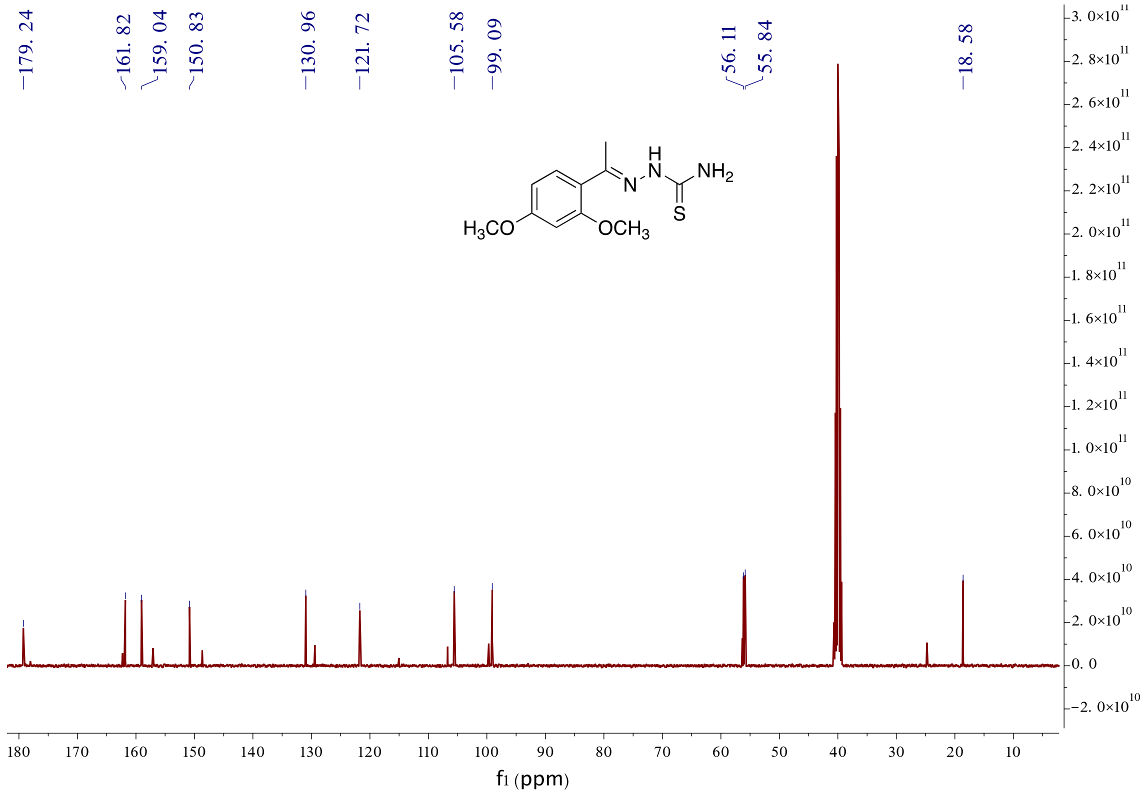

Figure S24. ^{13}C-NMR (up) and qNMR (down) of compound ***S*1k**.

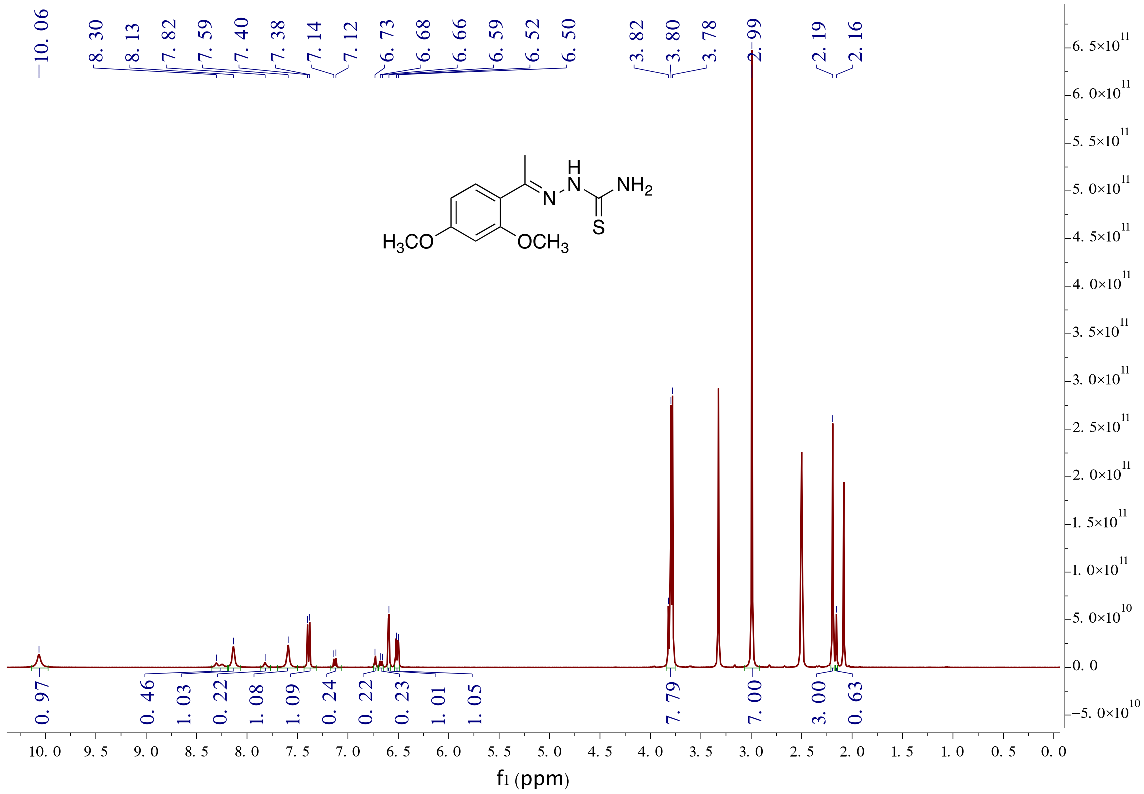

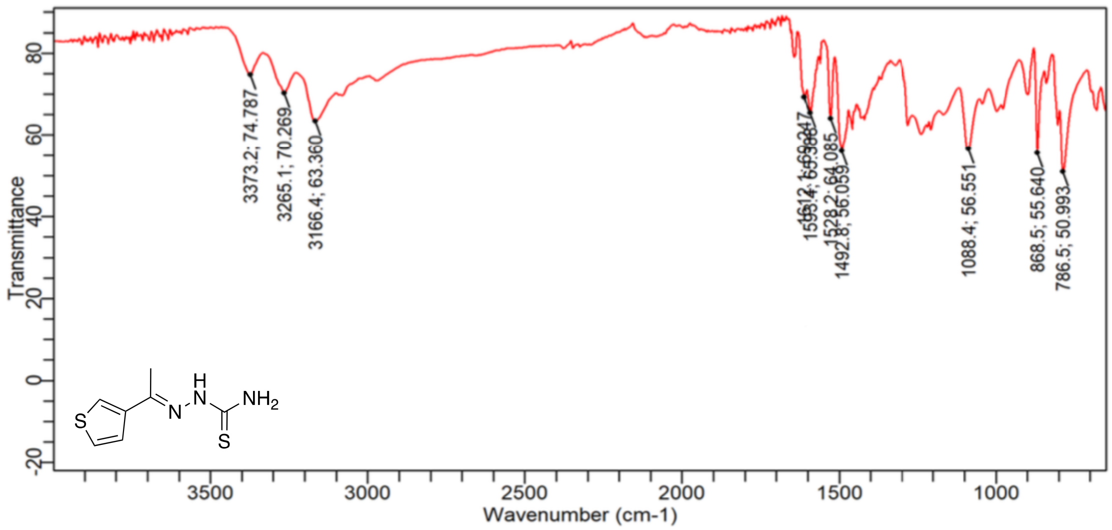

Figure S25. IR (up) and ^{1}H-NMR (down) of compound ***S*1l**.

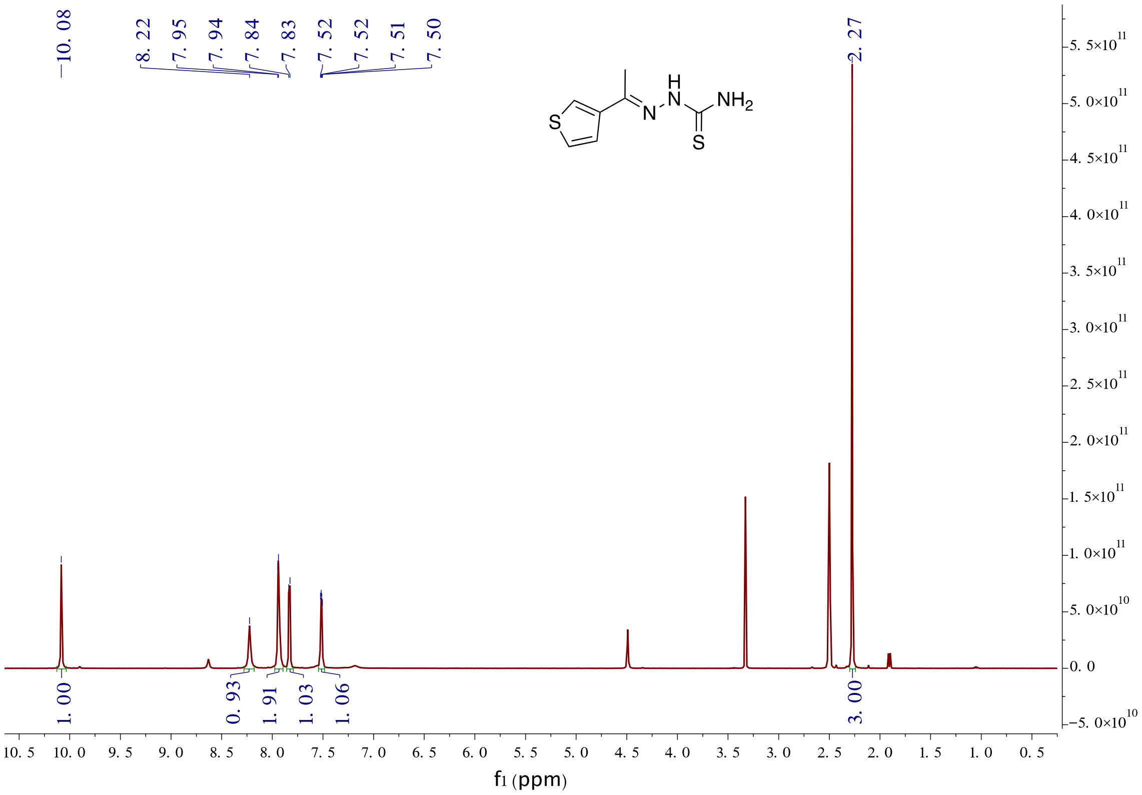

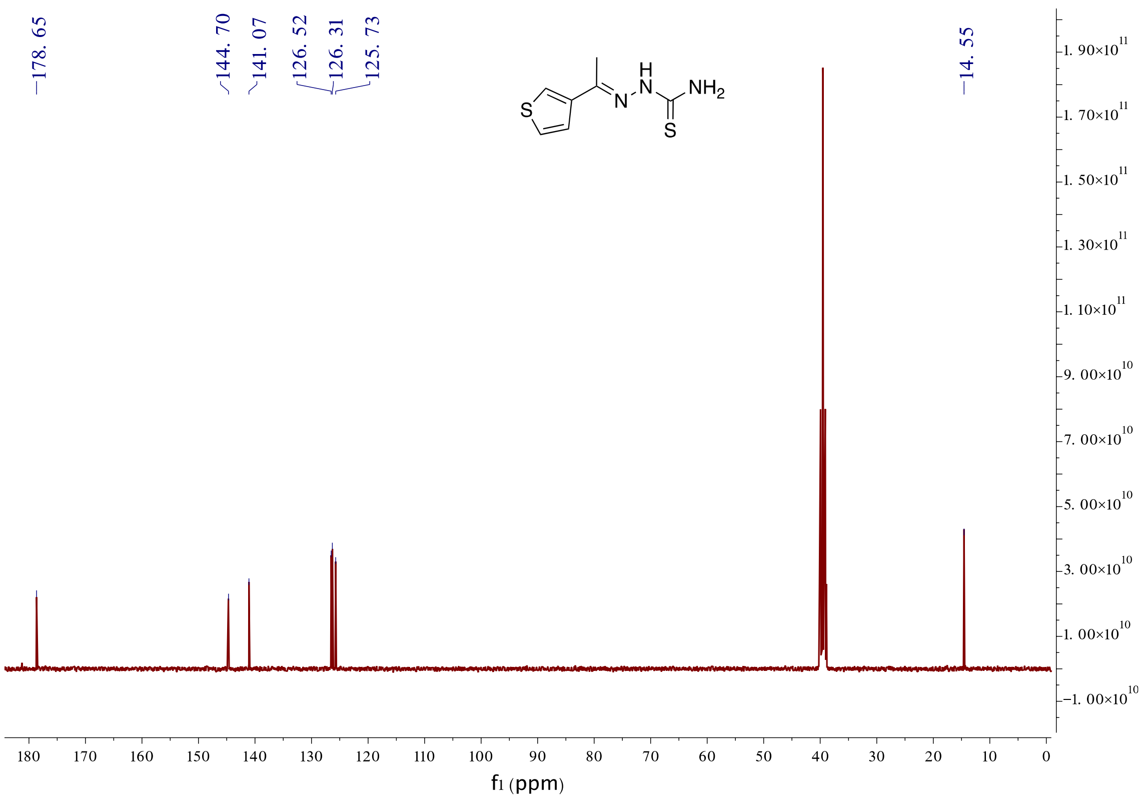

Figure S26. ^{13}C-NMR (up) and qNMR (down) of compound ***S*1l**.

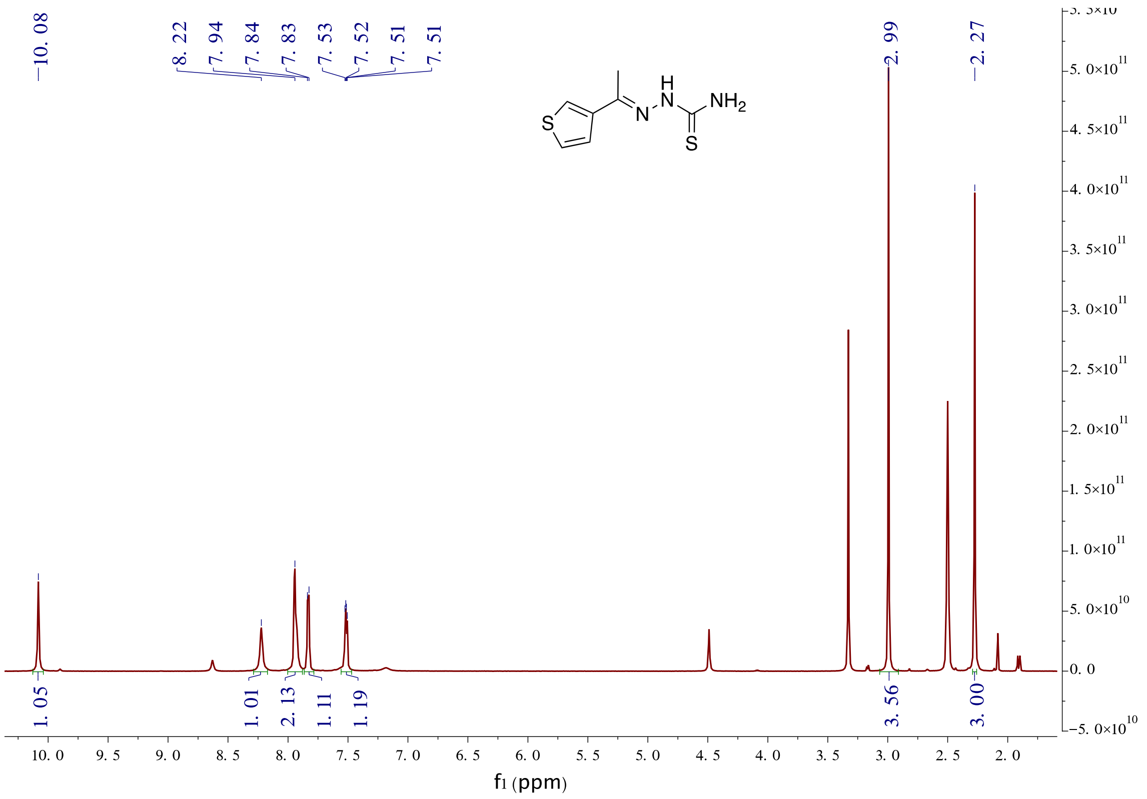

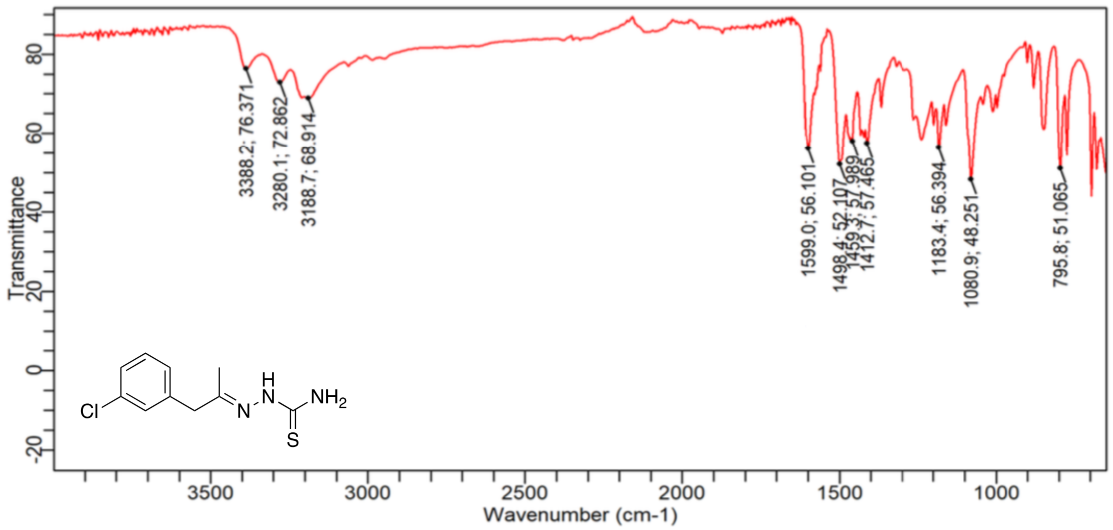

Figure S27. IR (up) and ^{1}H-NMR (down) of compound ***S*1m**.

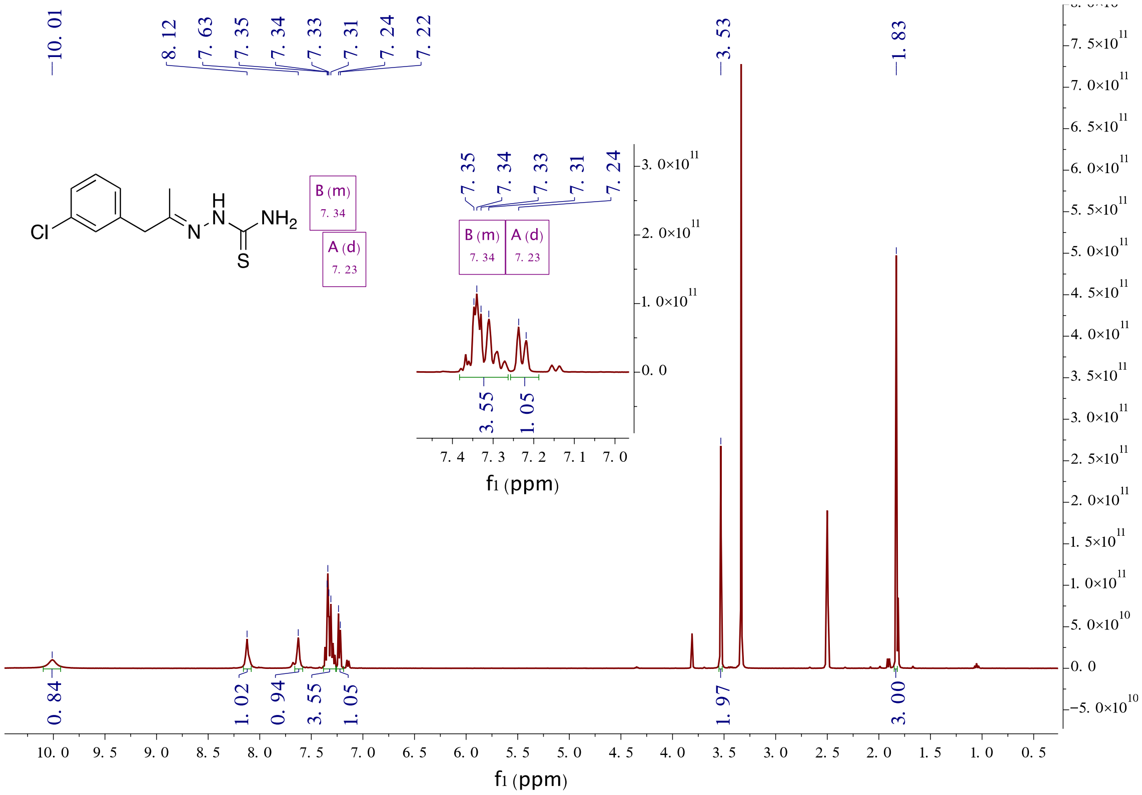

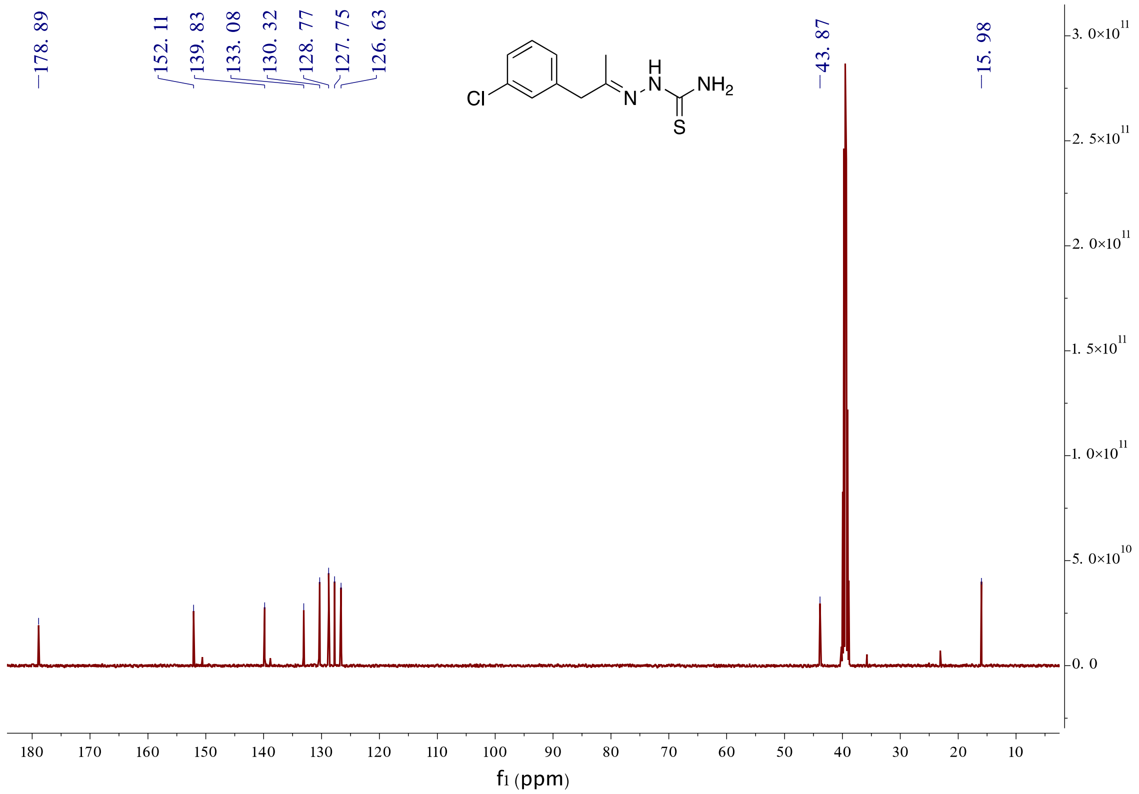

Figure S28. ^{13}C-NMR (up) and qNMR (down) of compound ***S*1m**.

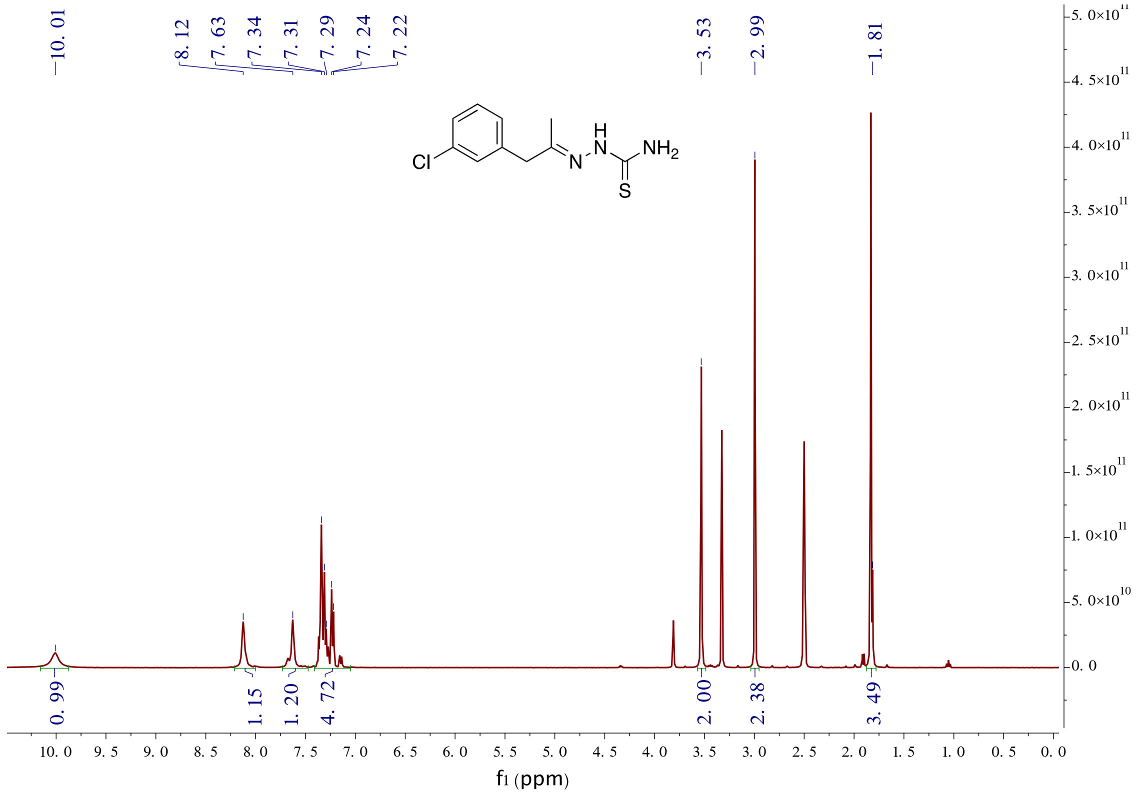

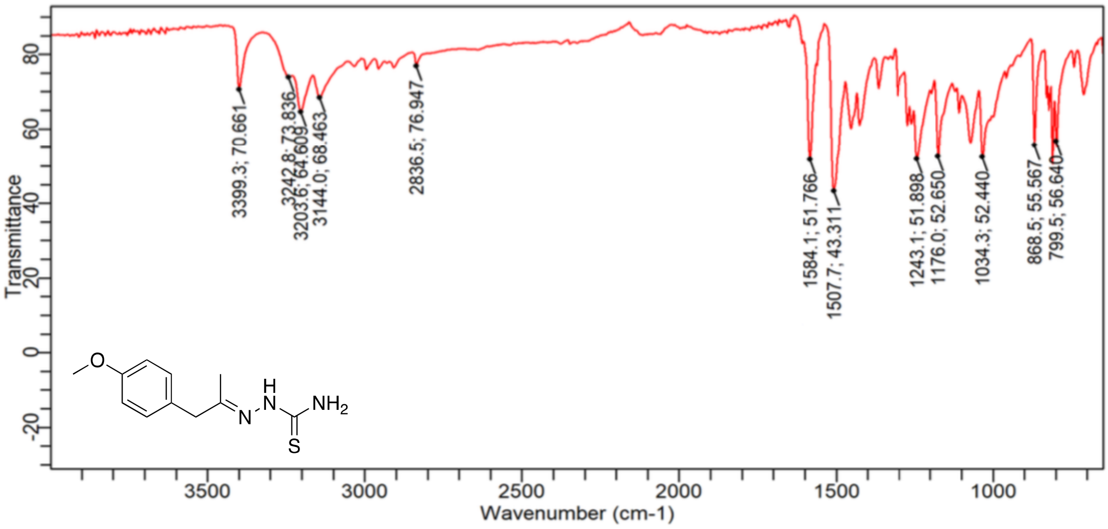

Figure S29. IR (up) and ^{1}H-NMR (down) of compound ***S*1n**.

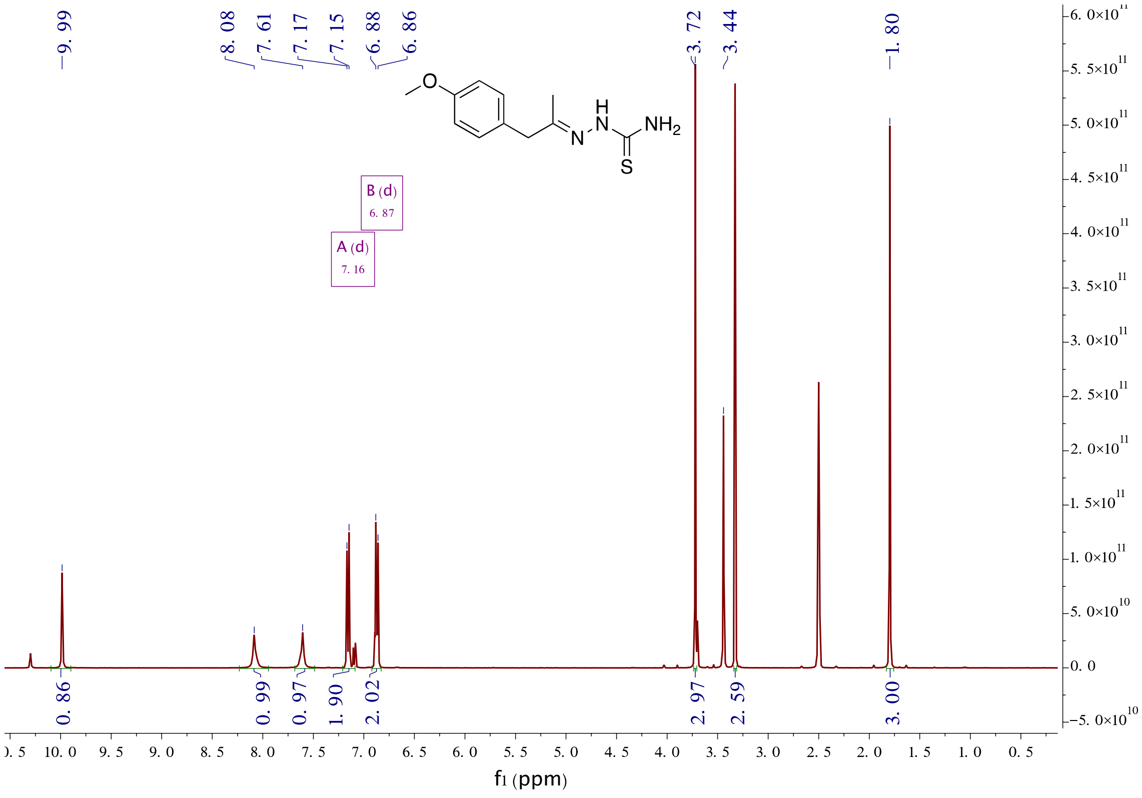

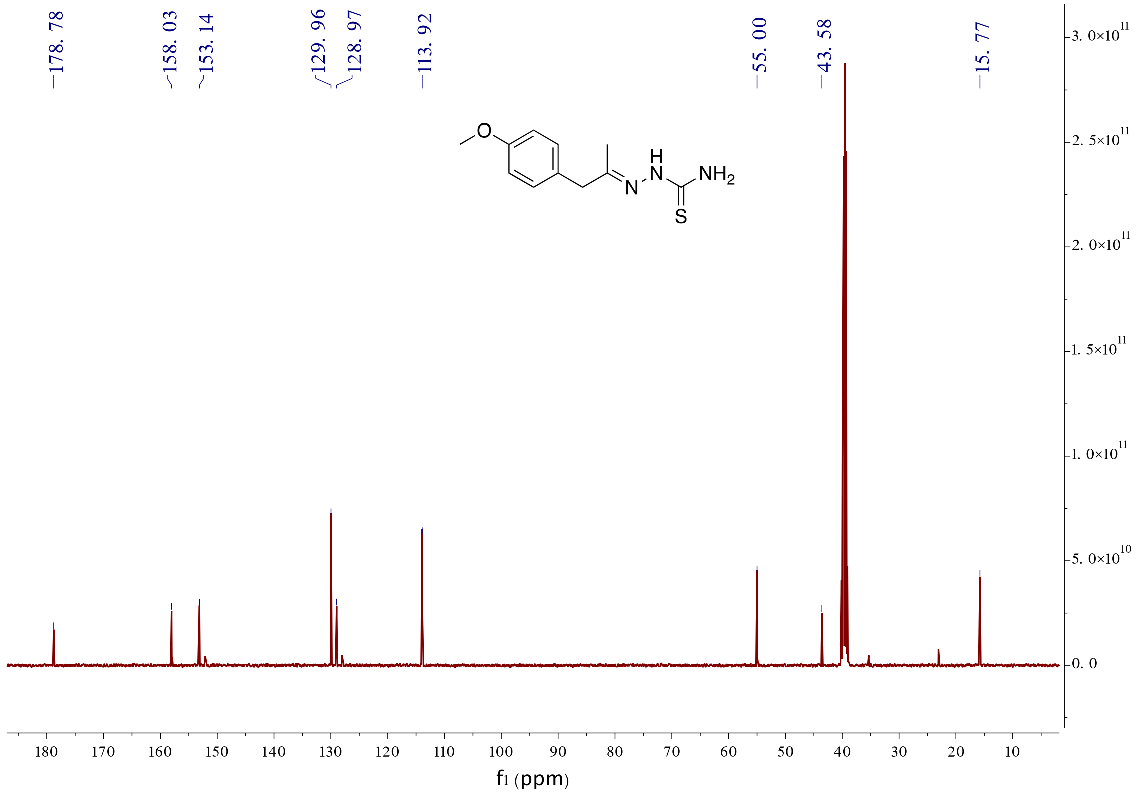

Figure S30. ^{13}C-NMR (up) and qNMR (down) of compound ***S*1n**.

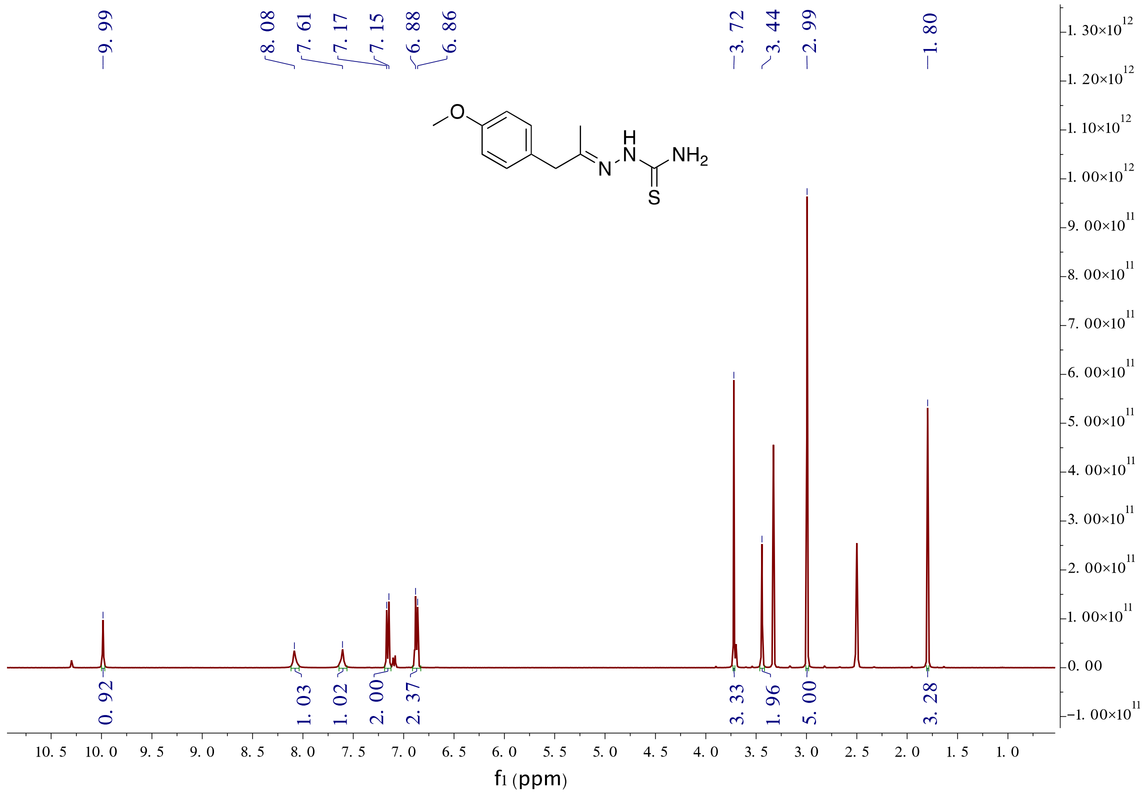

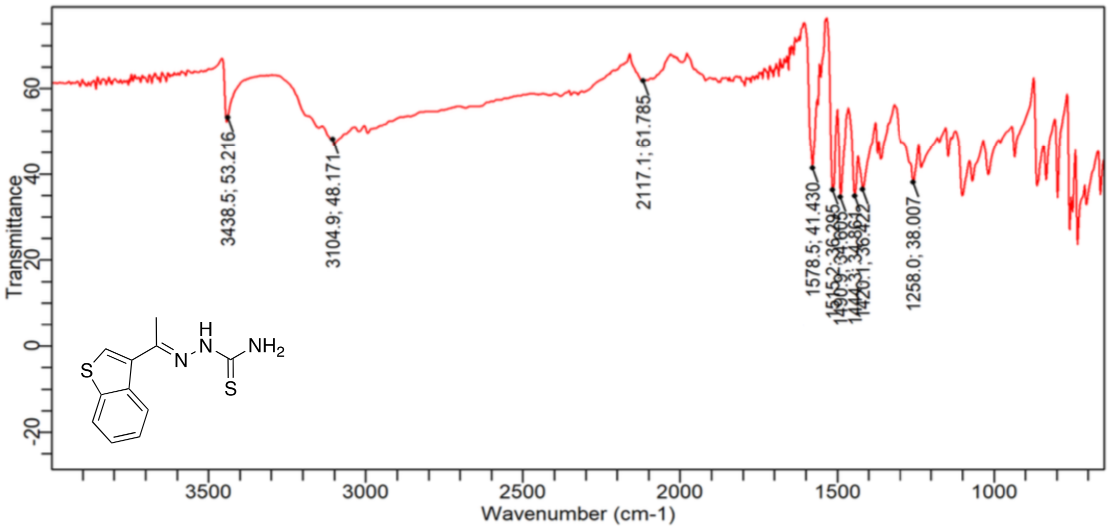

Figure S31. IR (up) and ^{1}H-NMR (down) of compound ***S1o***.

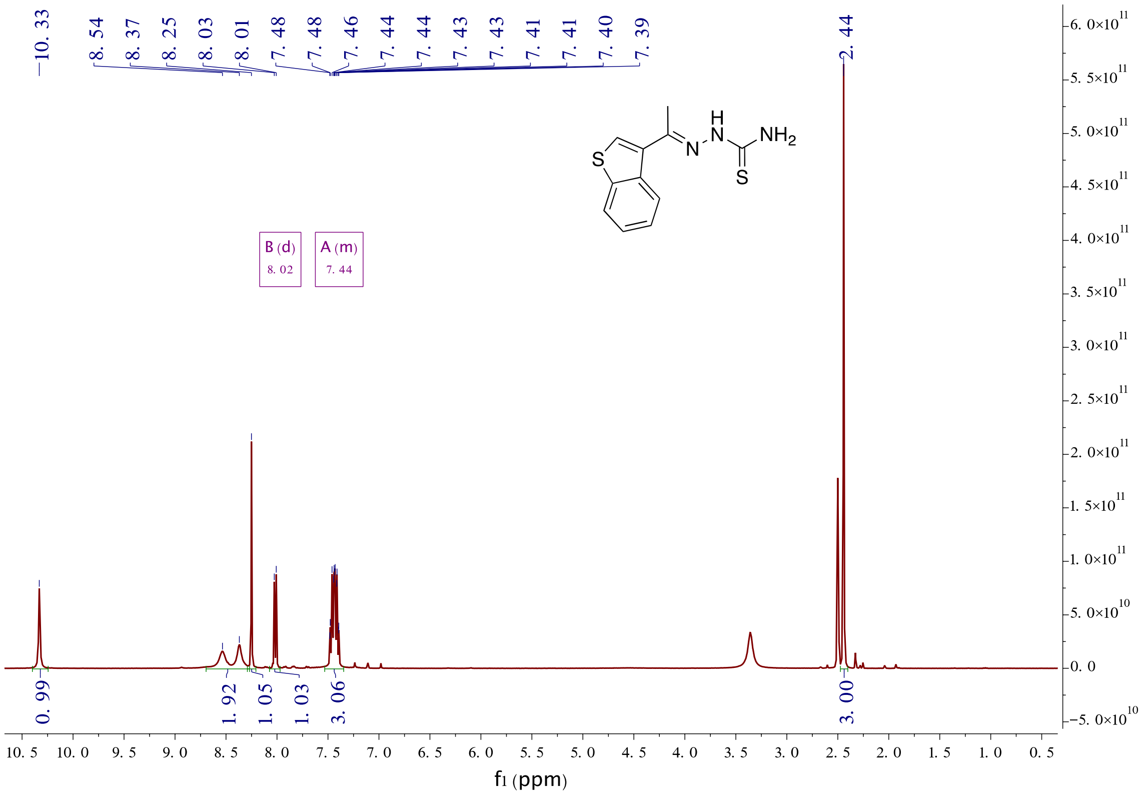

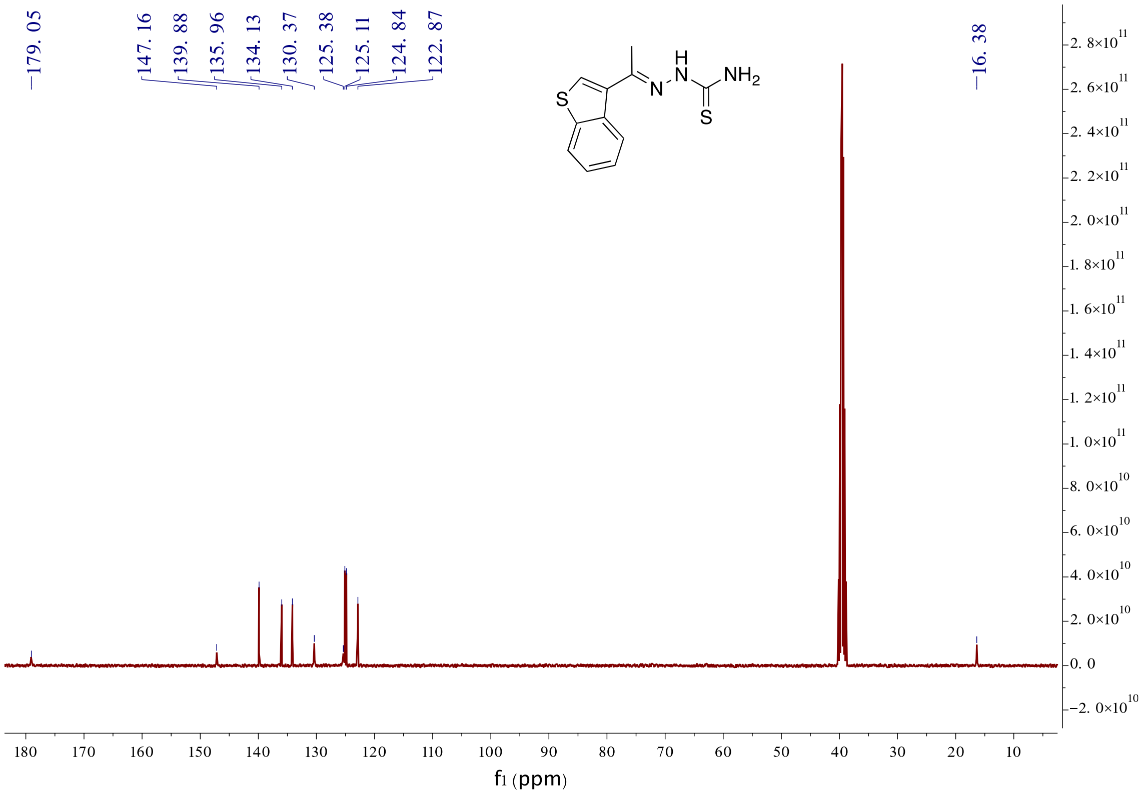

Figure S32. ^{13}C-NMR (up) and qNMR (down) of compound ***S*1o**.

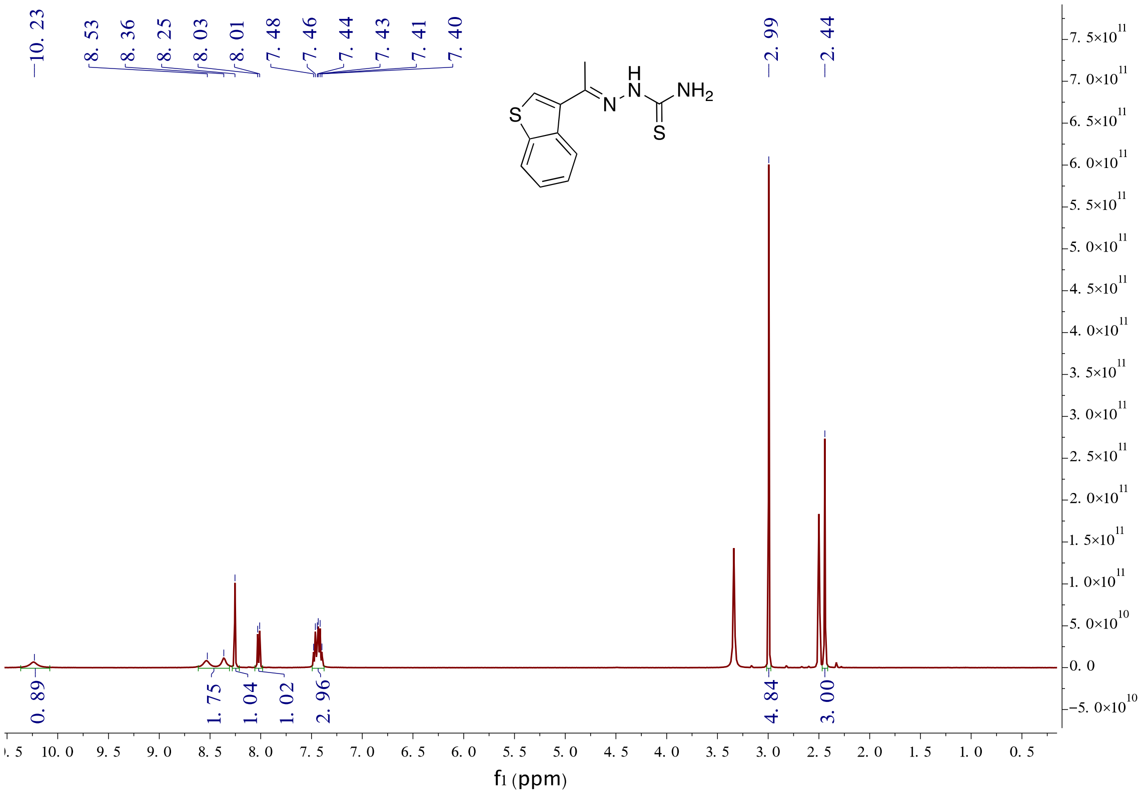

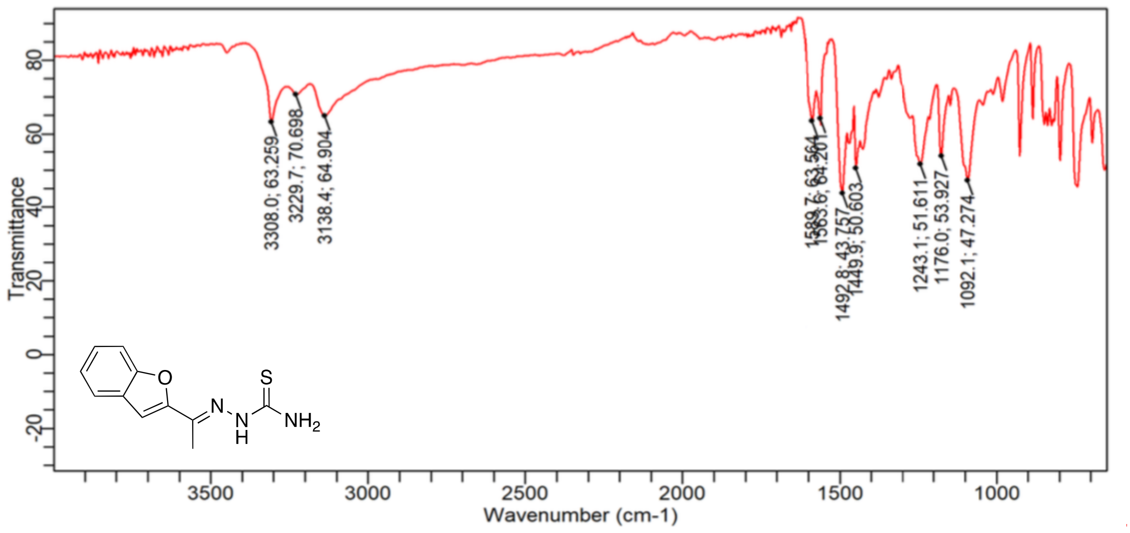

Figure S33. IR (up) and ^{1}H-NMR (down) of compound ***S*1p**.

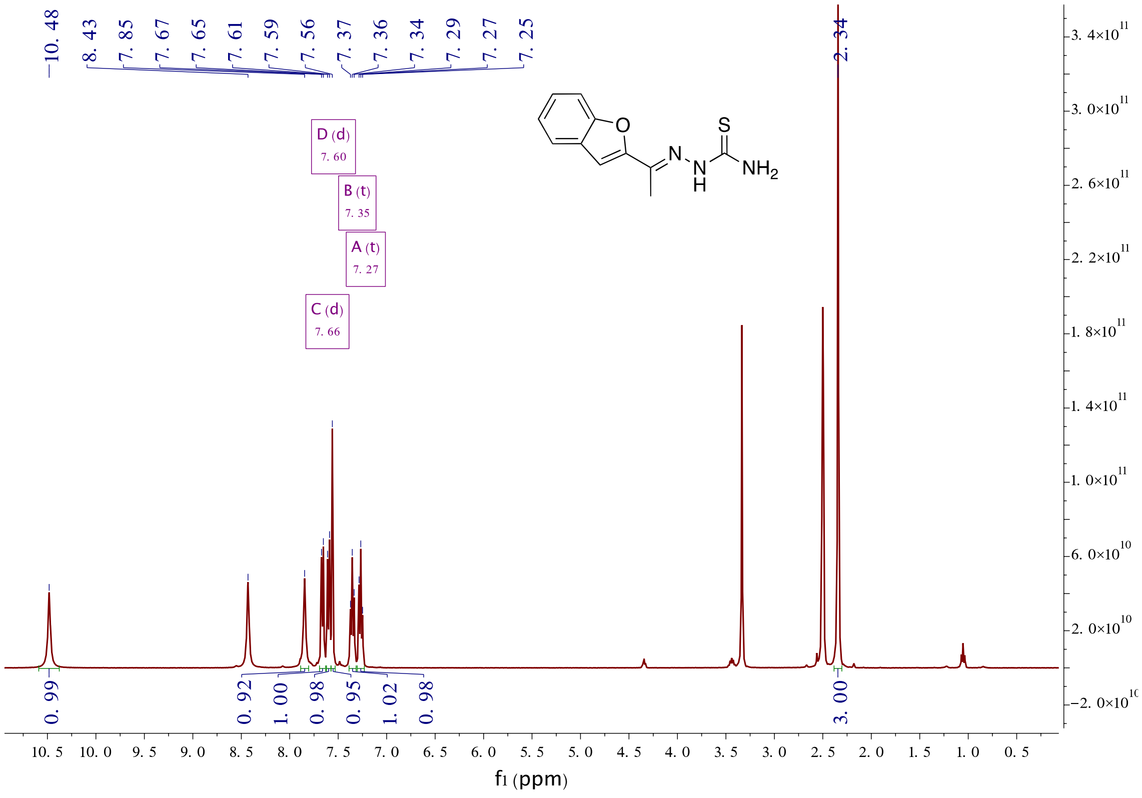

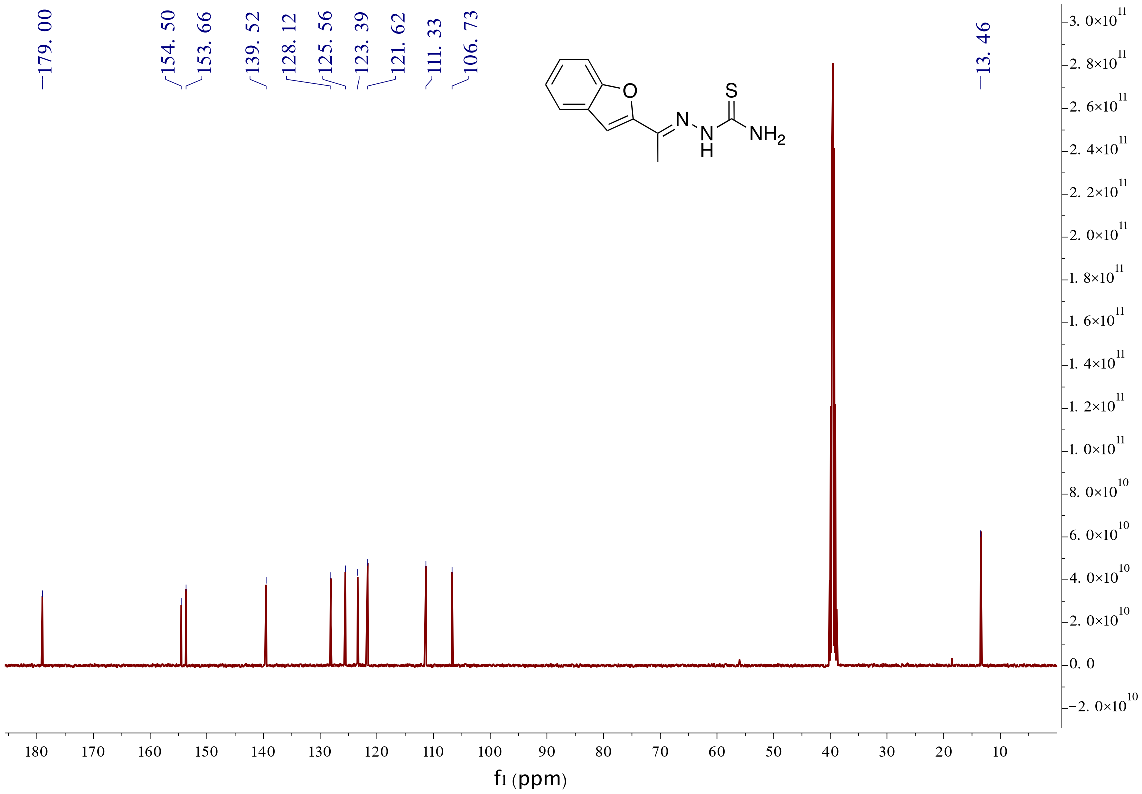

Figure S34. ^{13}C-NMR (up) and qNMR (down) of compound ***S*1p**.

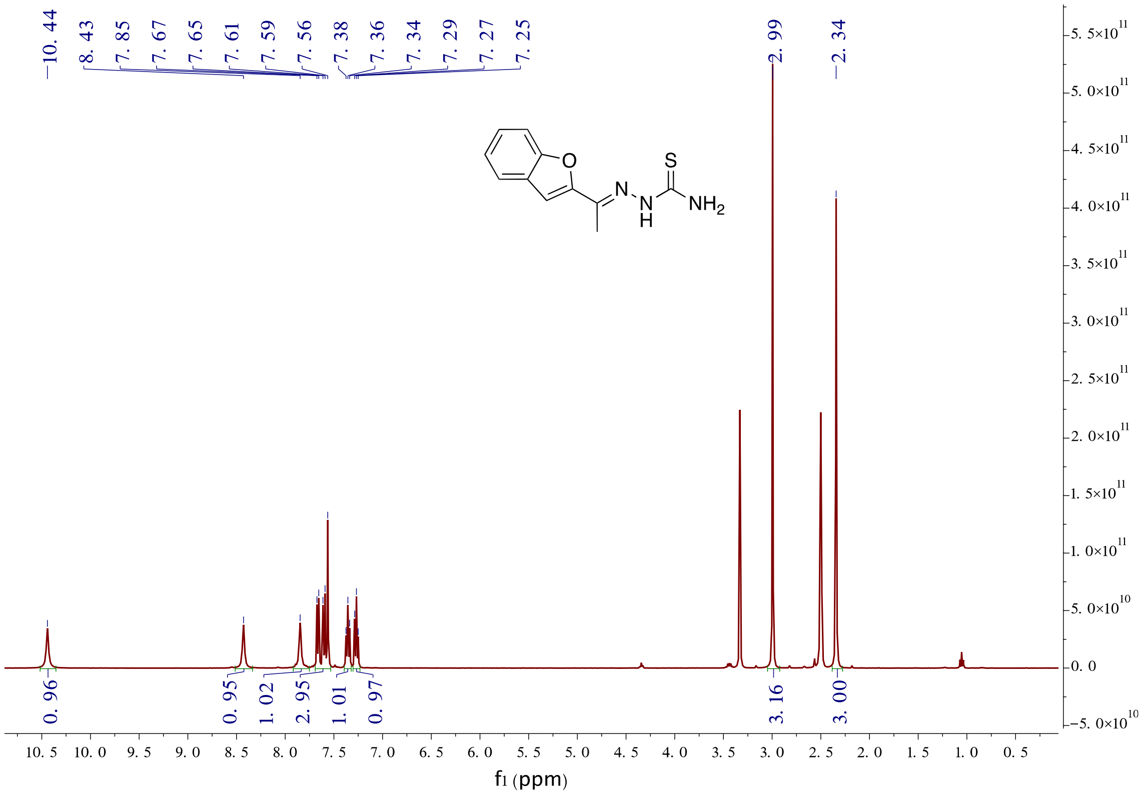

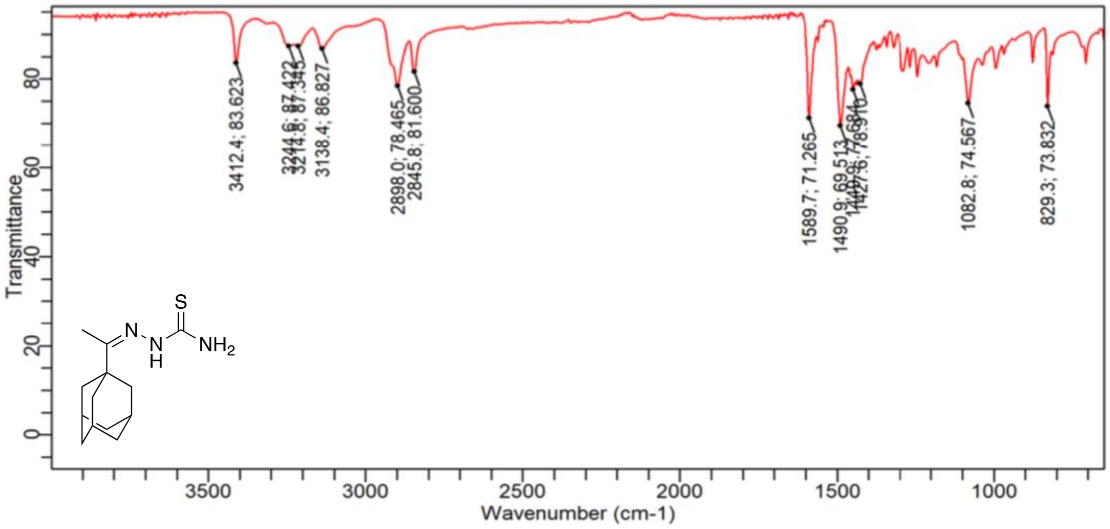

Figure S35. IR (up) and ^{1}H-NMR (down) of compound ***S*1q**.

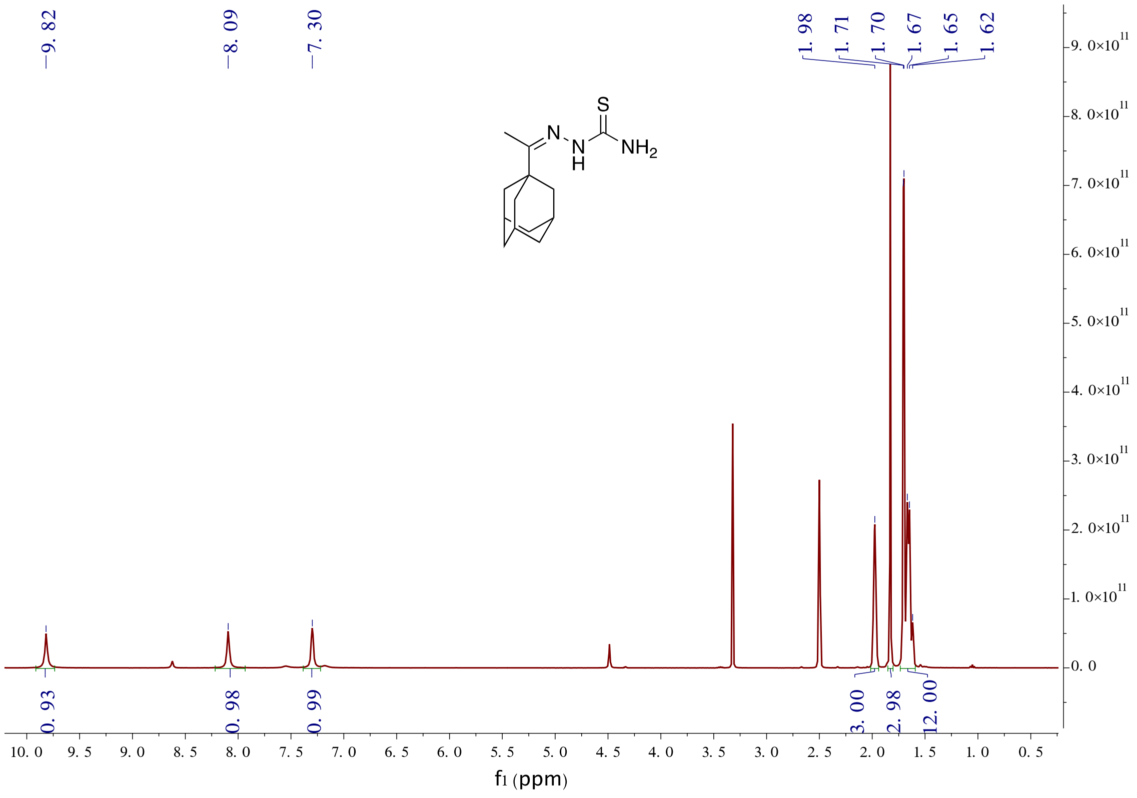

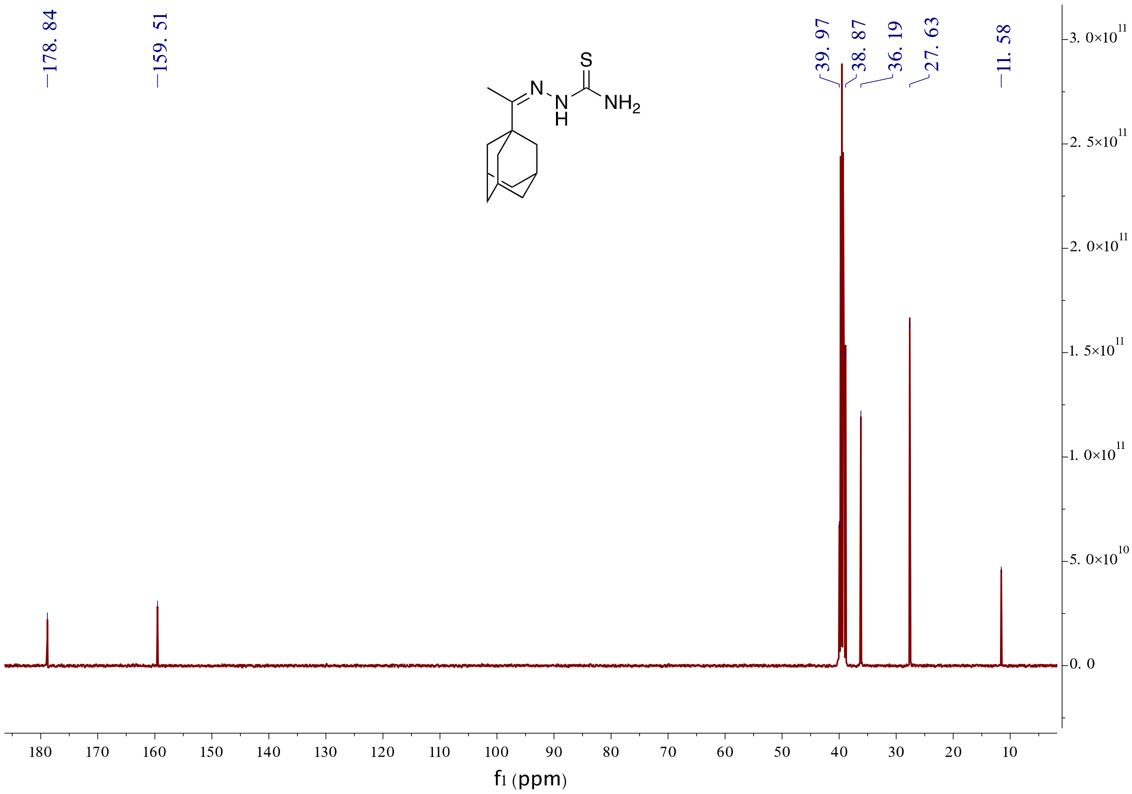

Figure S36. ^{13}C-NMR (up) and qNMR (down) of compound ***S*1q**.

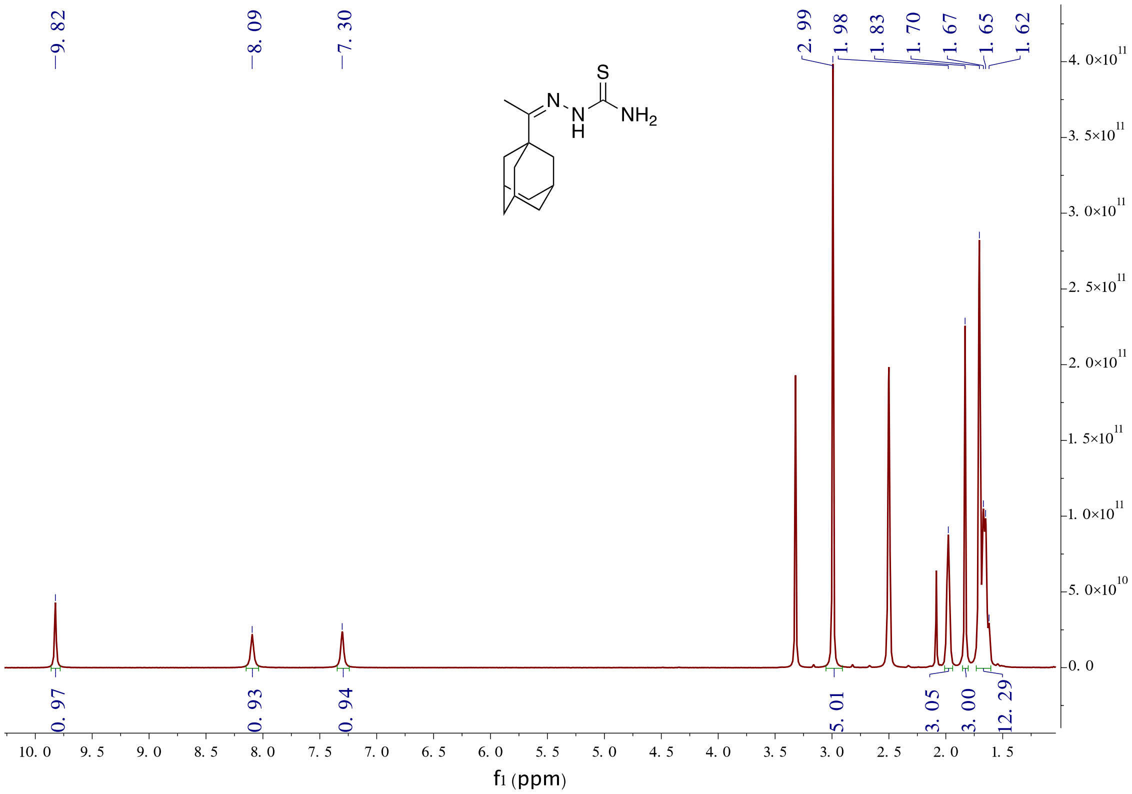

b. *Se*1 series

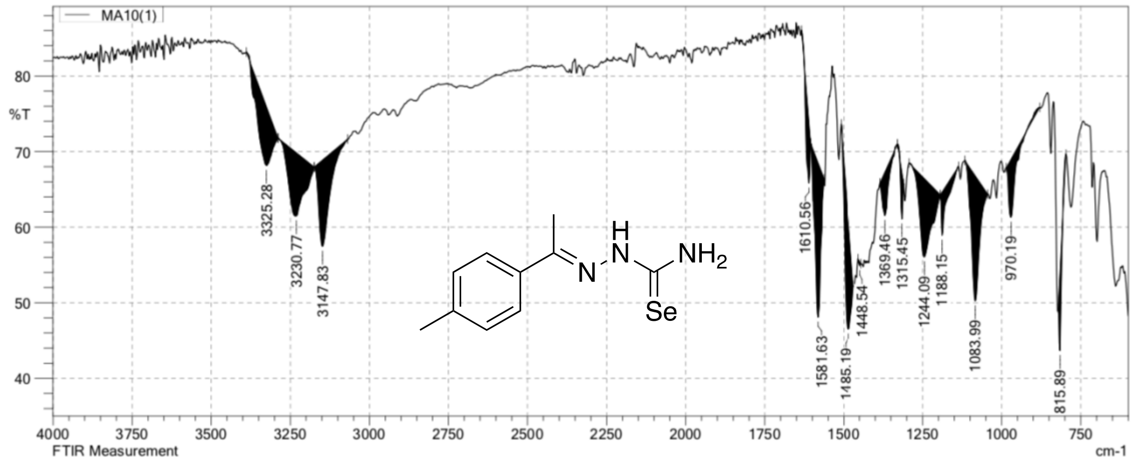

Figure S37. IR (up) and ^{1}H-NMR (down) of compound ***Se*1a**.

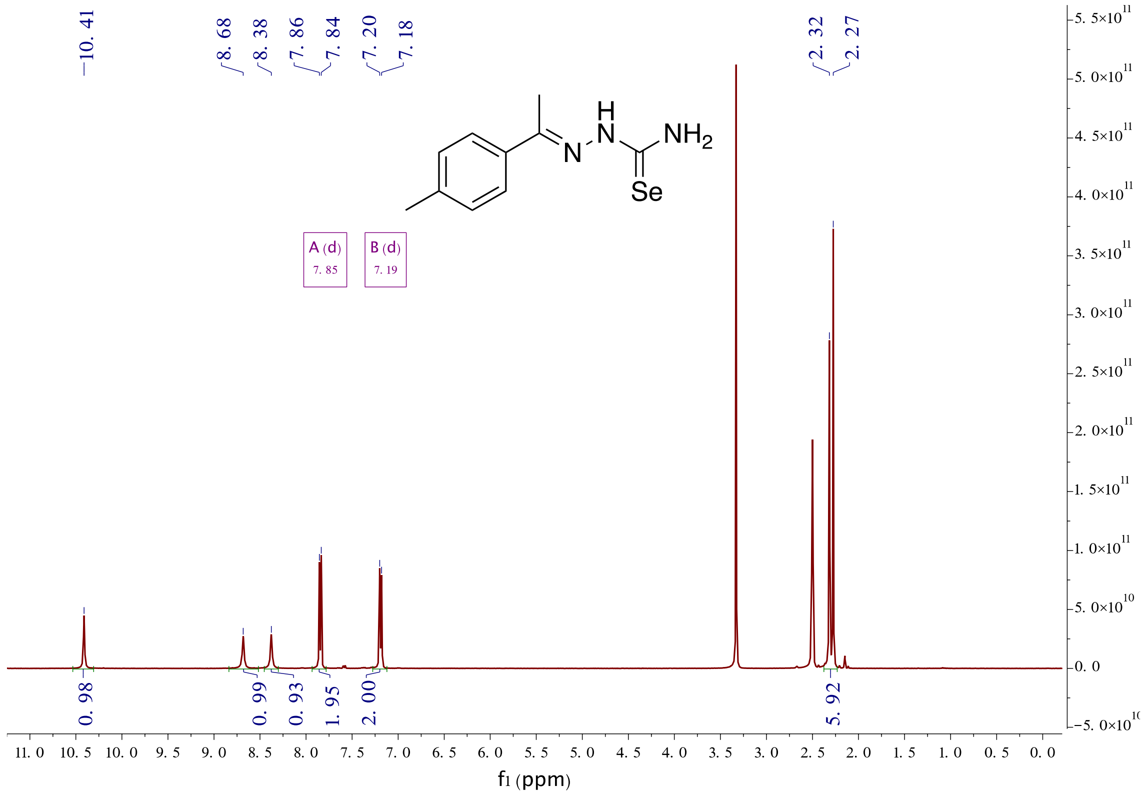

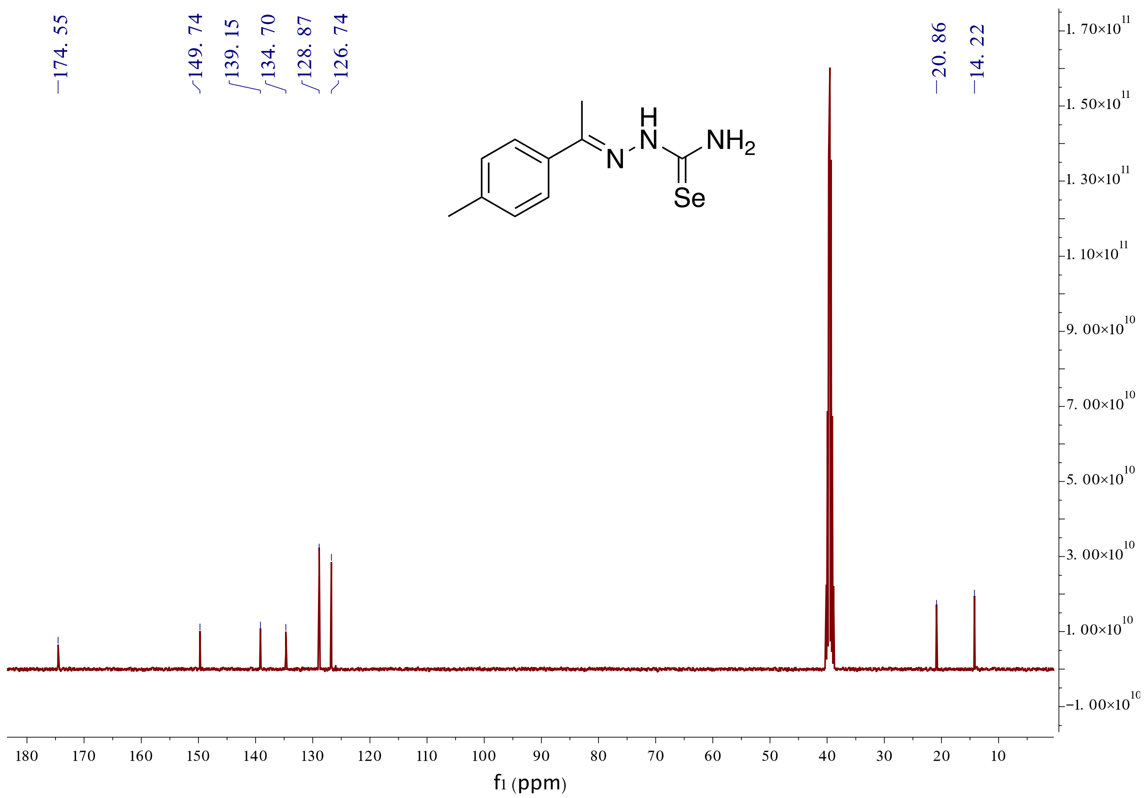

Figure S38. ^{13}C-NMR (up) and ^{77}Se-NMR (down) of compound ***Se*1a**.

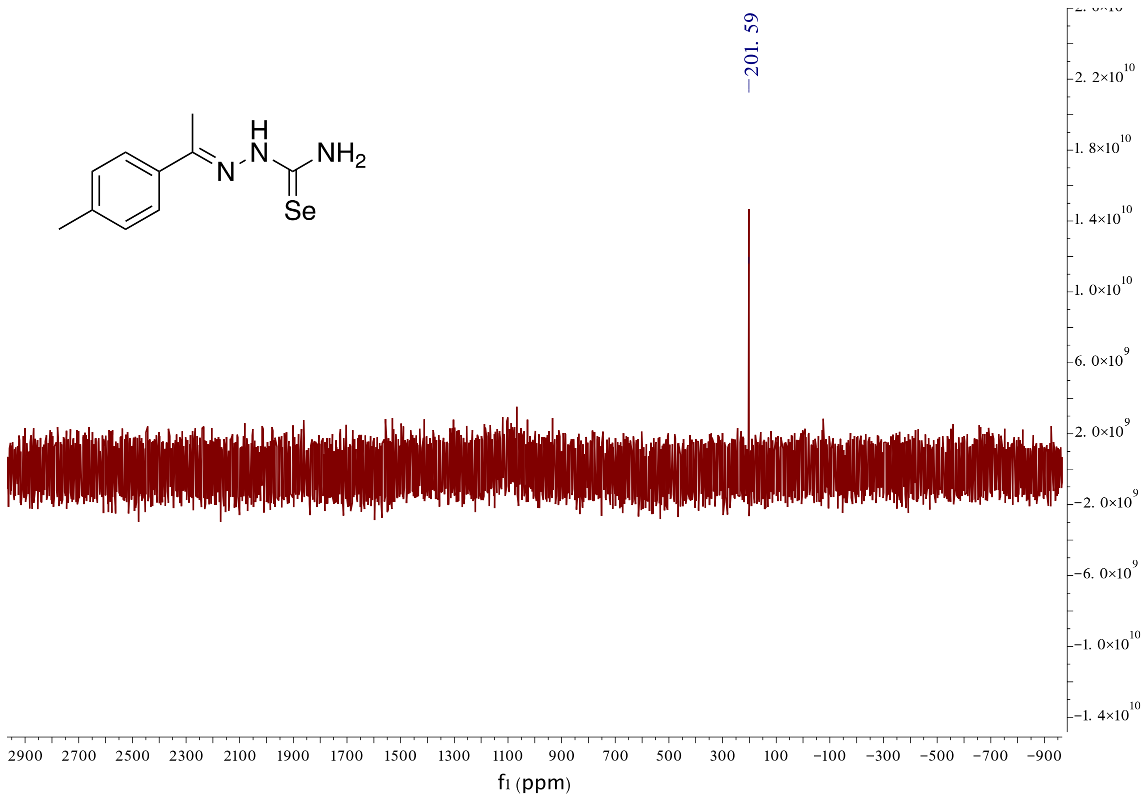

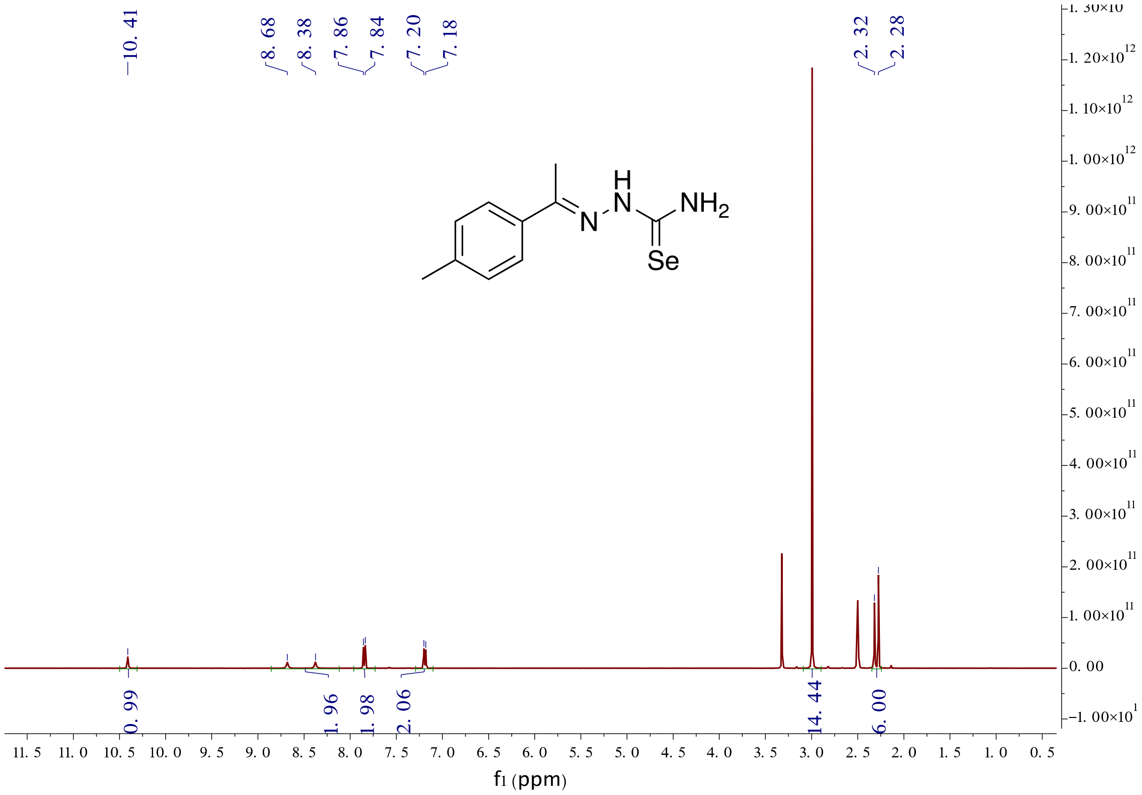

Figure S39. qNMR of compound ***Se*1a** (up) and IR of compound ***Se*1b** (down).

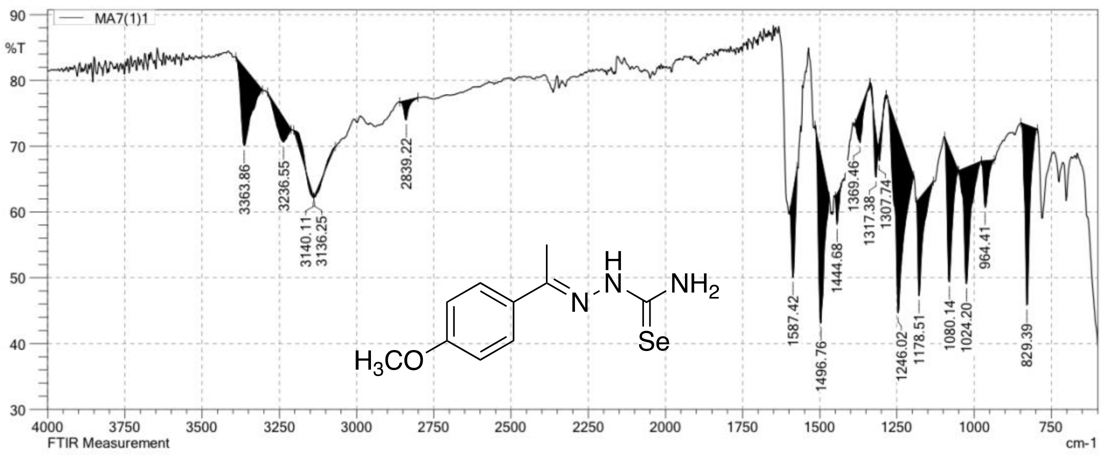

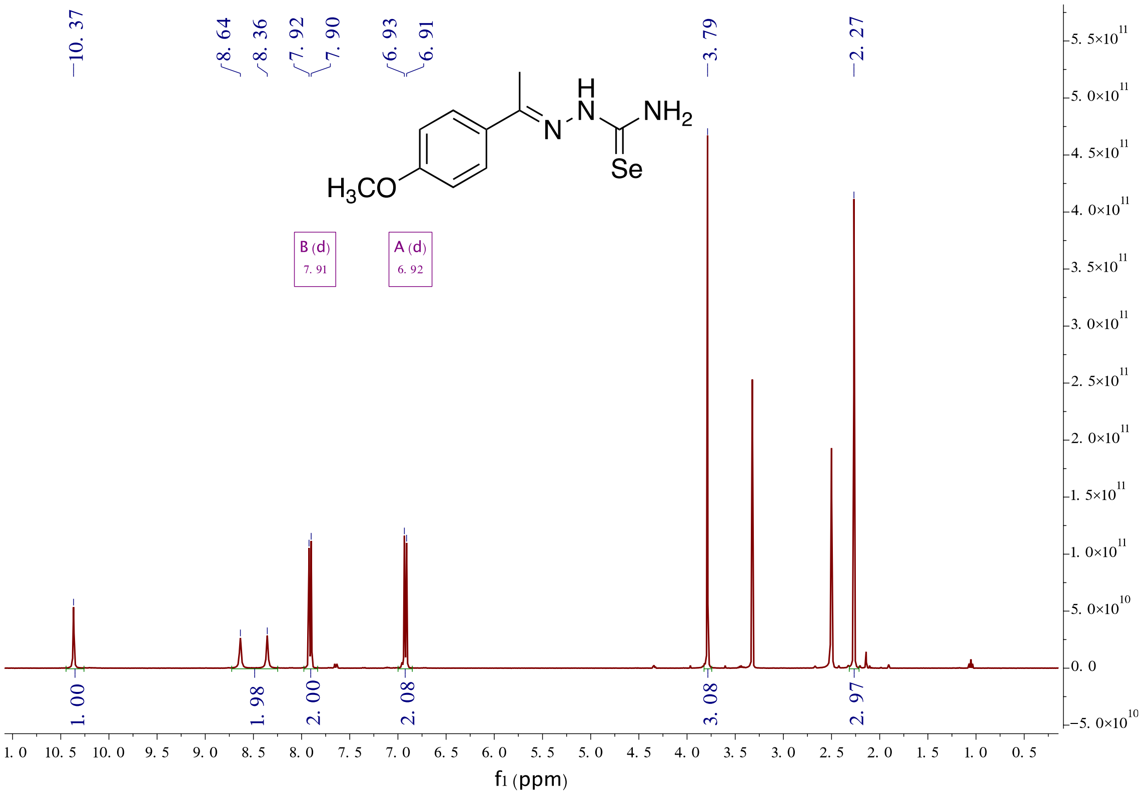

Figure S40. ^{1}H-NMR (up) and ^{13}C-NMR (down) of compound ***Se*1b**.

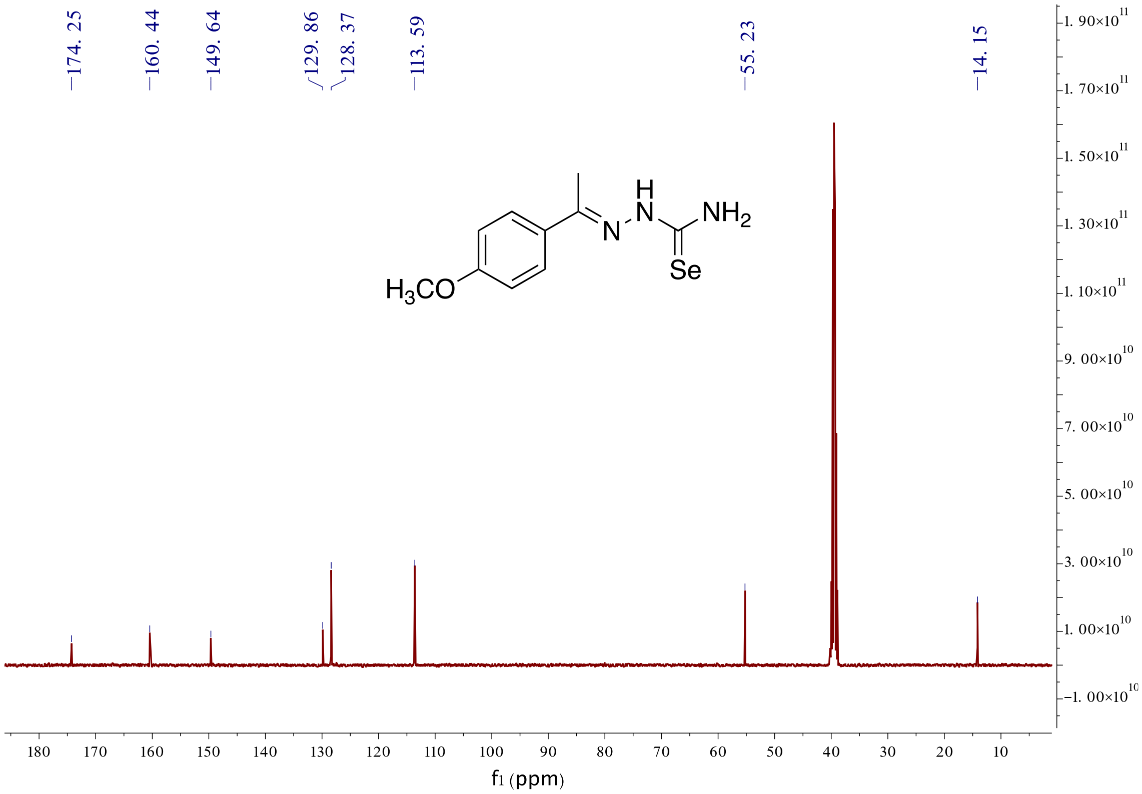

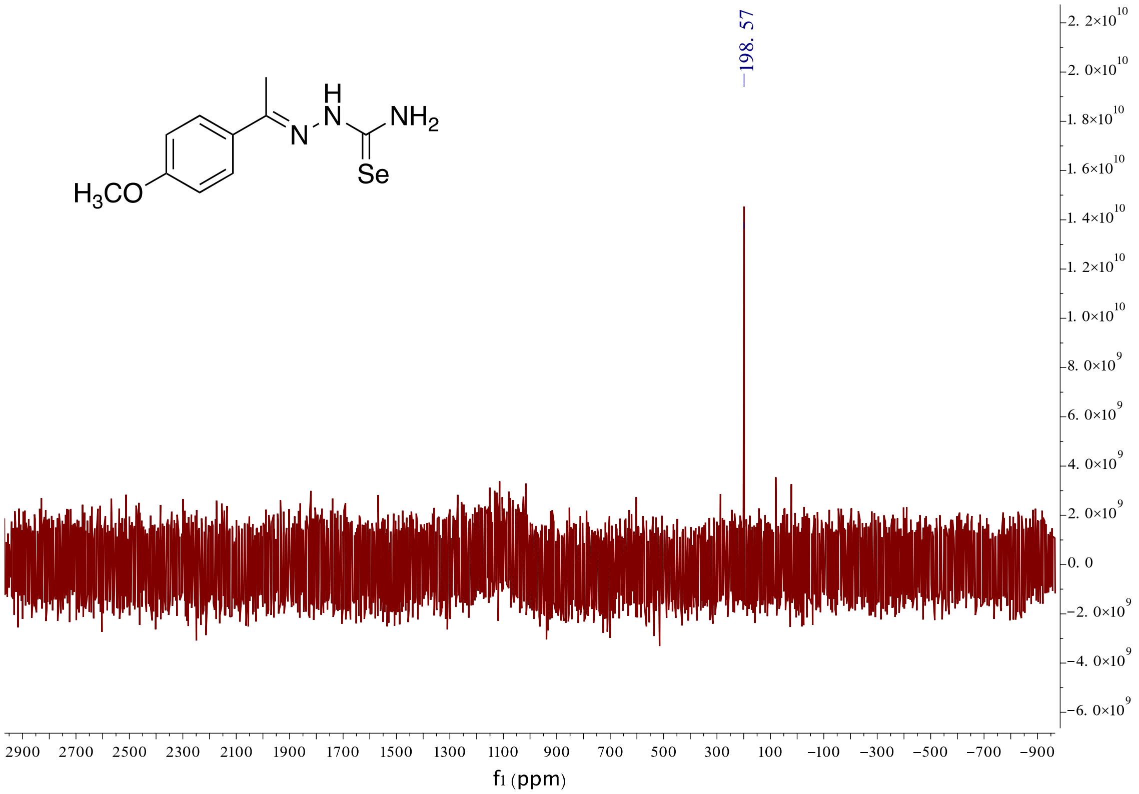

Figure S41. ^{77}Se-NMR (up) and qNMR (down) of compound ***Se*1b**.

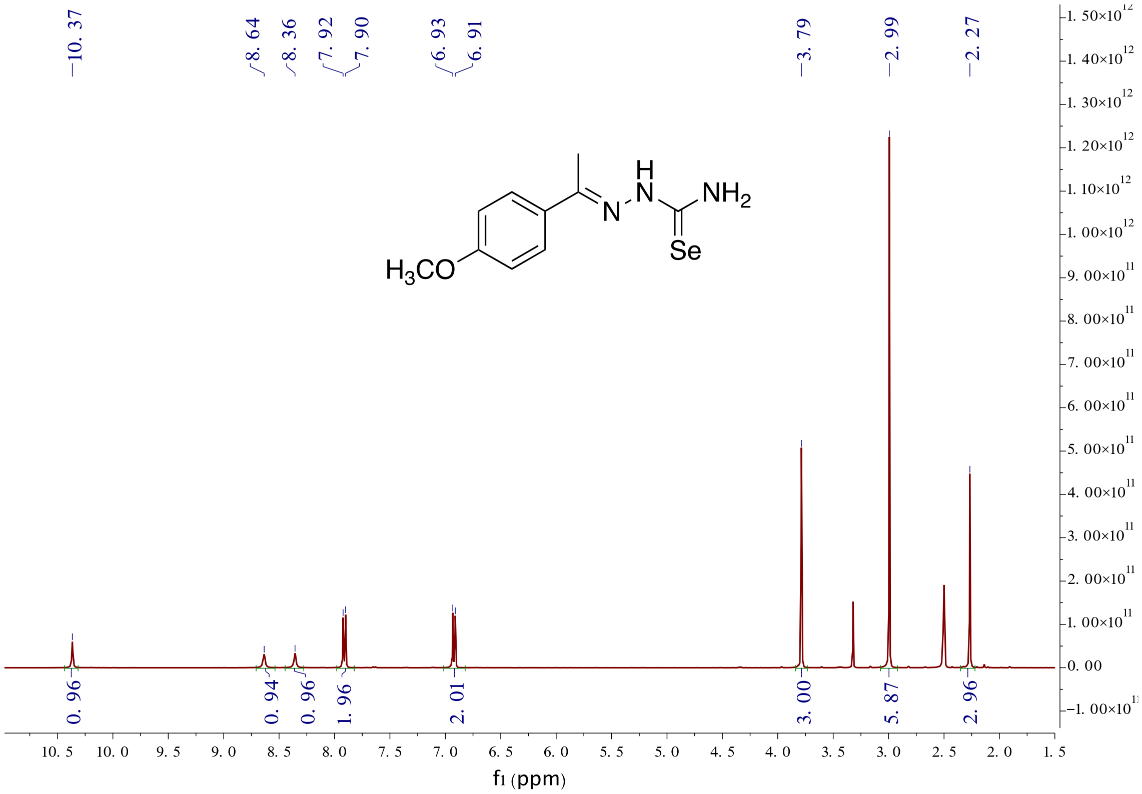

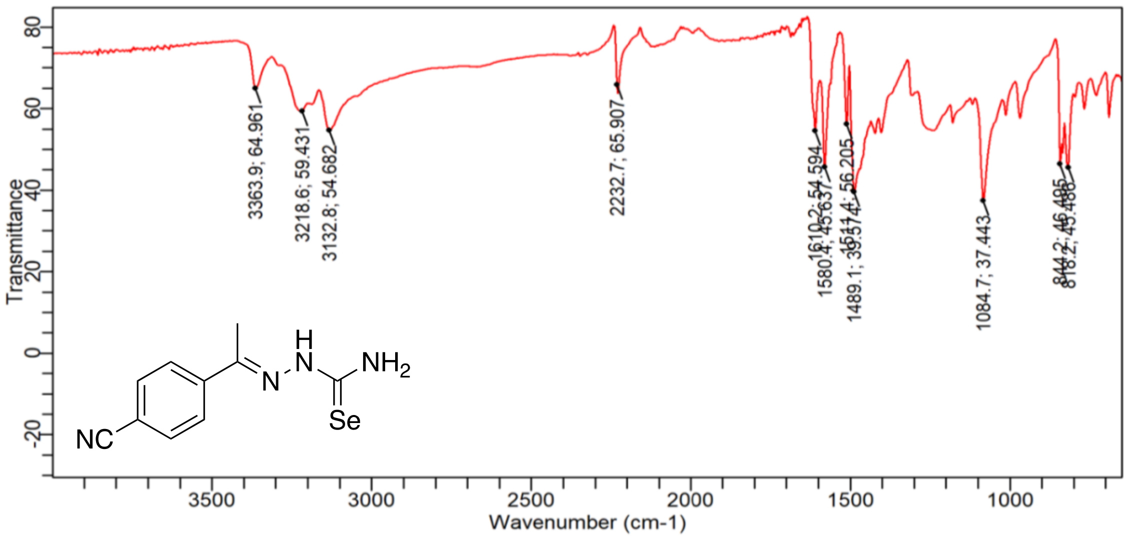

Figure S42. IR (up) and ^{13}C-NMR (down) of compound ***Se*1e**.

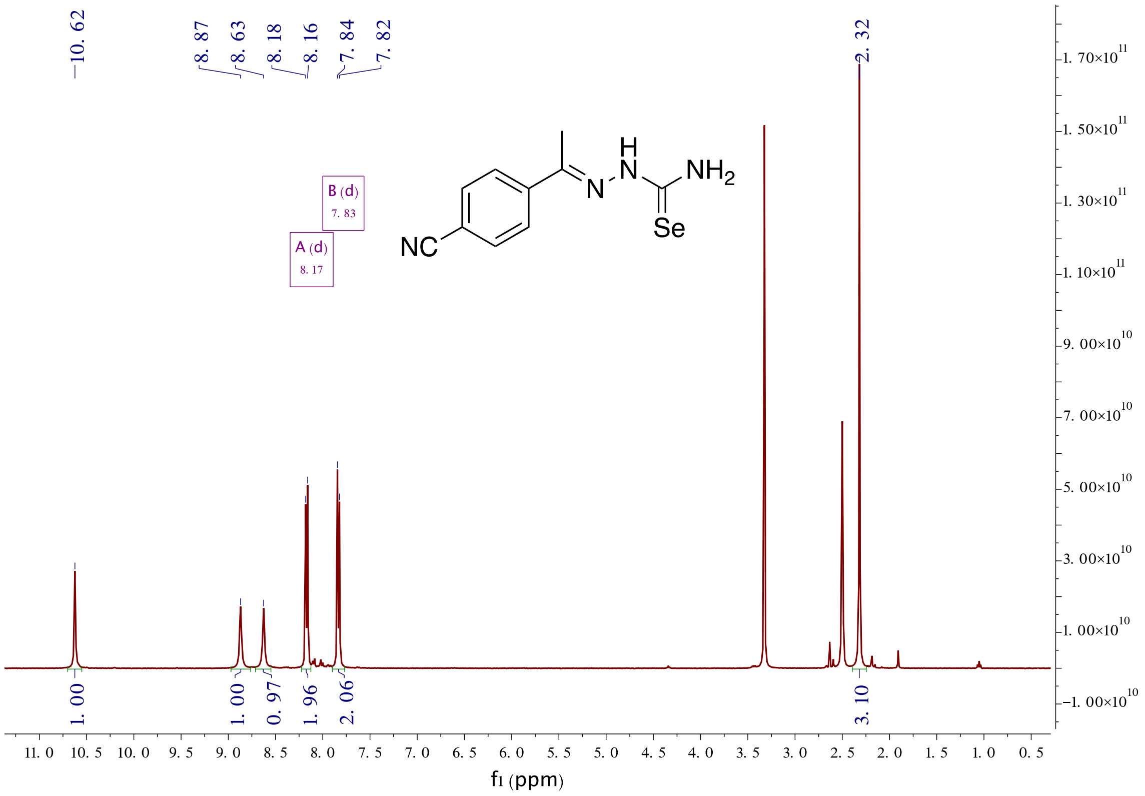

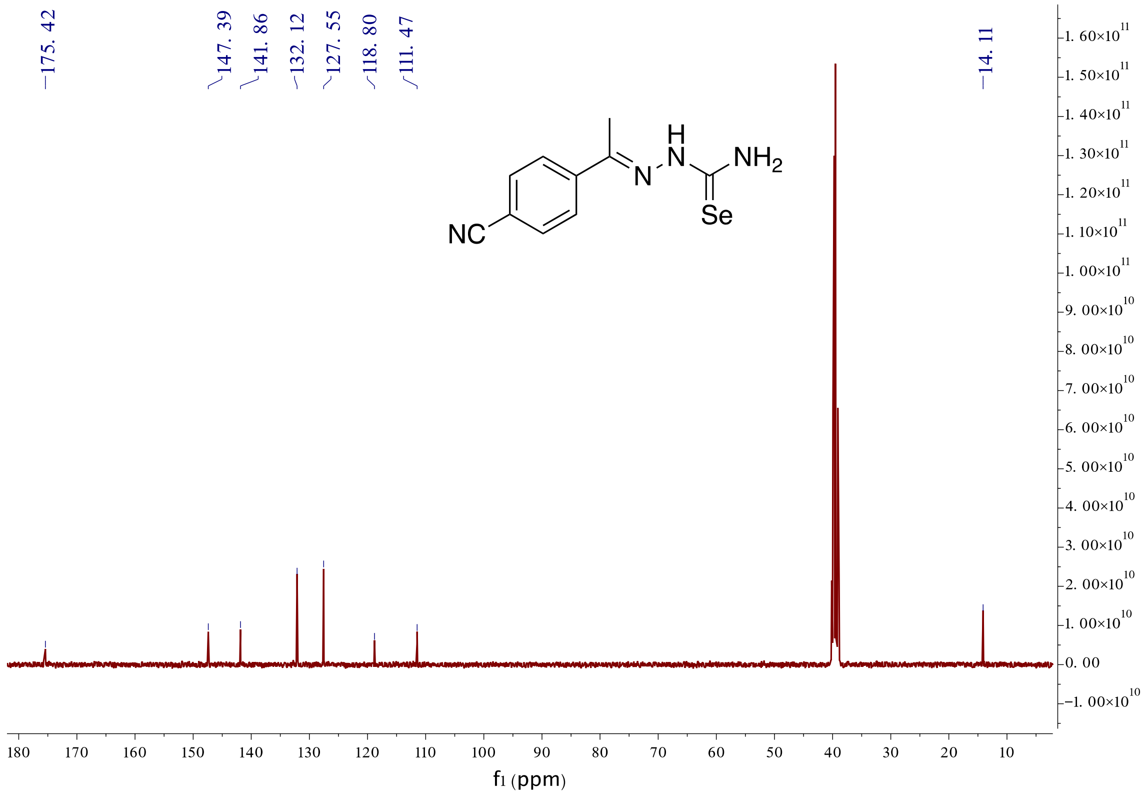

Figure S43. ^{13}C-NMR (up) and ^{77}Se-NMR (down) of compound ***Se*1e**.

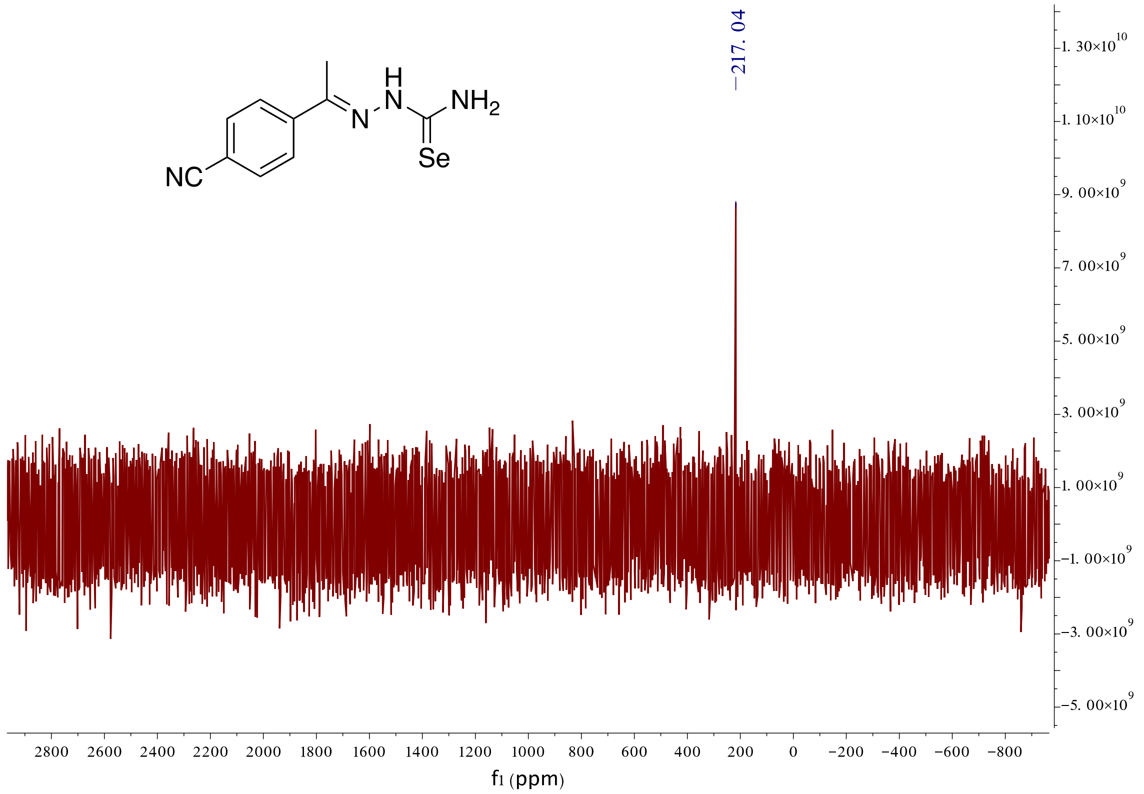

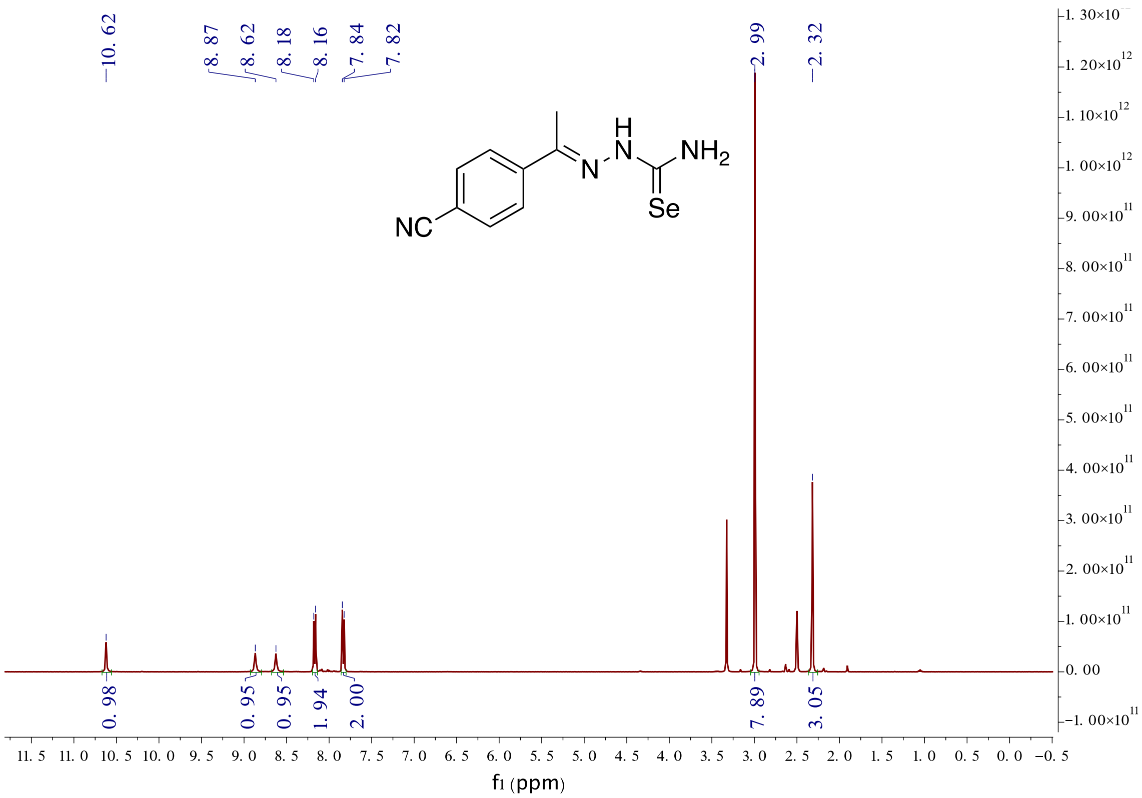

Figure S44. qNMR of compound ***Se*1e** (up) and IR of compound ***Se*1f** (down).

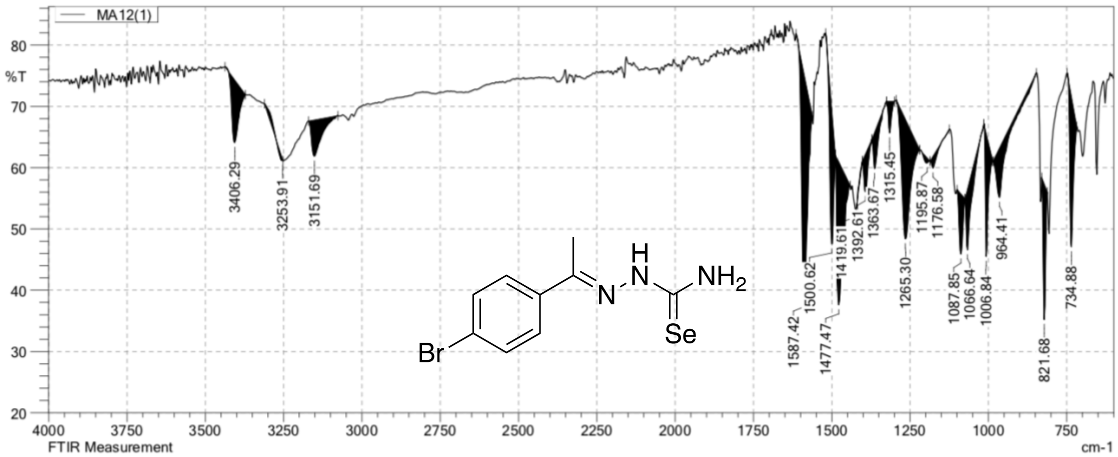

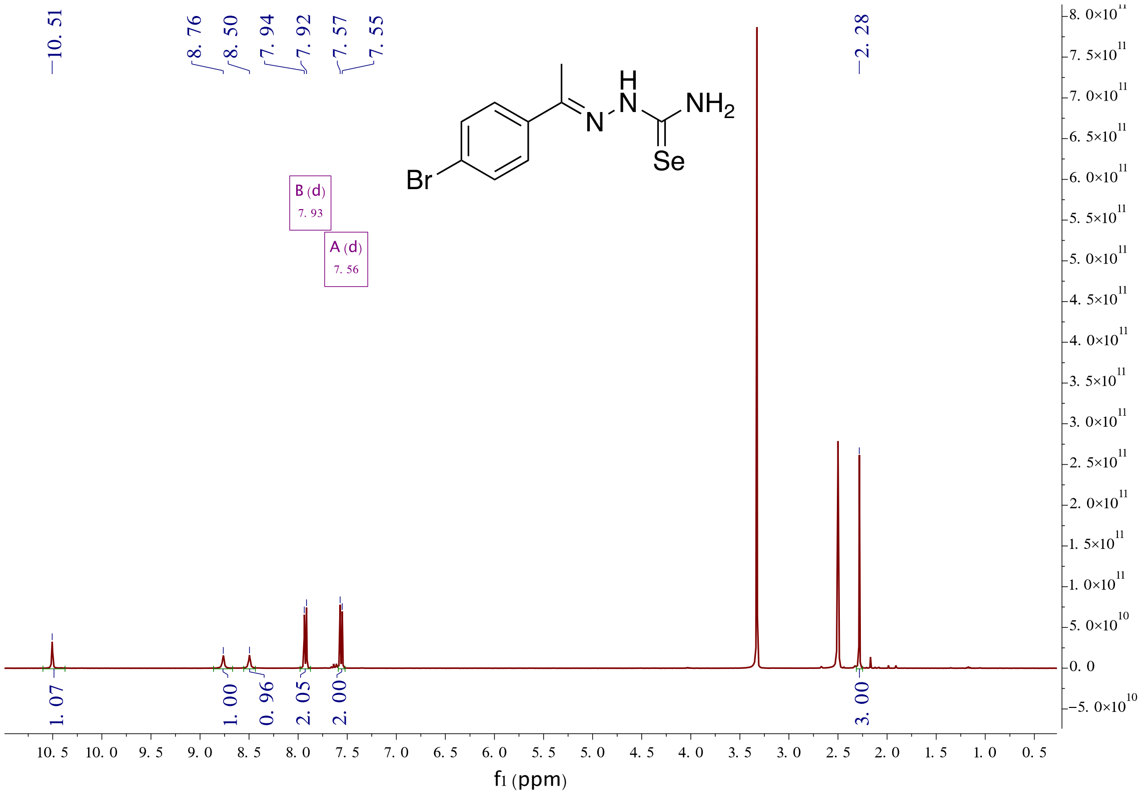

Figure S45. ^{1}H-NMR (up) and ^{13}C-NMR (down) of compound ***Se*1f**.

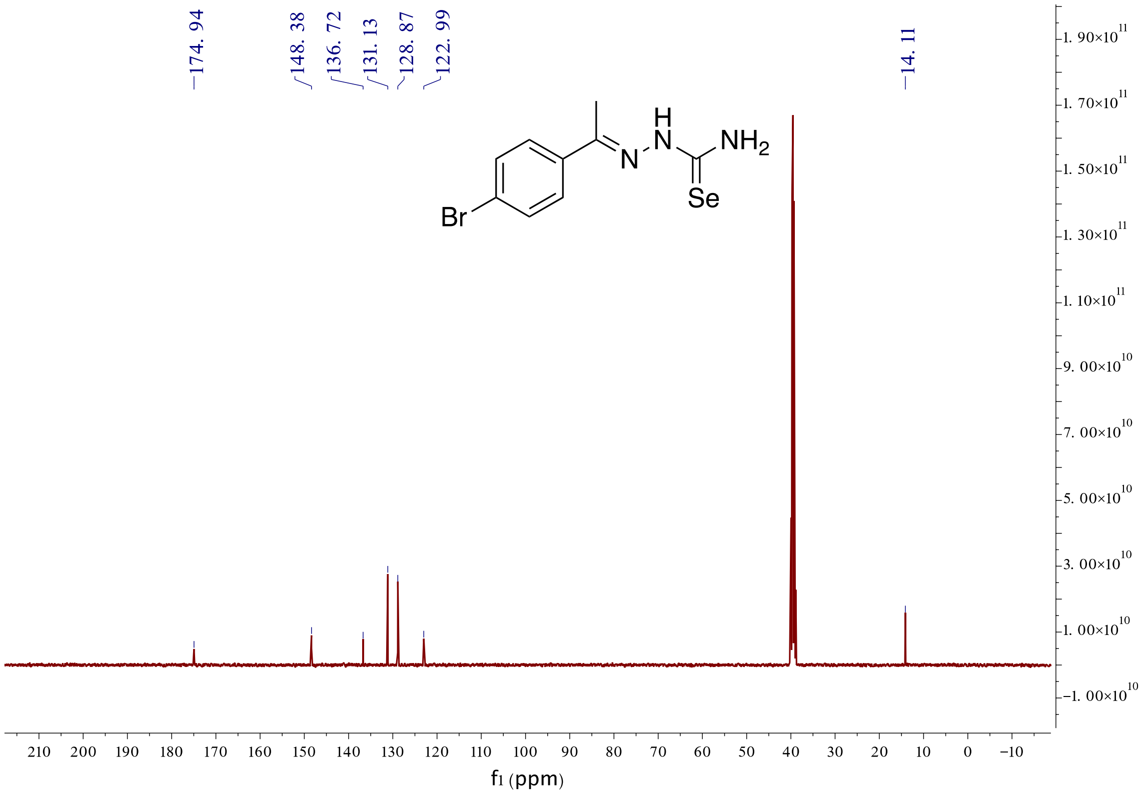

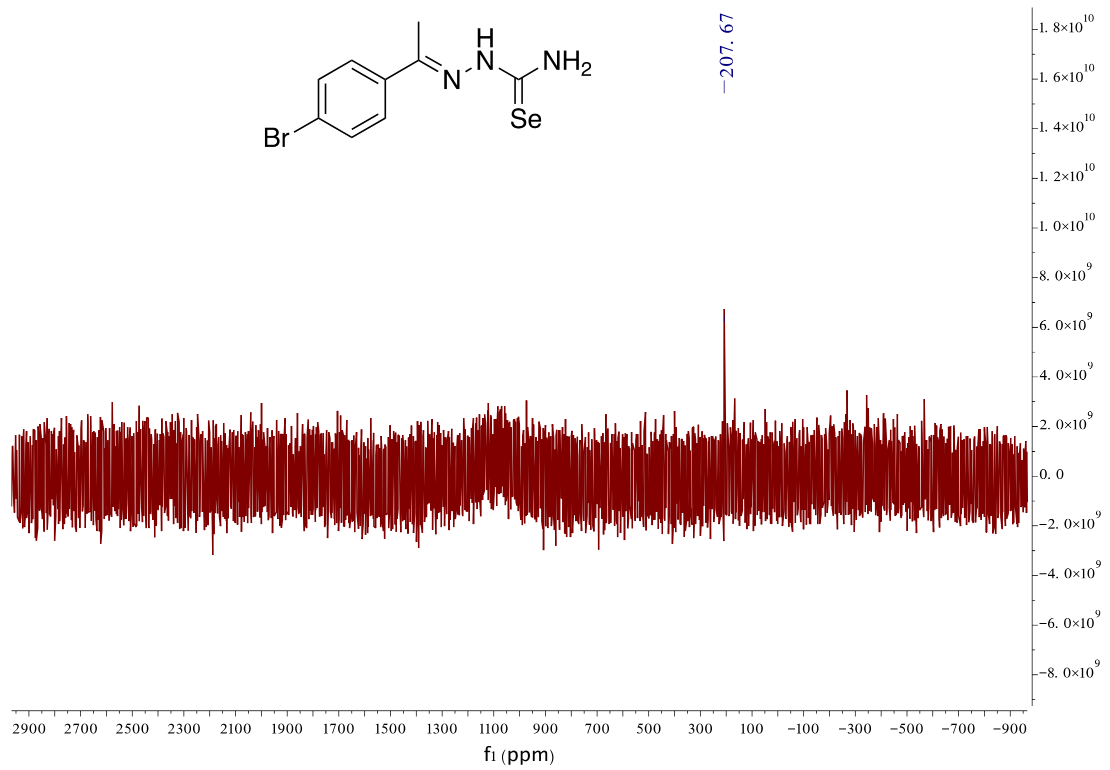

Figure S46. ^{77}Se-NMR (up) and qNMR (down) of compound ***Se*1f**.

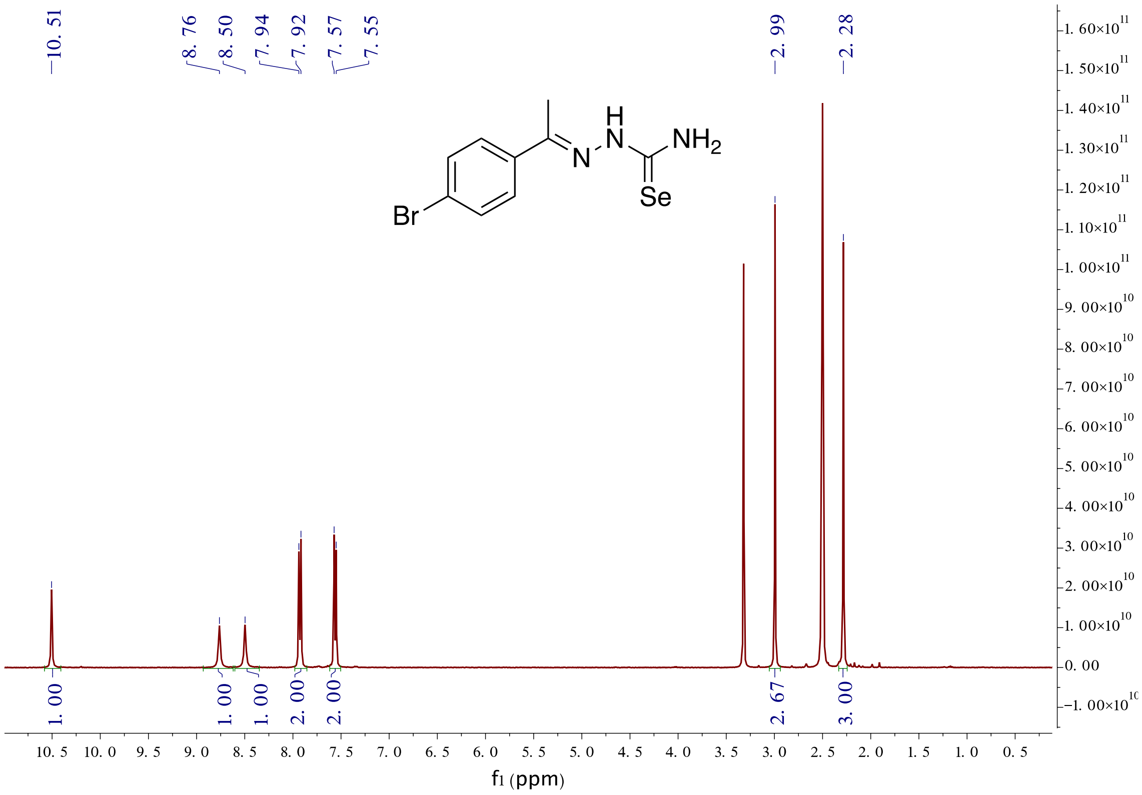

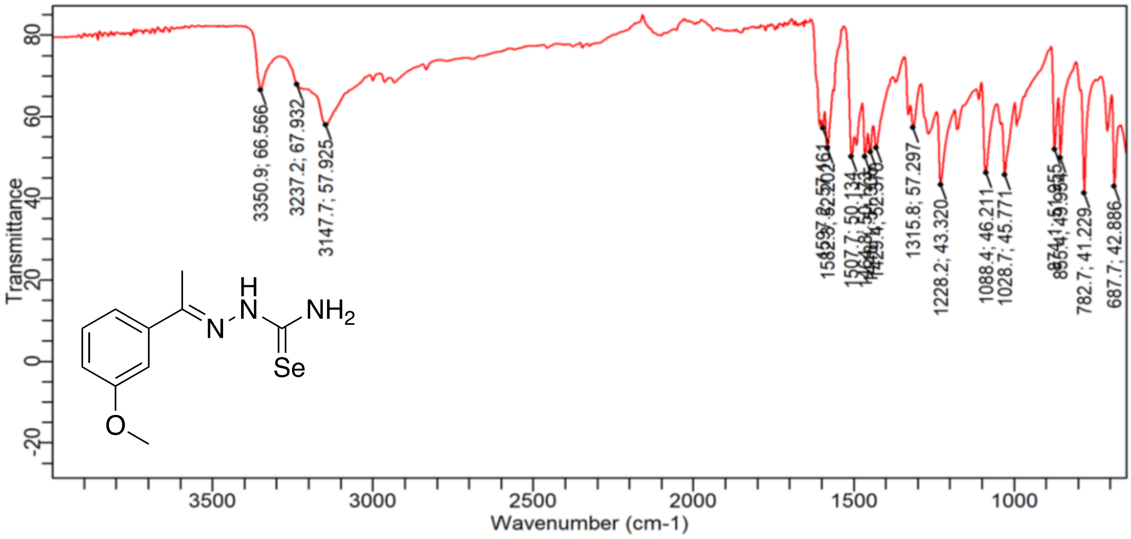

Figure S47. IR (up) and ^{1}H-NMR (down) of compound ***Se*1g**.

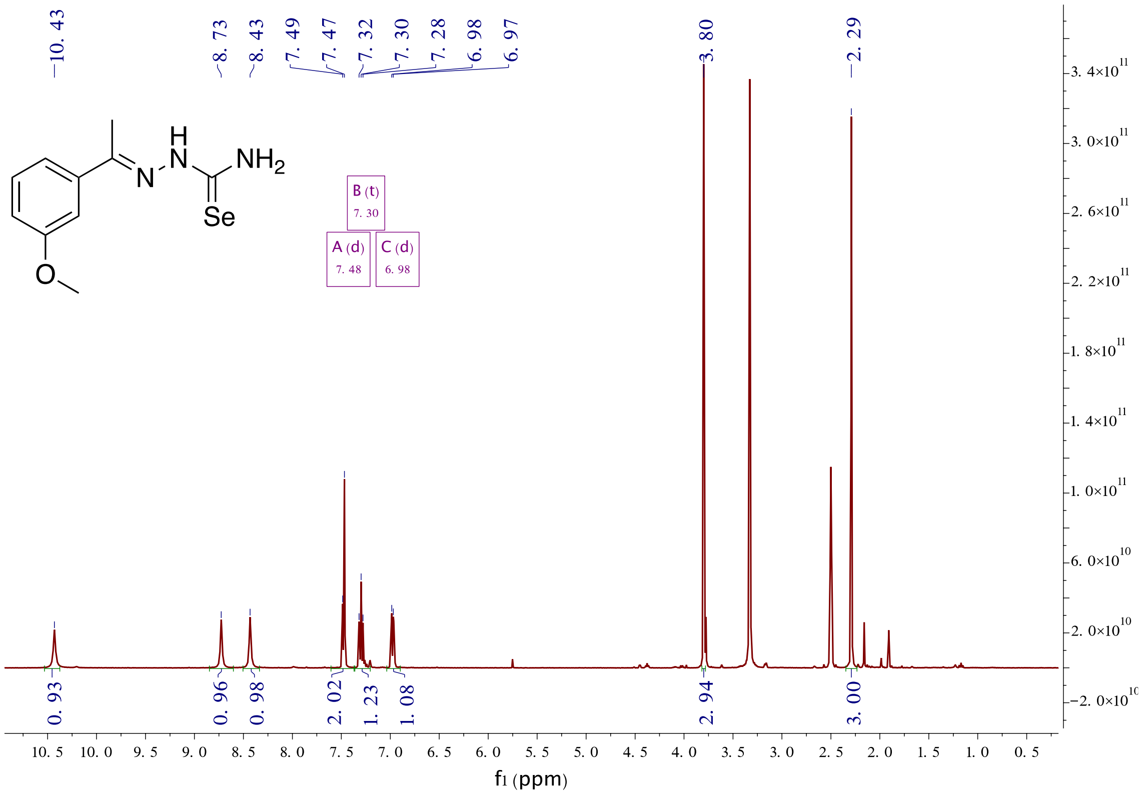

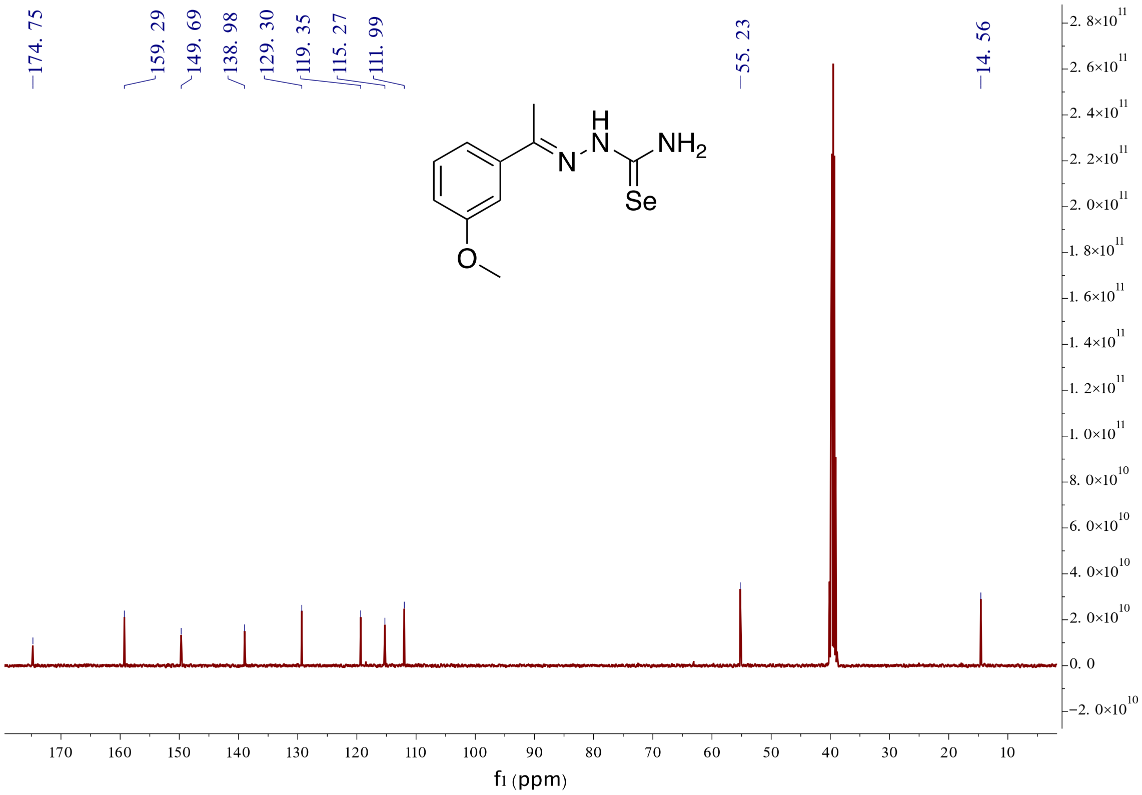

Figure S48. ^{13}C-NMR (up) and ^{77}Se-NMR (down) of compound ***Se*1g**.

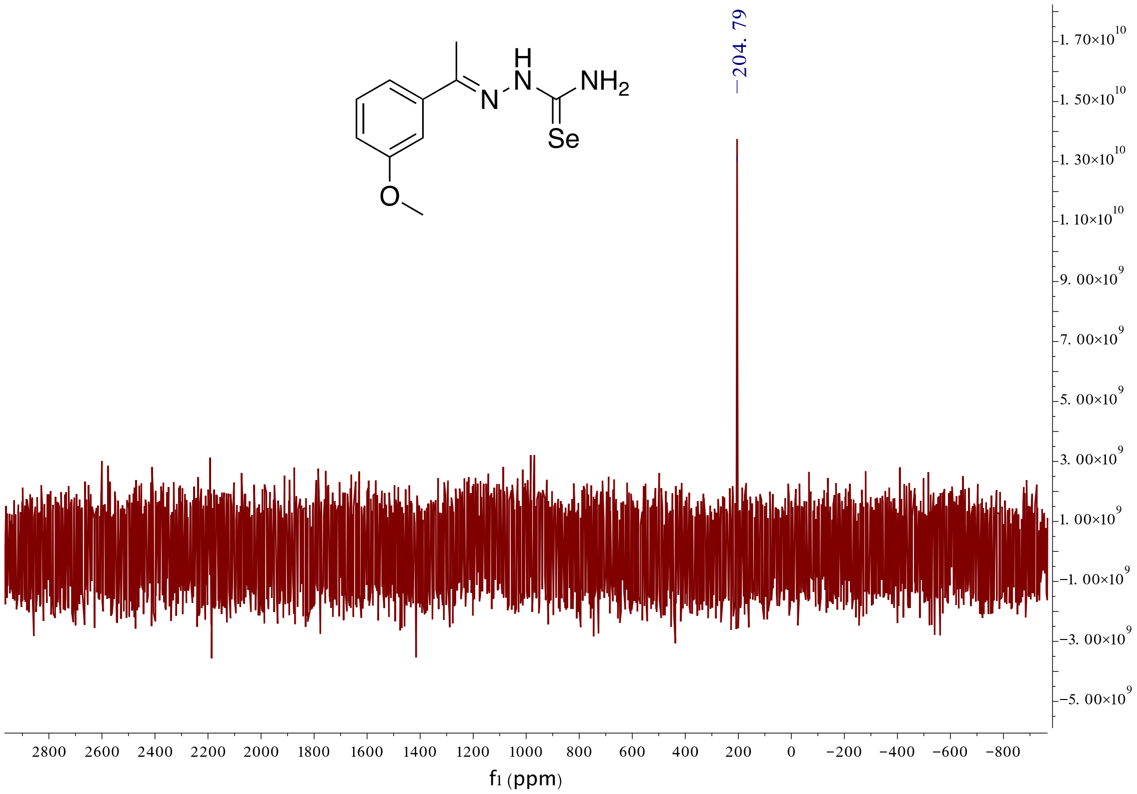

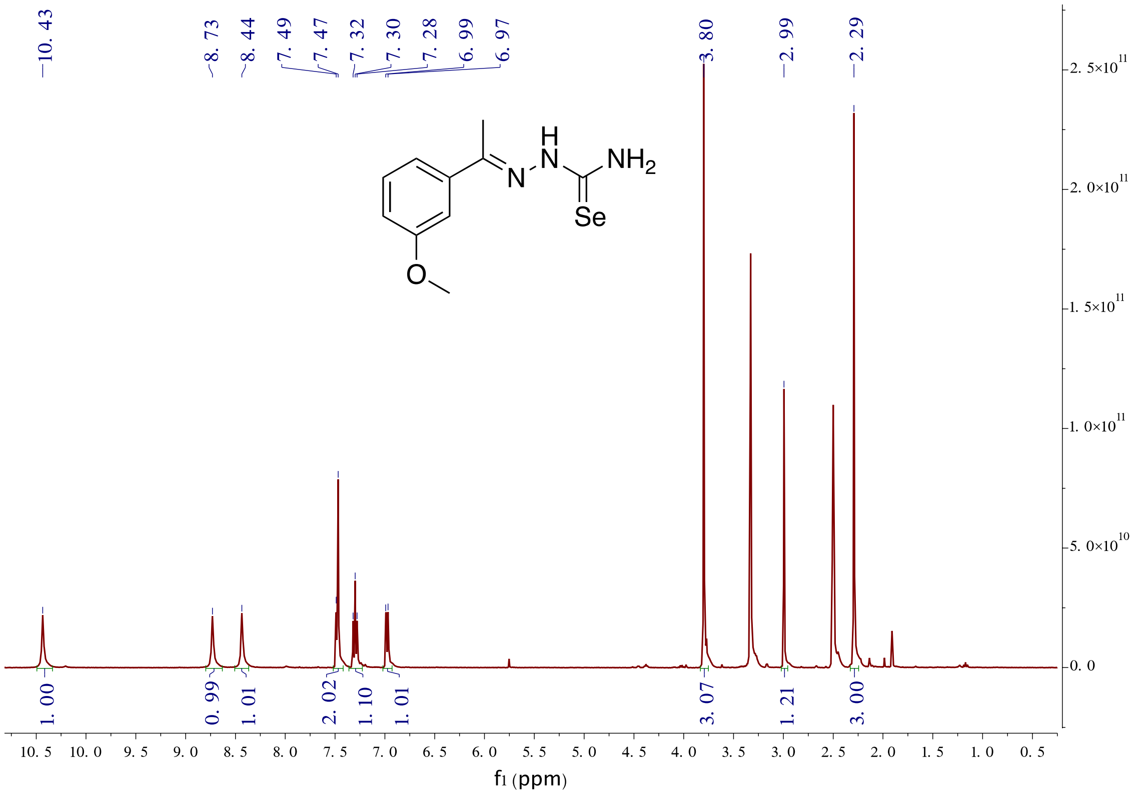

Figure S49. qNMR of compound ***Se*1g** (up) and IR of compound ***Se*1h** (down).

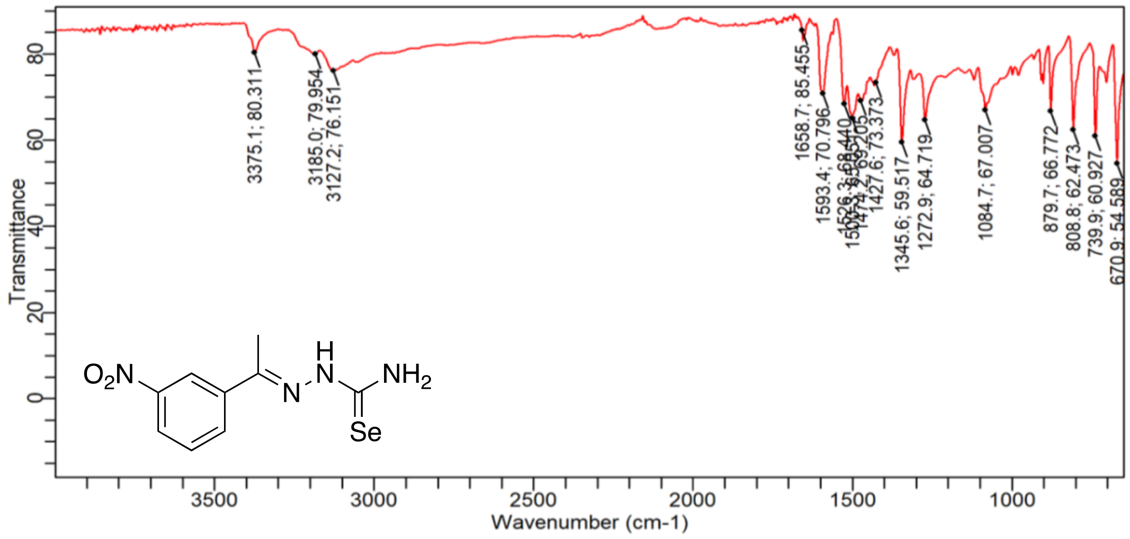

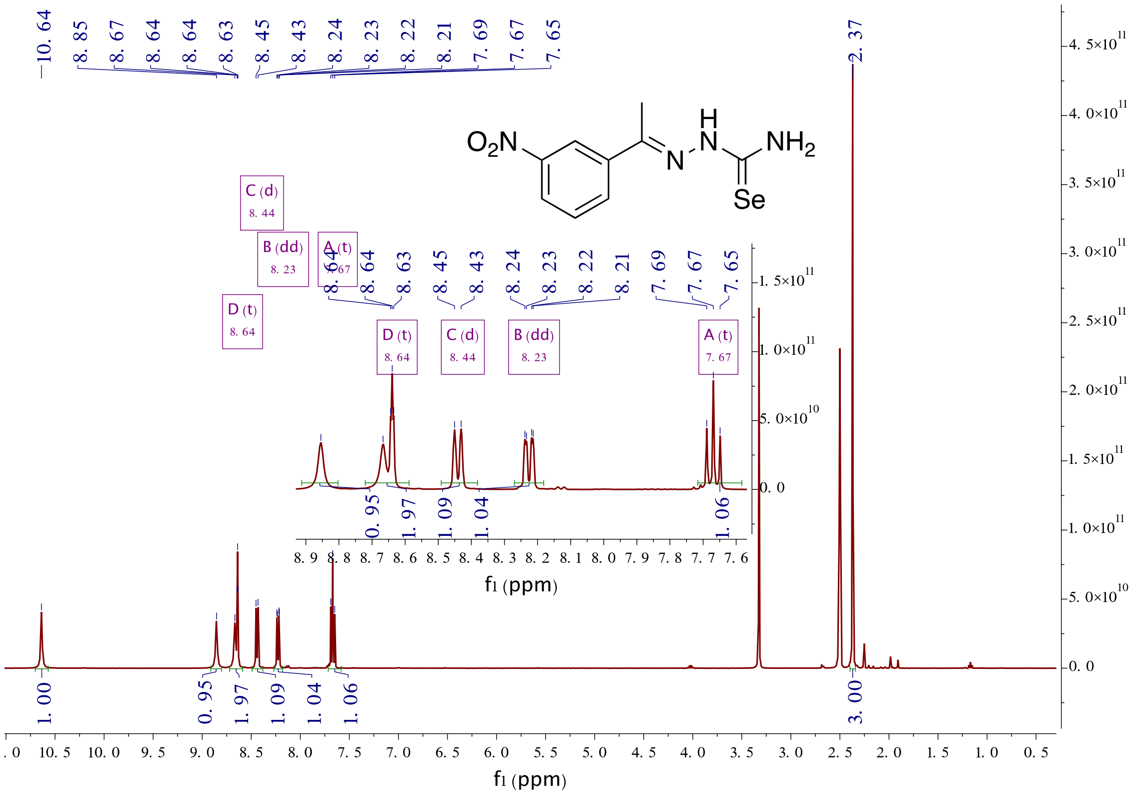

Figure S50. ^{1}H-NMR (up) and ^{13}C-NMR (down) of compound ***Se*1h**.

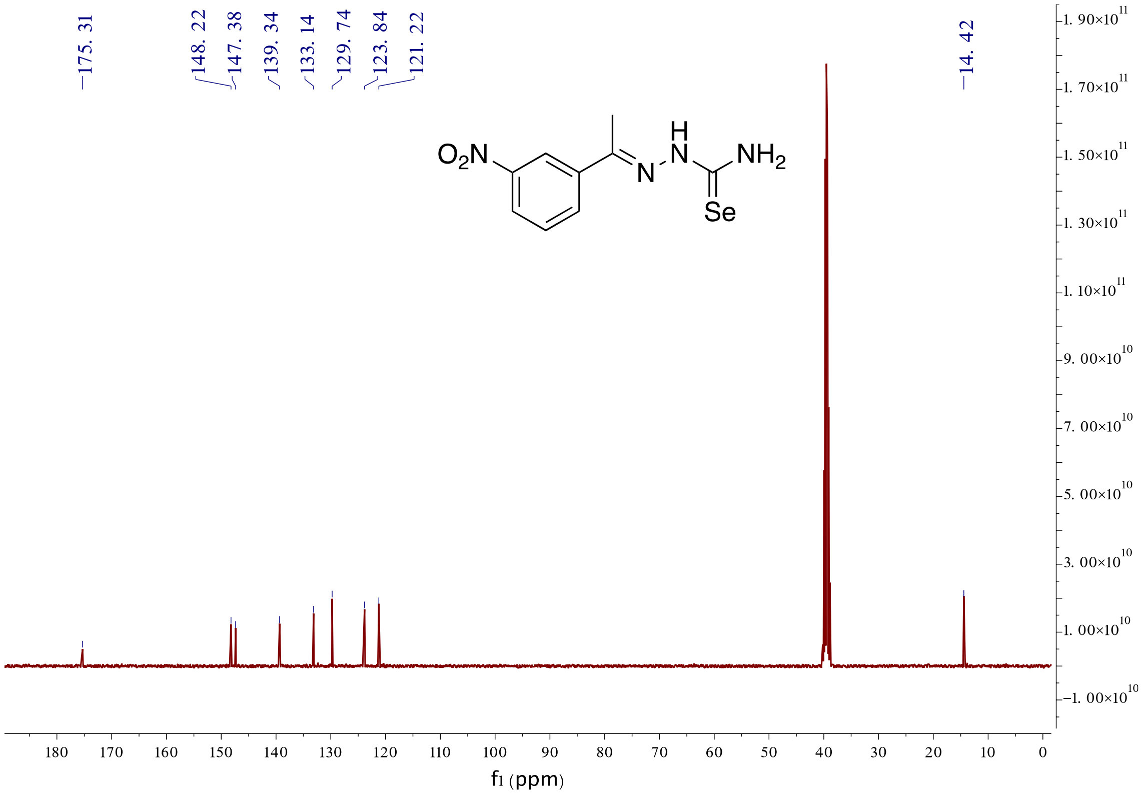

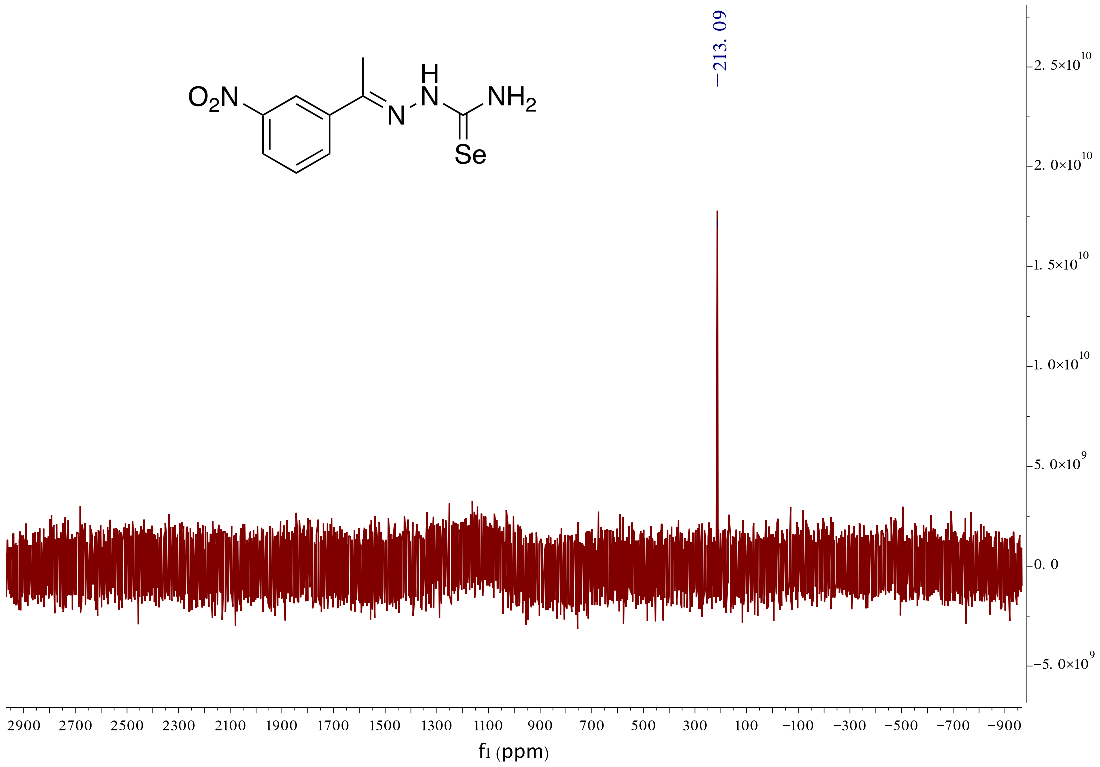

Figure S51. ^{77}Se-NMR (up) and qNMR (down) of compound ***Se*1h**.

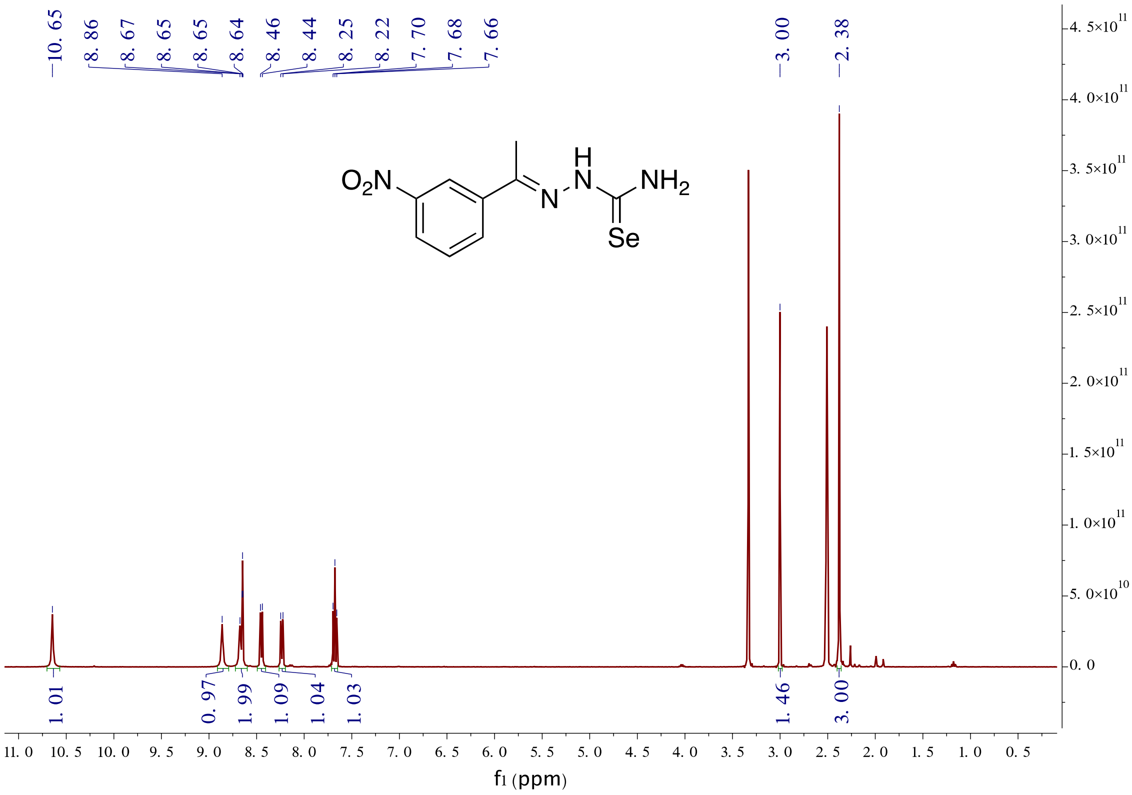

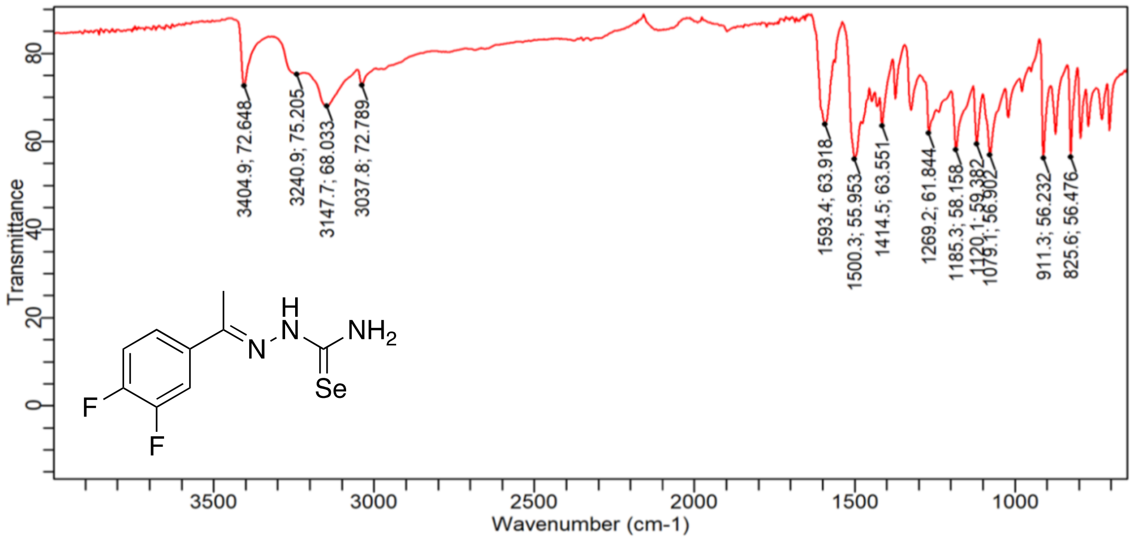

Figure S52. IR (up) and ^{1}H-NMR (down) of compound ***Se*1j**.

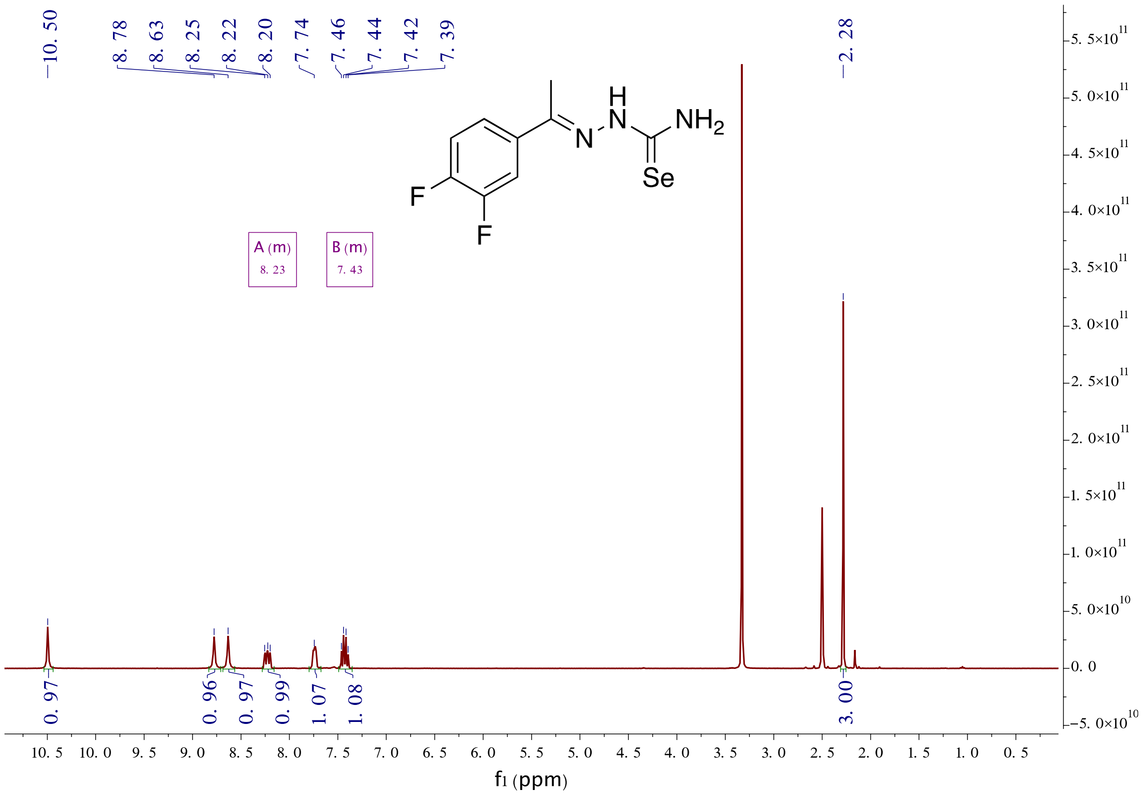

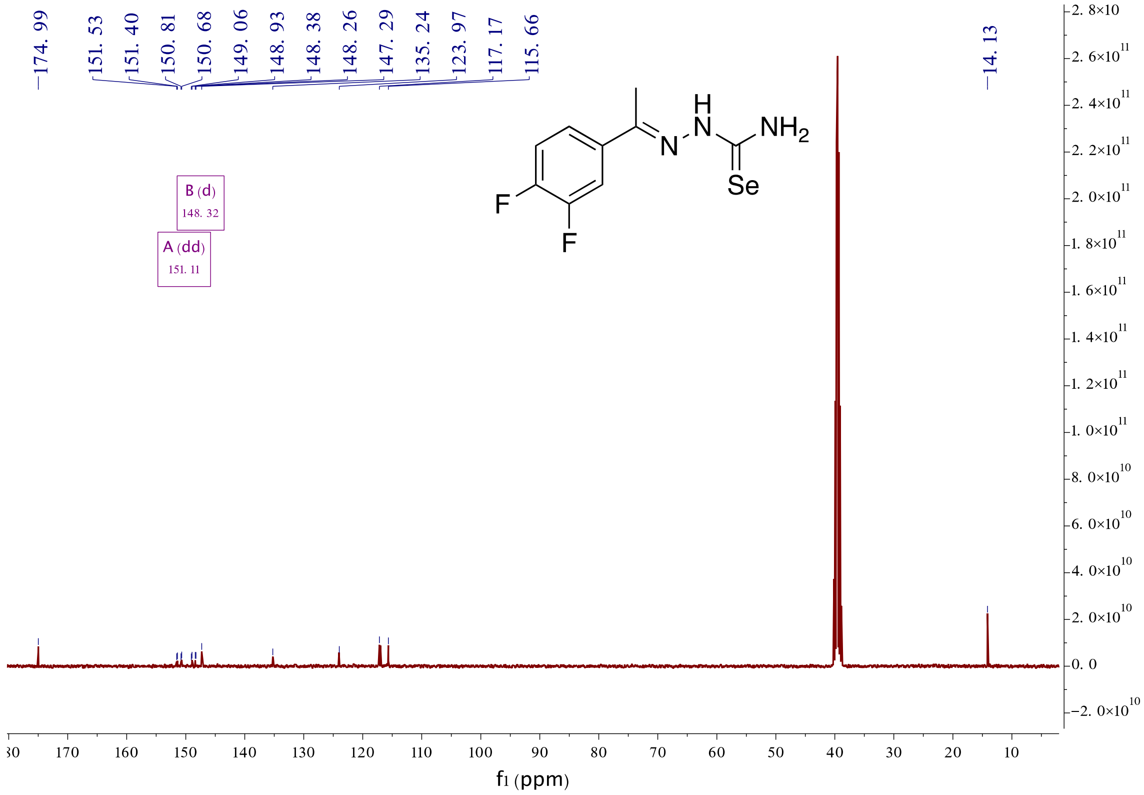

Figure S53. ^{1}H-NMR (up) and ^{77}Se-NMR (down) of compound ***Se*1j**.

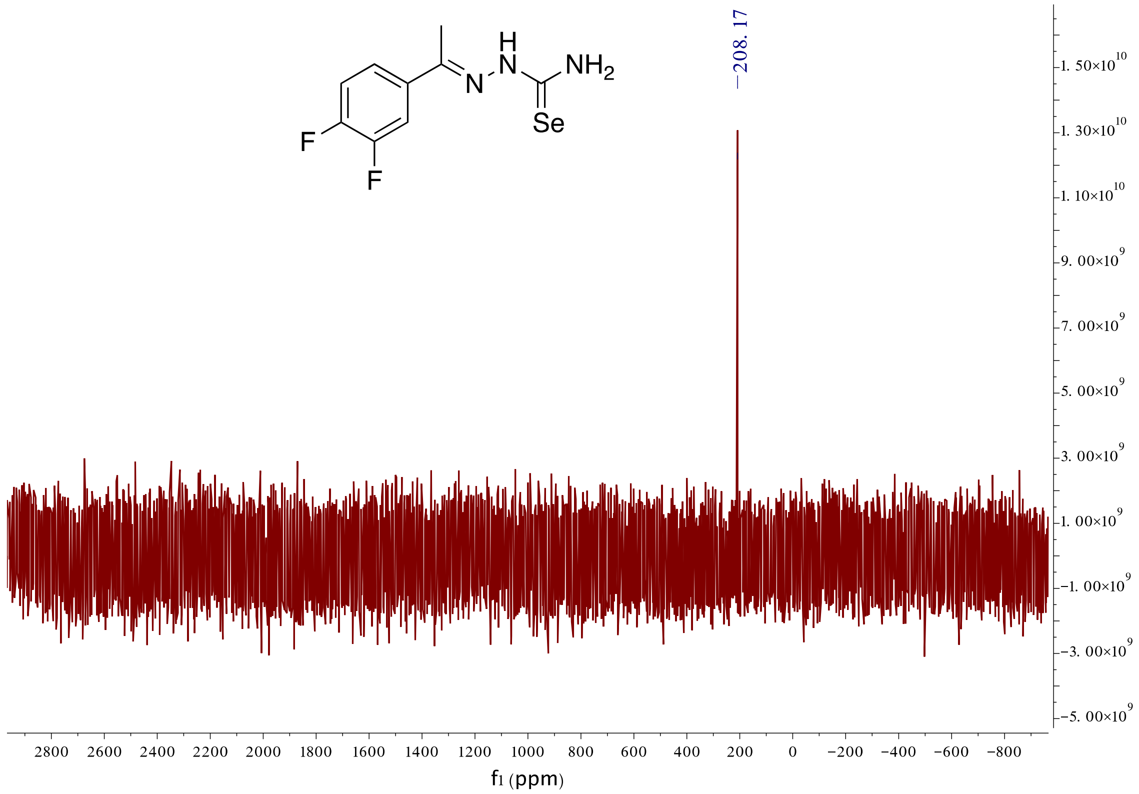

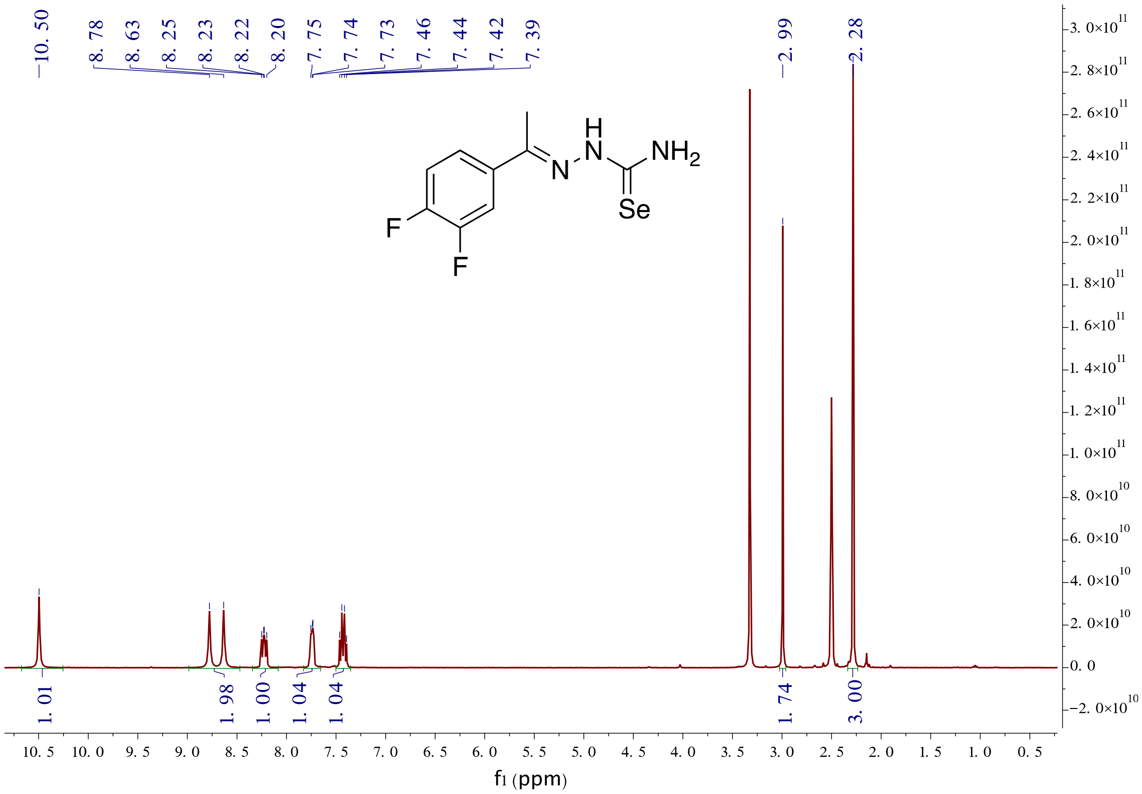

Figure S54. qNMR of compound ***Se*1j** (up) and IR of compound ***Se*1k** (down).

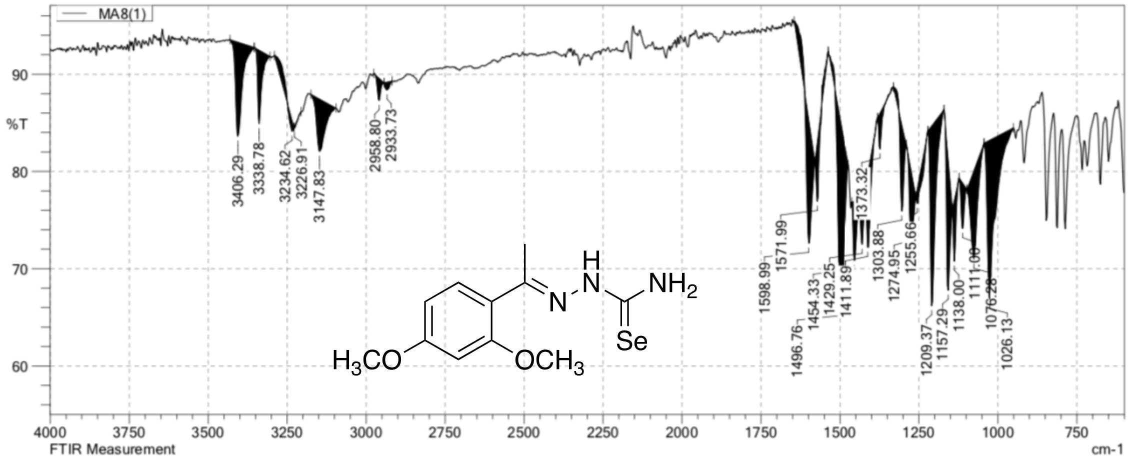

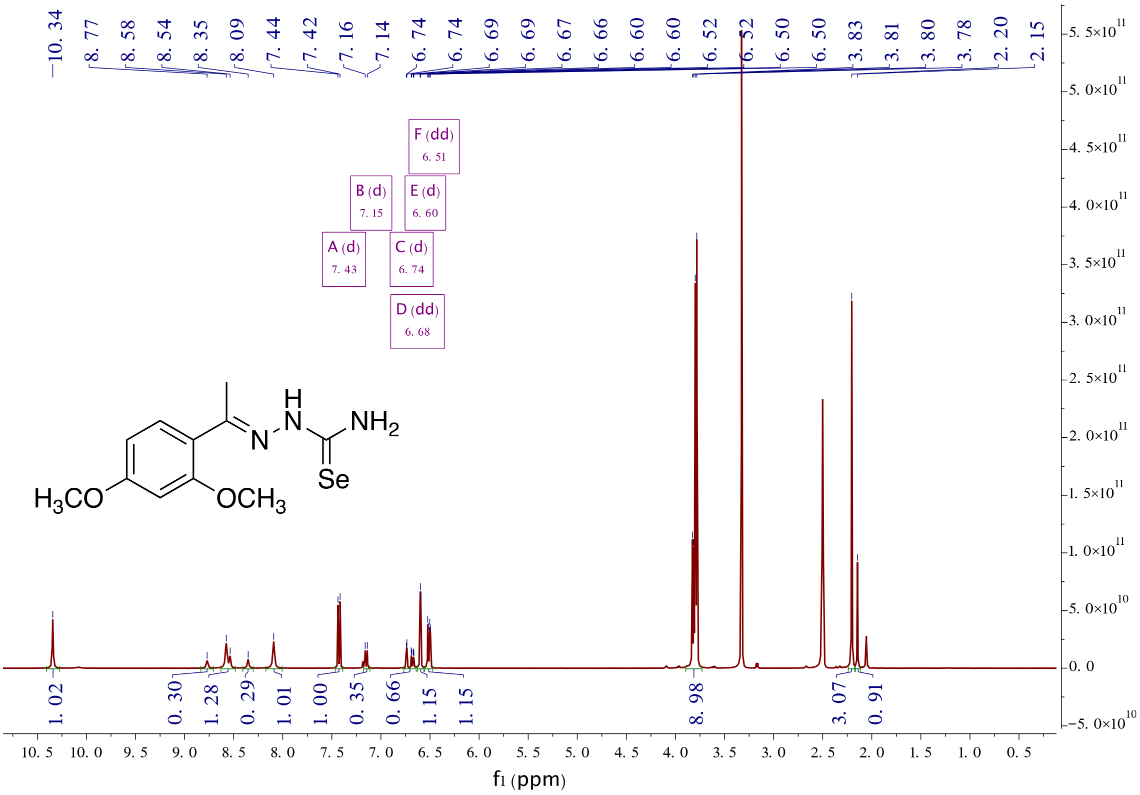

Figure S55. ^{1}H-NMR (up) and ^{13}C-NMR (down) of compound ***Se*1k**.

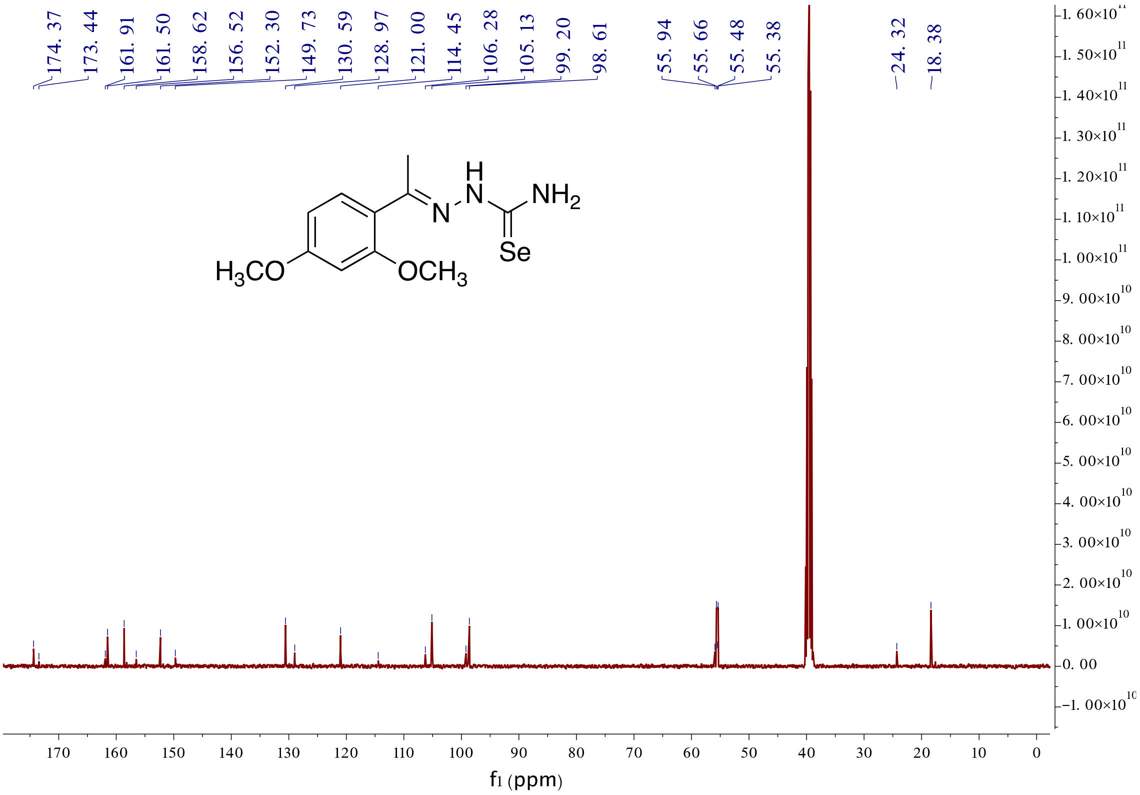

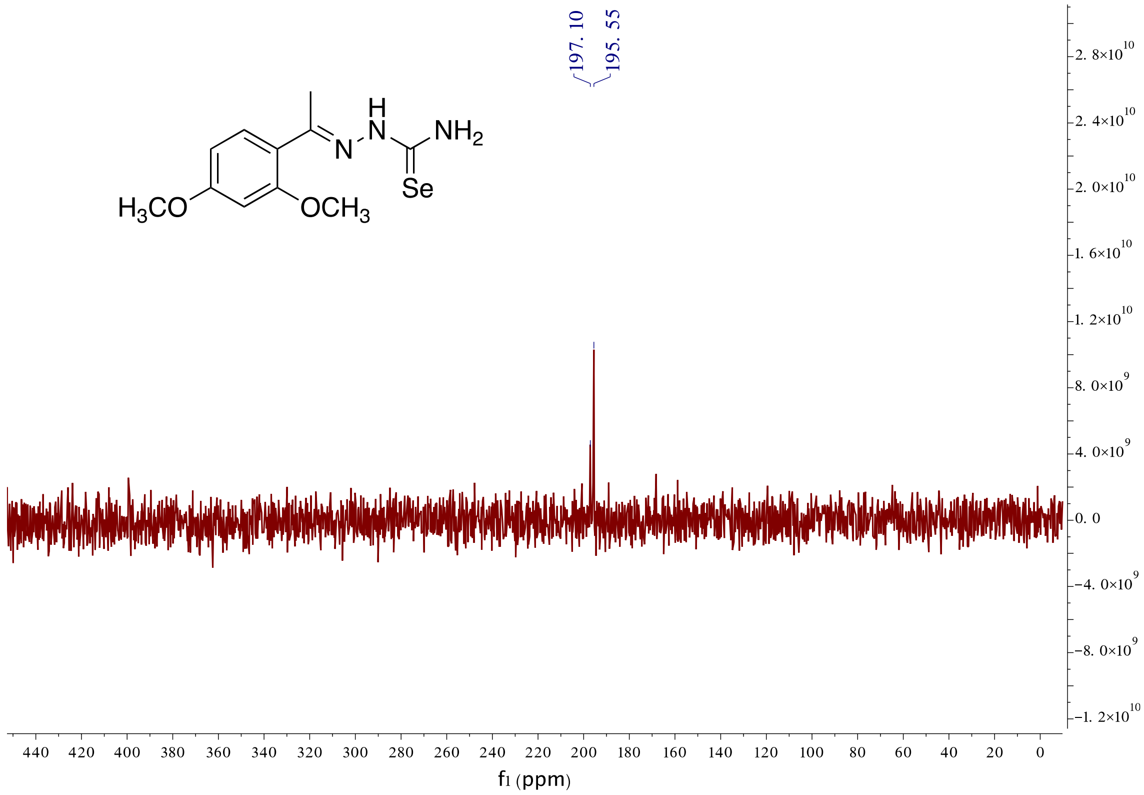

Figure S56. ^{77}Se-NMR (up) and qNMR (down) of compound ***Se*1k**.

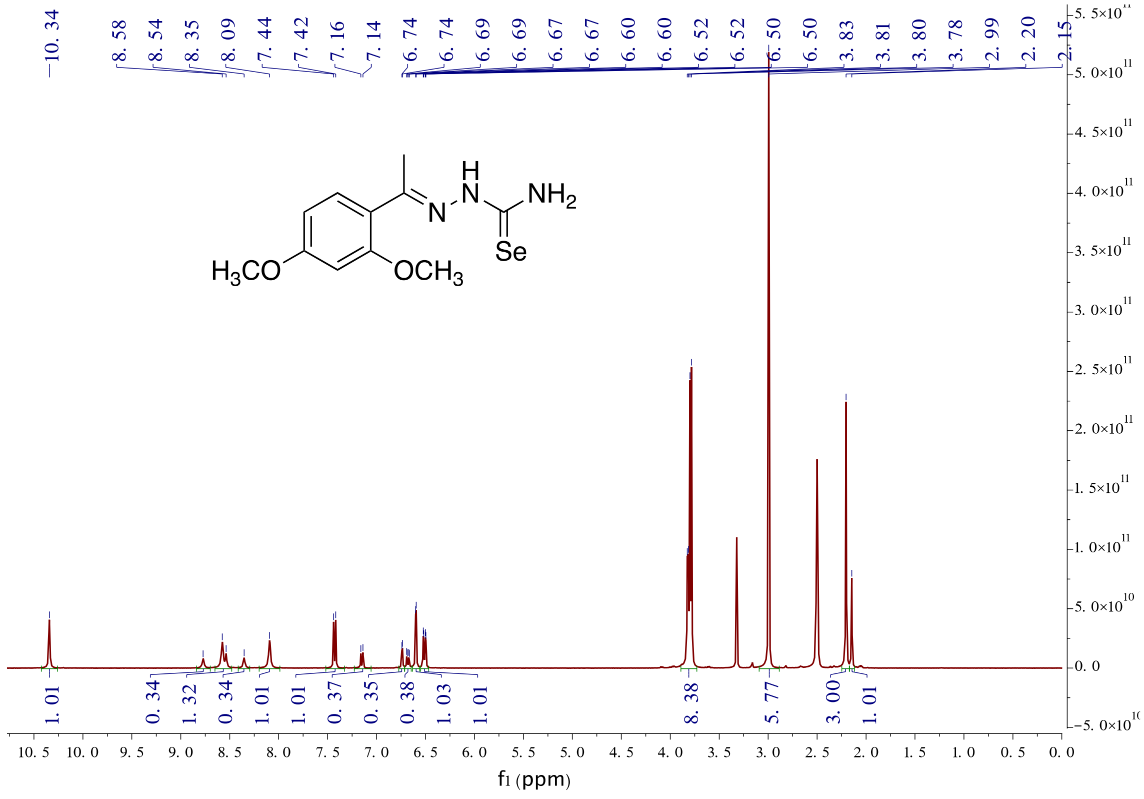

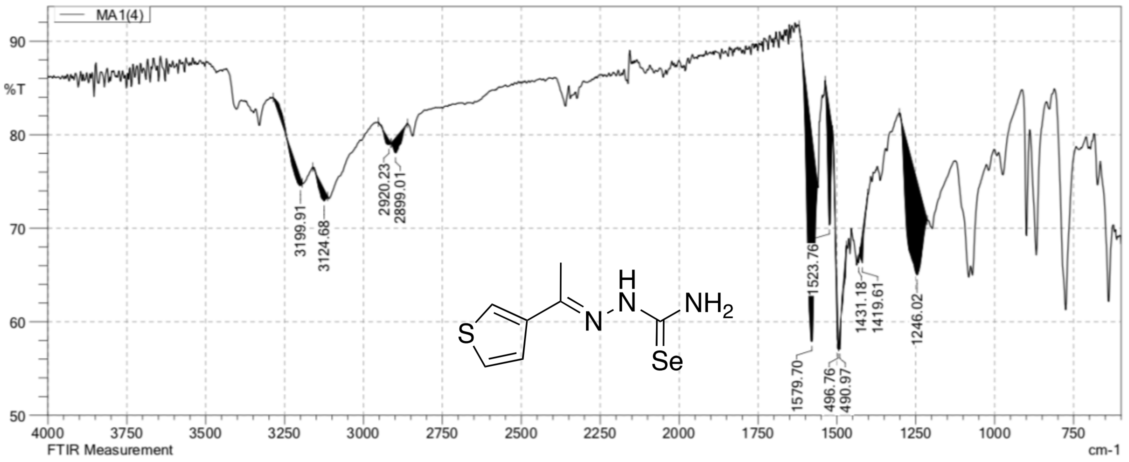

Figure S57. IR (up) and ^{1}H-NMR (down) of compound ***Se*1l**.

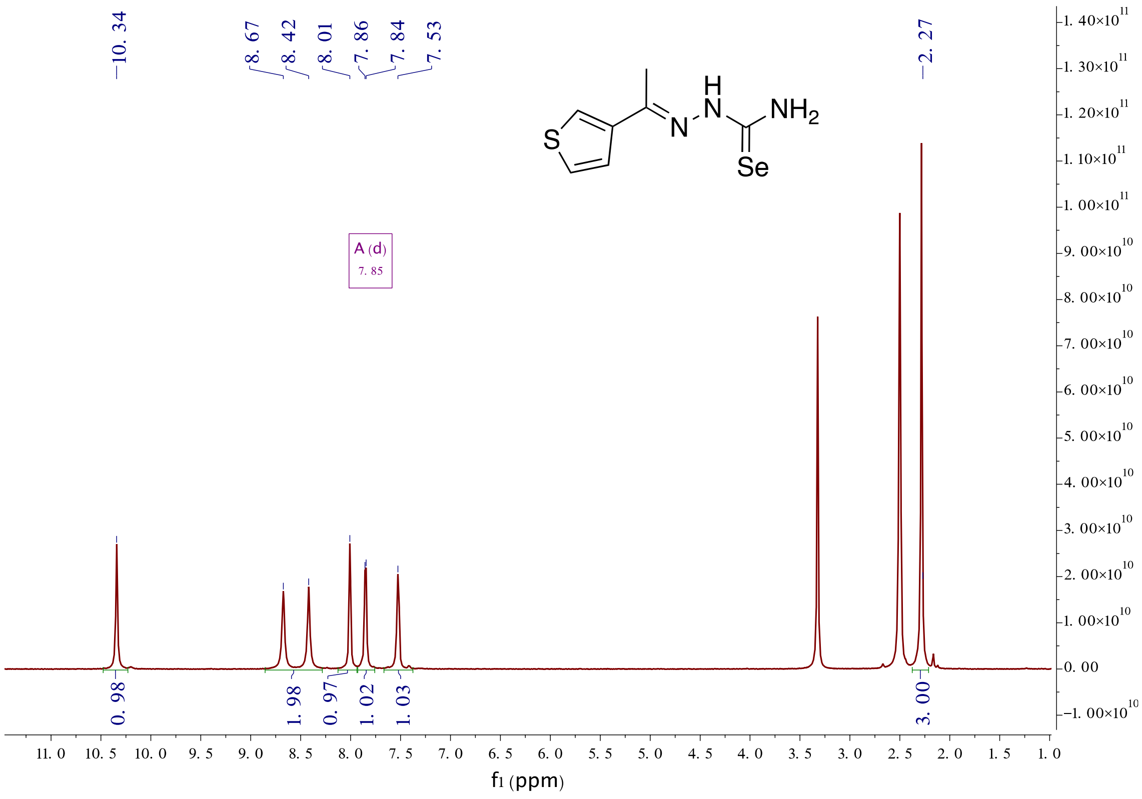

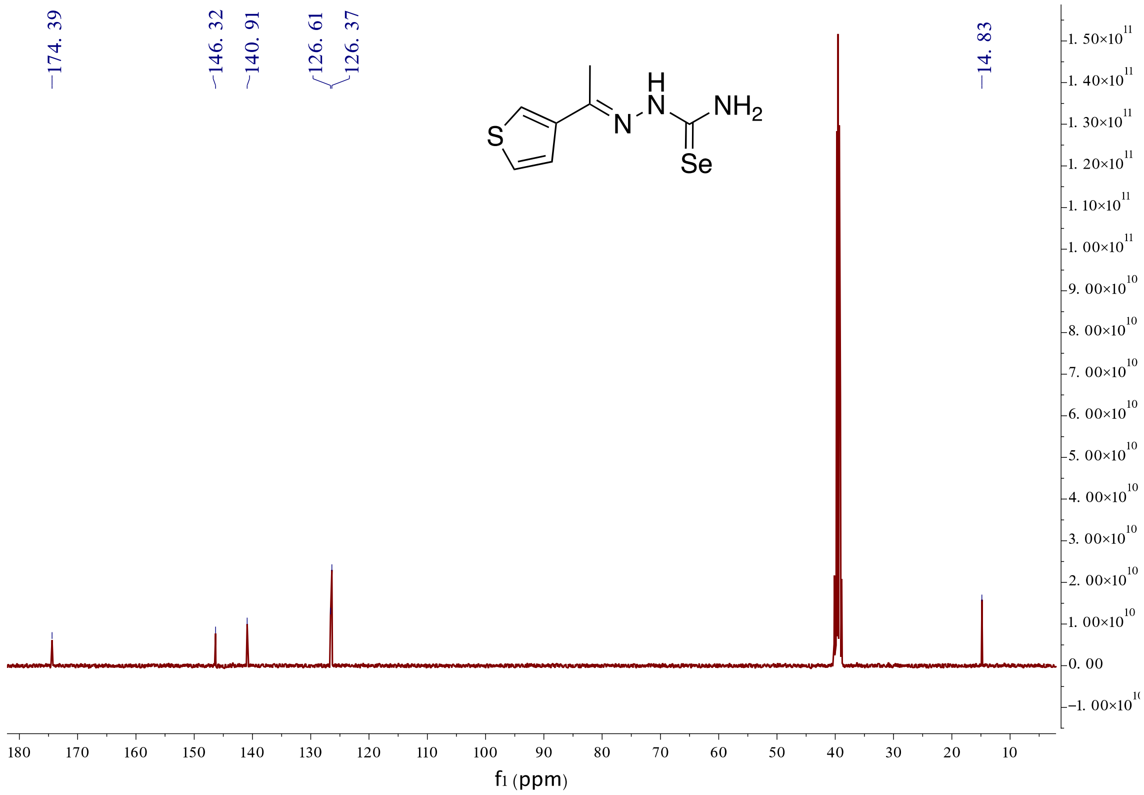

Figure S58. ^{13}C-NMR (up) and ^{77}Se-NMR (down) of compound ***Se*1l**.

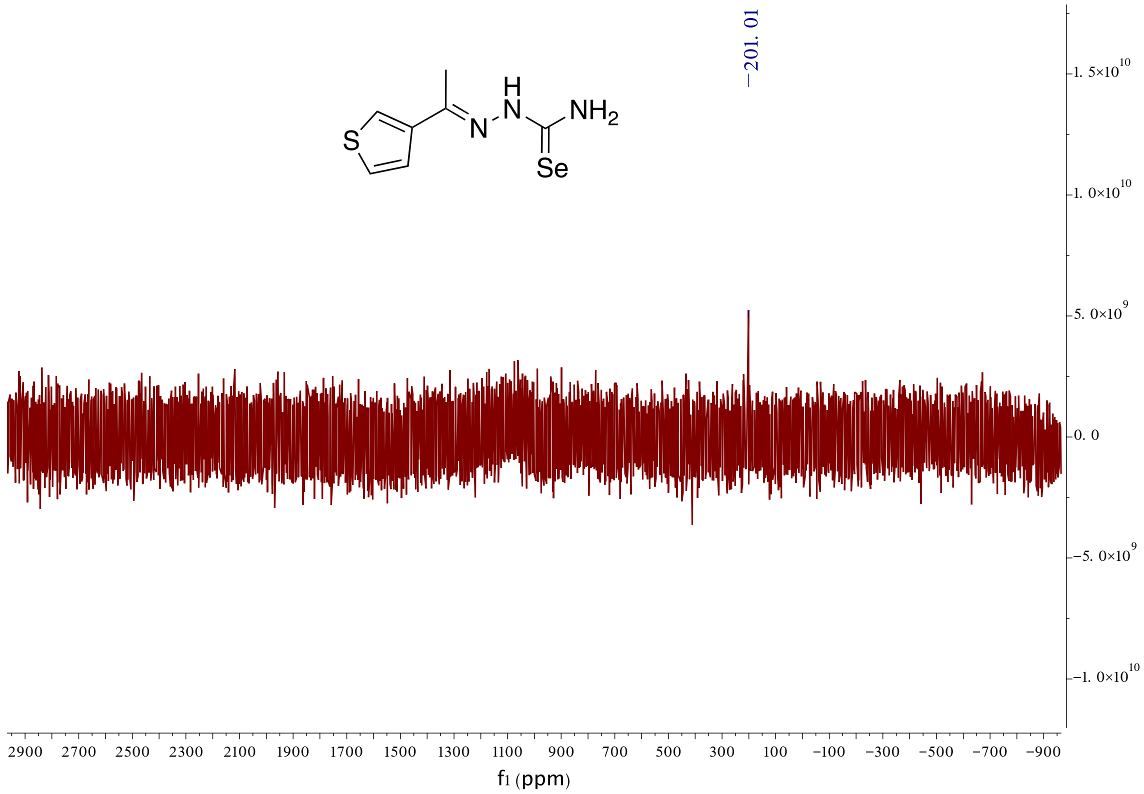

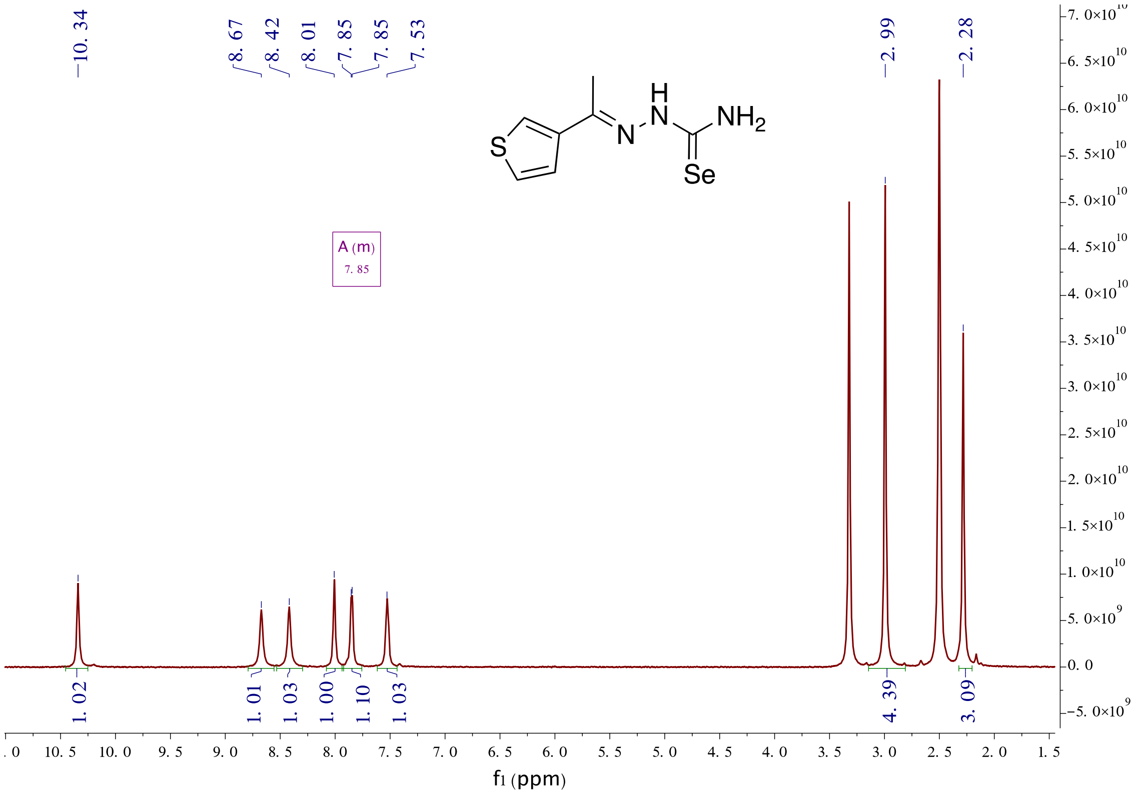

Figure S59. qNMR of compound ***Se*1l** (up) and IR of compound ***Se*1o** (down).

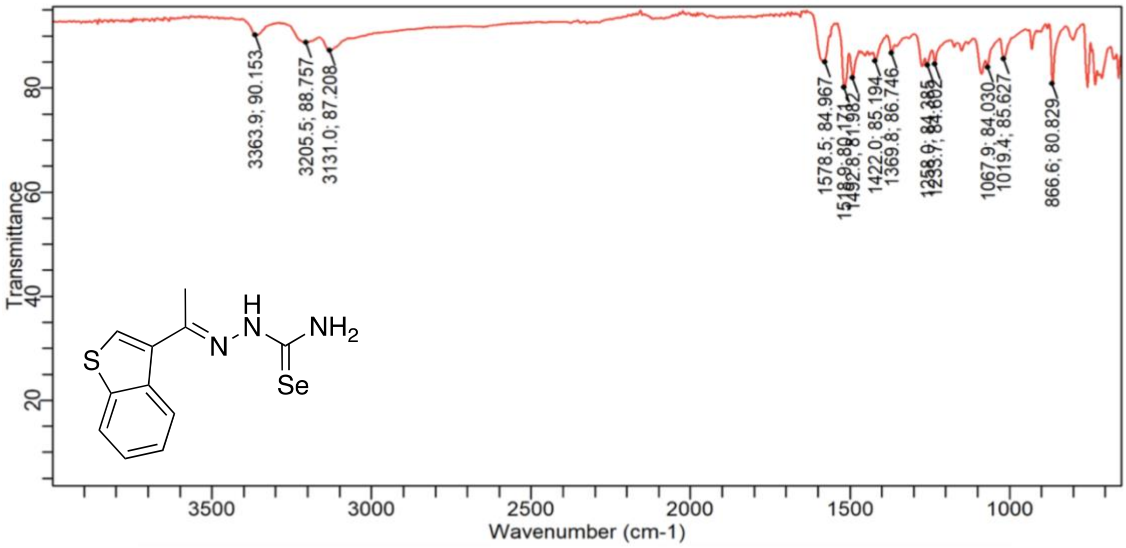

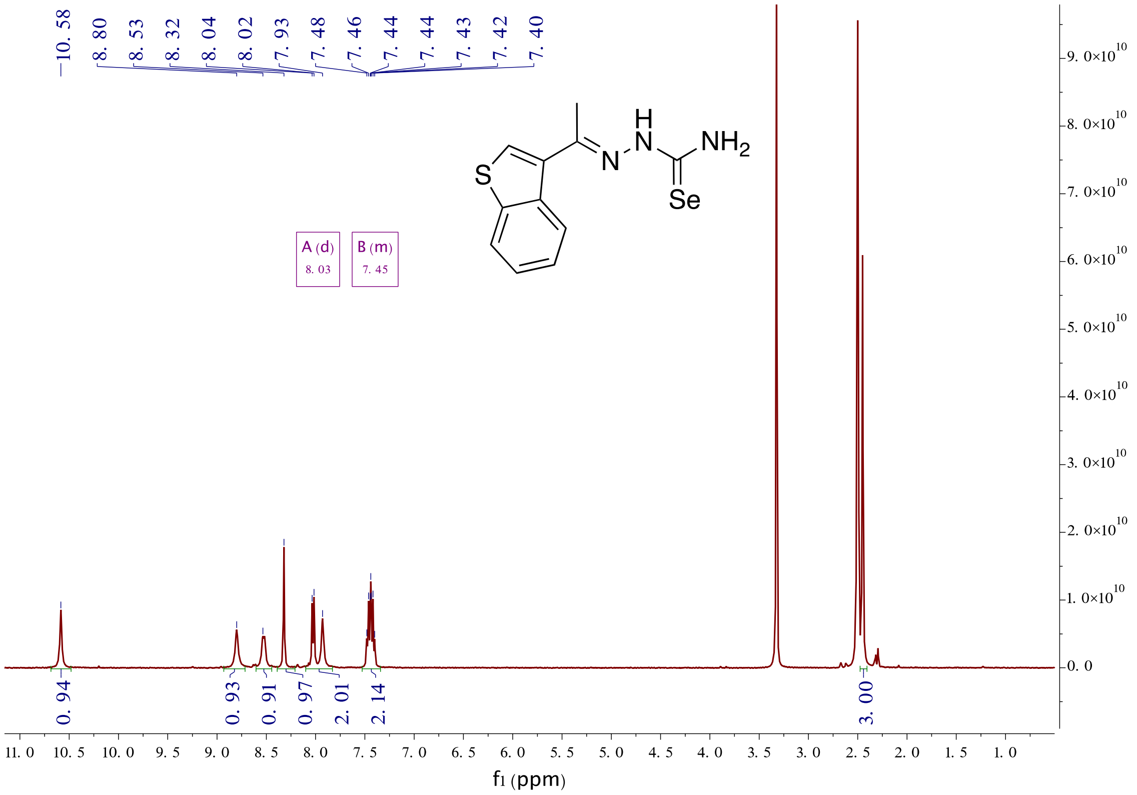

Figure S60. ^{1}H-NMR (up) and ^{13}C-NMR (down) of compound ***Se*1o**.

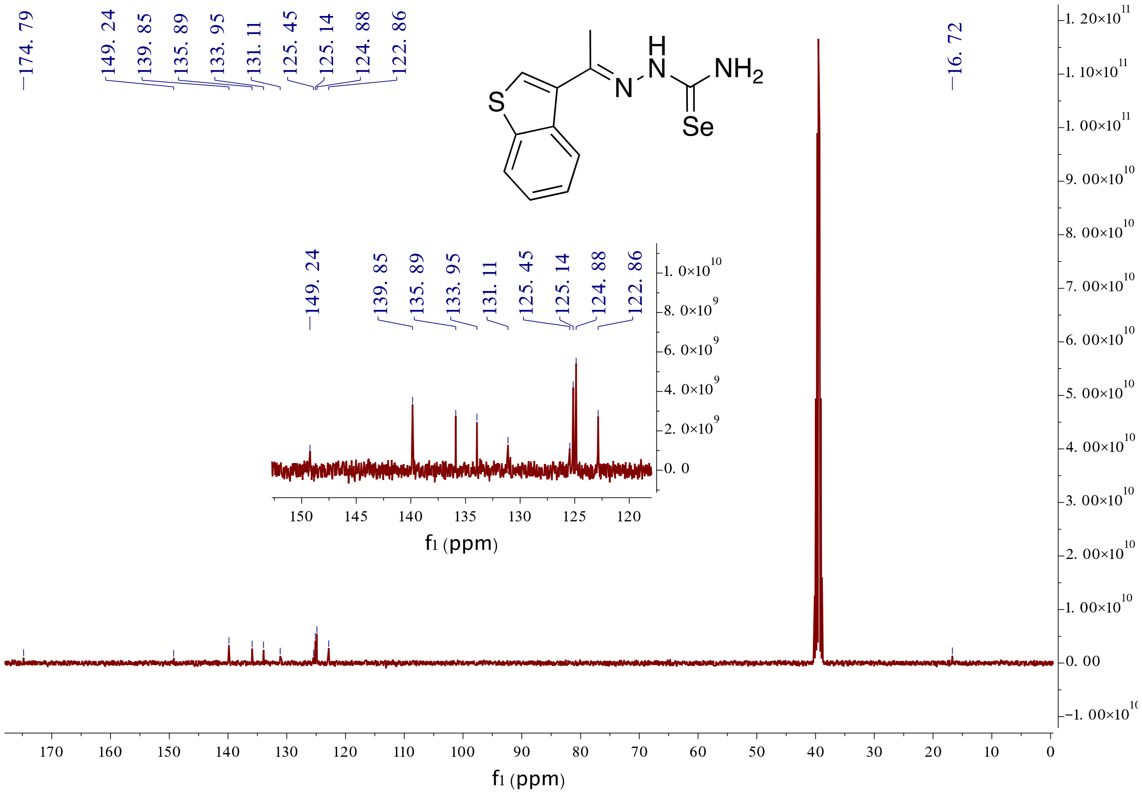

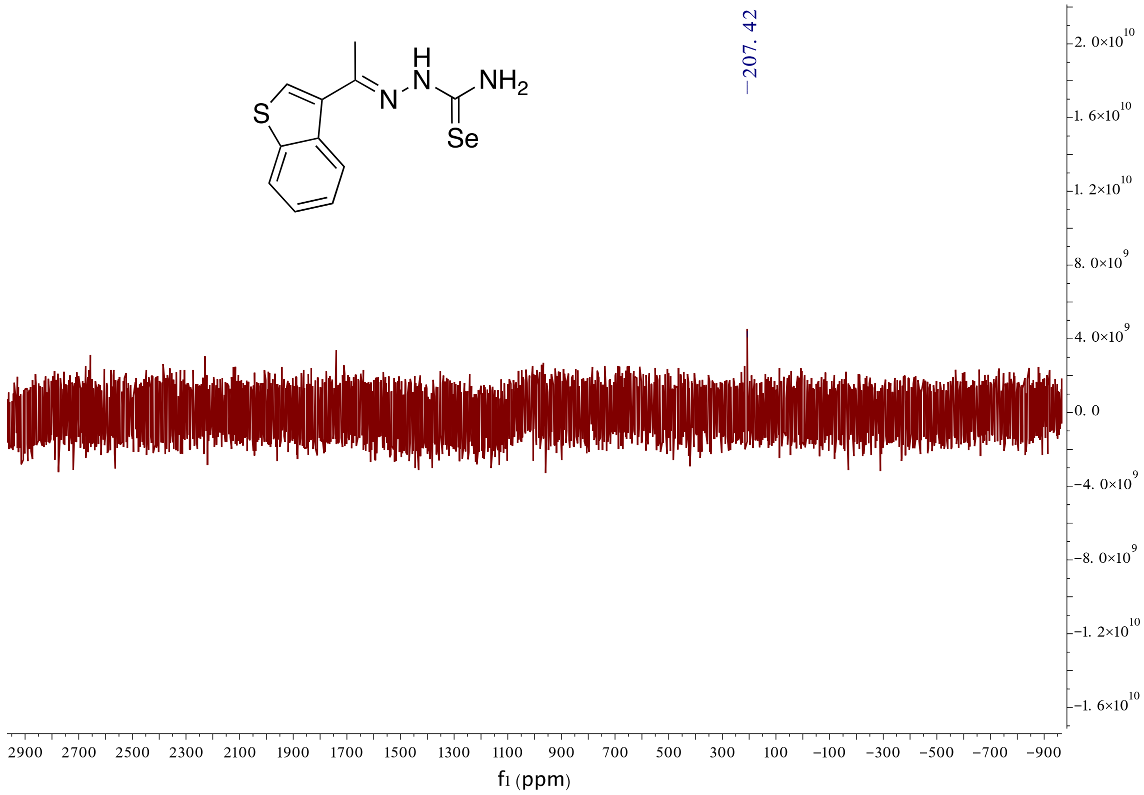

Figure S61. ^{77}Se-NMR (up) and qNMR (down) of compound ***Se*1o**.

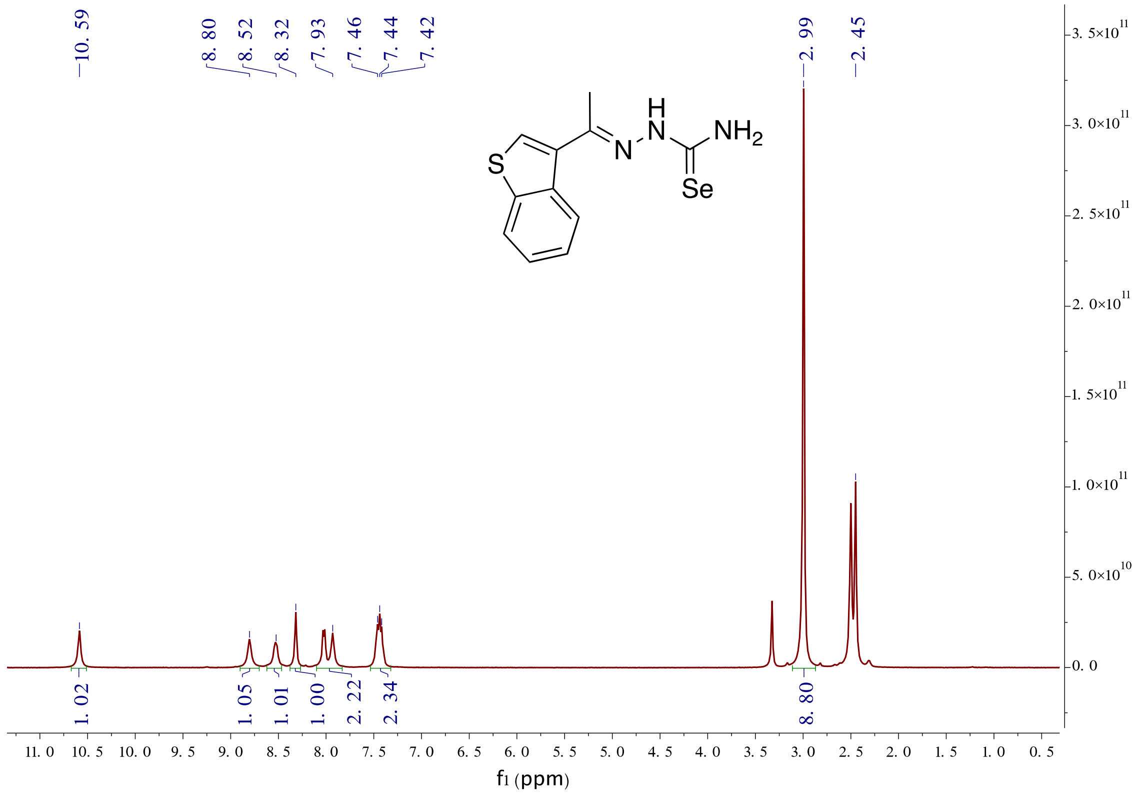

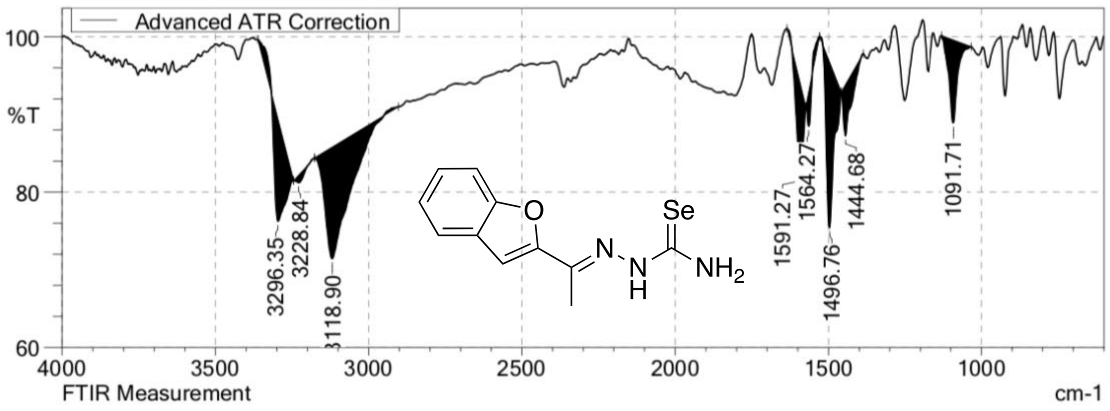

Figure S62. IR (up) and ^{1}H-NMR (down) of compound ***Se*1p**.

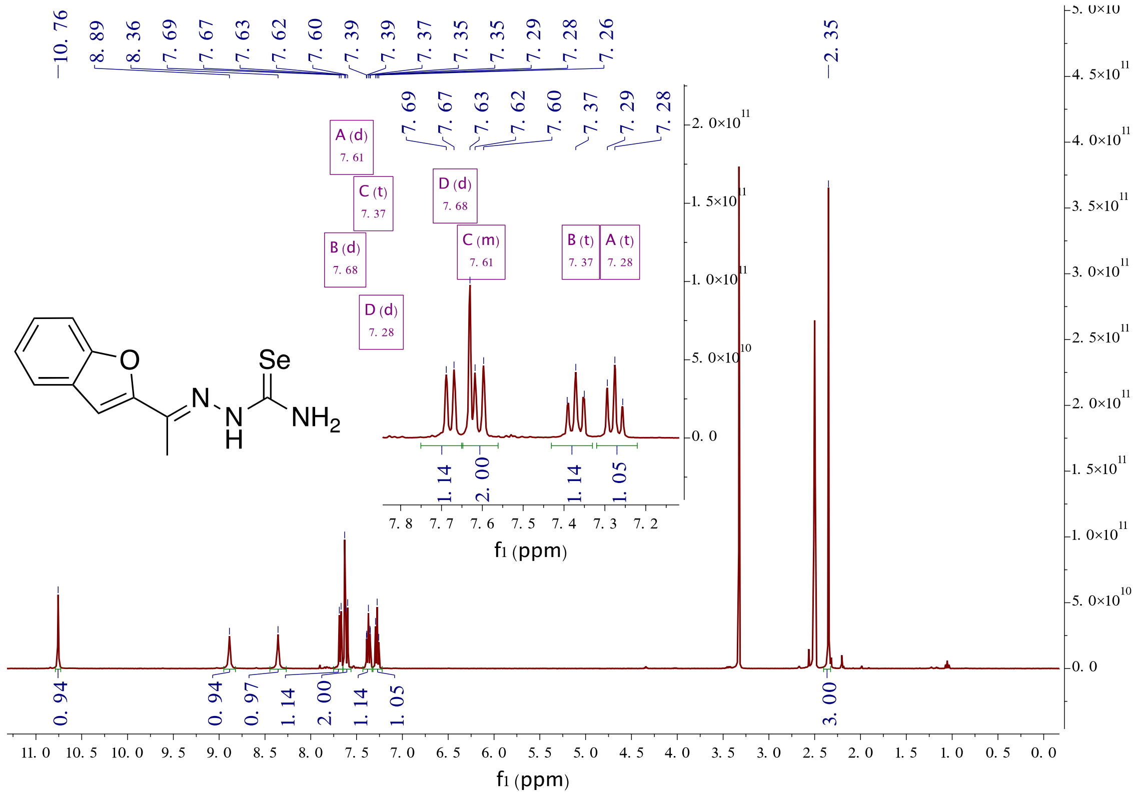

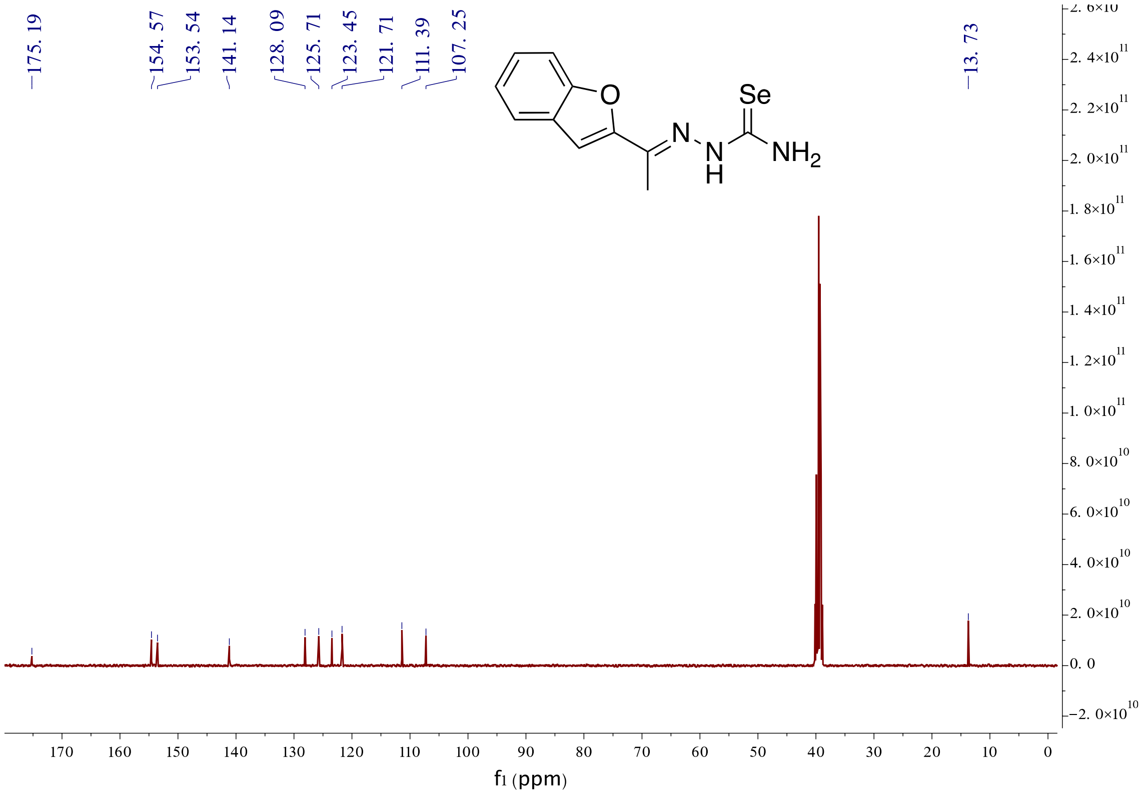

Figure S63. ^{13}C-NMR (up) and ^{77}Se-NMR (down) of compound ***Se*1p**.

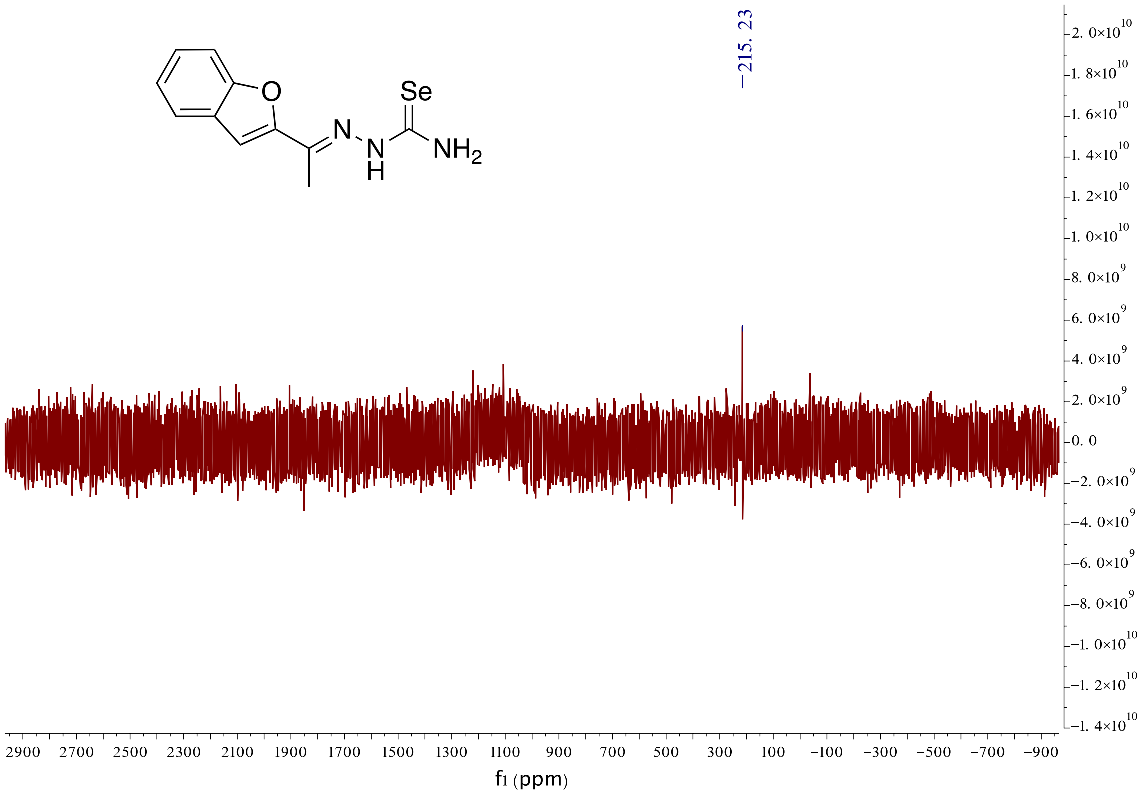

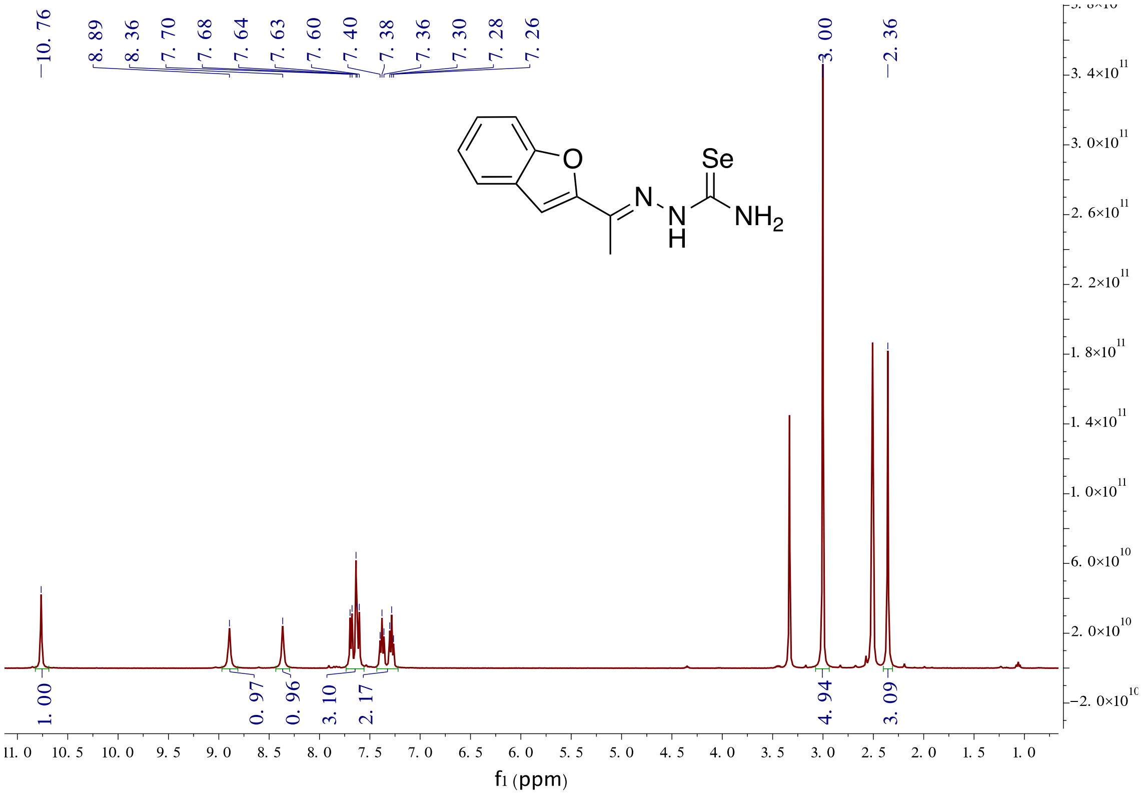

Figure S64. qNMR of compound ***Se*1p** (up) and IR of compound ***Se*1q** (down).

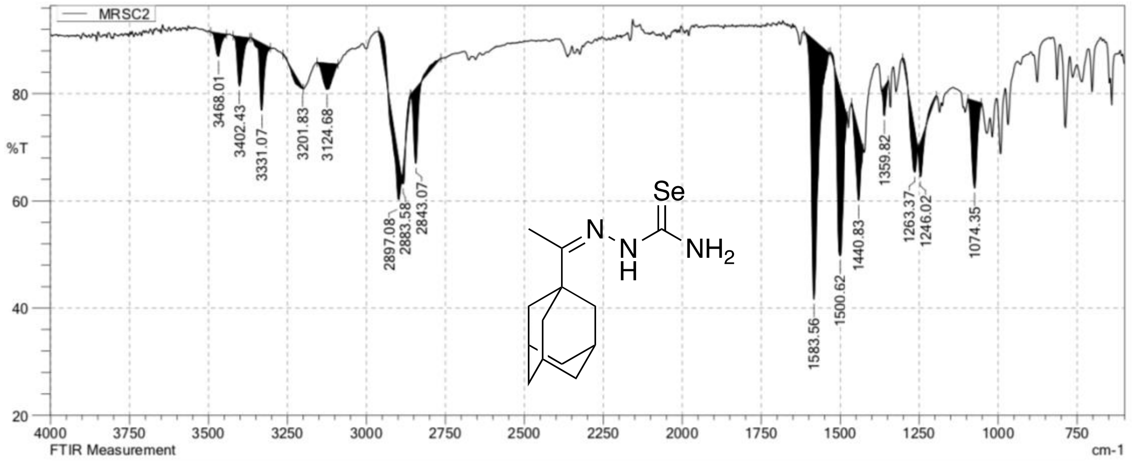

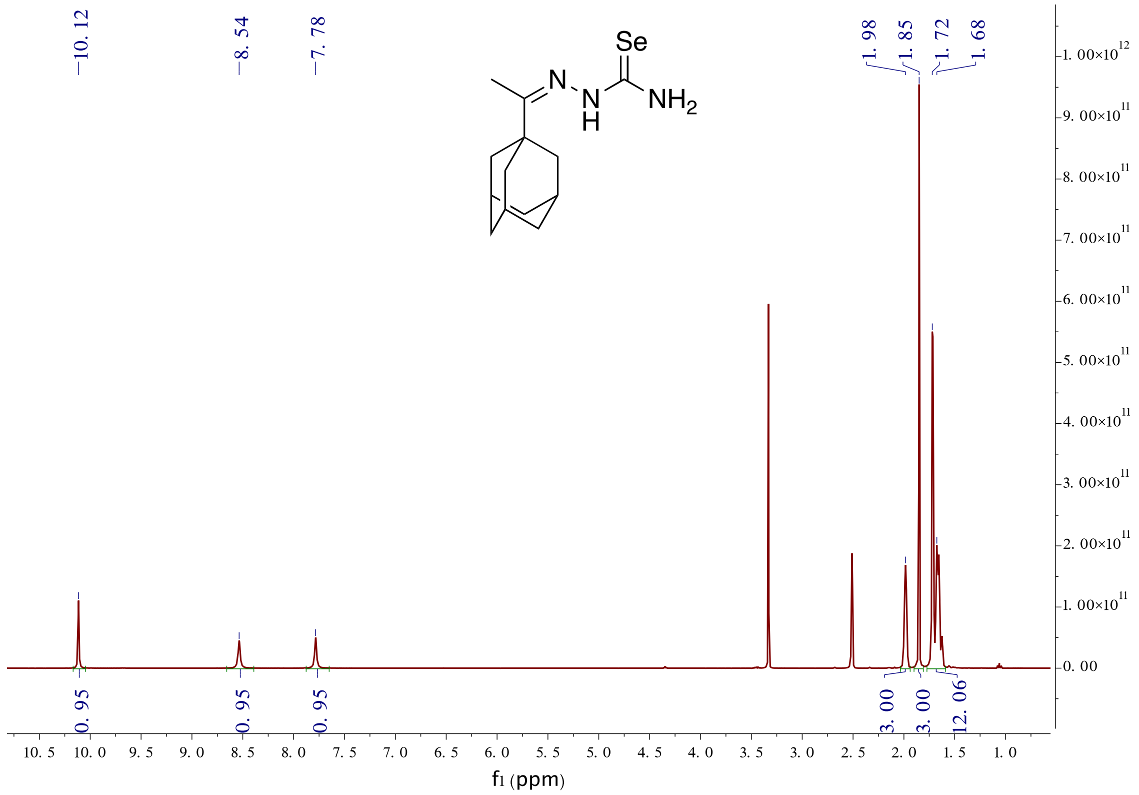

Figure S65. ^{1}H-NMR (up) and ^{13}C-NMR (down) of compound ***Se*1q**.

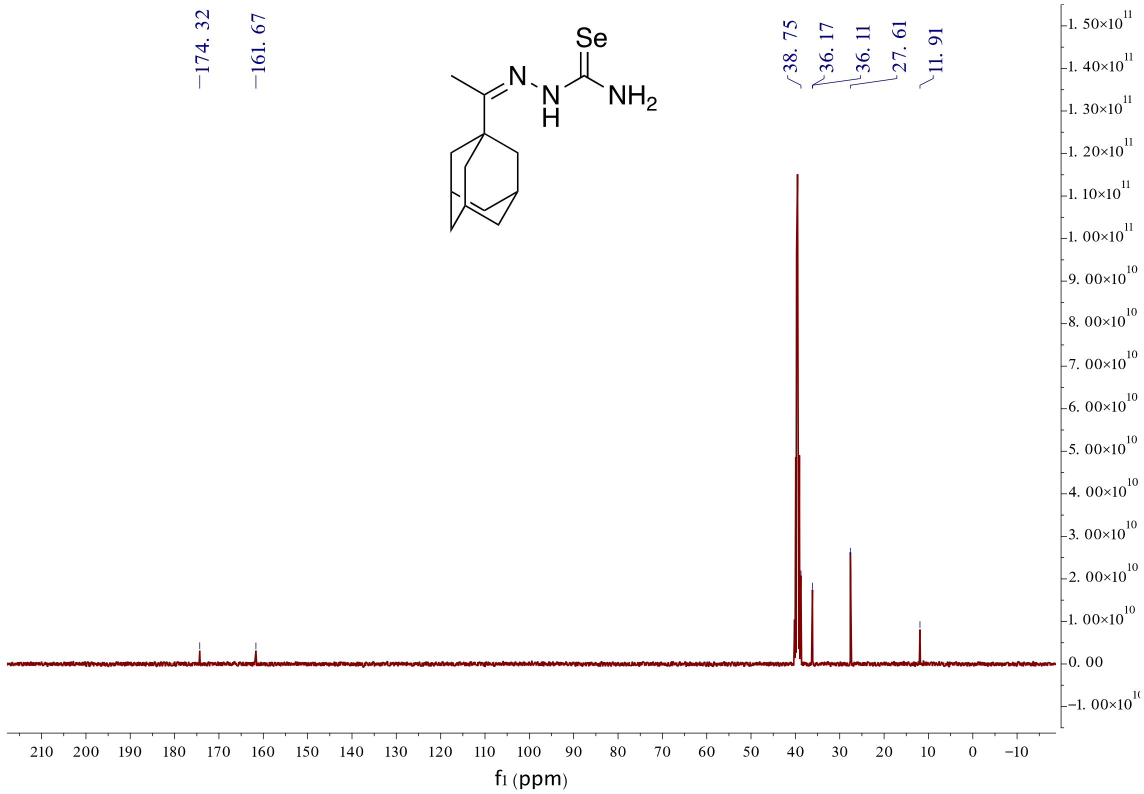

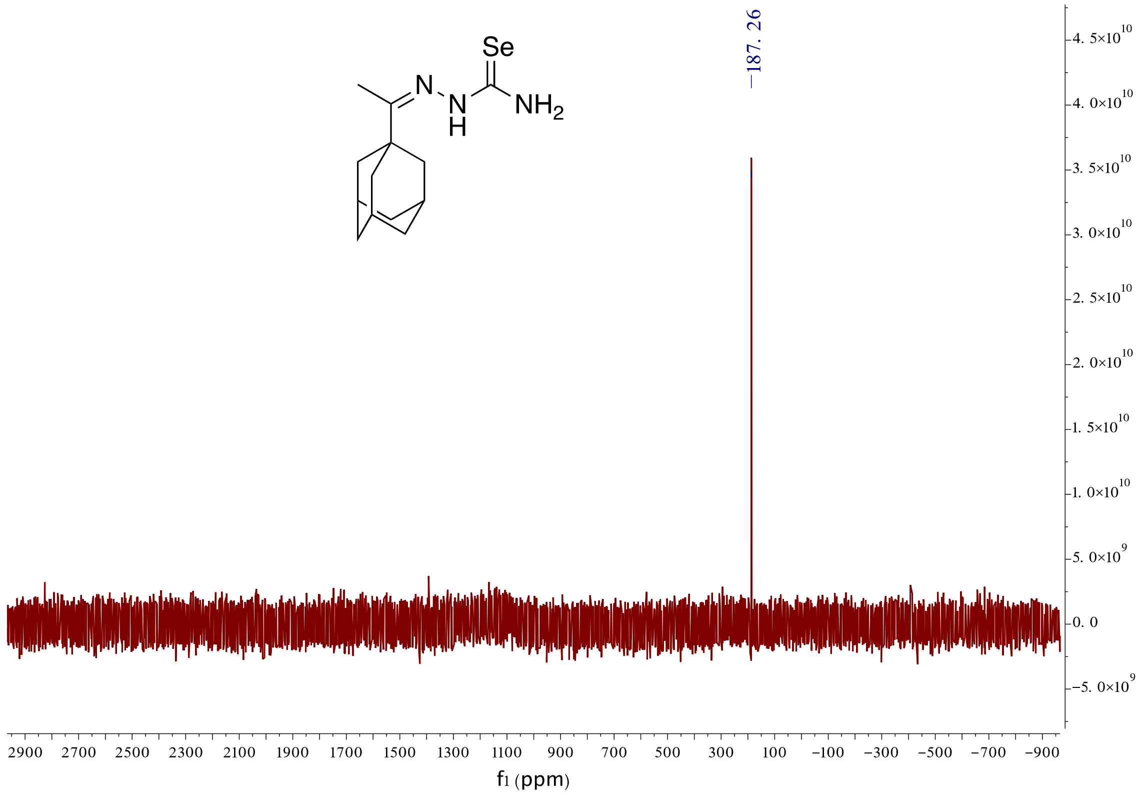

Figure S66. ^{77}Se-NMR (up) and qNMR (down) of compound ***Se*1q**.

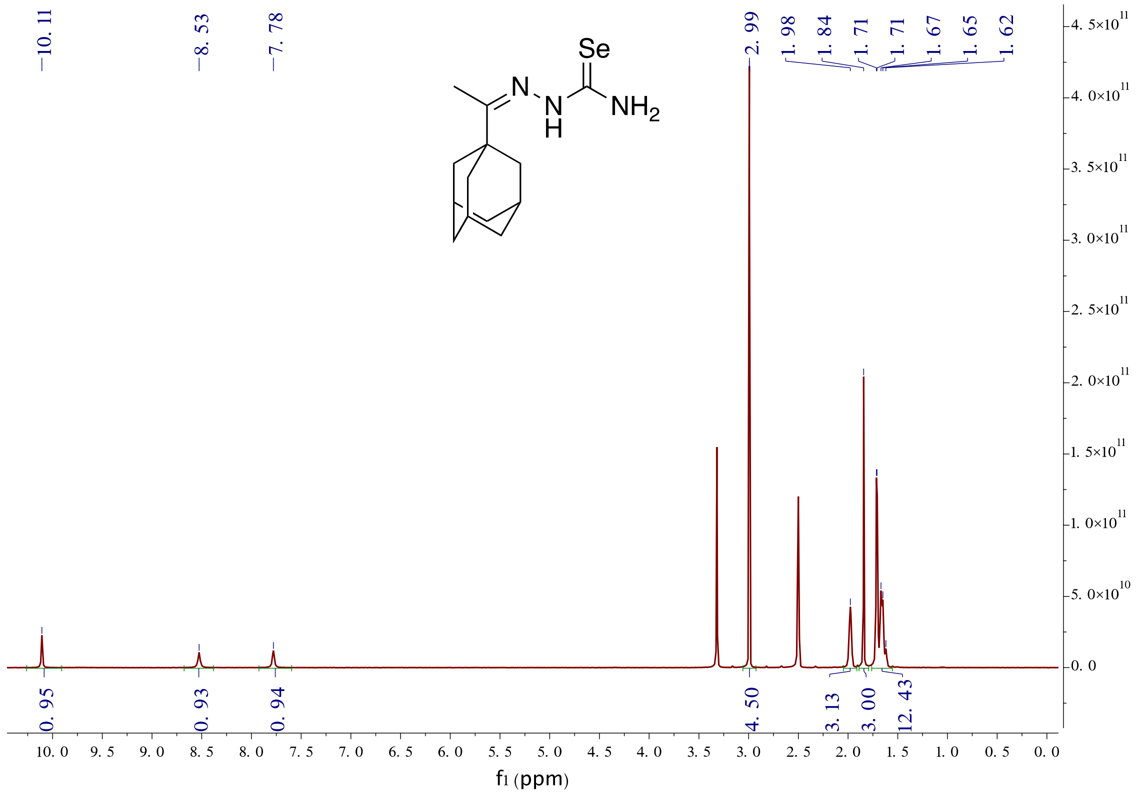

c. *S2* series

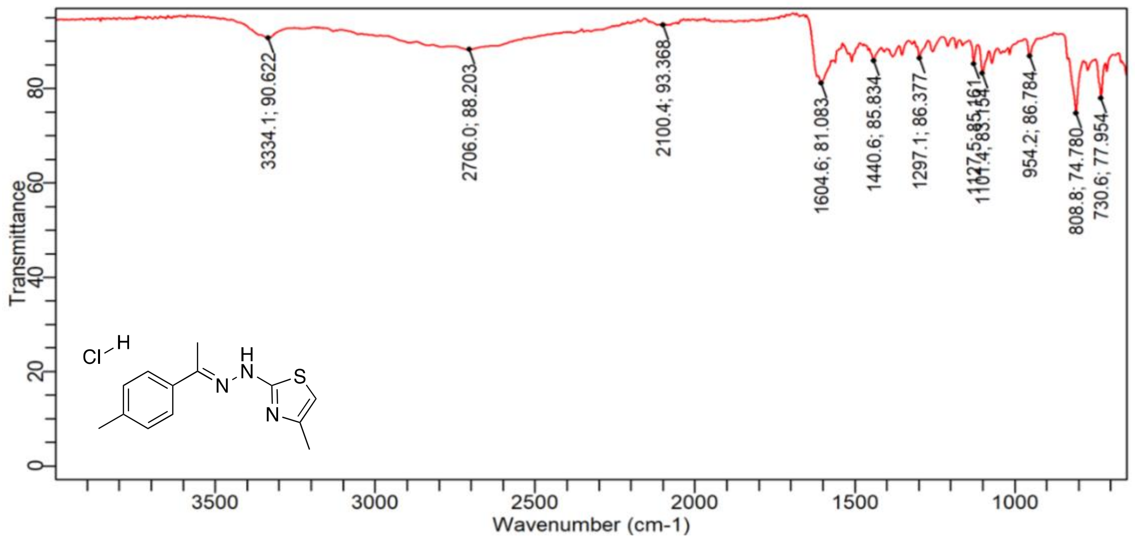

Figure S67. IR (up) and ^{1}H-NMR (down) of compound ***S2*a**.

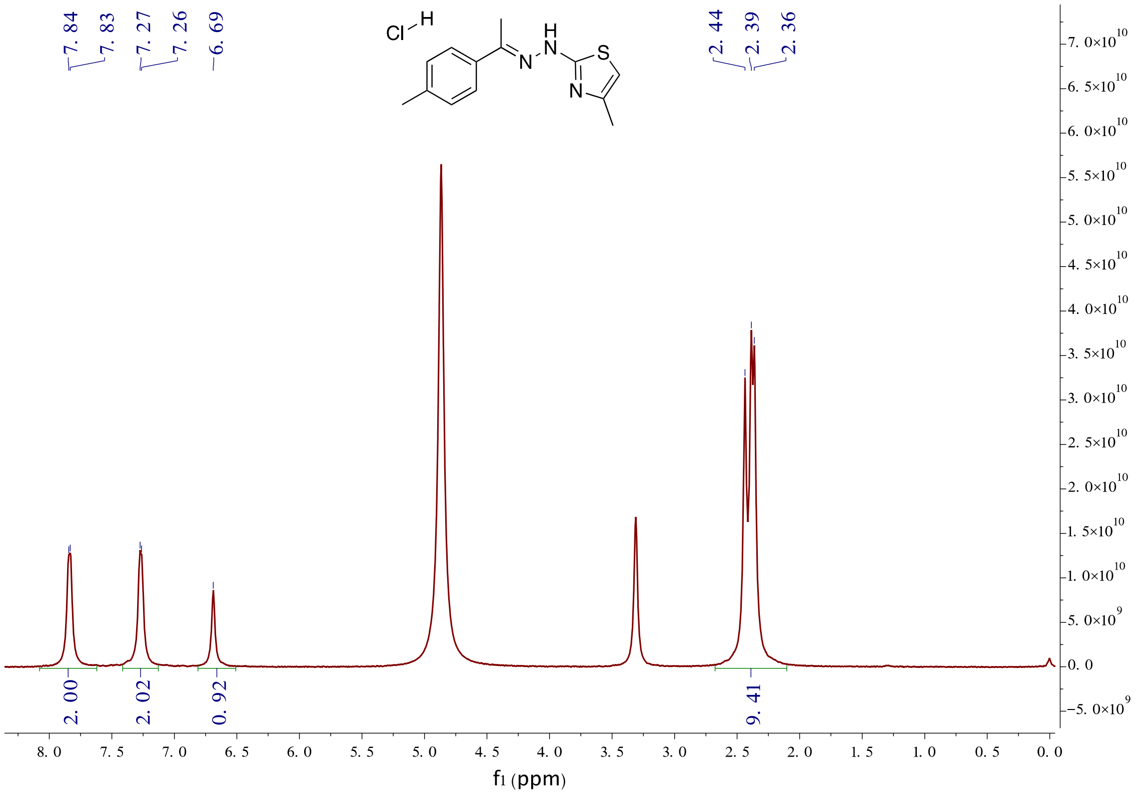

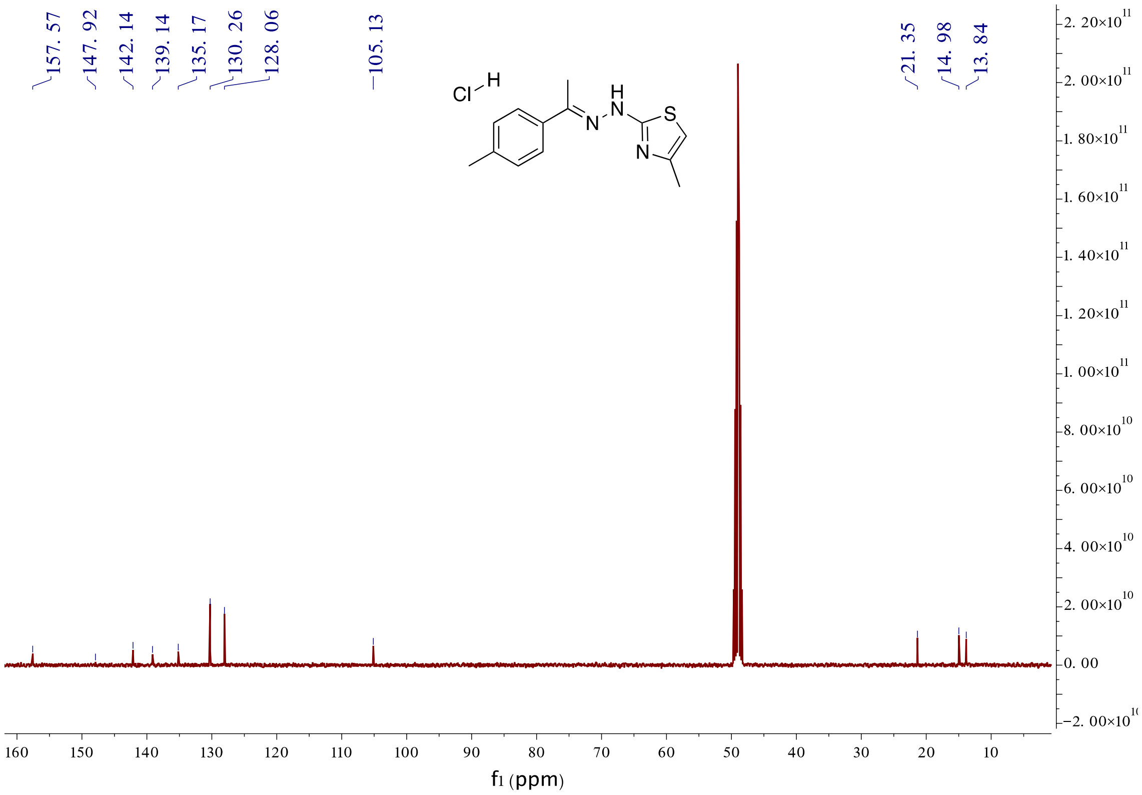

Figure S68. ^{13}C-NMR (up) and qNMR (down) of compound ***S*2a**.

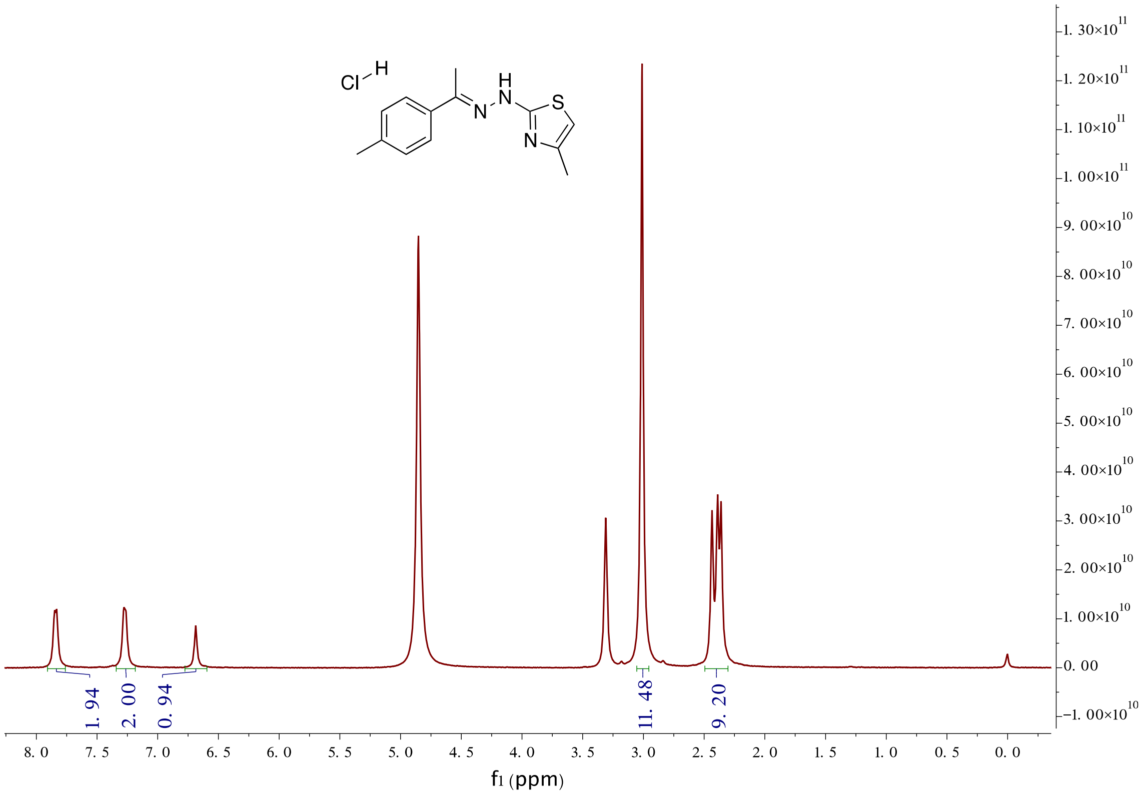

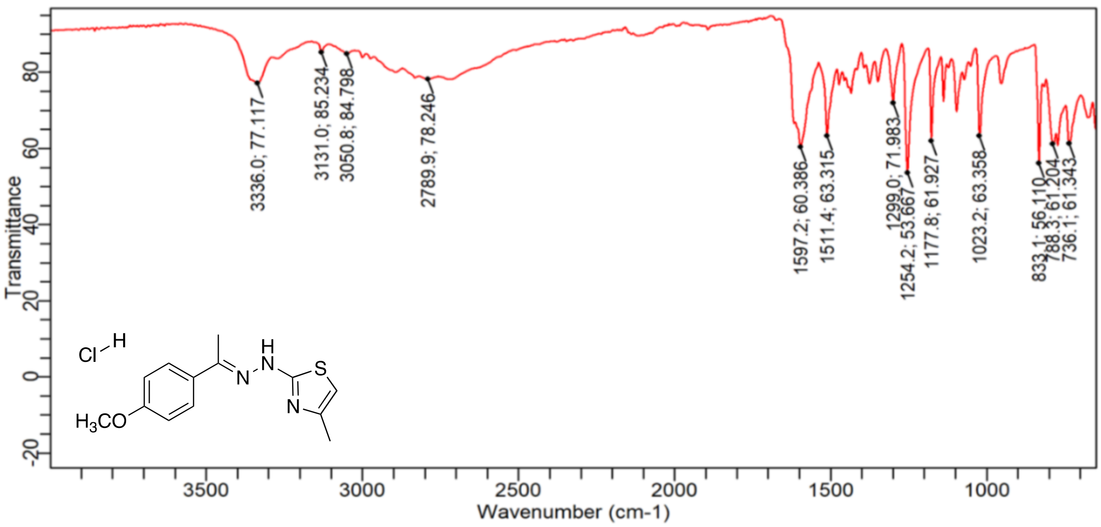

Figure S69. IR (up) and ^{1}H-NMR (down) of compound ***S*2b**.

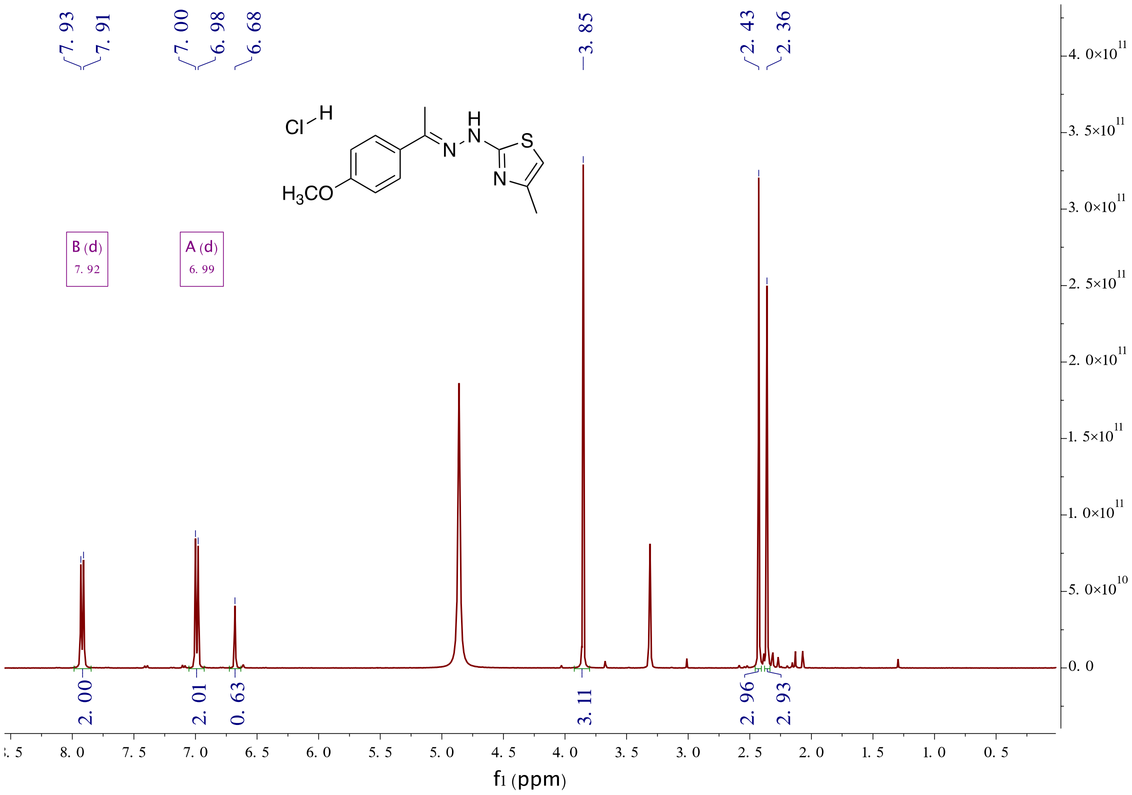

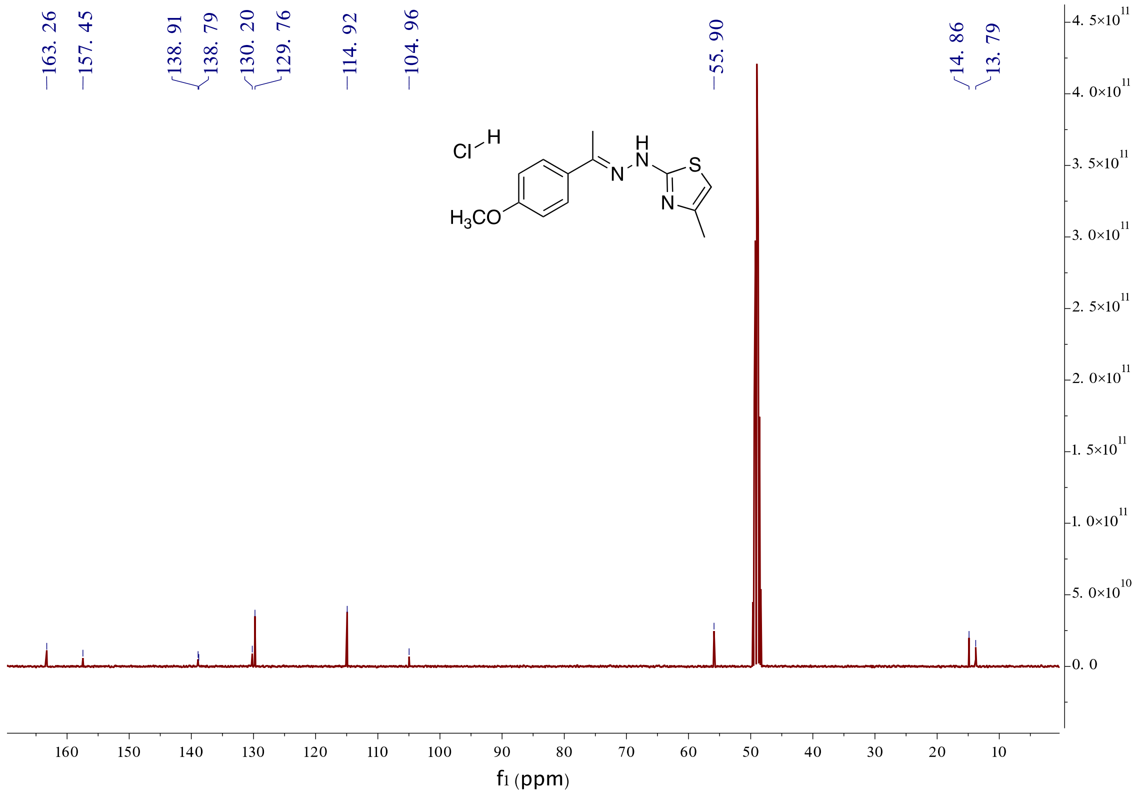

Figure S70. ^{13}C-NMR (up) and qNMR (down) of compound ***S*2b**.

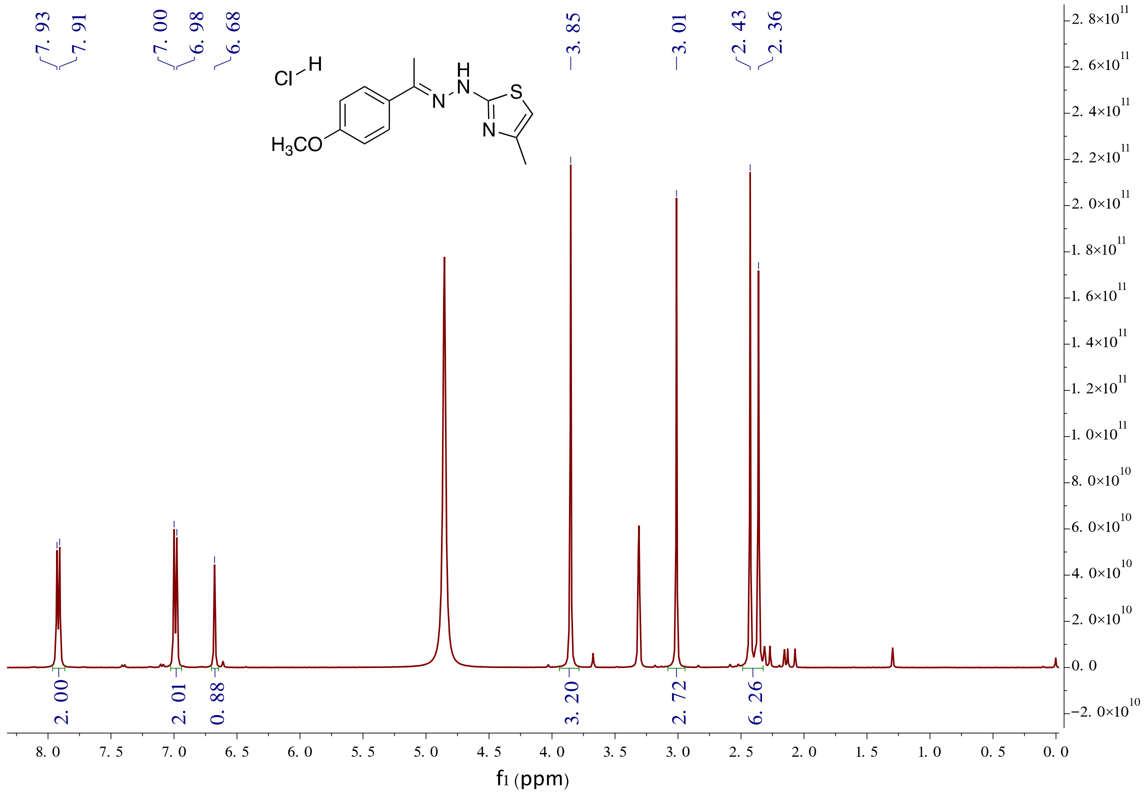

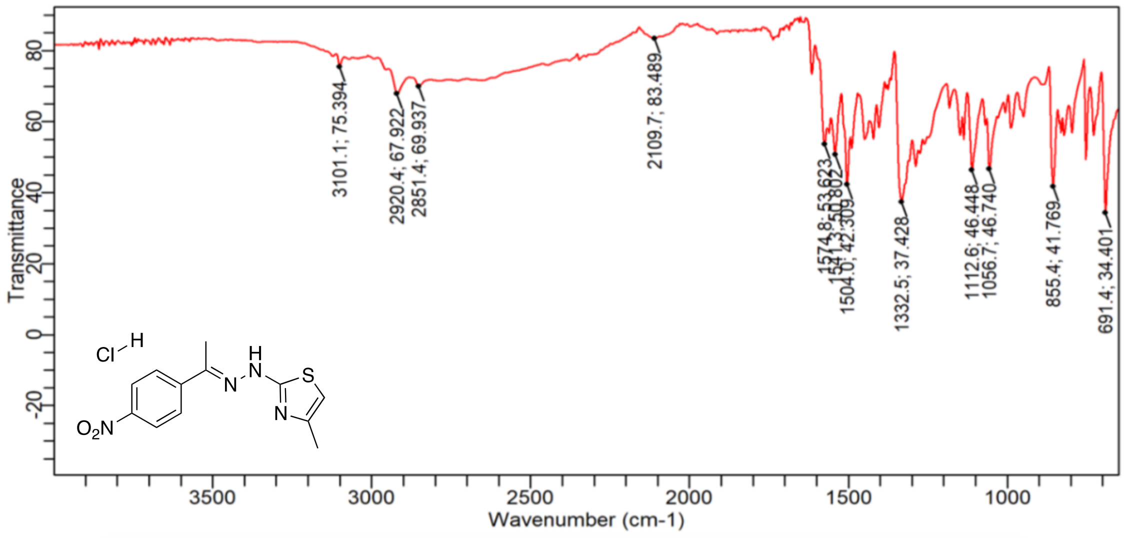

Figure S71. IR (up) and ^{1}H-NMR (down) of compound ***S*2cs**.

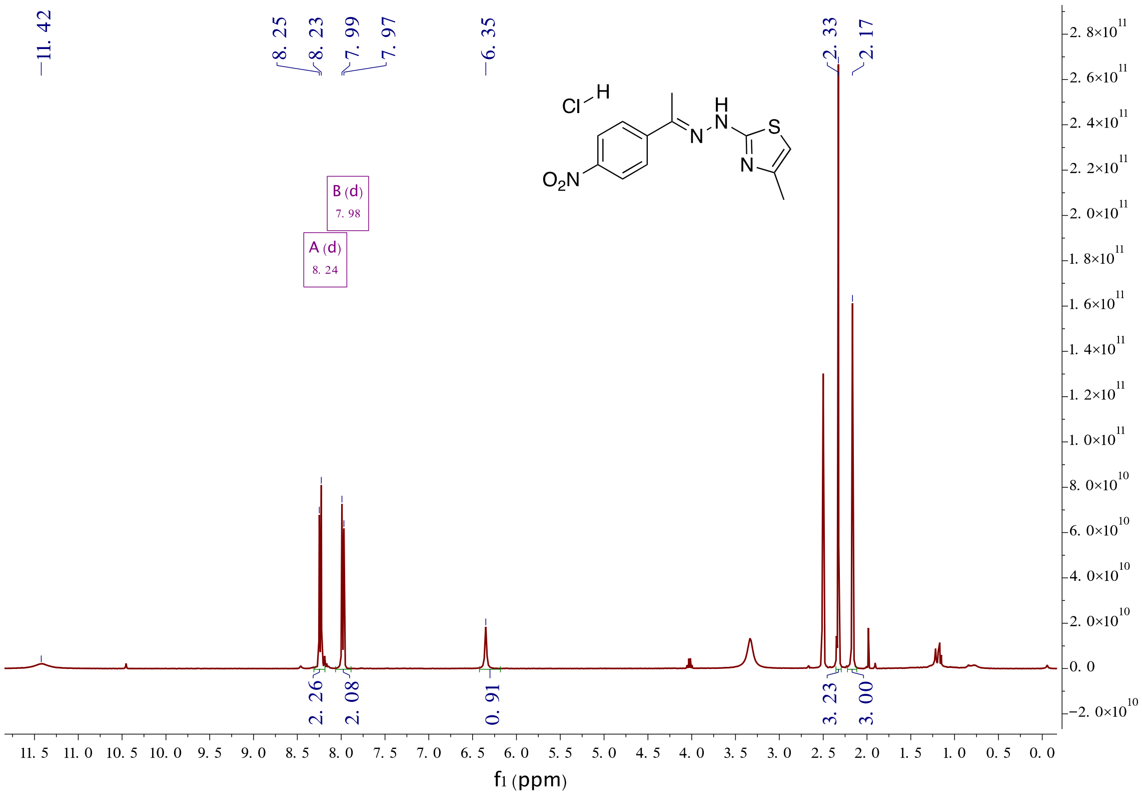

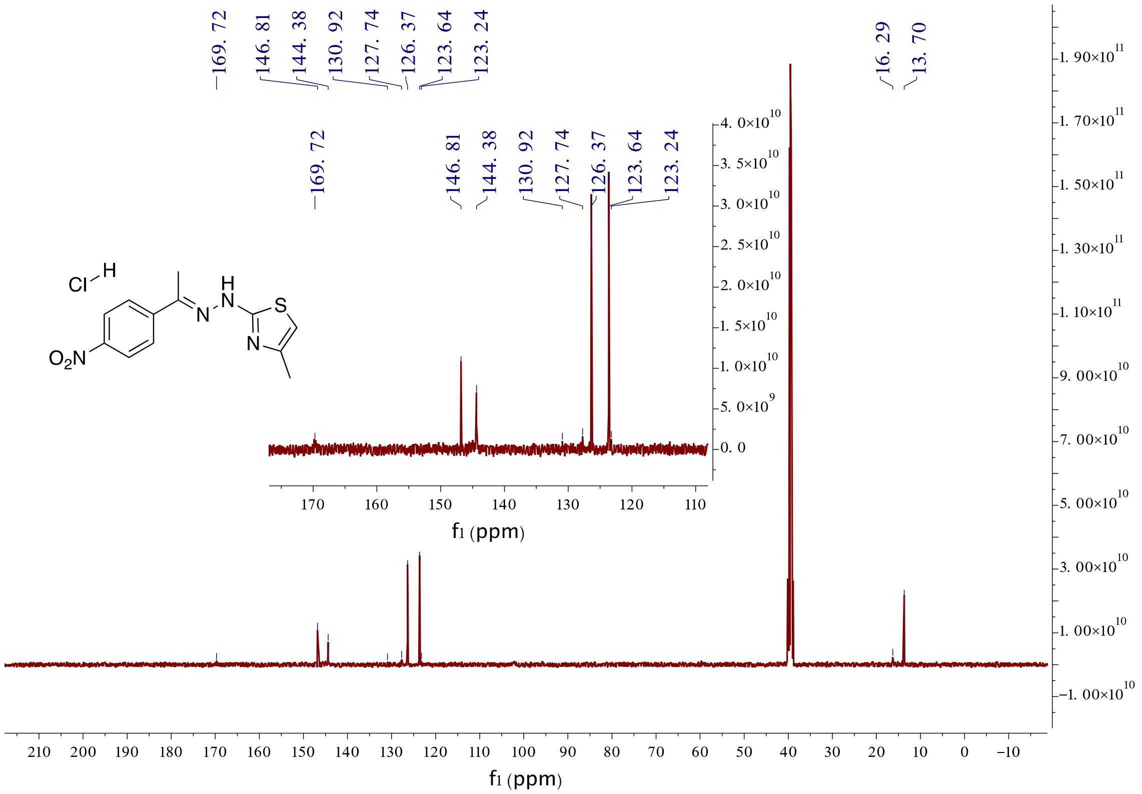

Figure S72. ^{13}C-NMR (up) and qNMR (down) of compound ***S*2c**.

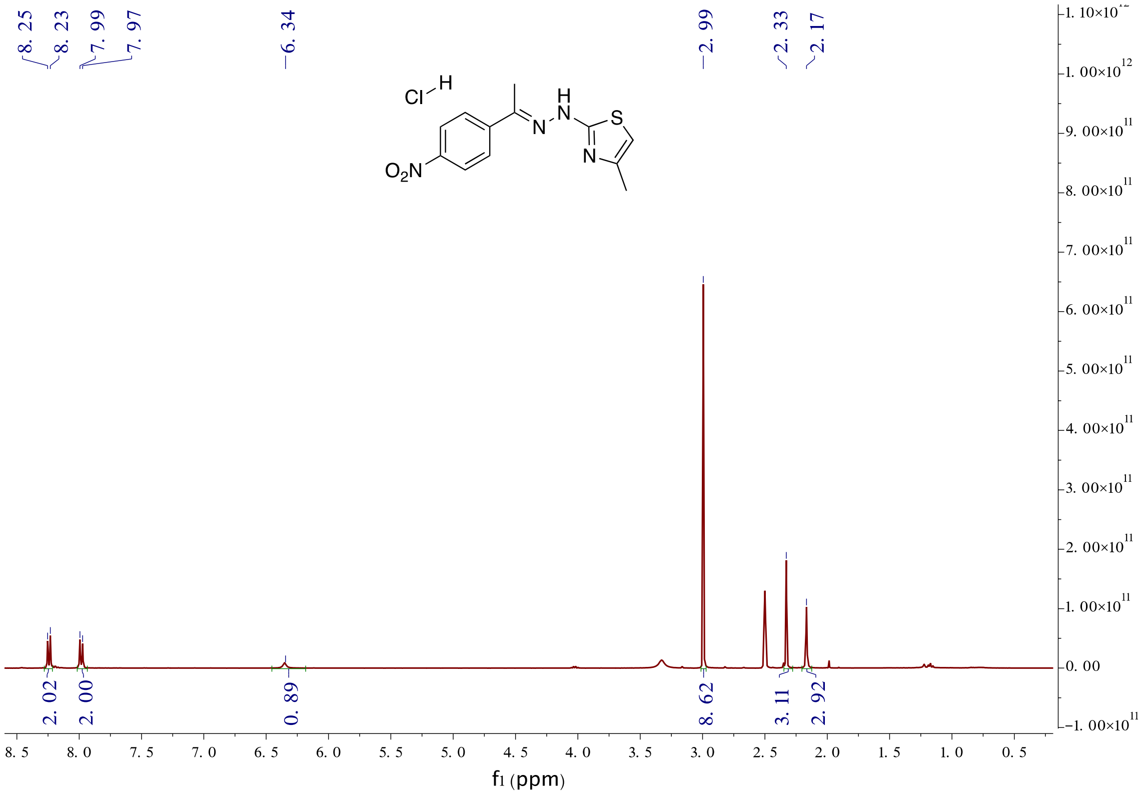

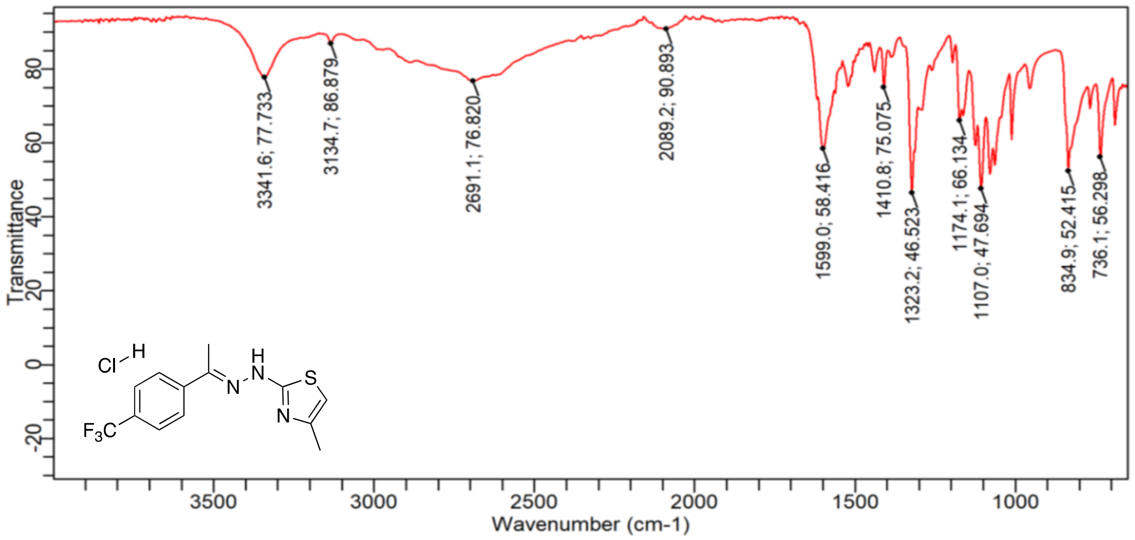

Figure S73. IR (up) and ^{1}H-NMR (down) of compound ***S*2d**.

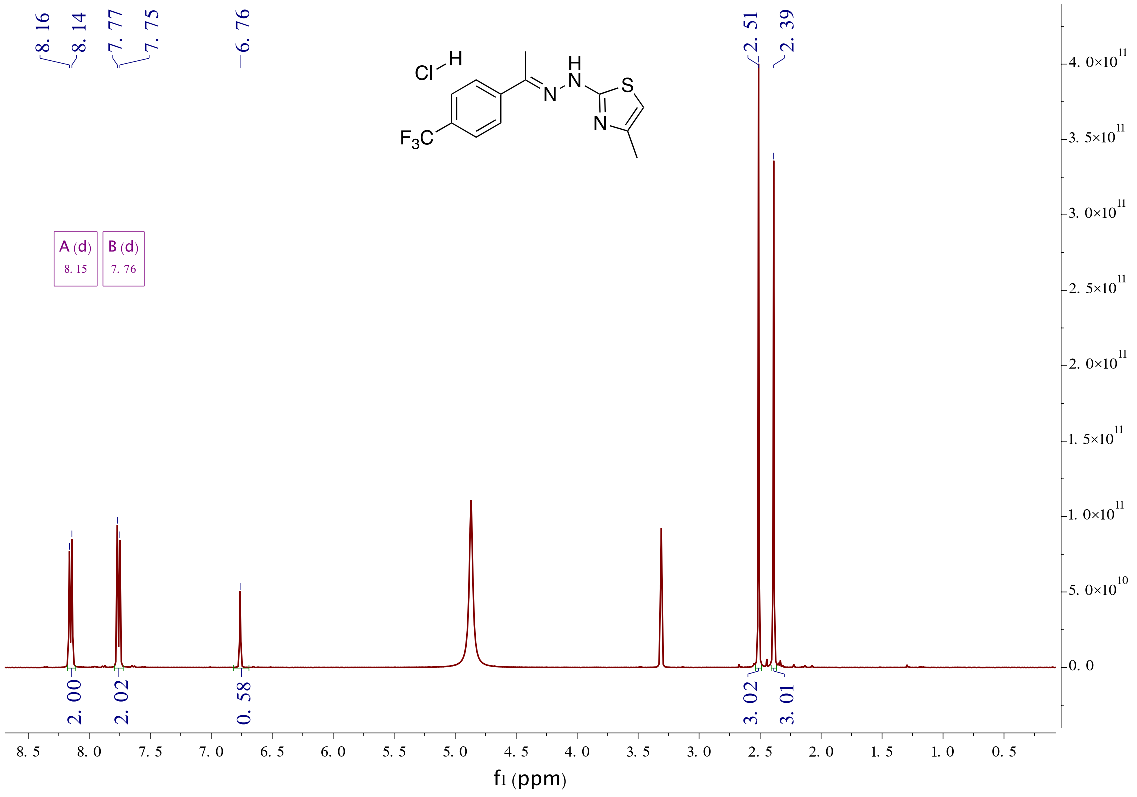

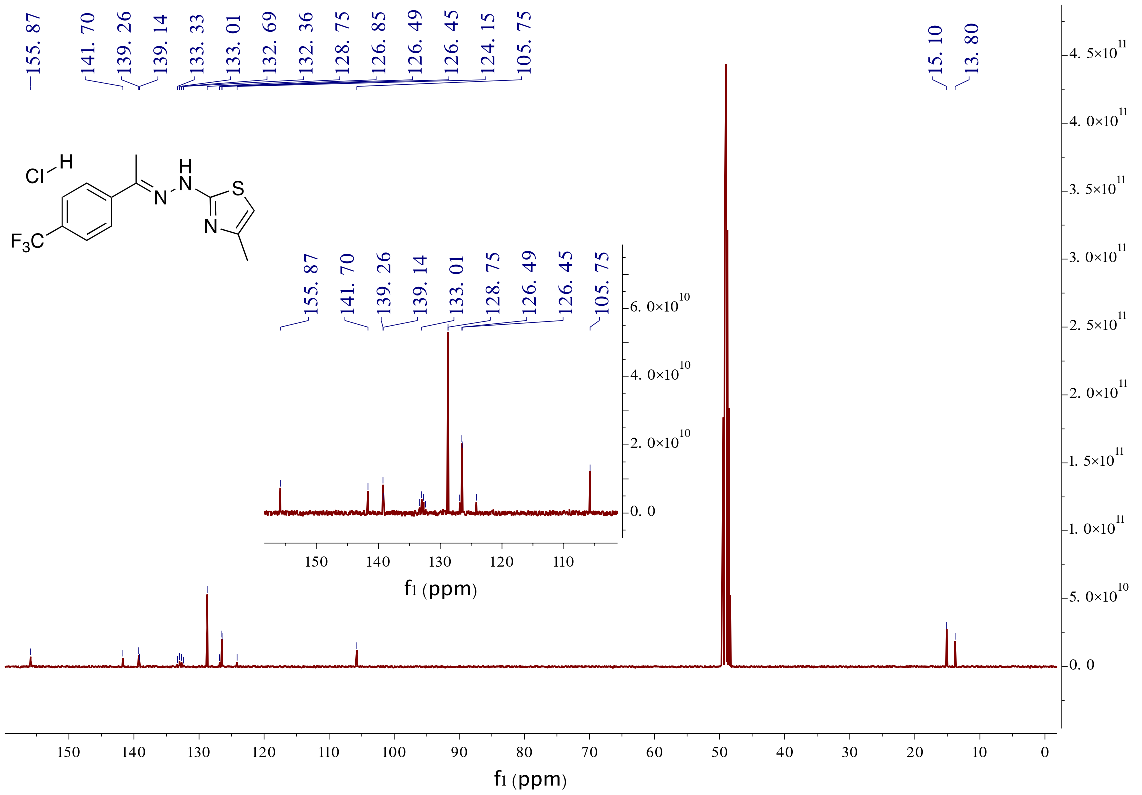

Figure S74. ^{13}C-NMR (up) and qNMR (down) of compound ***S*2d**.

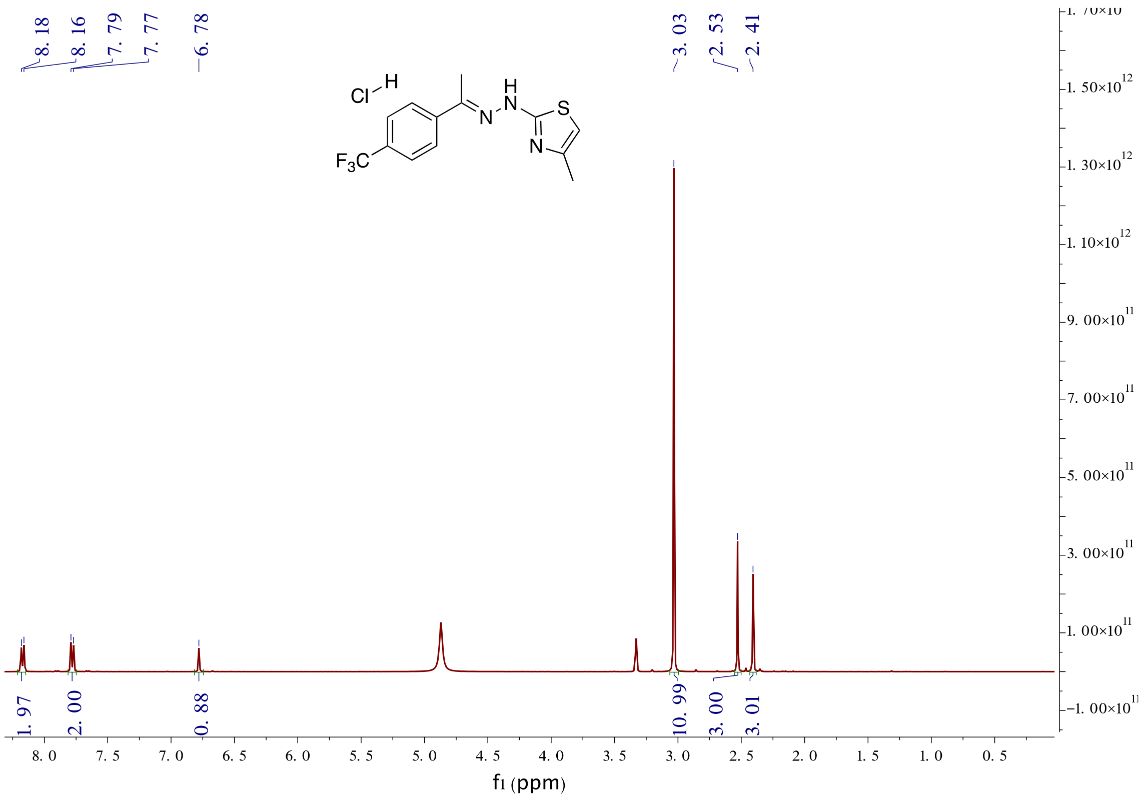

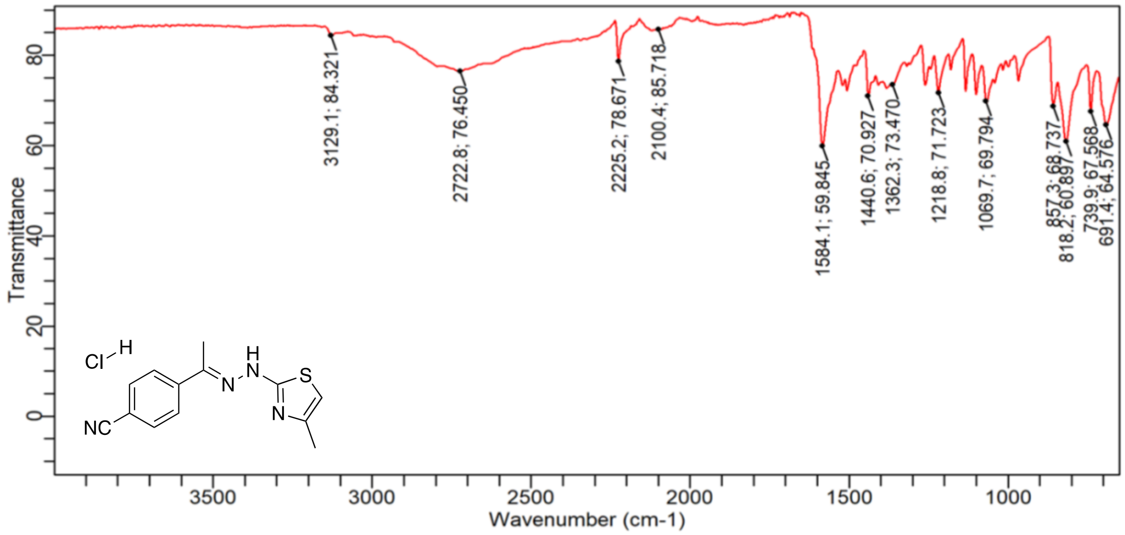

Figure S75. IR (up) and ¹H-NMR (down) of compound ***S*2e**.

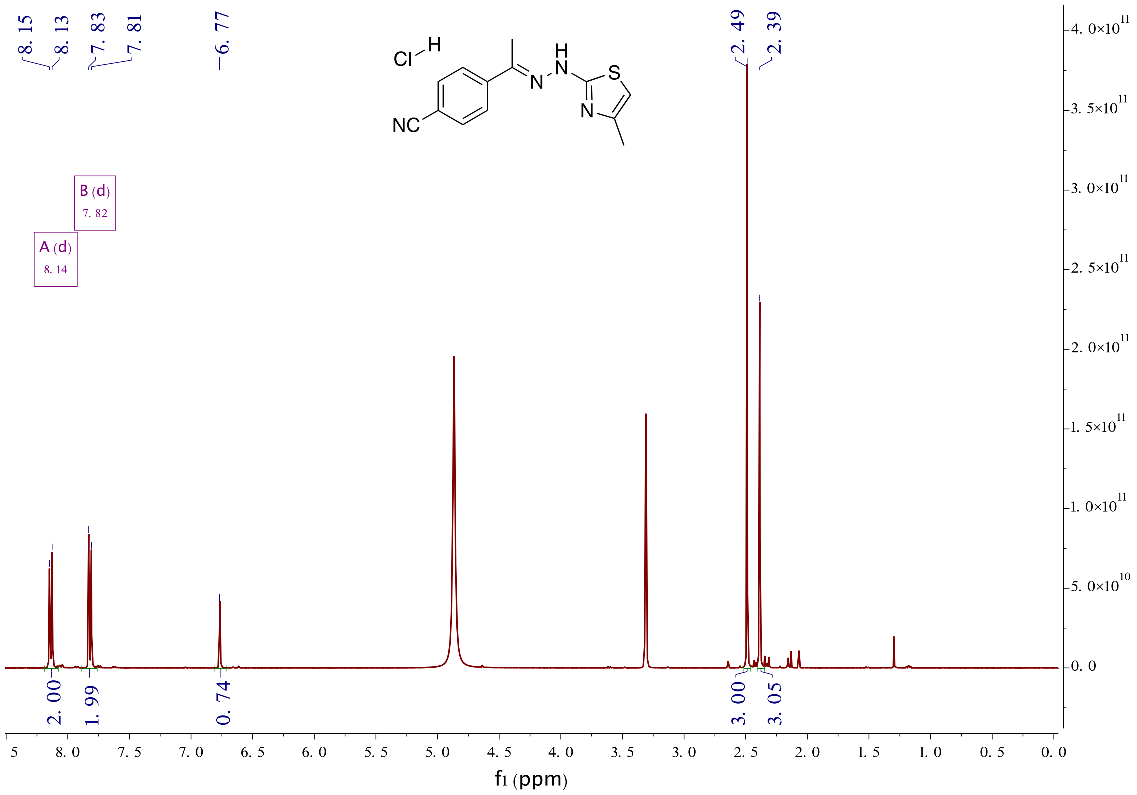

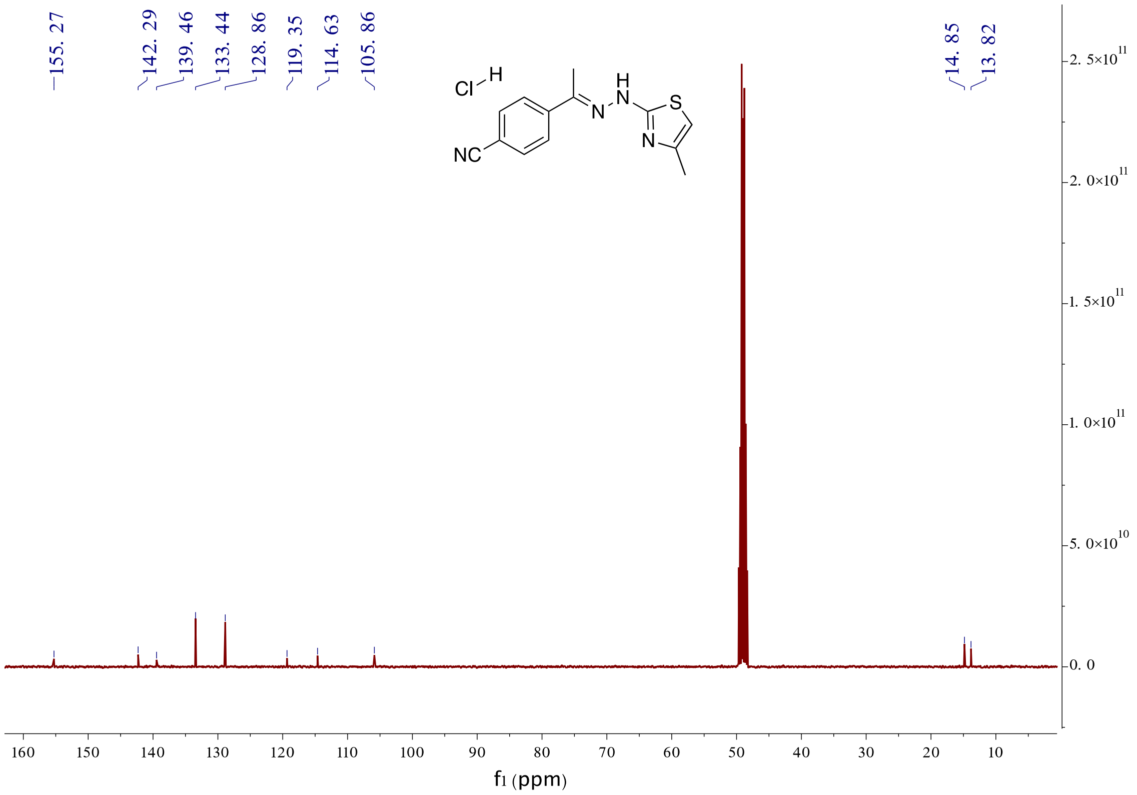

Figure S76. ^{13}C-NMR (up) and qNMR (down) of compound ***S*2e**.

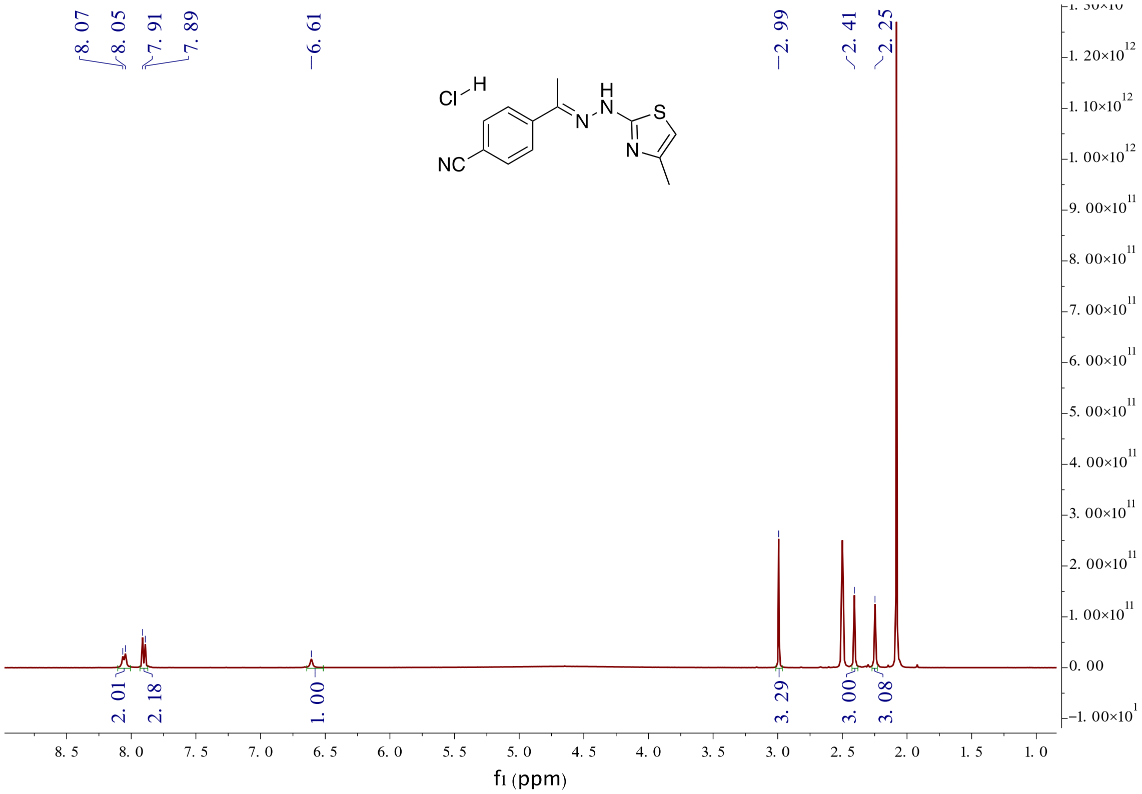

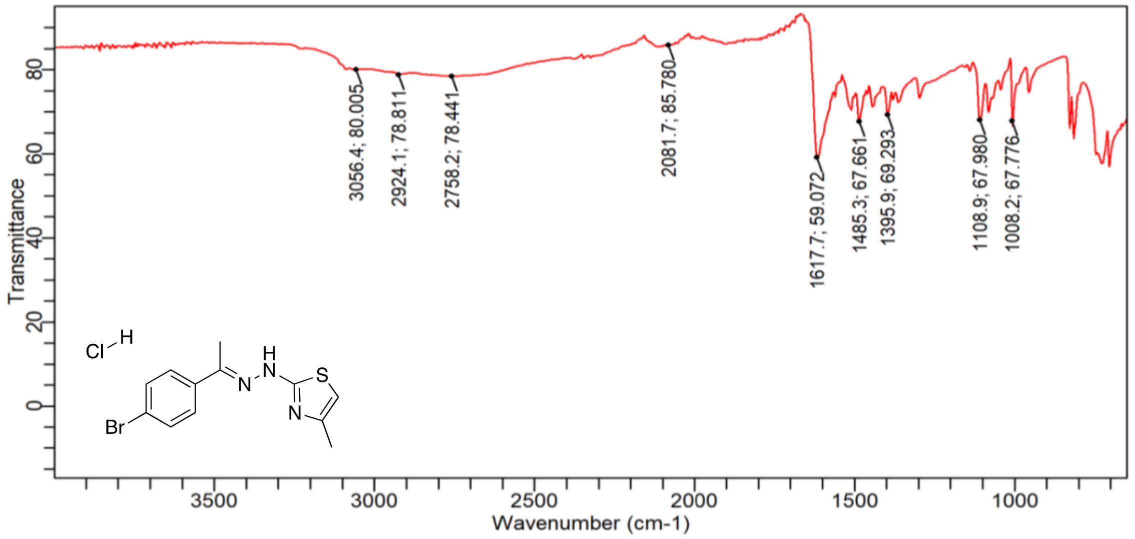

Figure S77. IR (up) and ^{1}H-NMR (down) of compound ***S2f***.

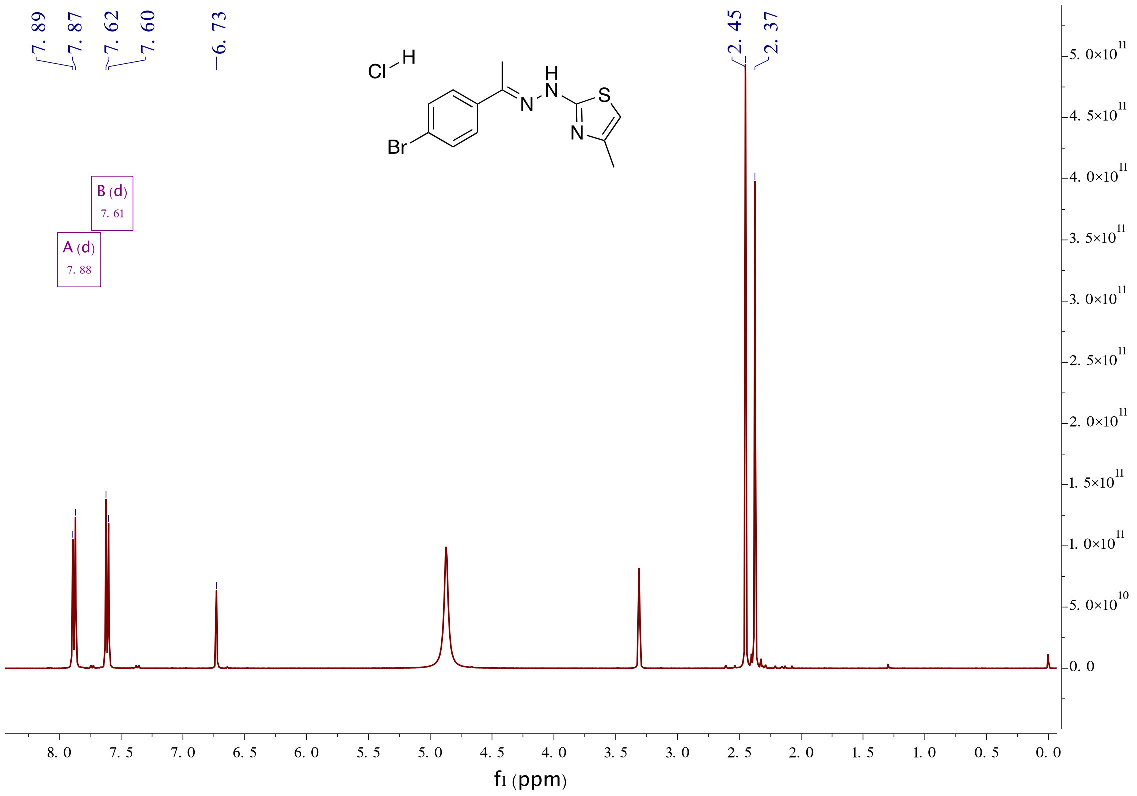

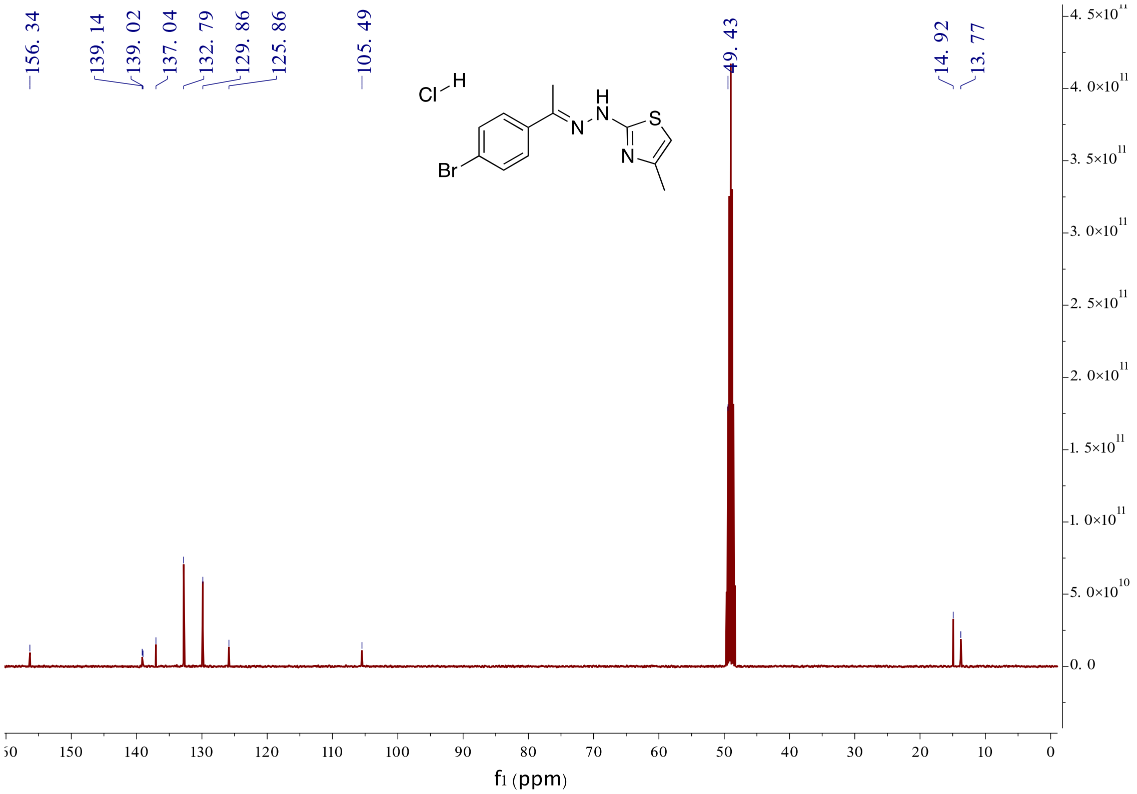

Figure S78. ^{13}C-NMR (up) and qNMR (down) of compound ***S*2f**.

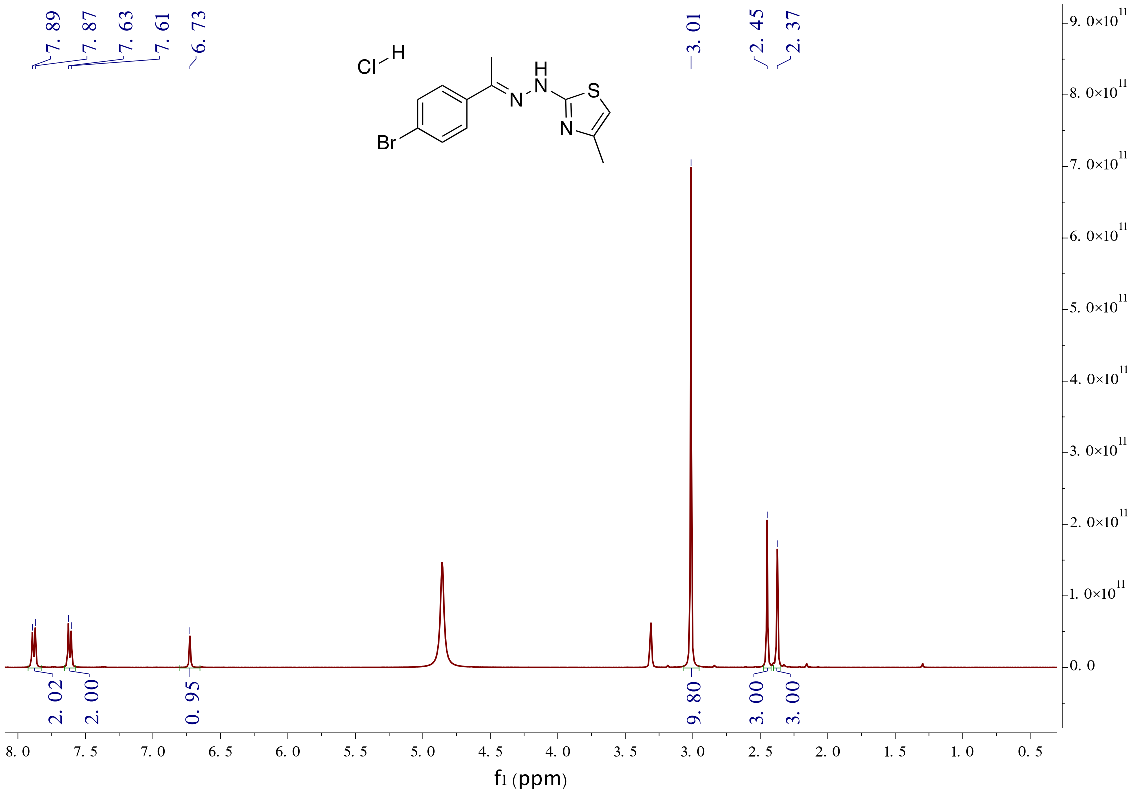

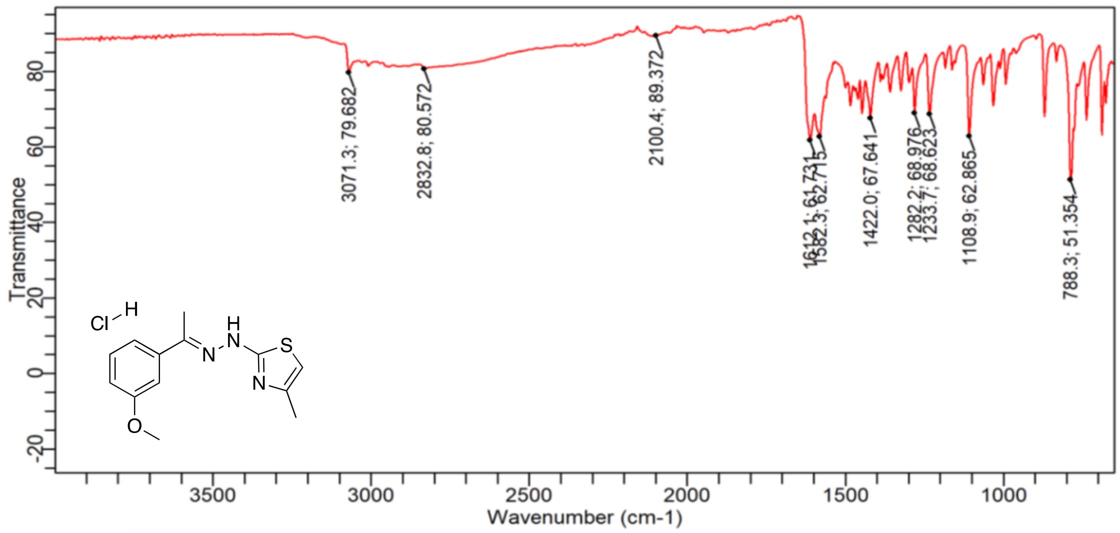

Figure S79. IR (up) and ^{1}H-NMR (down) of compound ***S*2g**.

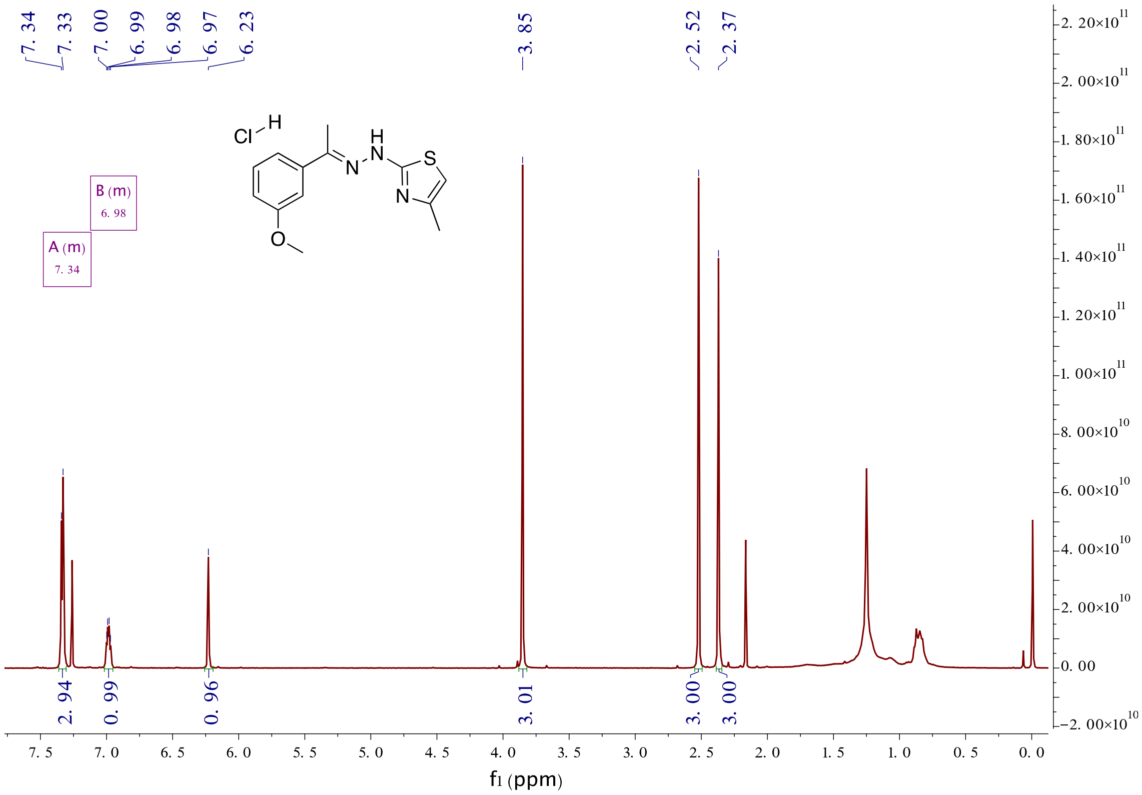

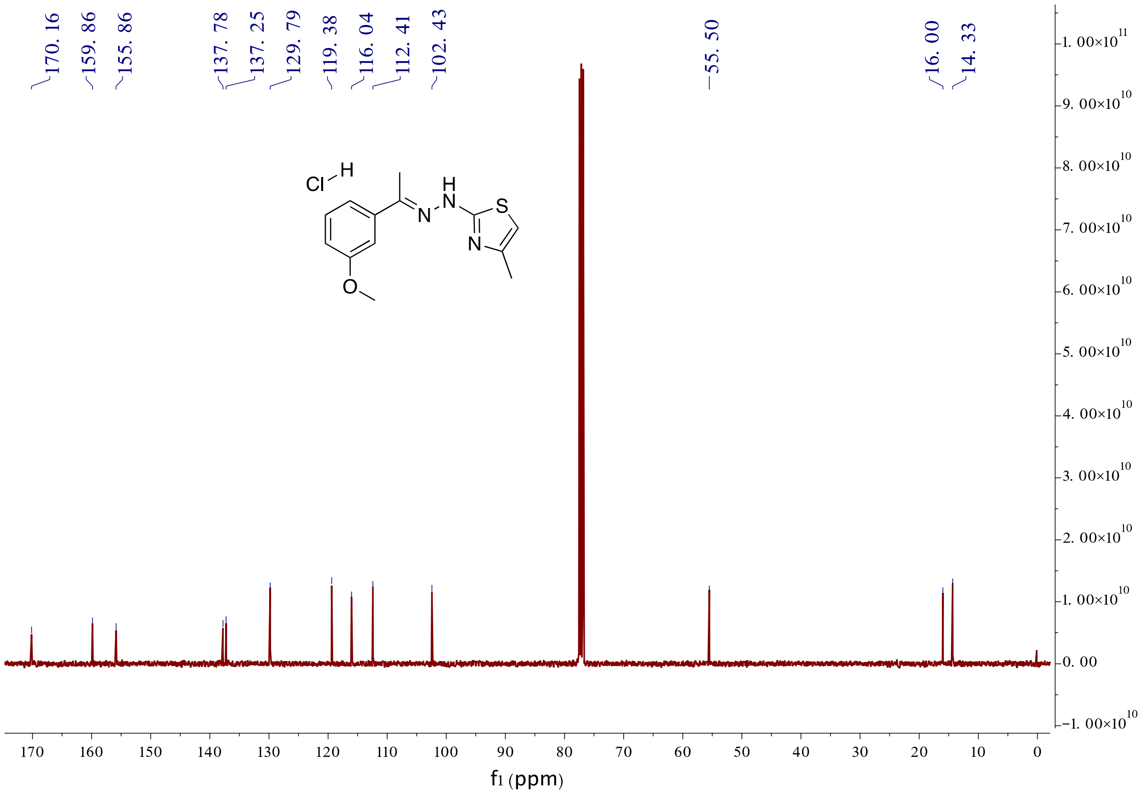

Figure S80. ^{13}C-NMR (up) and qNMR (down) of compound ***S*2g**.

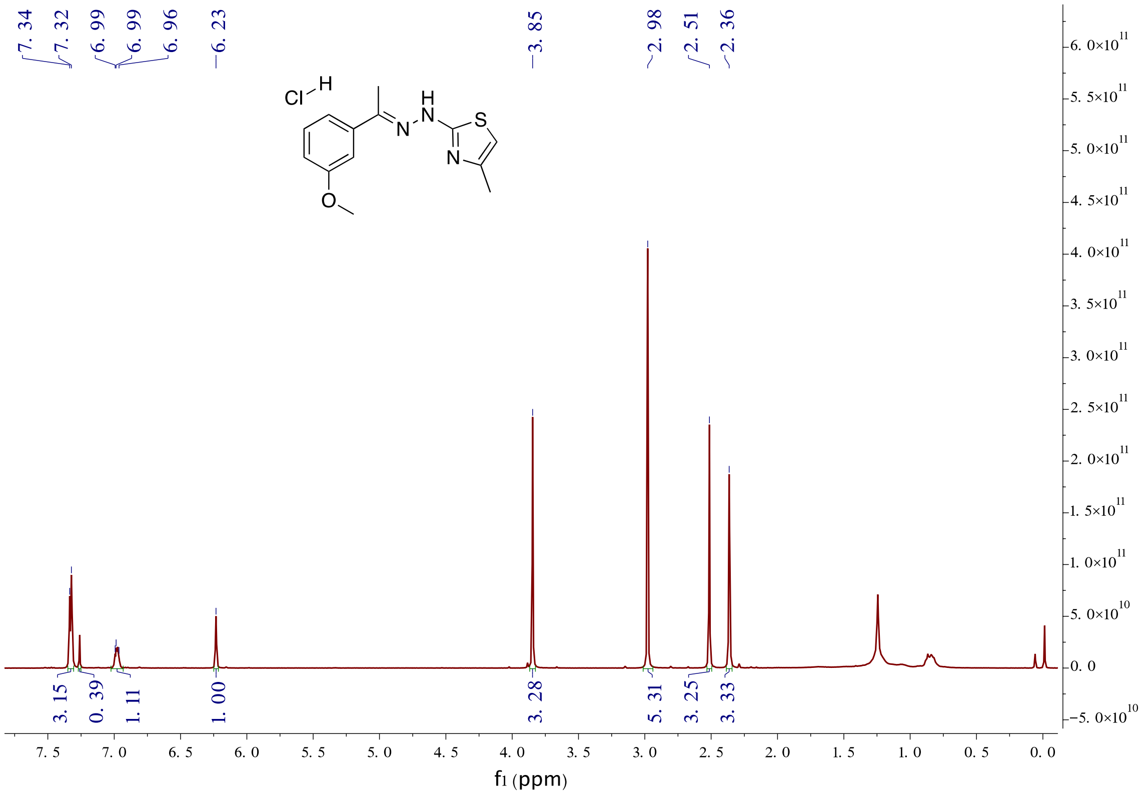

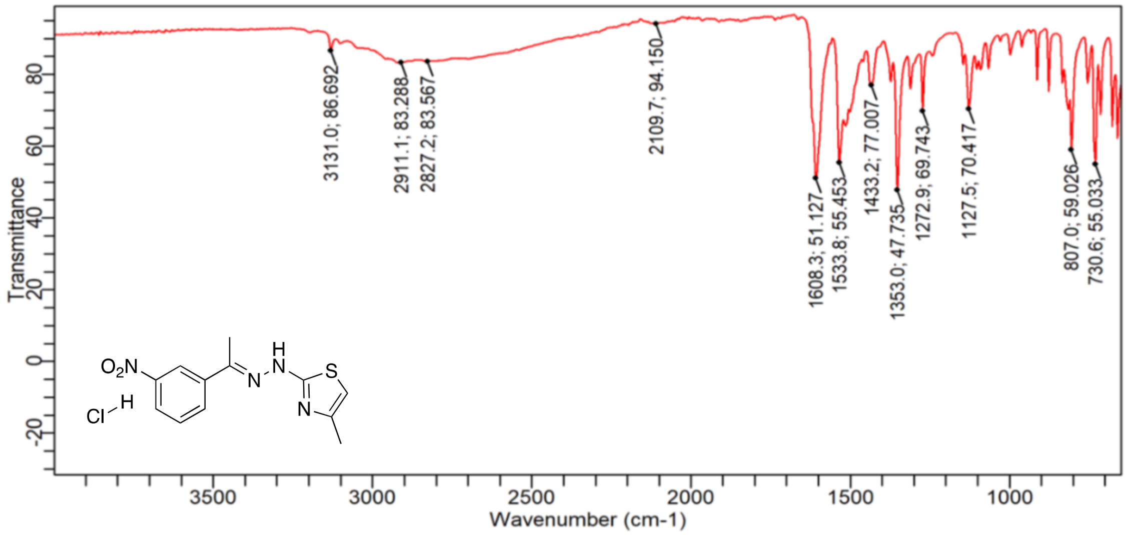

Figure S81. IR (up) and ^{1}H-NMR (down) of compound ***S*2h**.

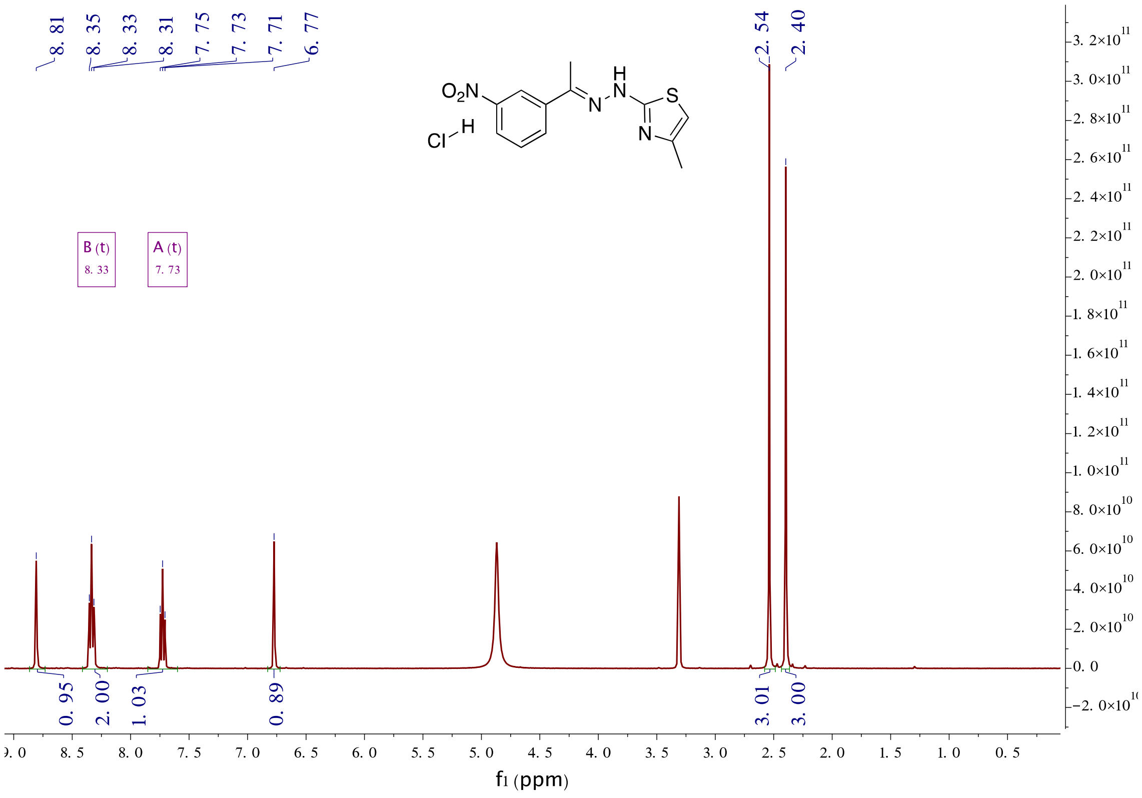

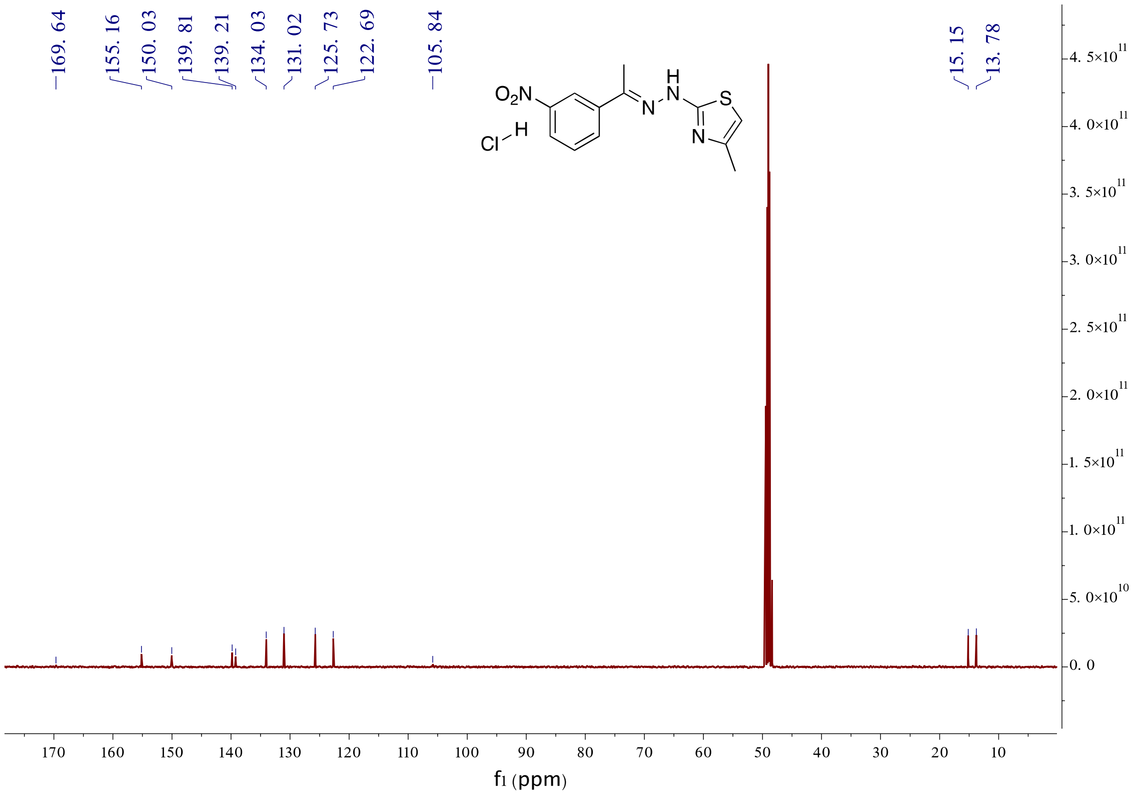

Figure S82. IR (up) and ^{1}H-NMR (down) of compound ***S*2h**.

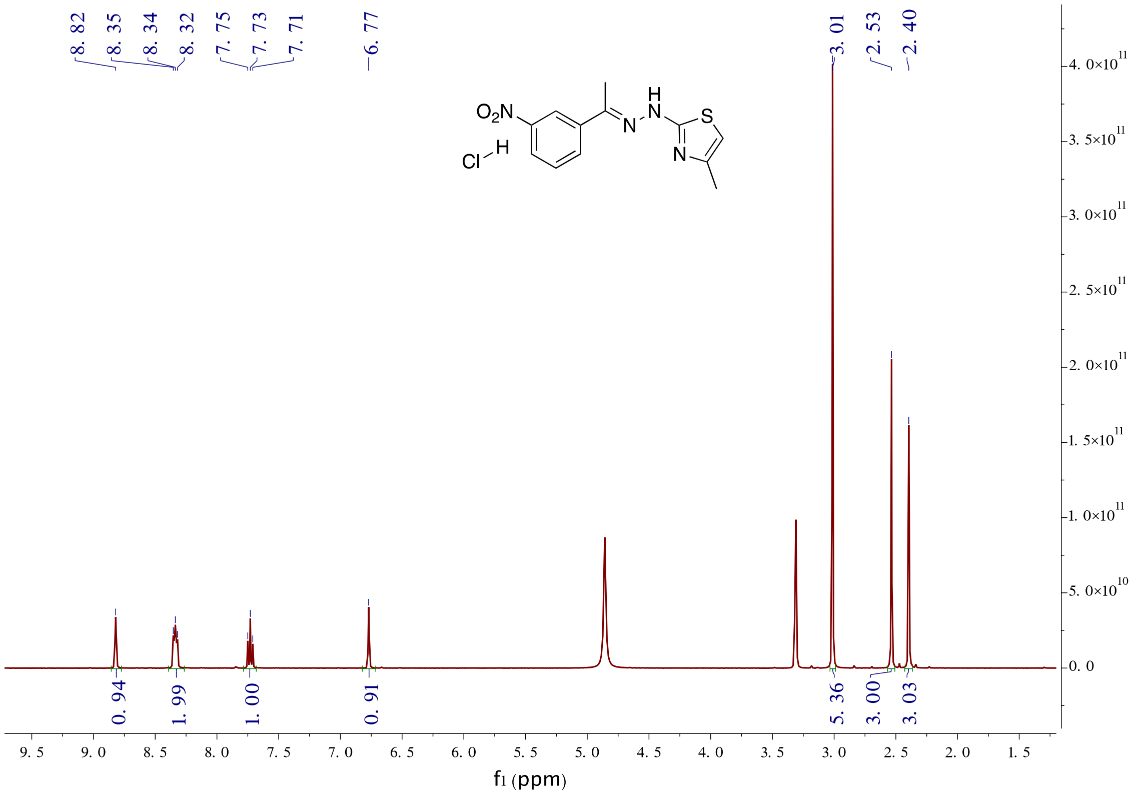

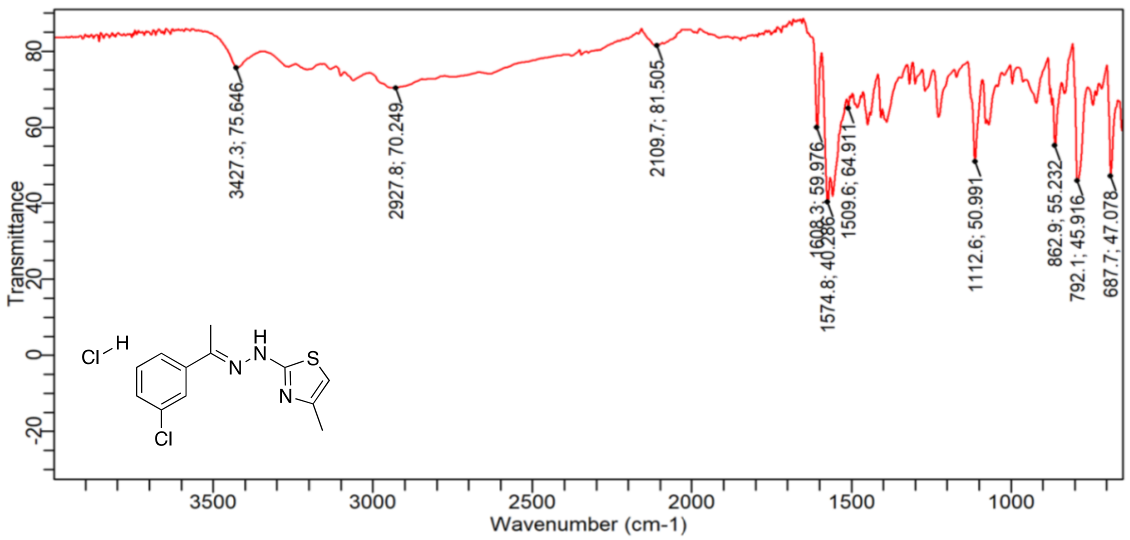

Figure S83. IR (up) and ^{1}H-NMR (down) of compound ***S2i***.

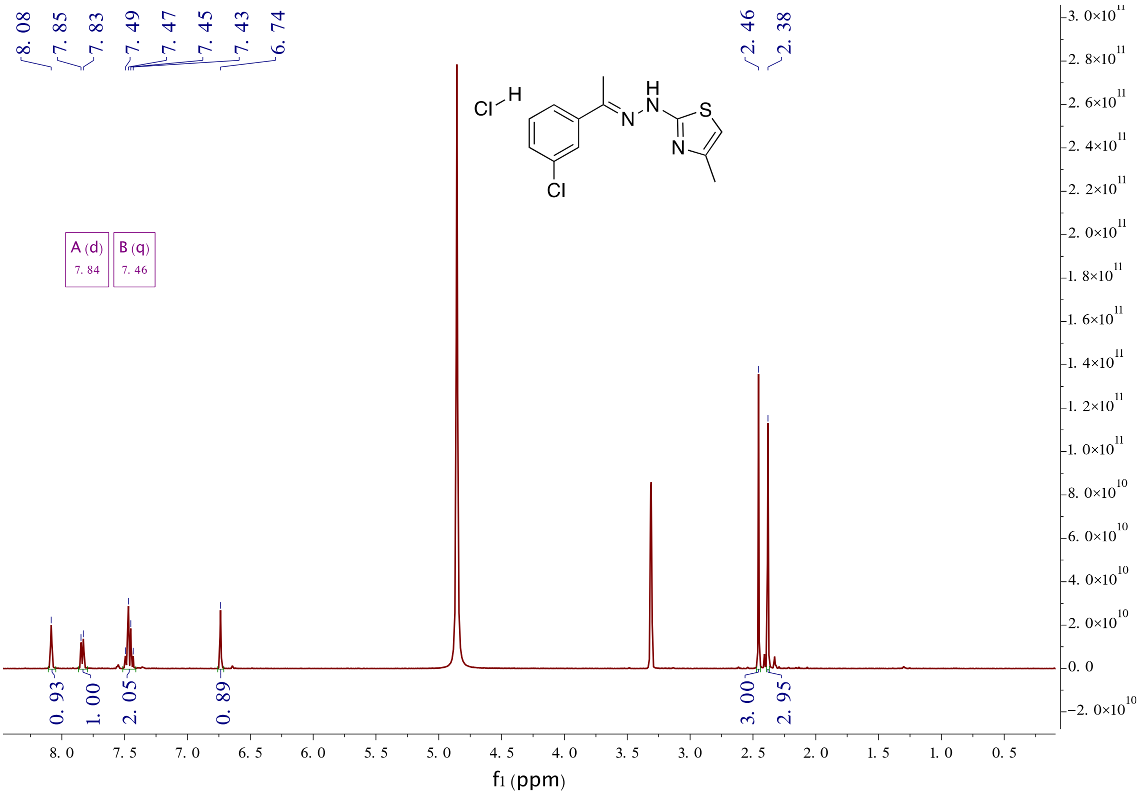

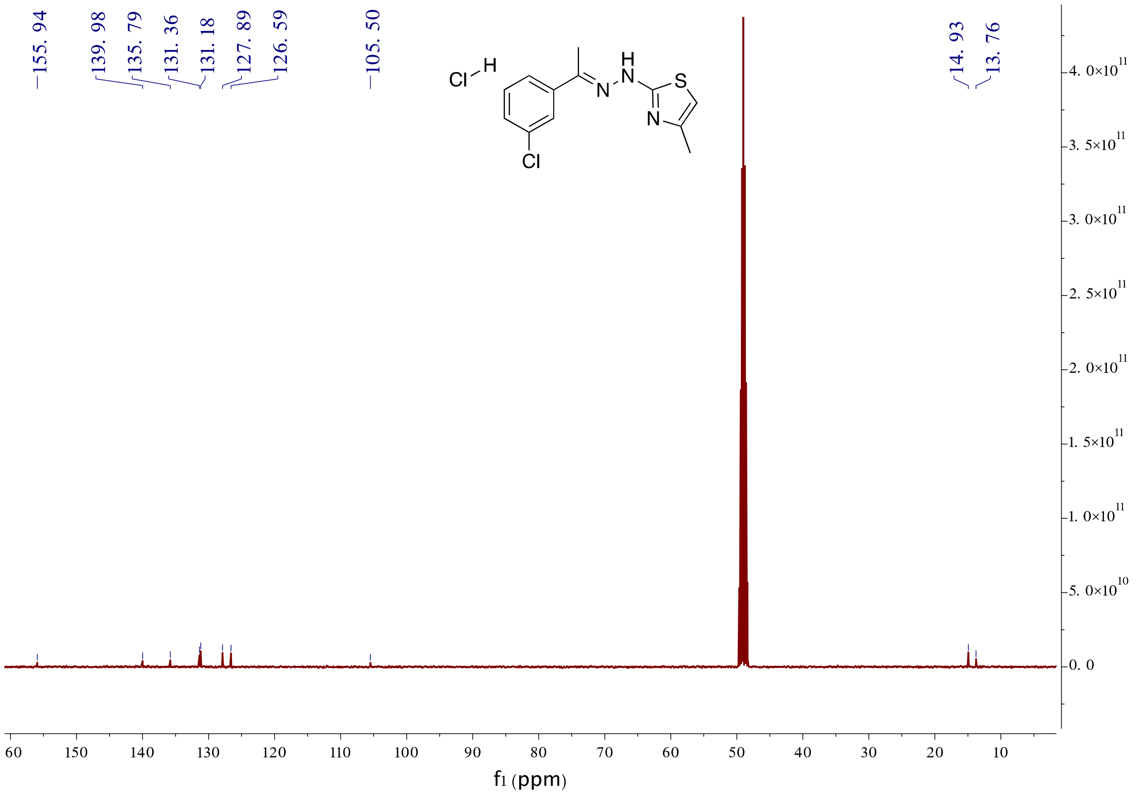

Figure S84. ^{13}C-NMR (up) and qNMR (down) of compound ***S2i***.

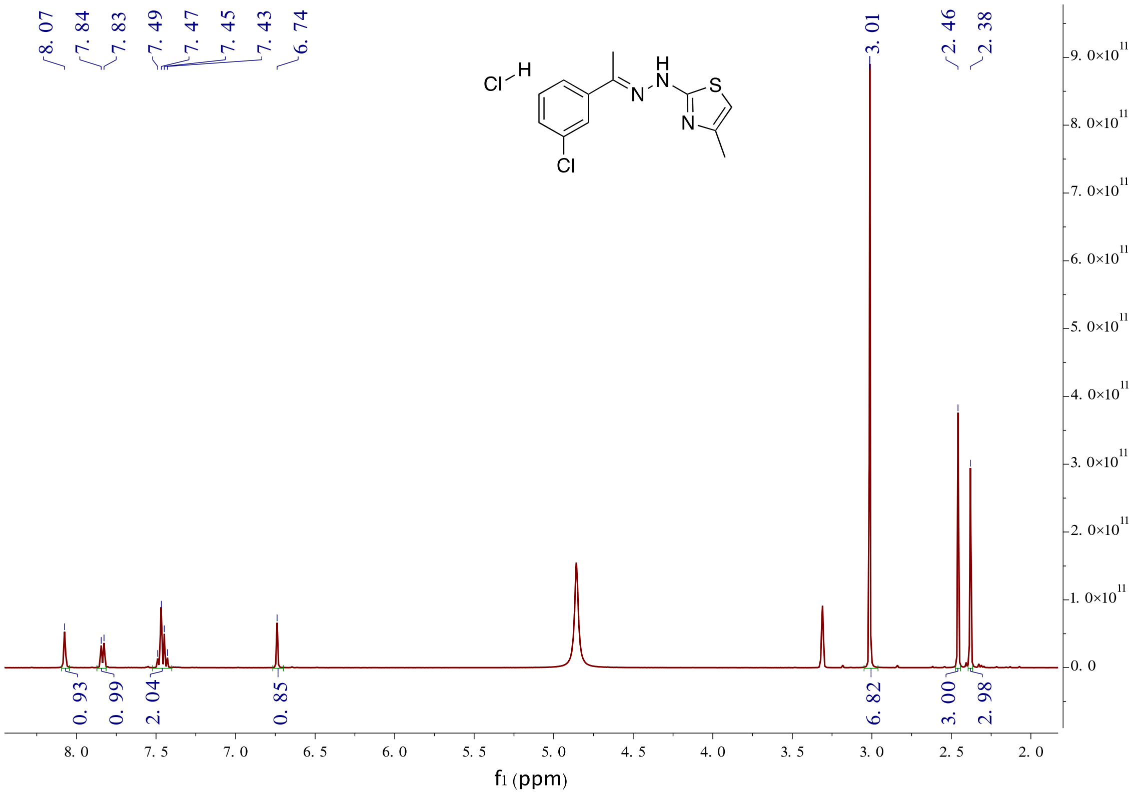

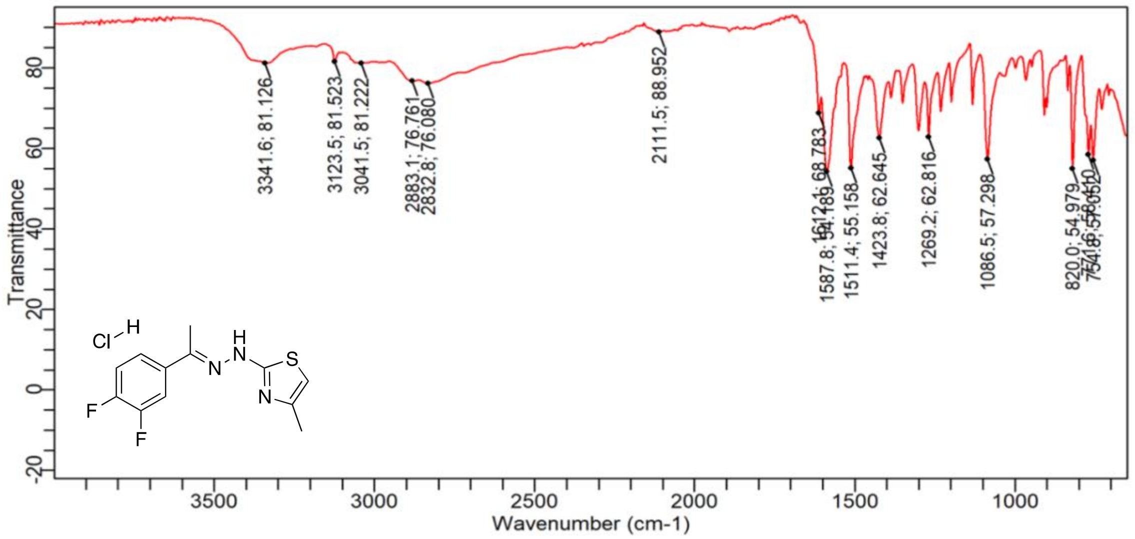

Figure S85. IR (up) and ^{1}H-NMR (down) of compound ***S*2j**.

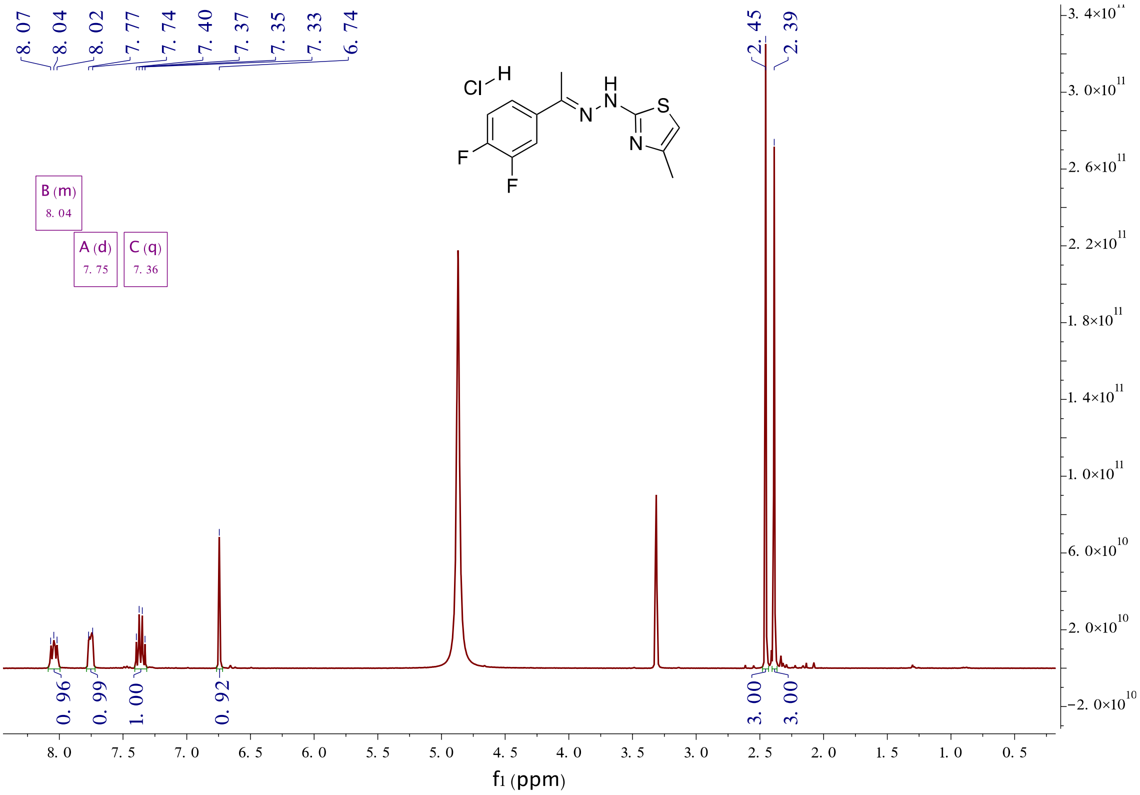

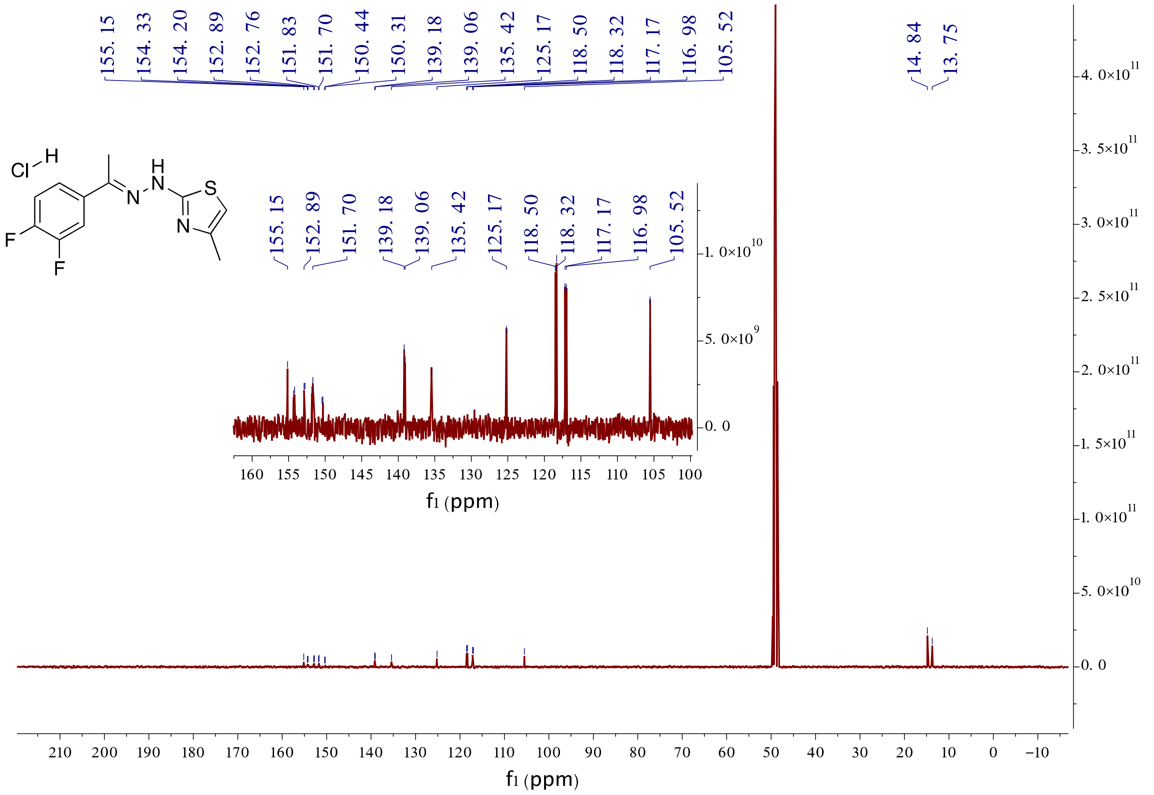

Figure S86. ^{13}C-NMR (up) and qNMR (down) of compound ***S*2j**.

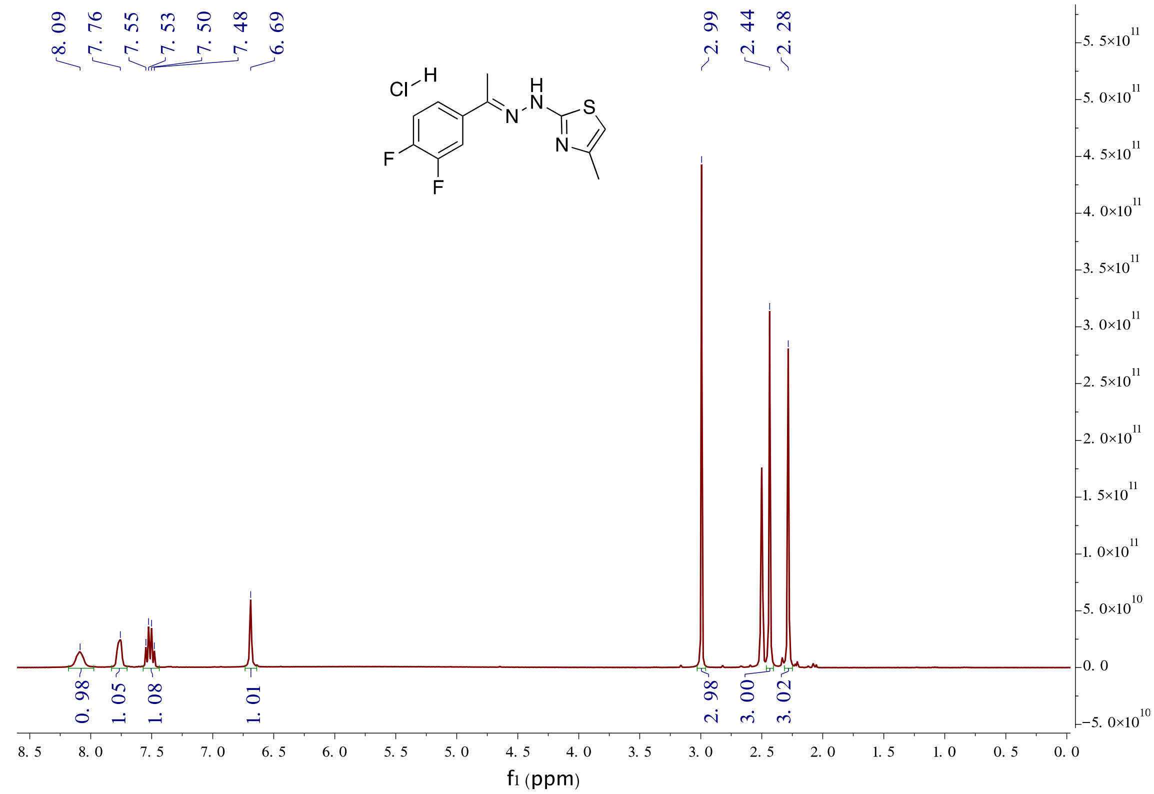

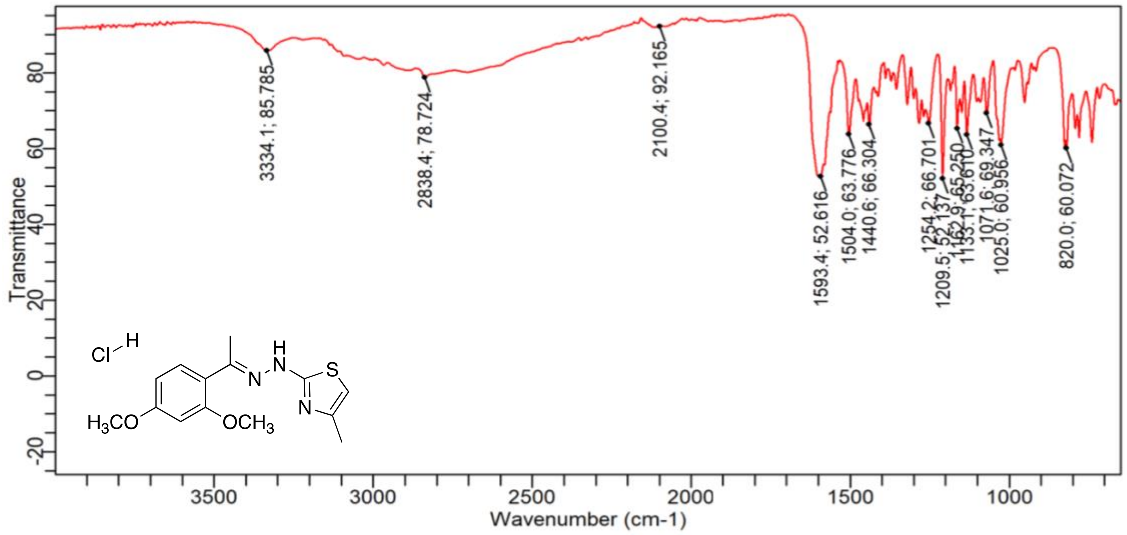

Figure S87. IR (up) and ^{1}H-NMR (down) of compound ***S2k***.

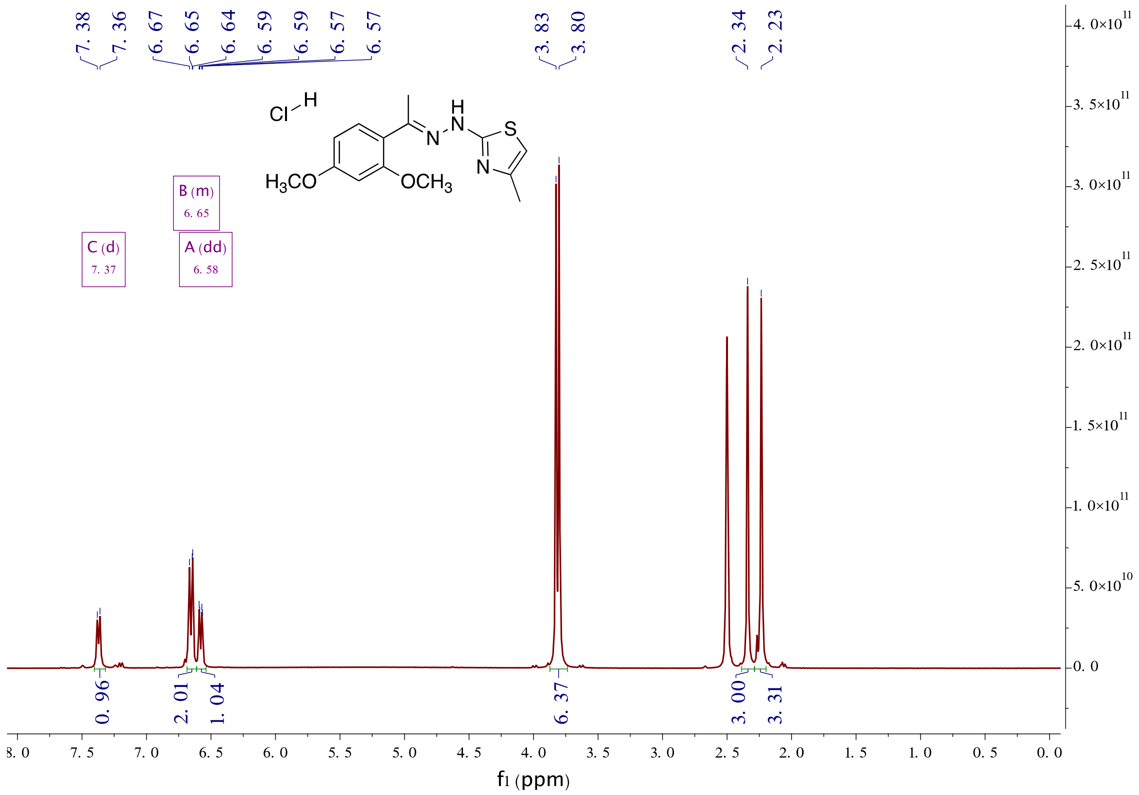

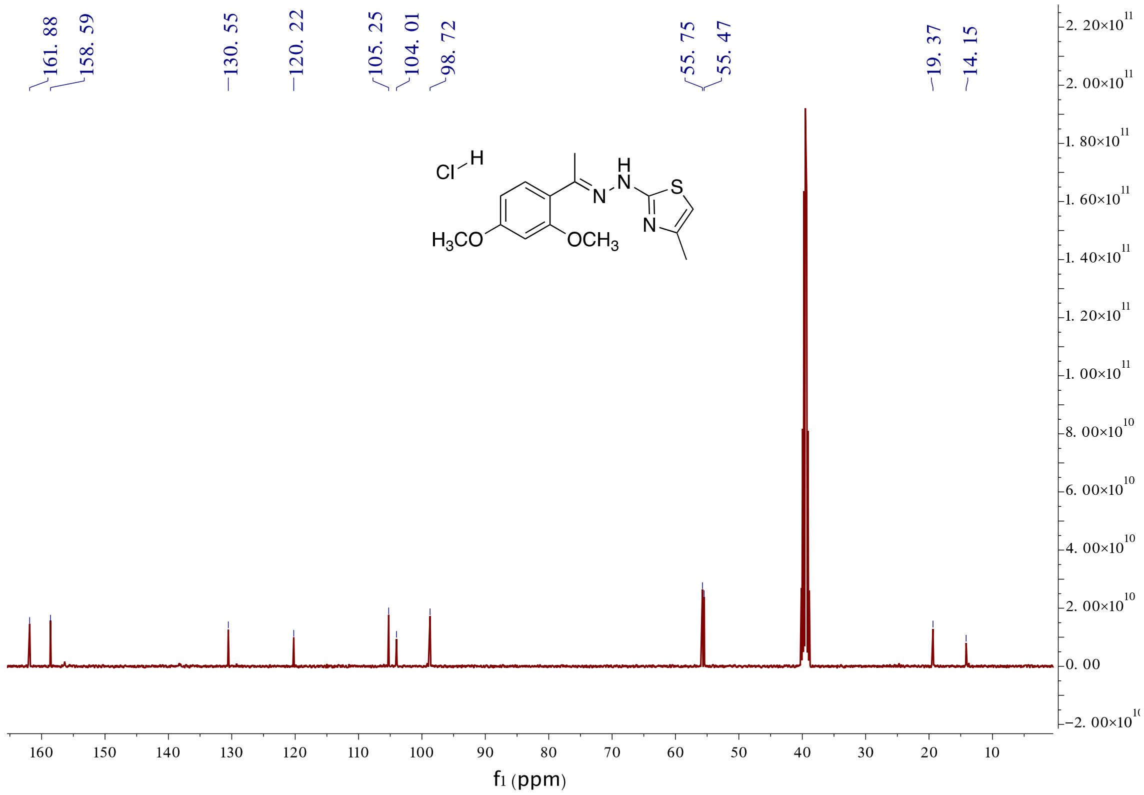

Figure S88. ^{13}C-NMR (up) and qNMR (down) of compound ***S*2k**.

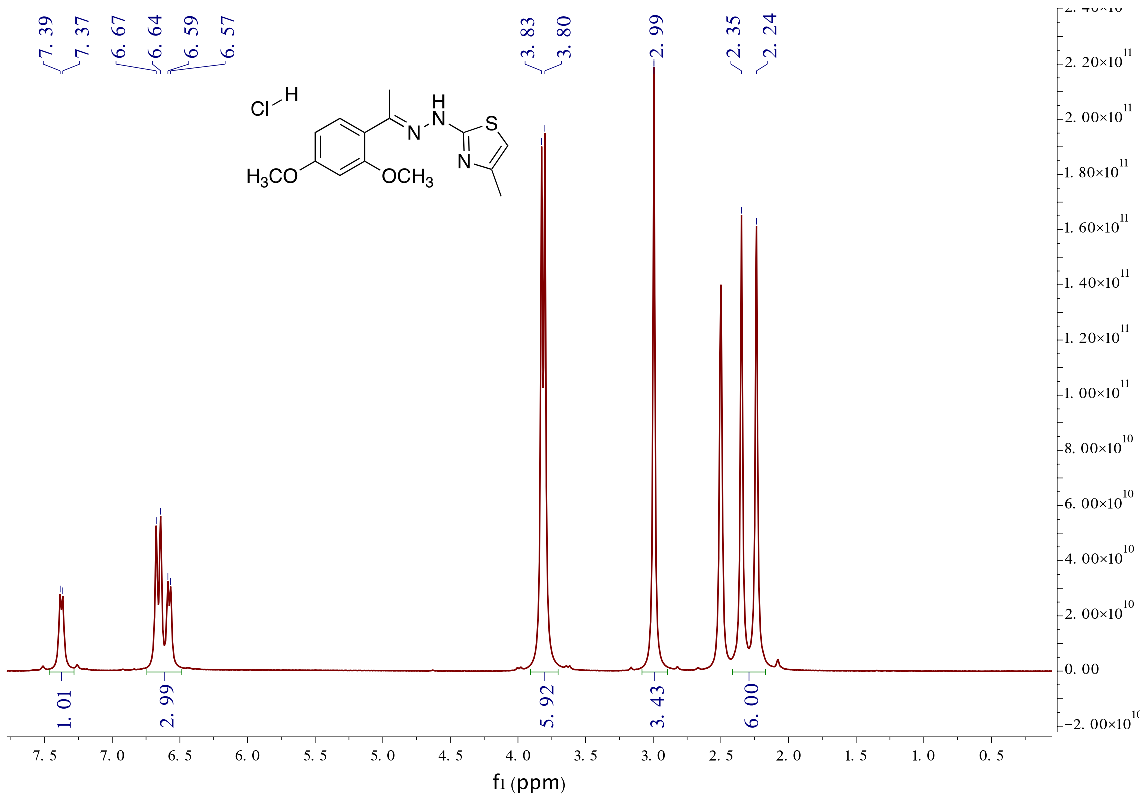

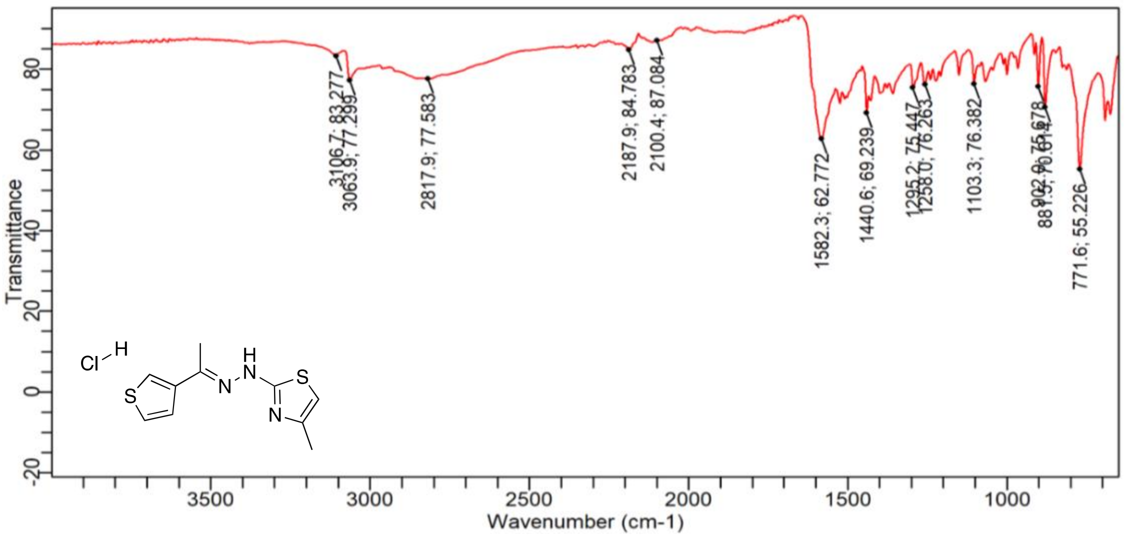

Figure S89. IR (up) and ^{1}H-NMR (down) of compound ***S*2l**.

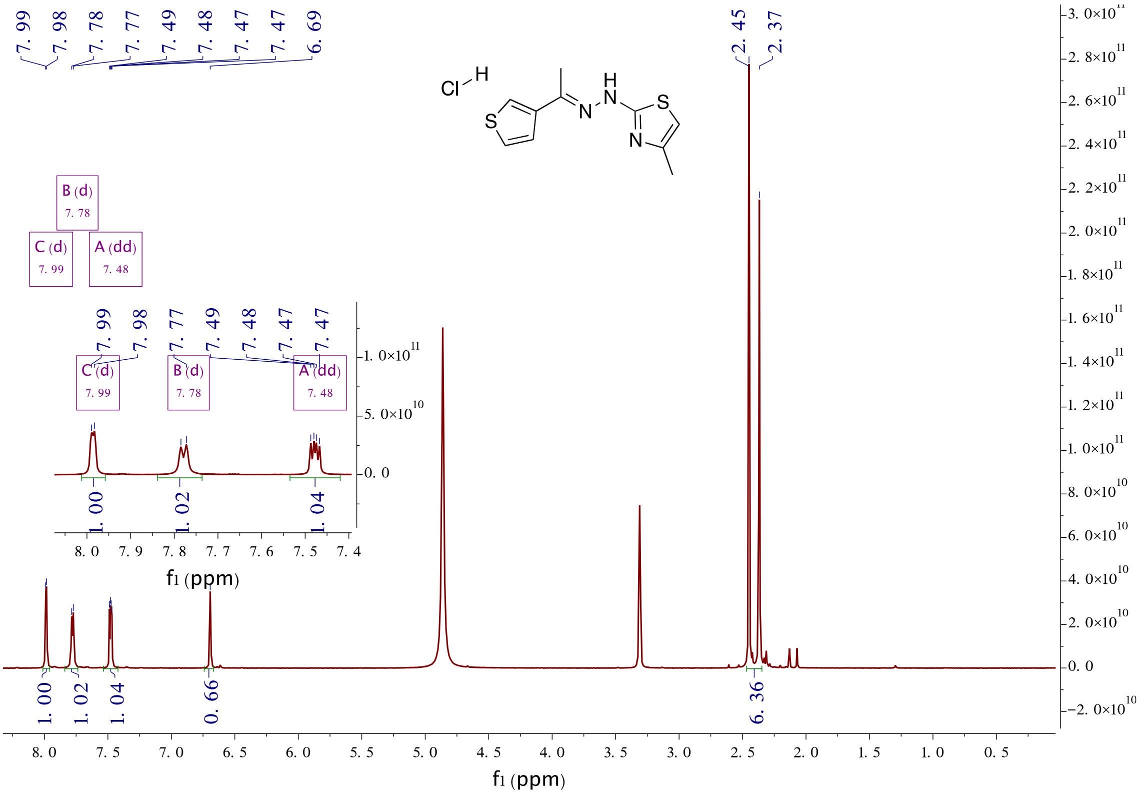

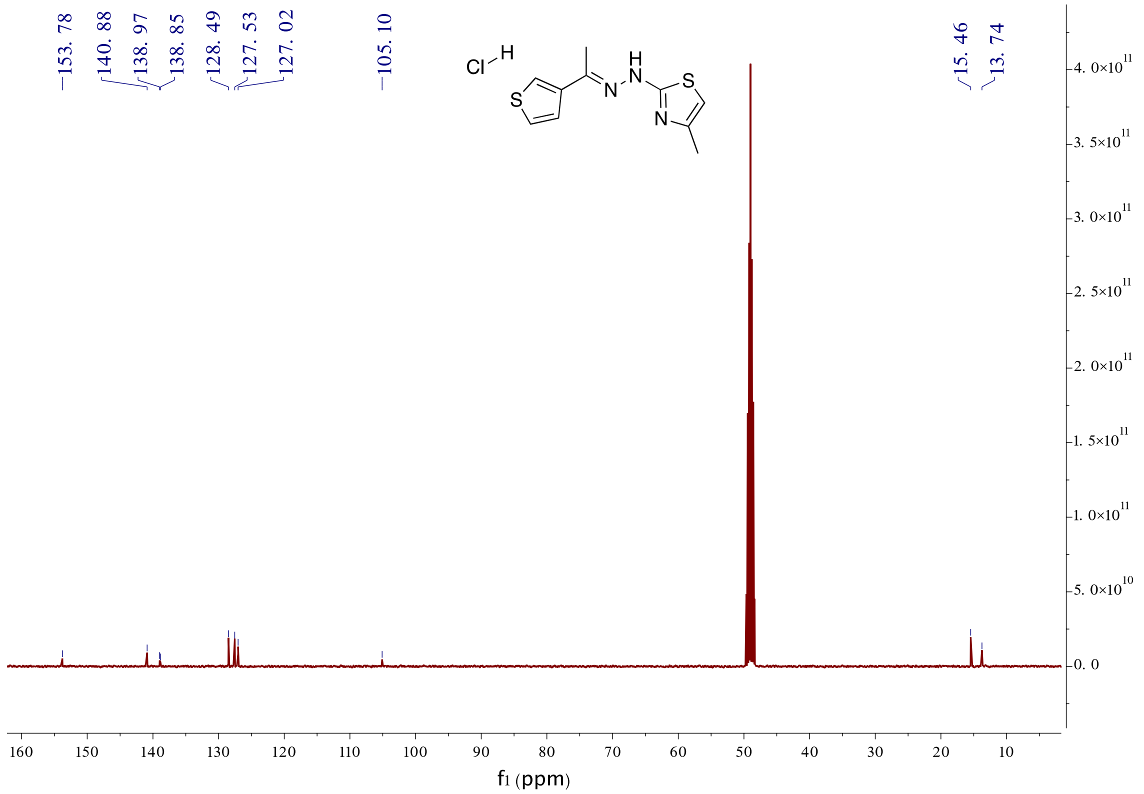

Figure S90. ^{13}C-NMR (up) and qNMR (down) of compound ***S*2l**.

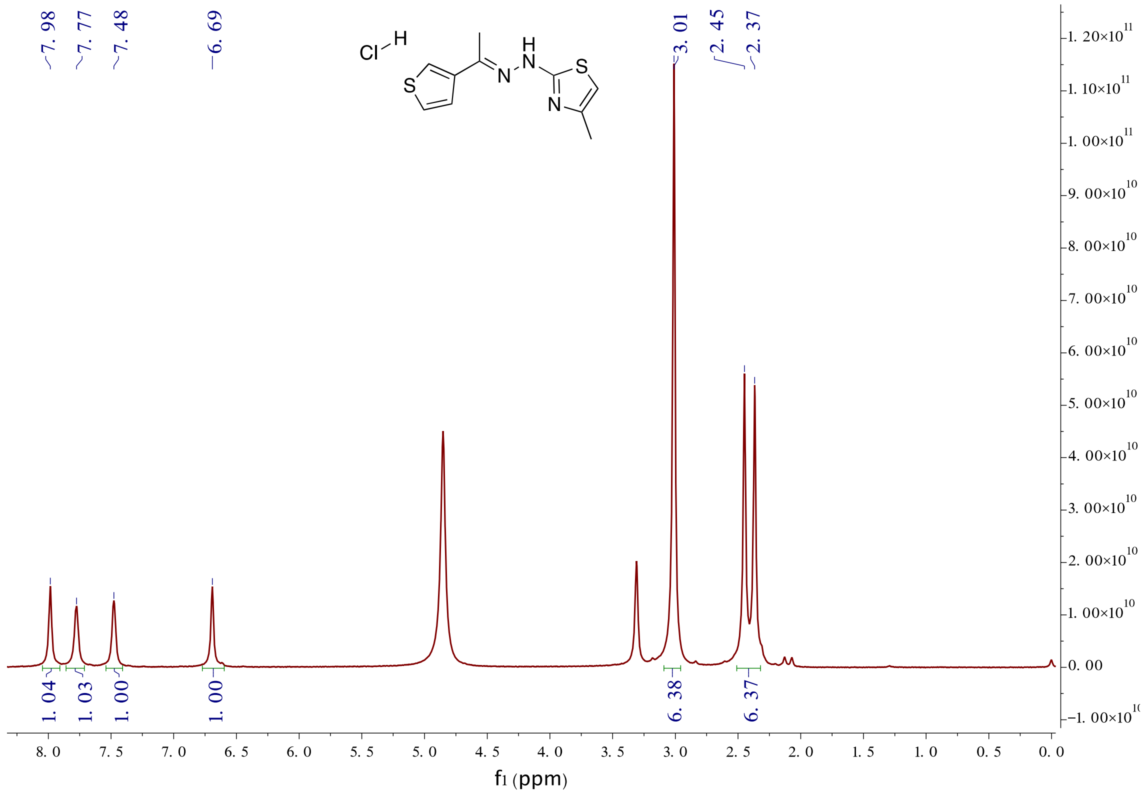

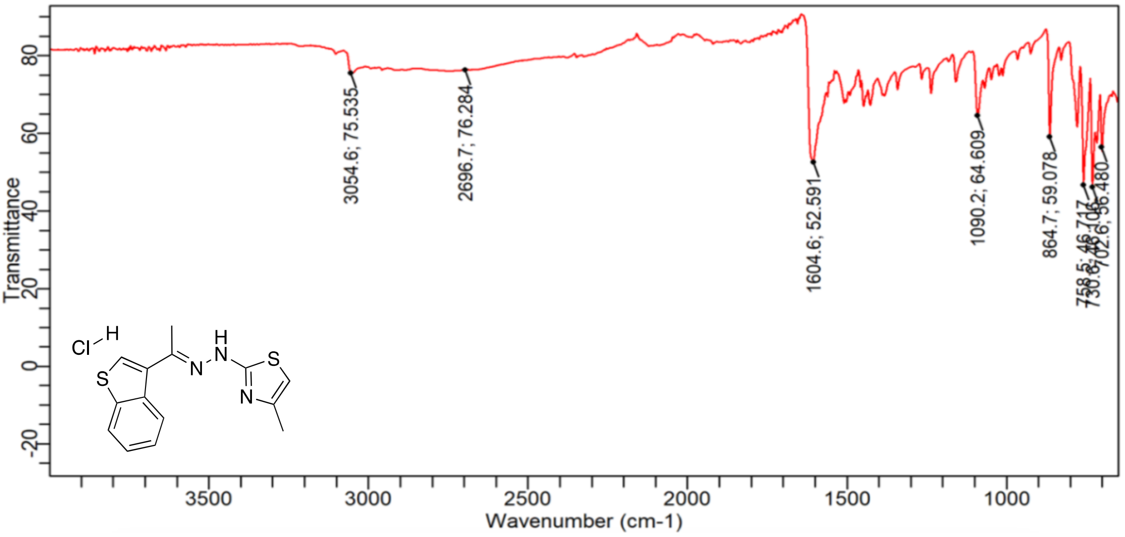

Figure S91. IR (up) and ^{1}H-NMR (down) of compound ***S2o***.

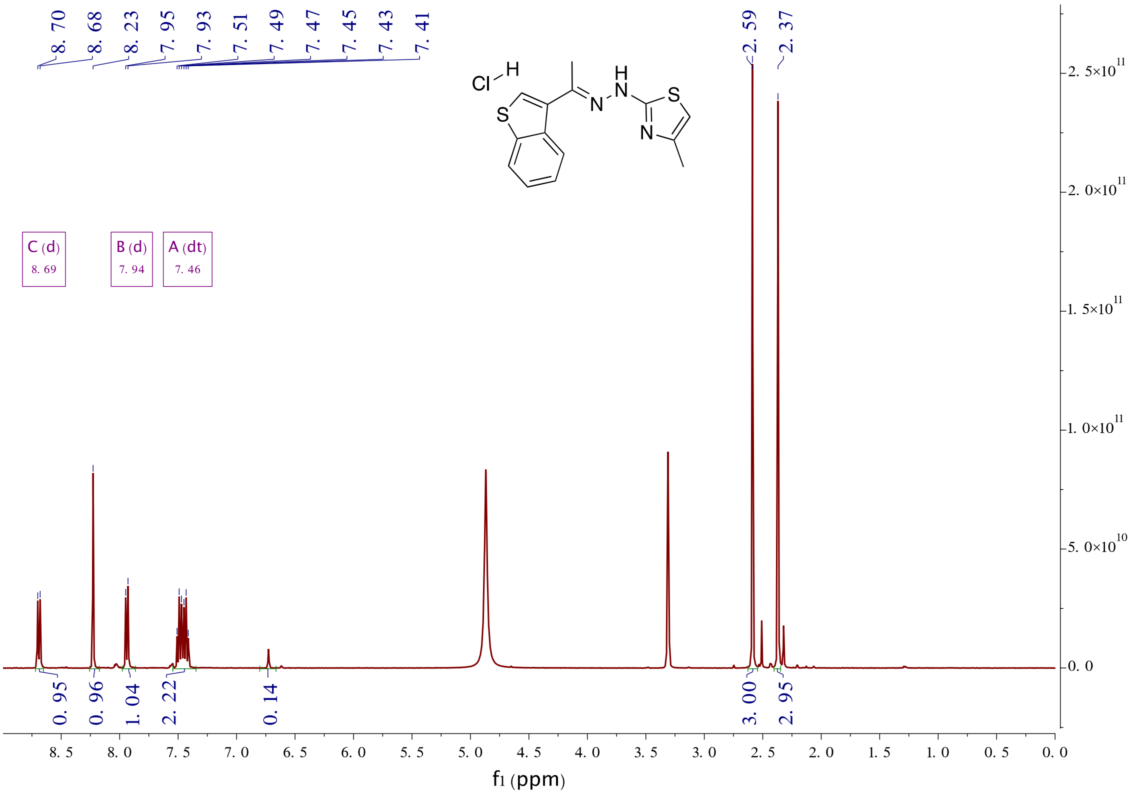

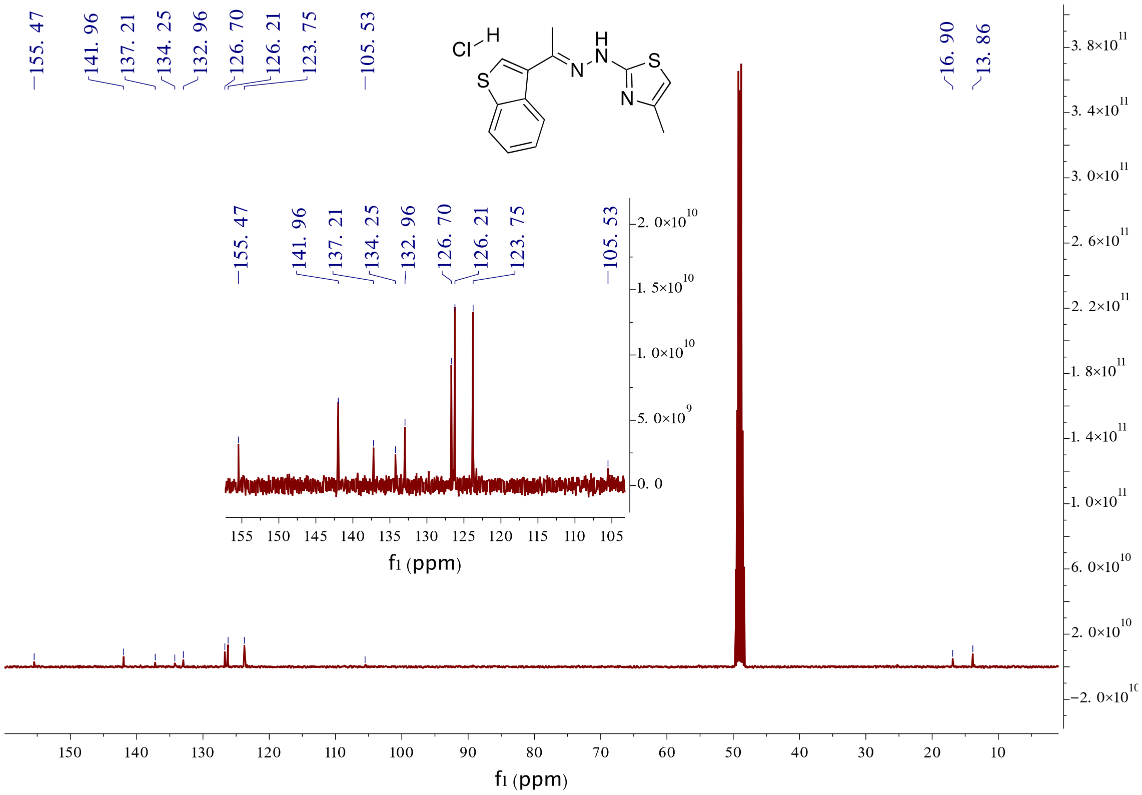

Figure S92. ^{13}C-NMR (up) and qNMR (down) of compound ***S*2o**.

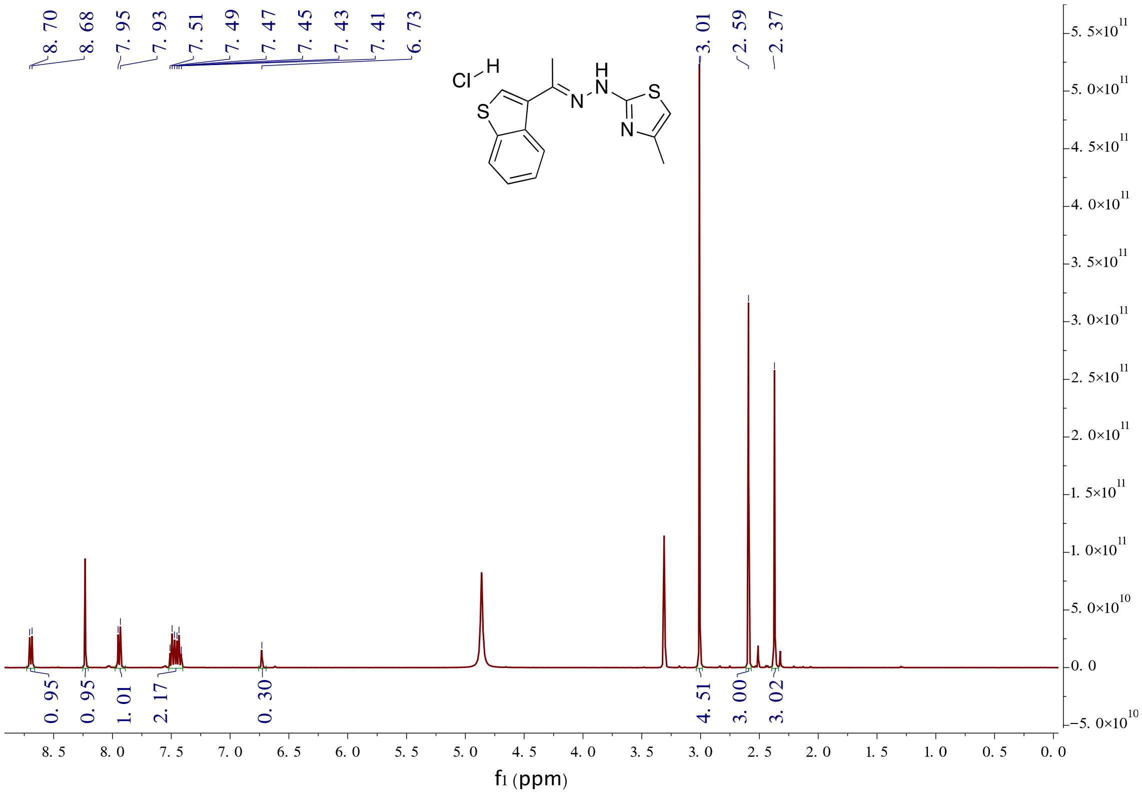

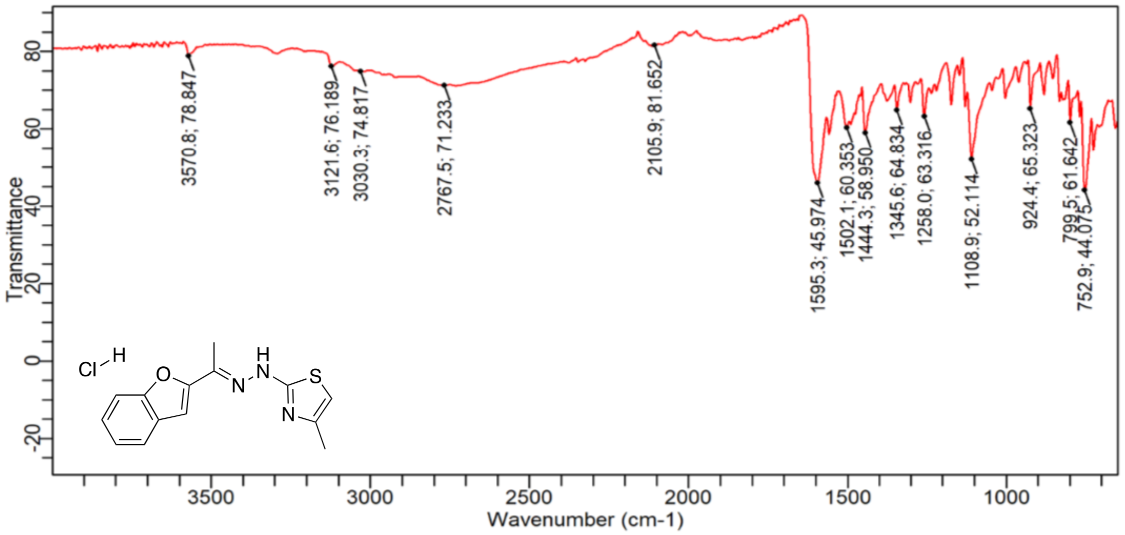

Figure S93. IR (up) and ^{1}H-NMR (down) of compound ***S*2p**.

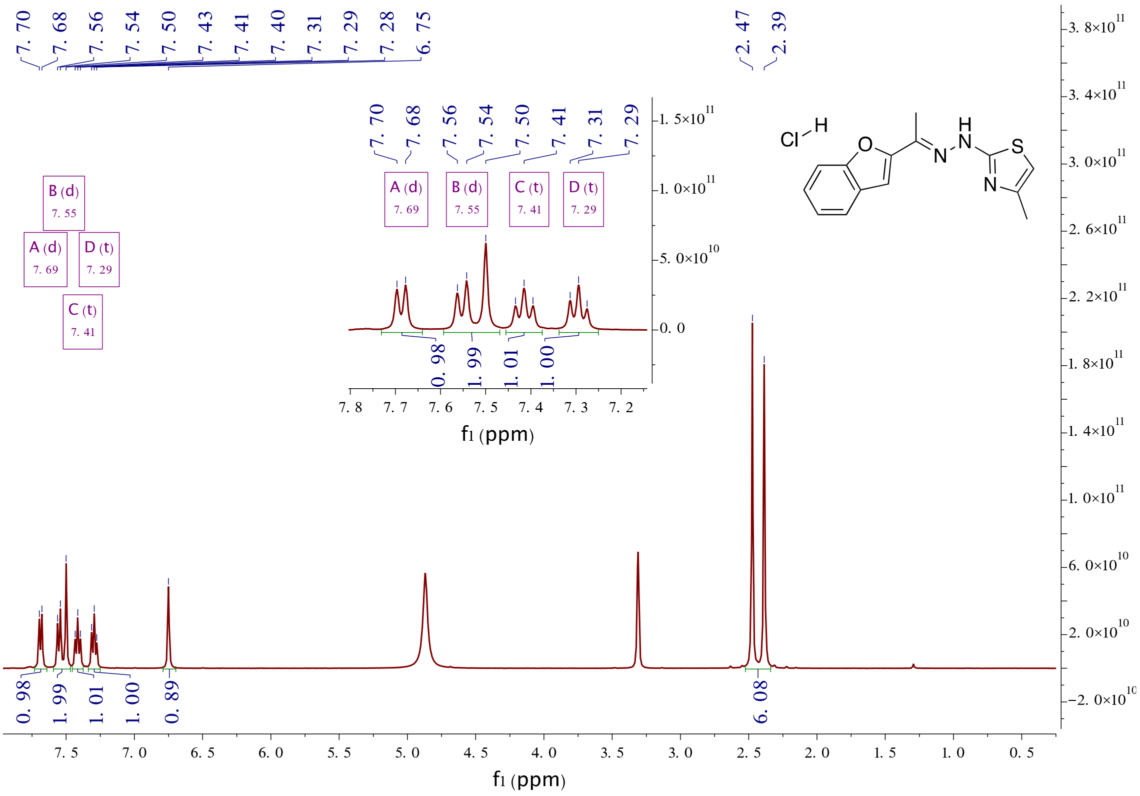

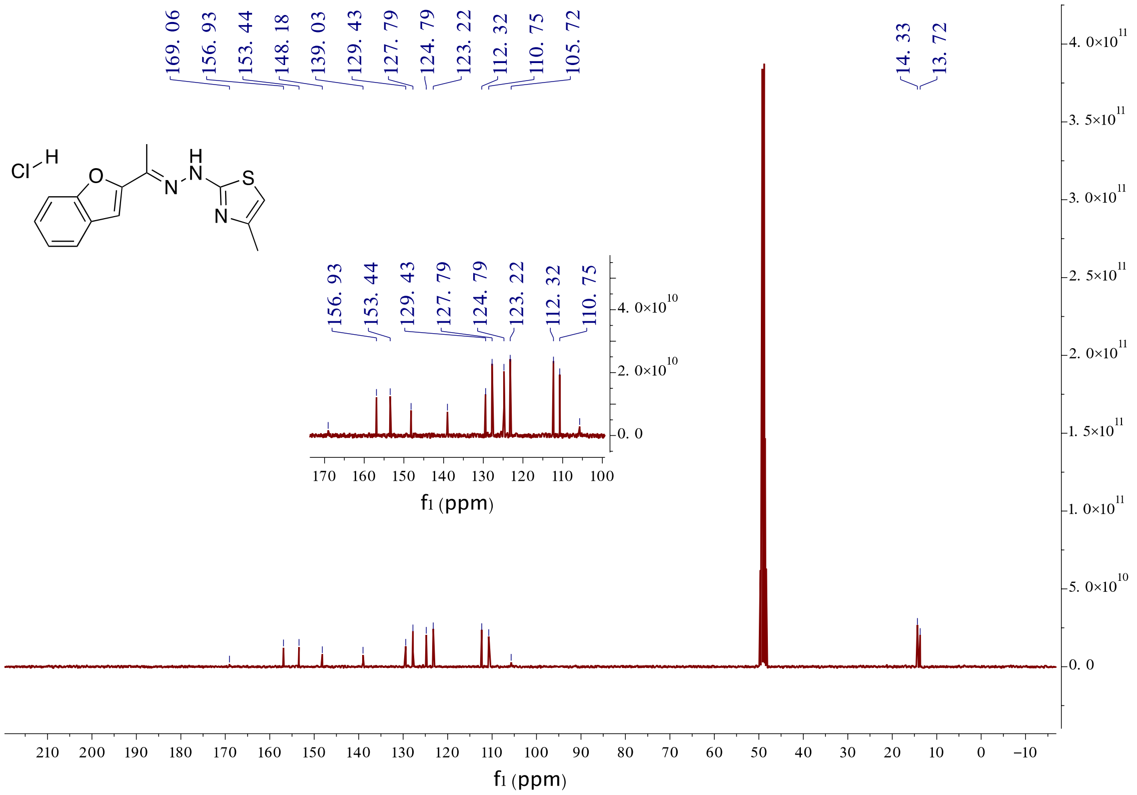

Figure S94. ^{13}C-NMR (up) and qNMR (down) of compound ***S*2p**.

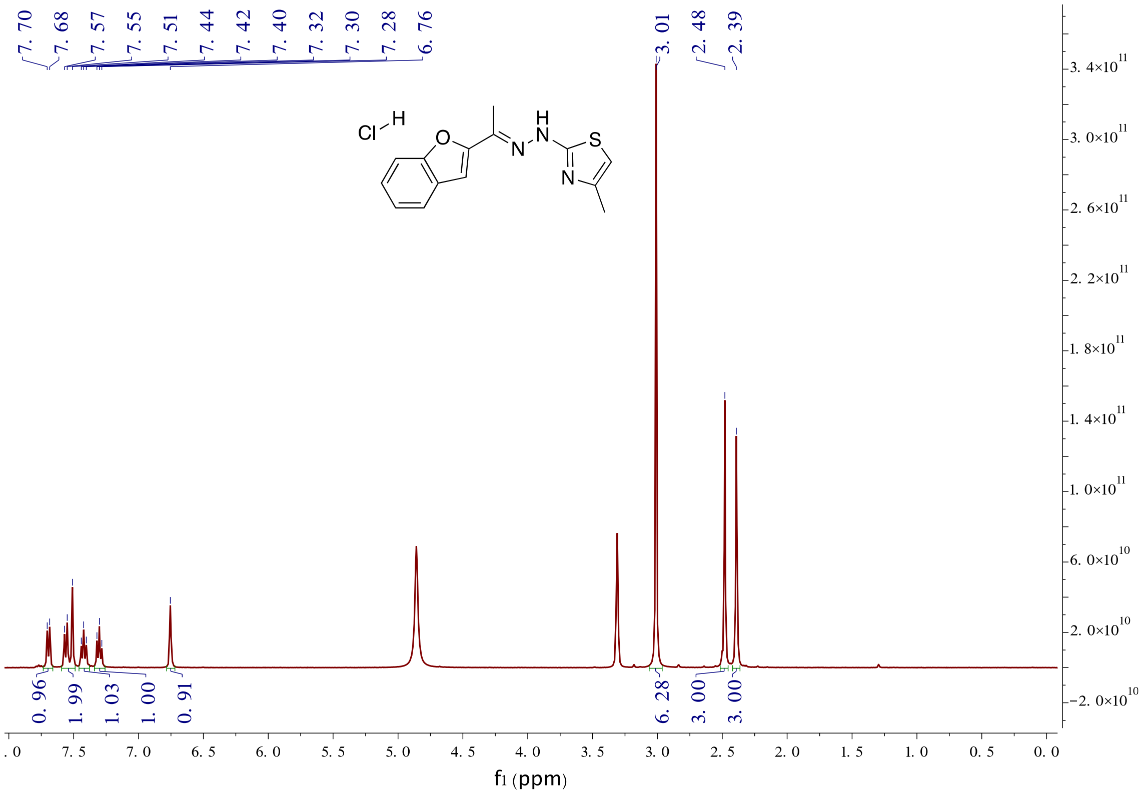

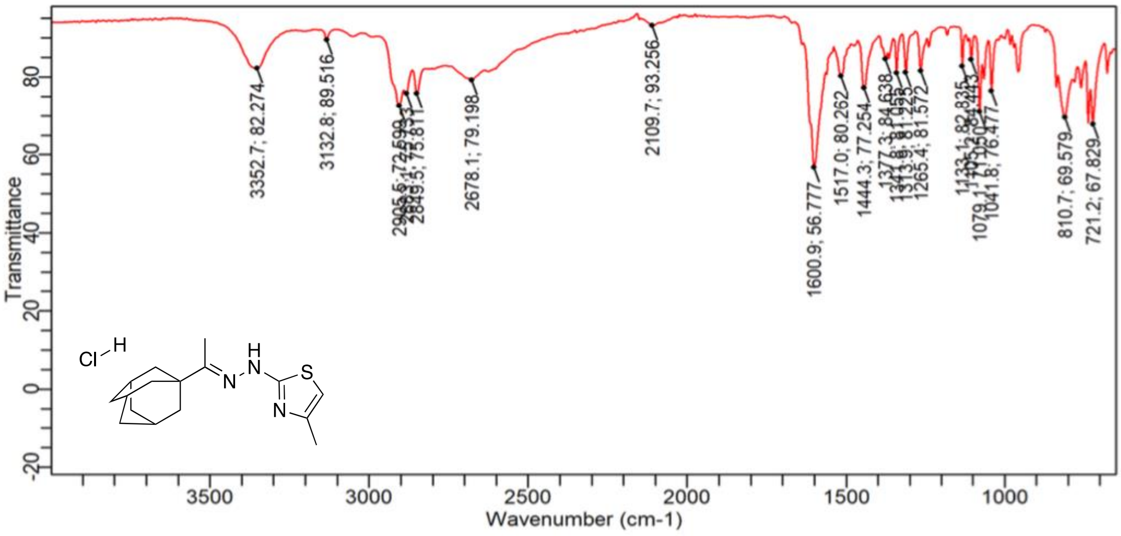

Figure S95. IR (up) and ^{1}H-NMR (down) of compound ***S*2q**.

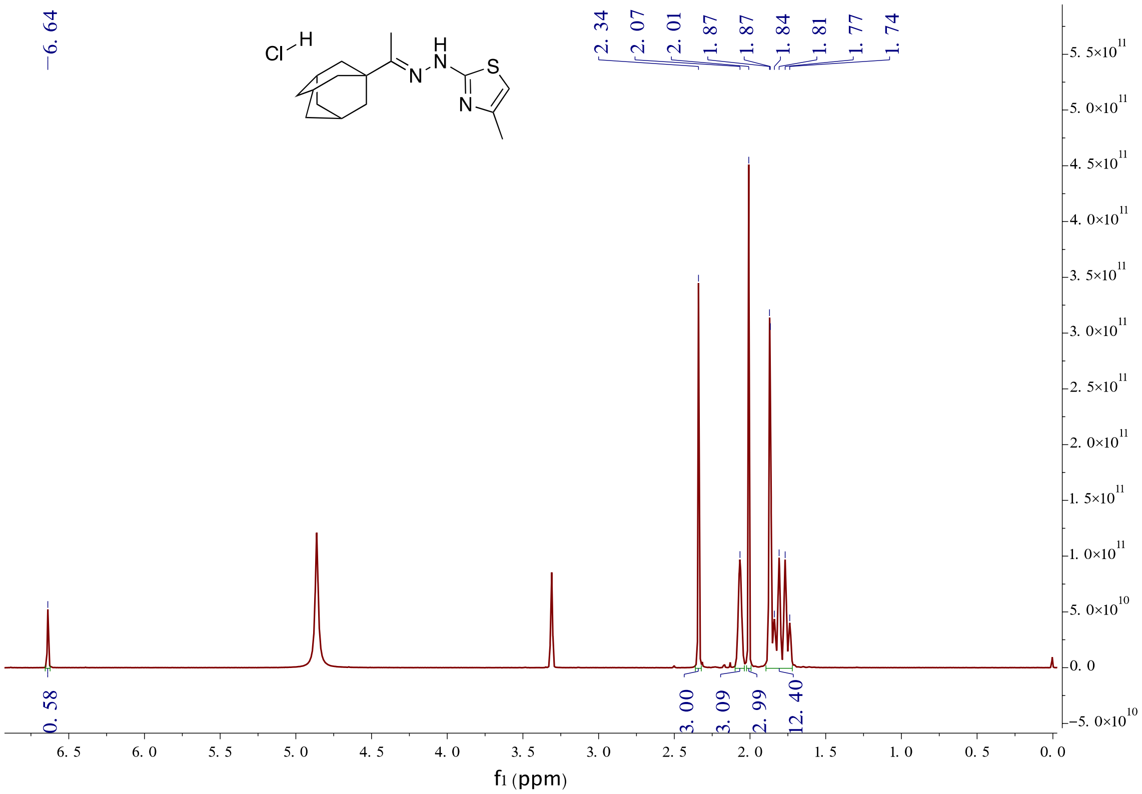

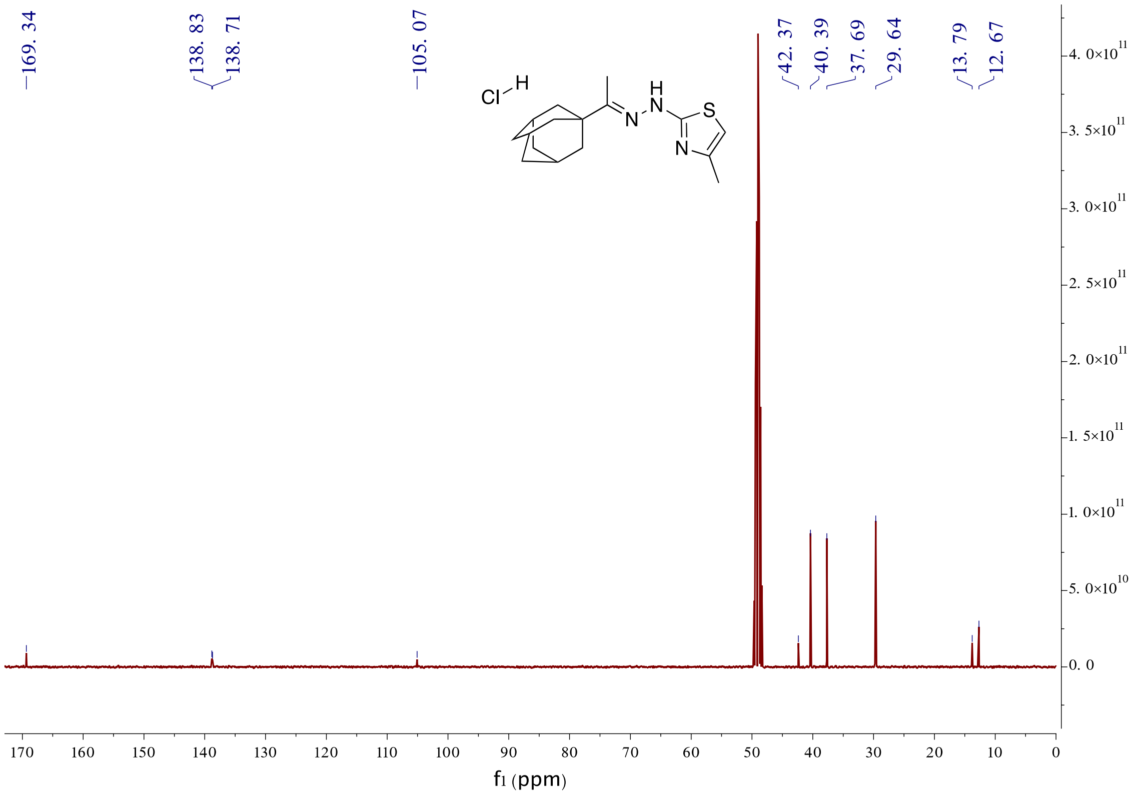

Figure S96. ^{13}C-NMR (up) and qNMR (down) of compound ***S*2q**.

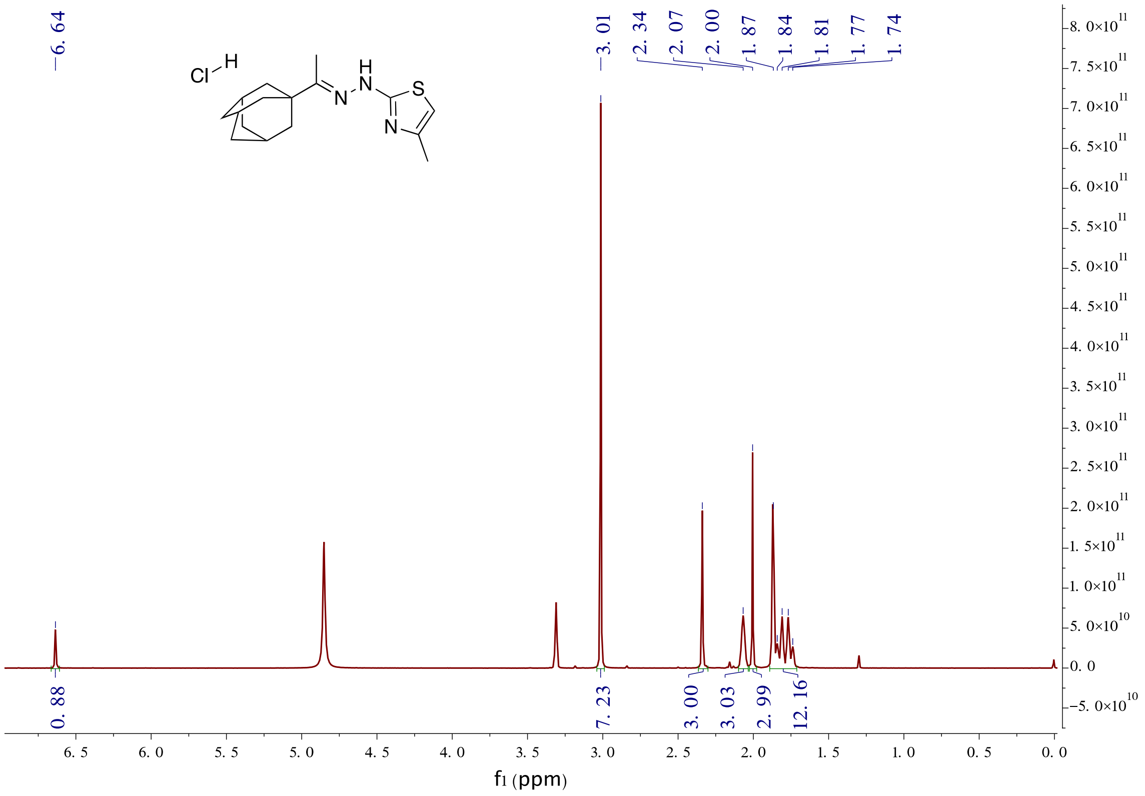

d. *Se*2 series

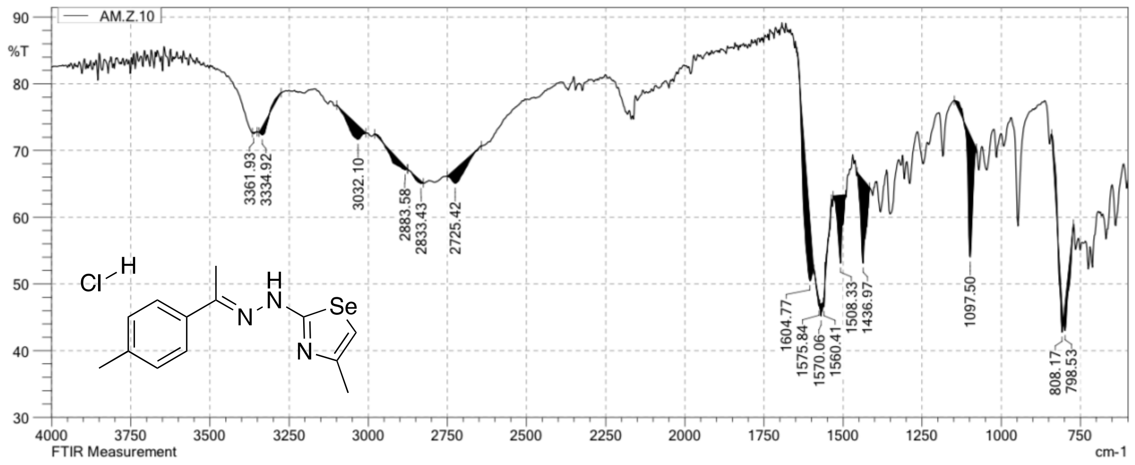

Figure S97. IR (up) and ^{1}H-NMR (down) of compound ***Se*2a**.

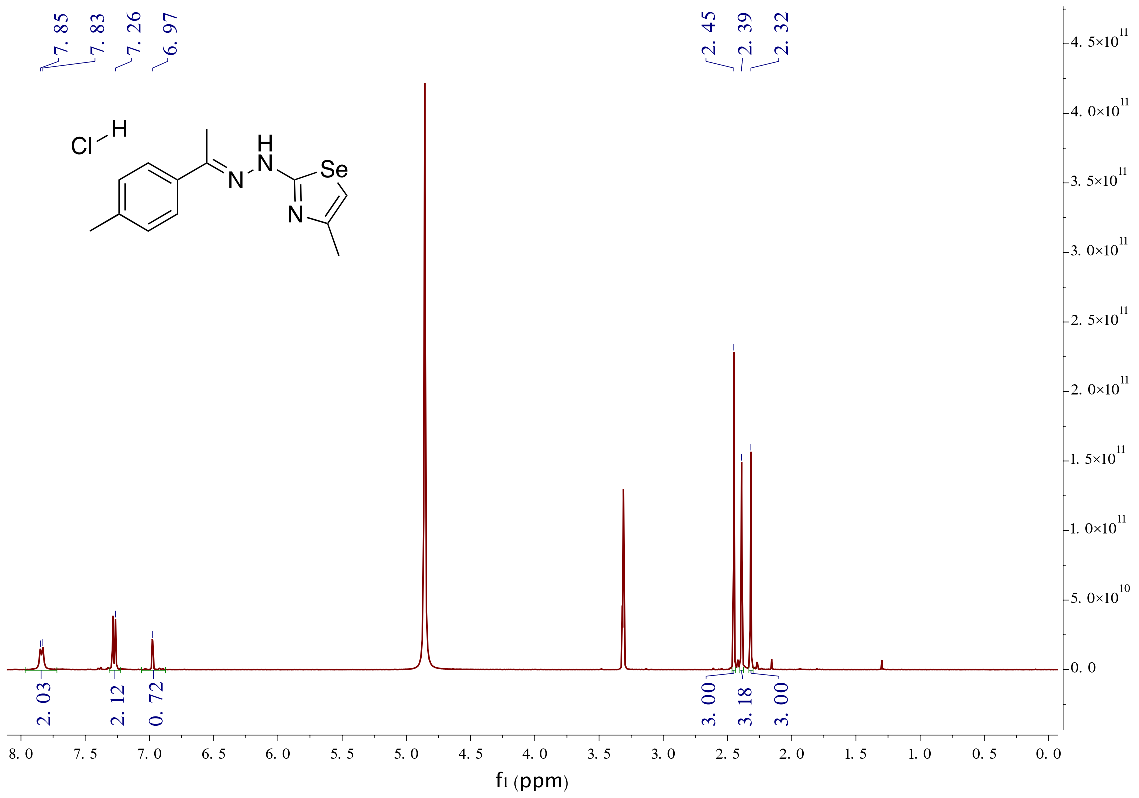

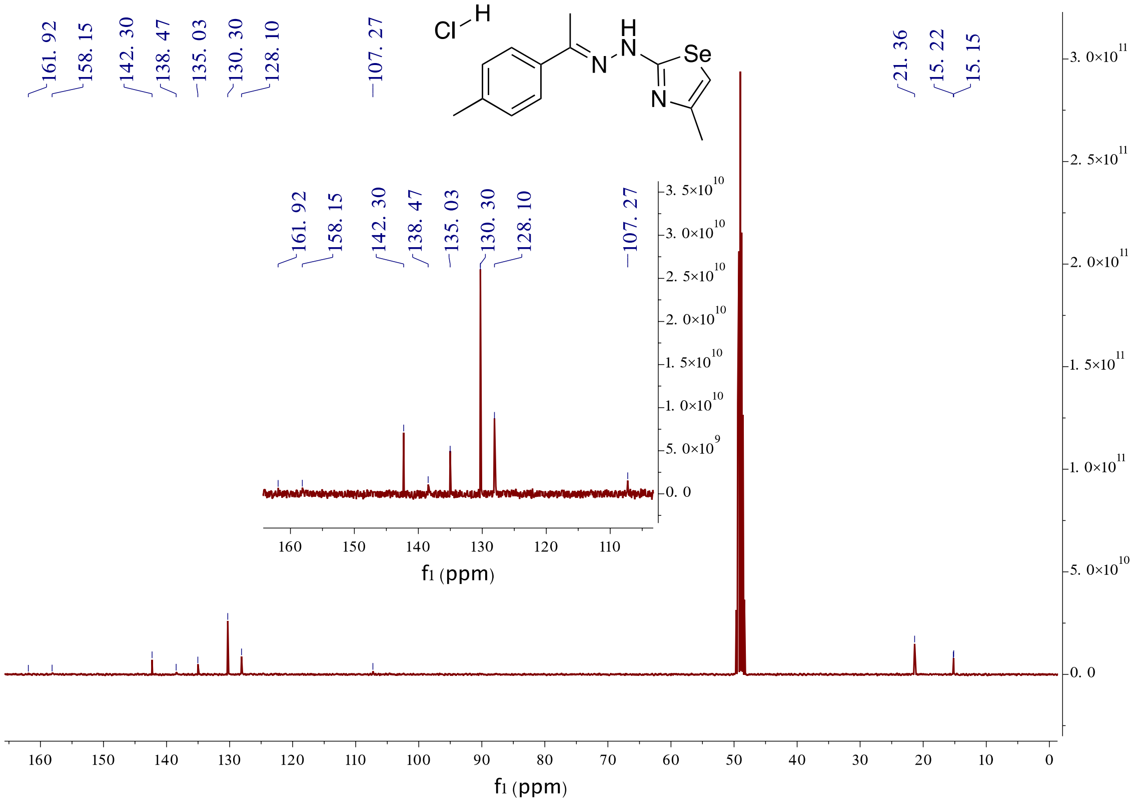

Figure S98. ^{13}C-NMR (up) and qNMR (down) of compound ***Se*2a**.

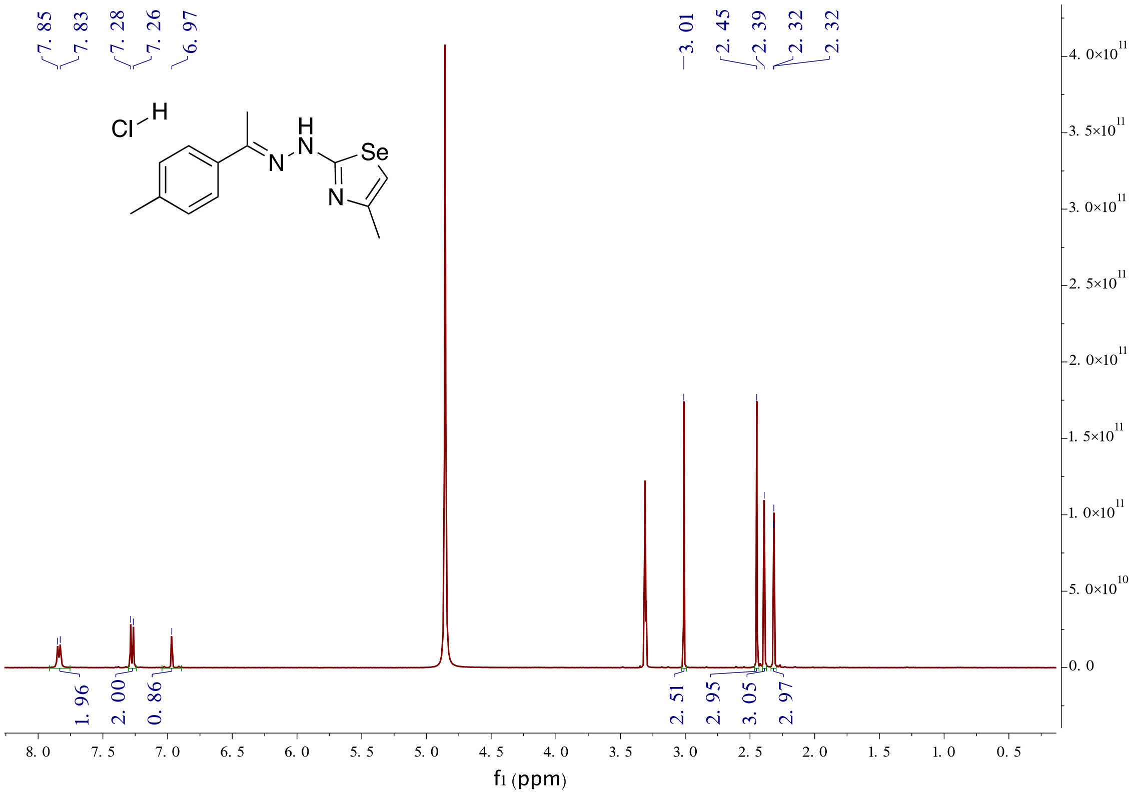

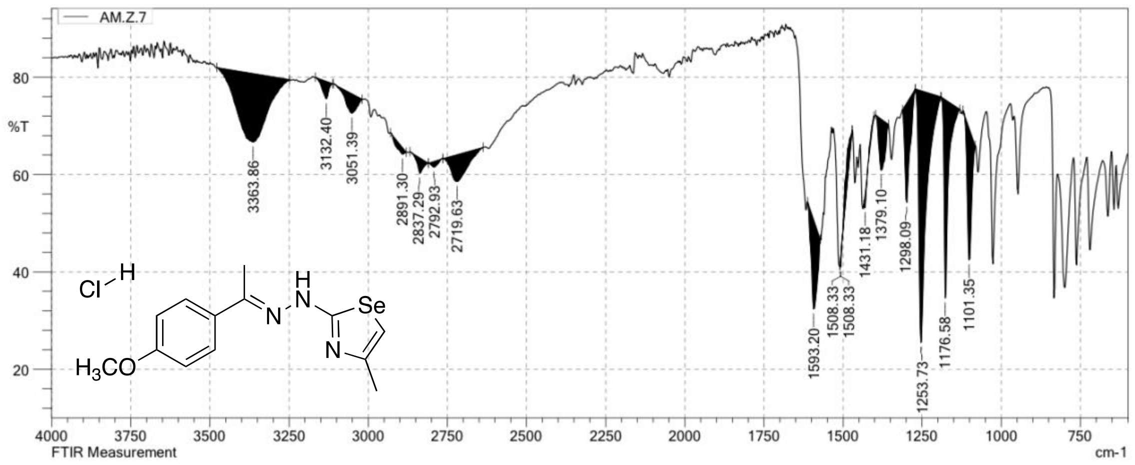

Figure S99. IR (up) and ^{1}H-NMR (down) of compound ***Se*2b**.

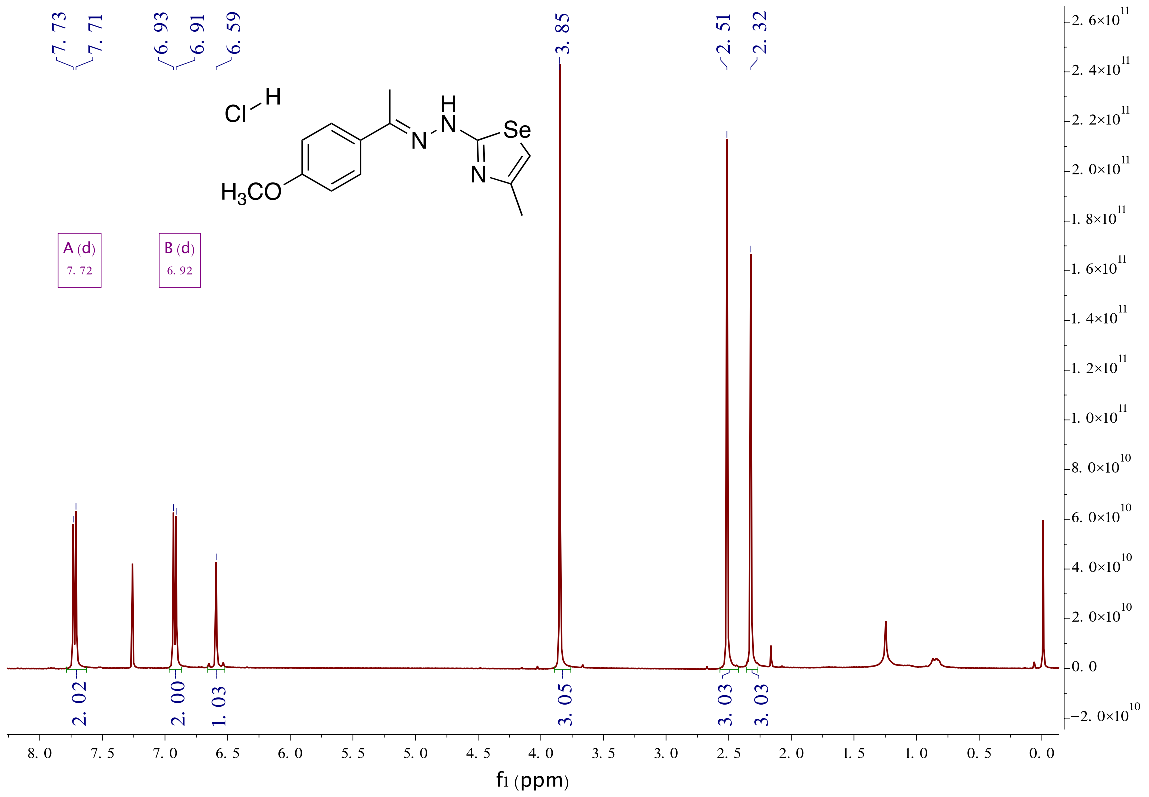

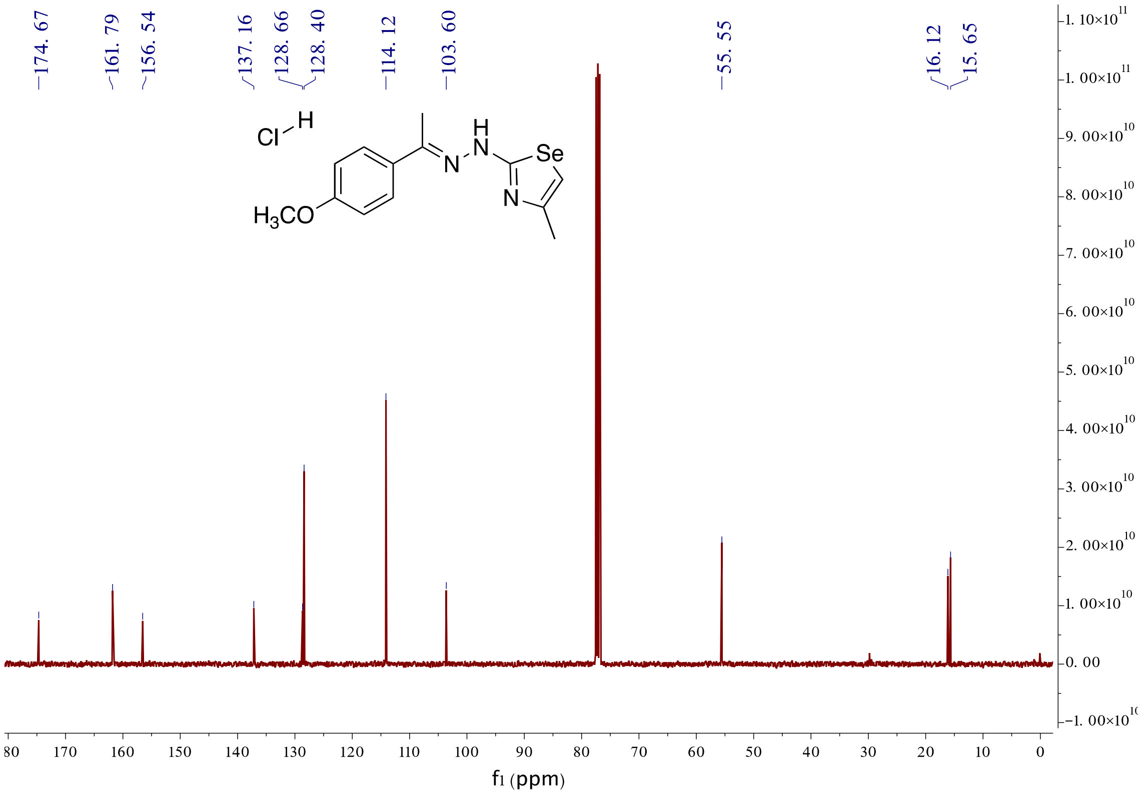

Figure S100. ^{13}C-NMR (up) and qNMR (down) of compound ***Se*2b**.

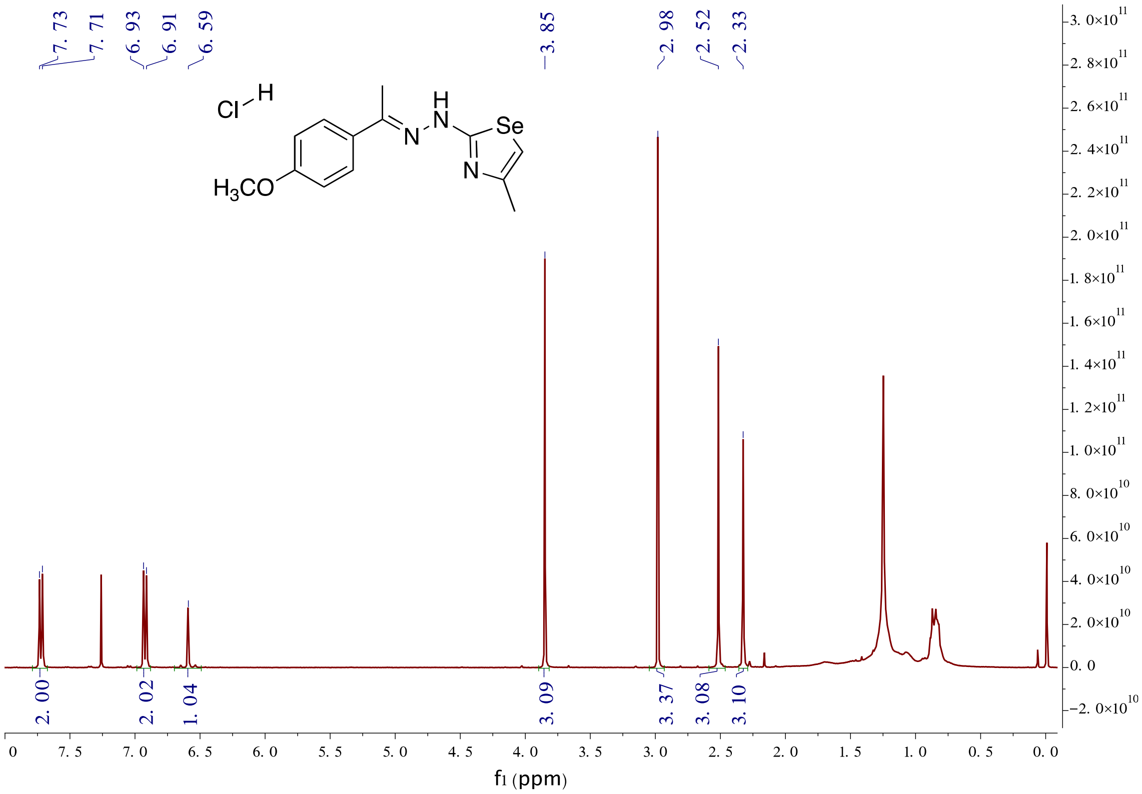

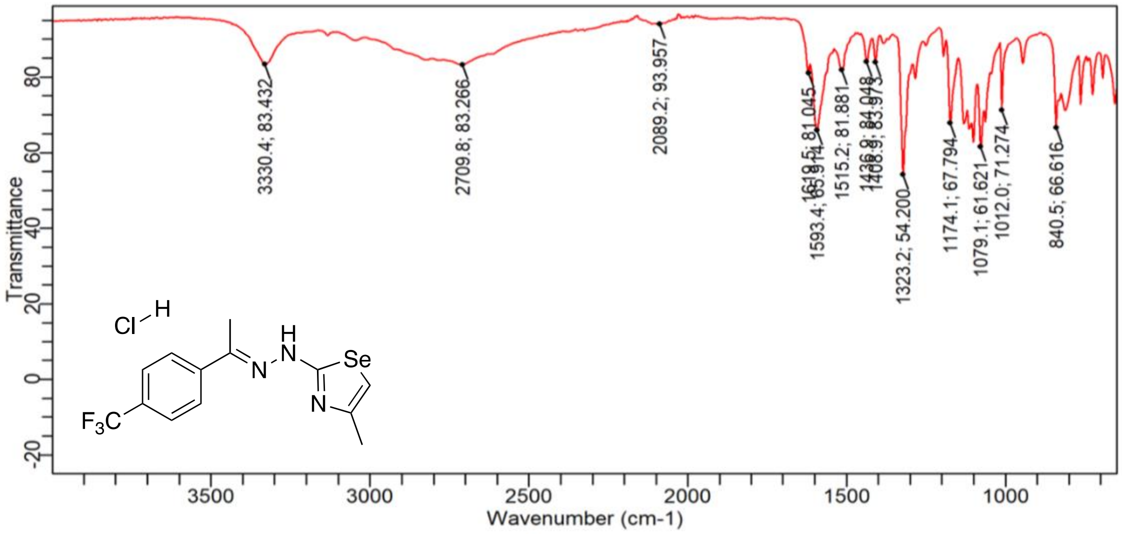

Figure S101. IR (up) and ^{1}H-NMR (down) of compound ***Se*2d**.

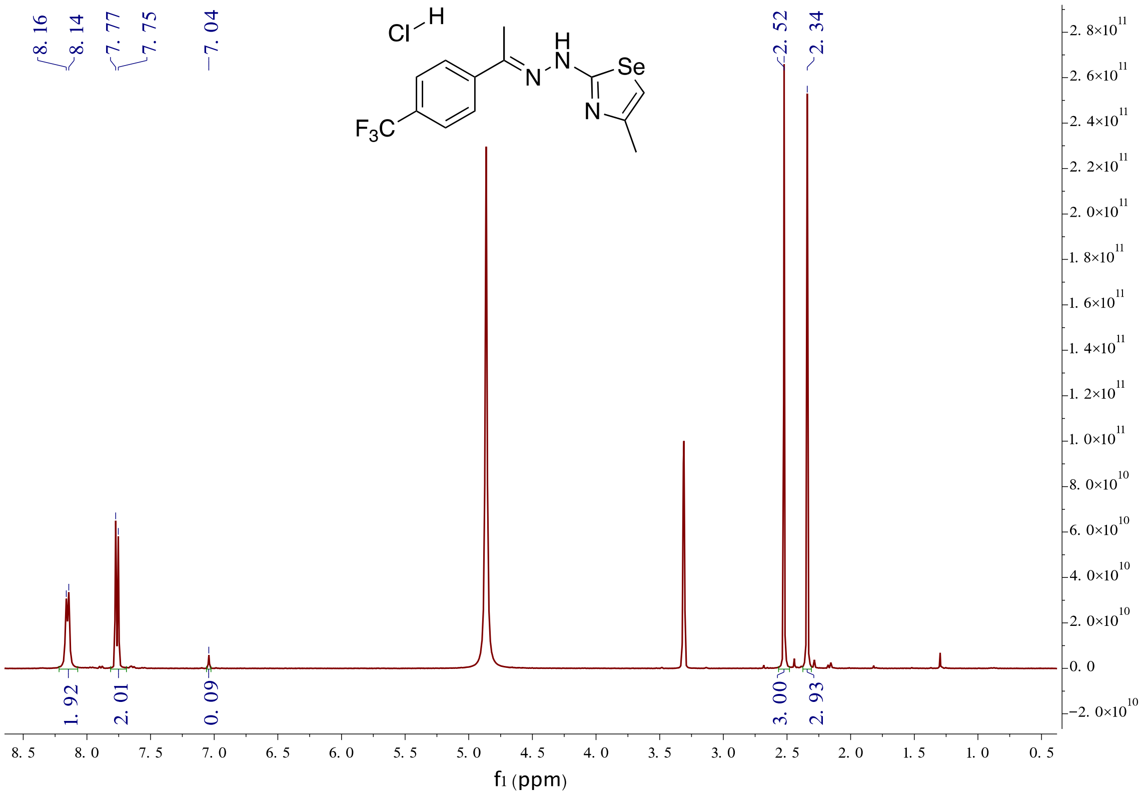

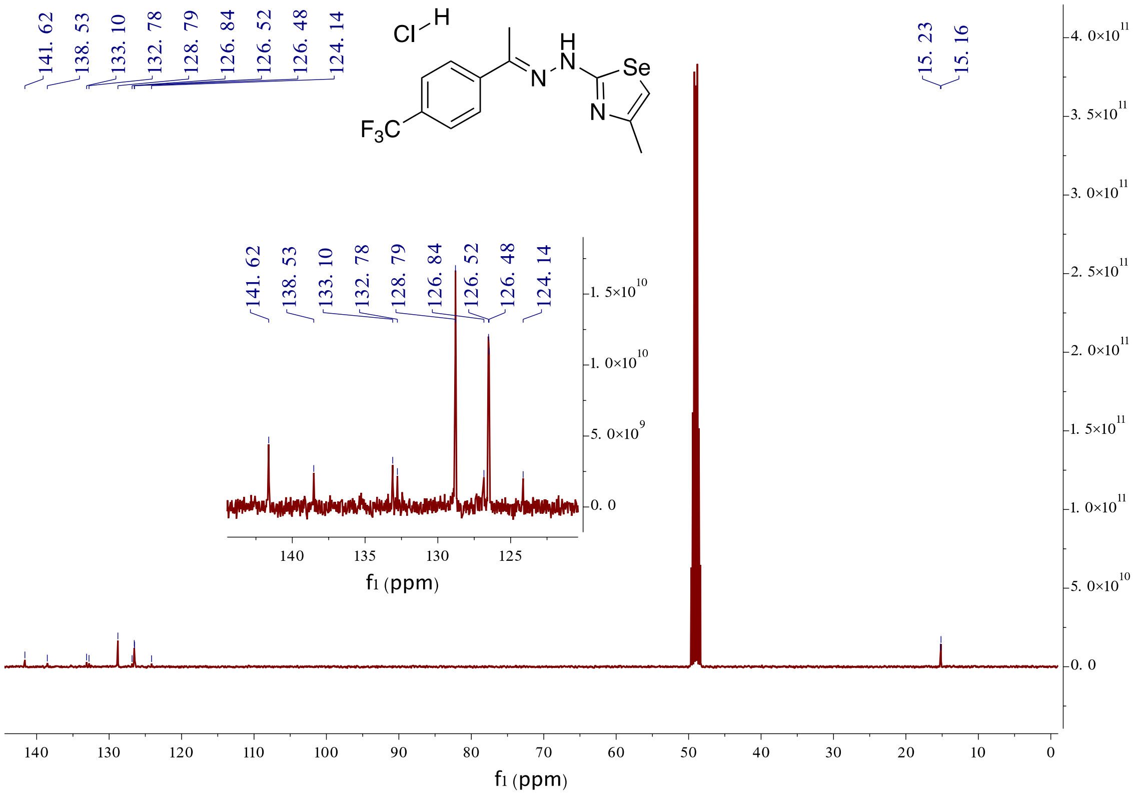

Figure S102. ^{13}C-NMR (up) and qNMR (down) of compound ***Se*2d**.

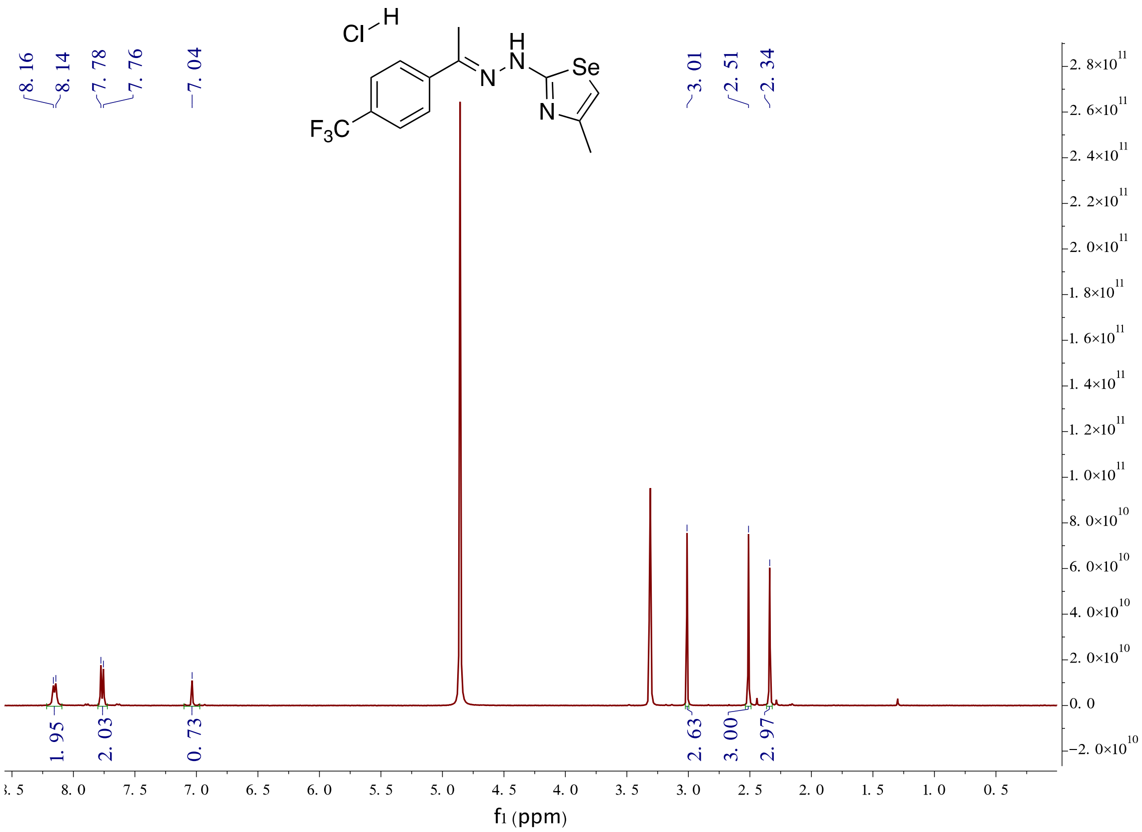

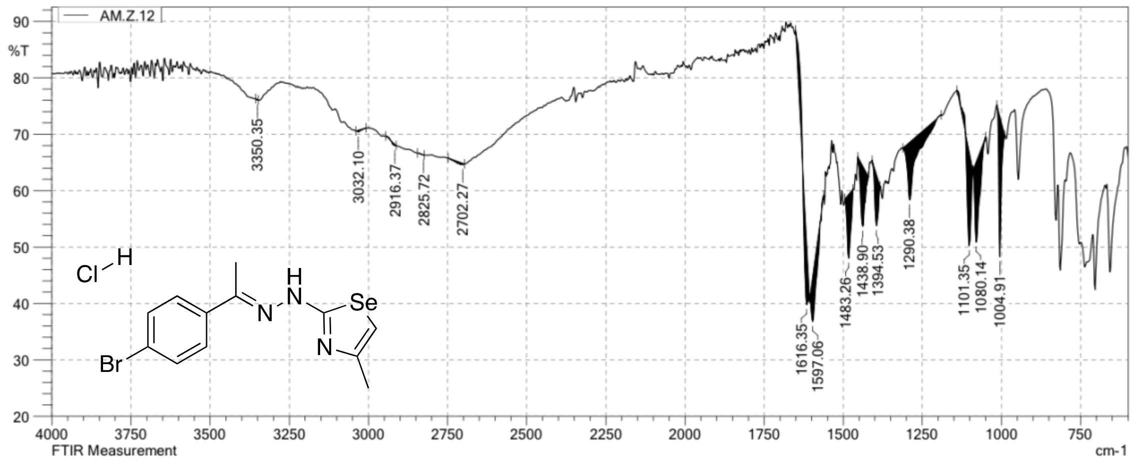

Figure S103. IR (up) and ^{1}H-NMR (down) of compound ***Se*2f**.

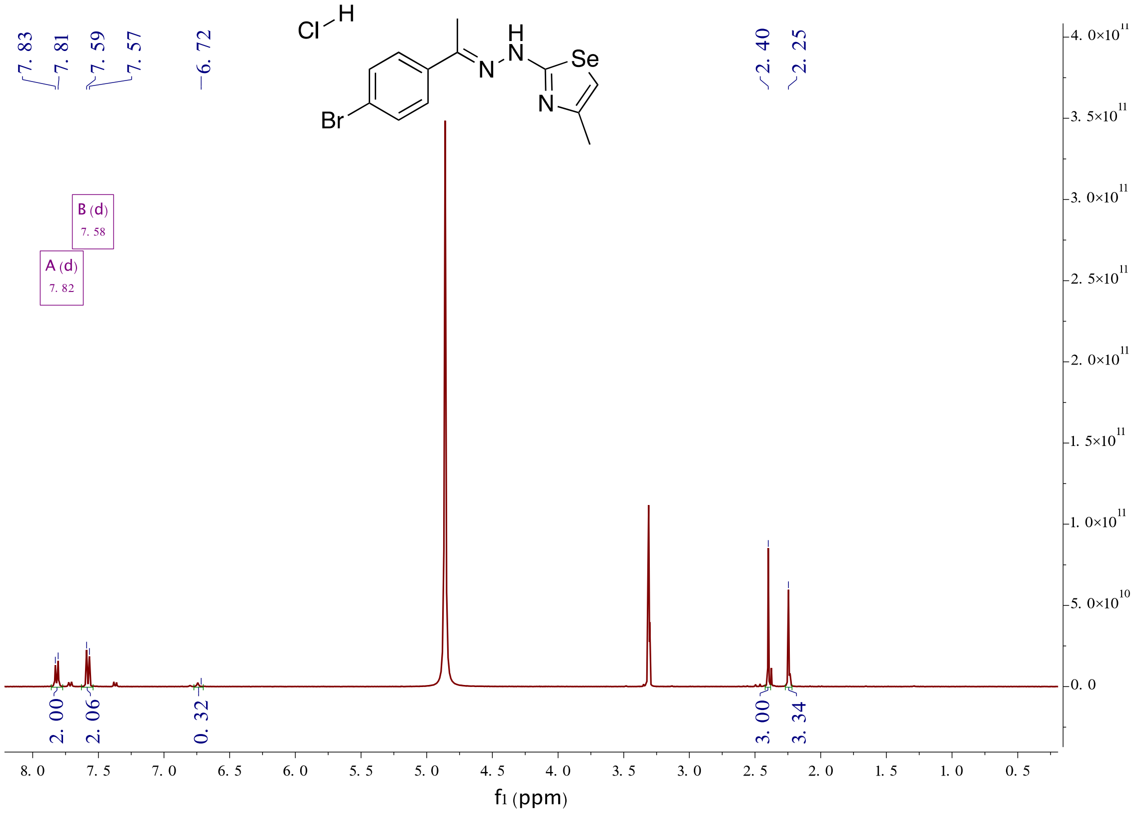

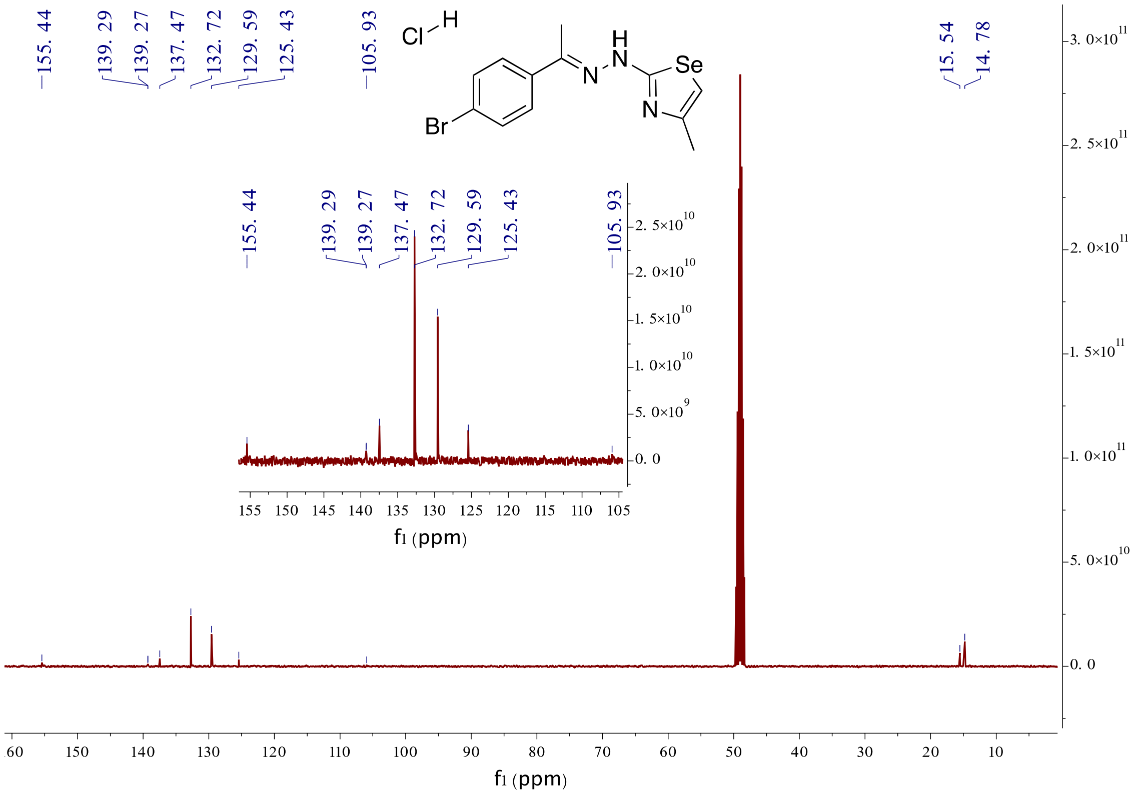

Figure S104. ^{13}C-NMR (up) and qNMR (down) of compound ***Se*2f**.

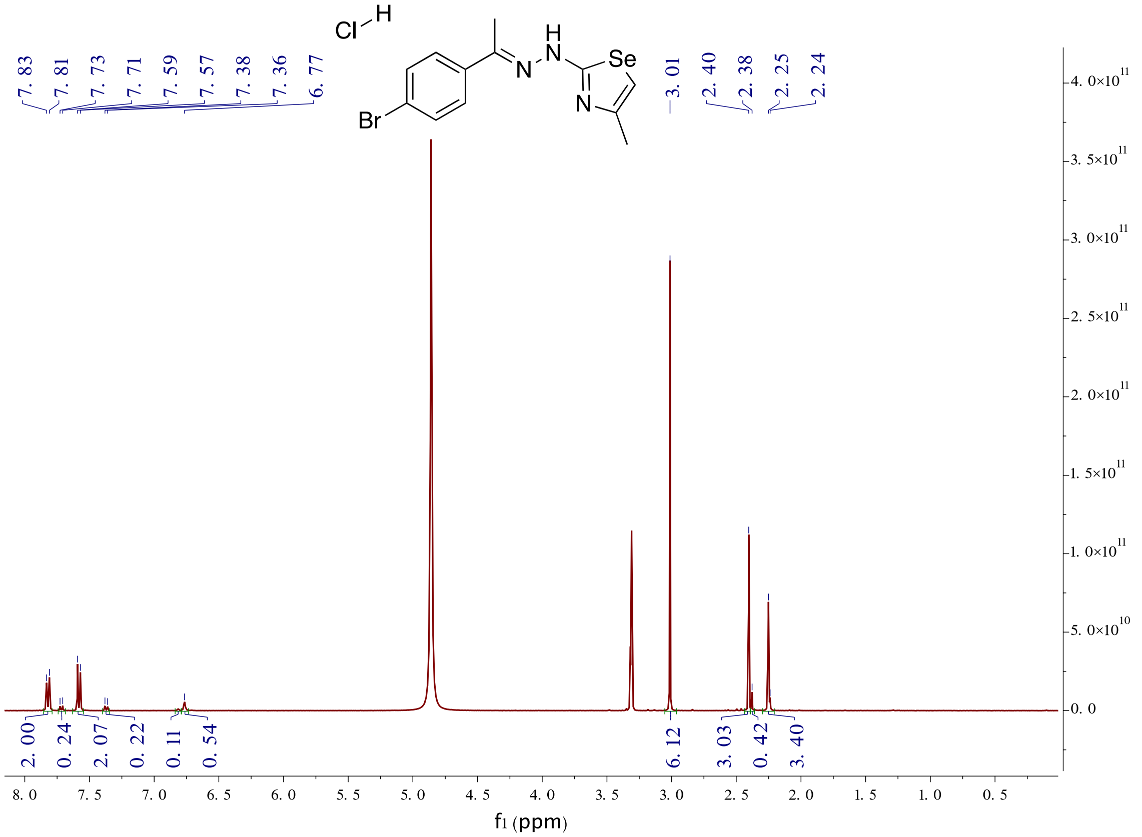

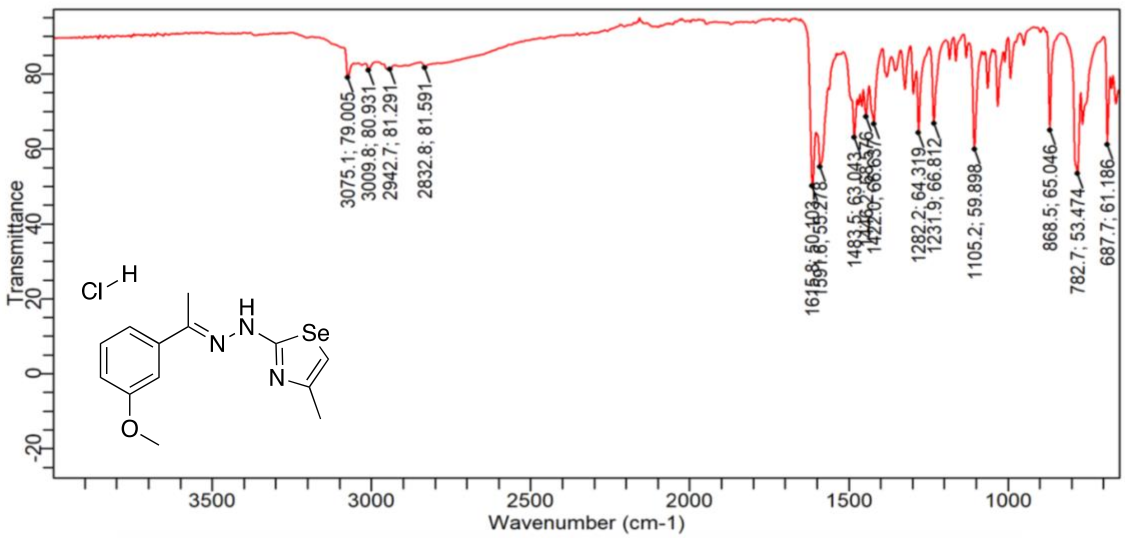

Figure S105. IR (up) and ^{1}H-NMR (down) of compound ***Se*2g**.

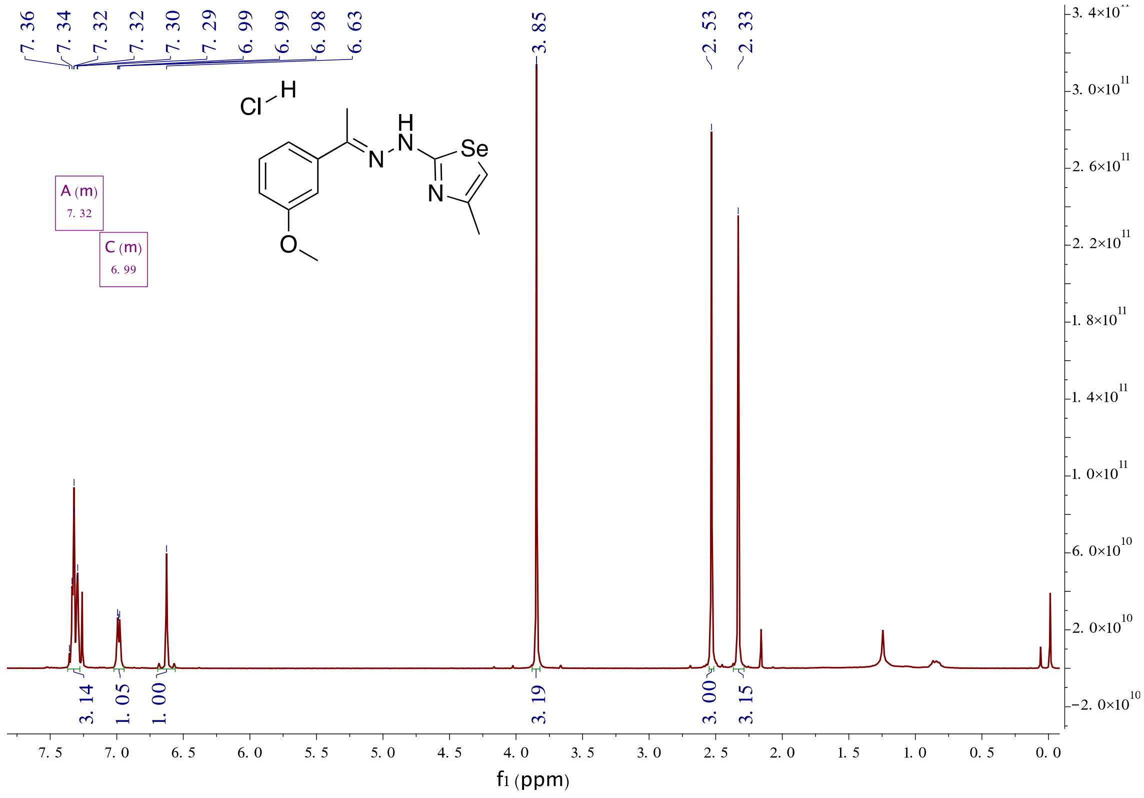

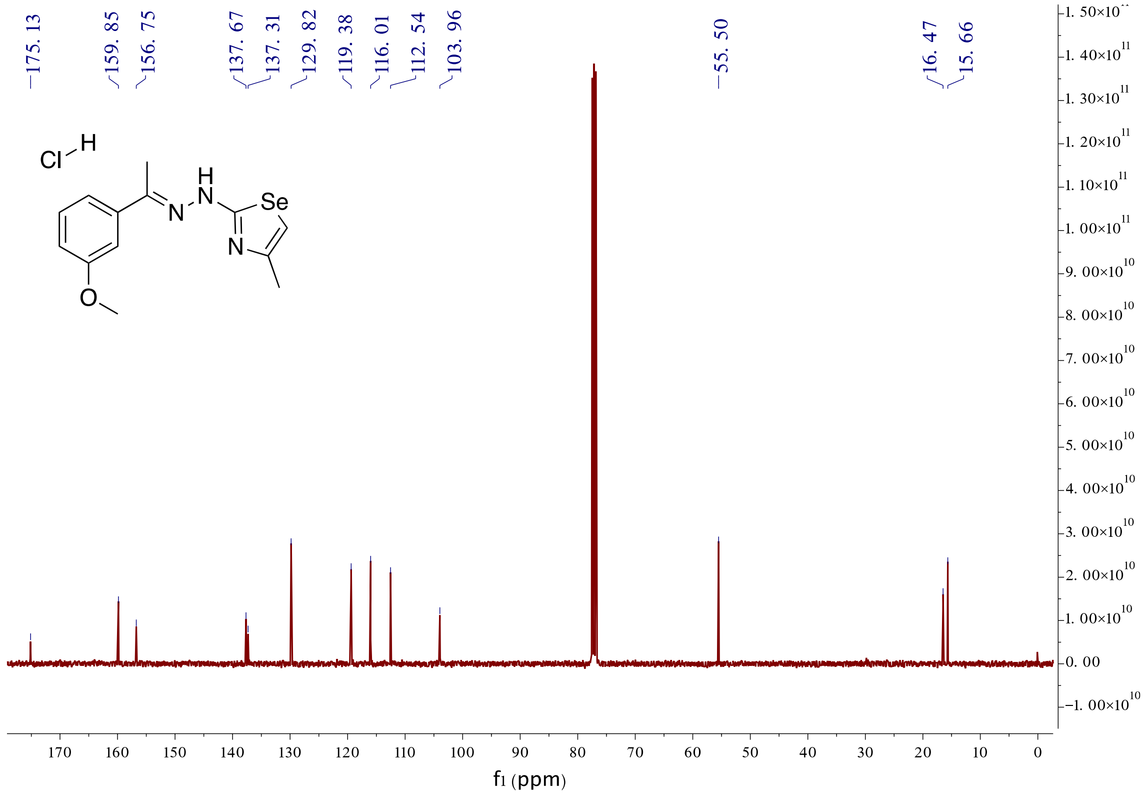

Figure S106. ^{13}C-NMR (up) and qNMR (down) of compound ***Se*2g**.

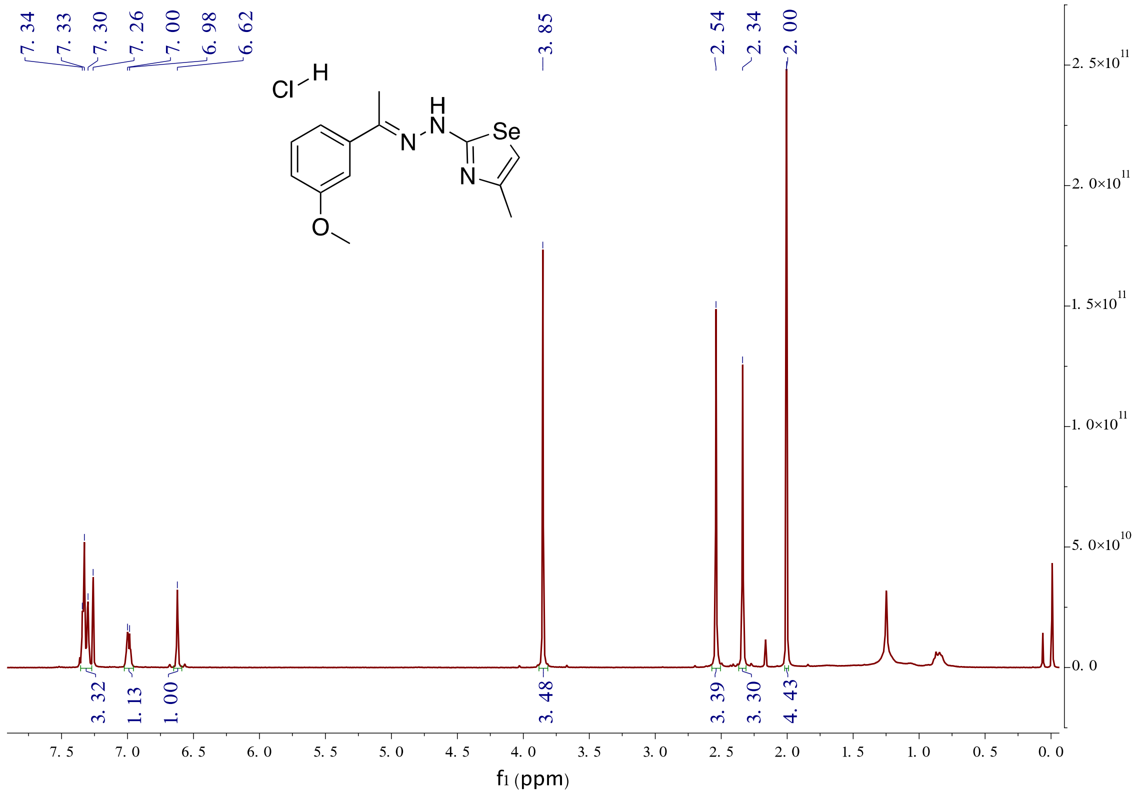

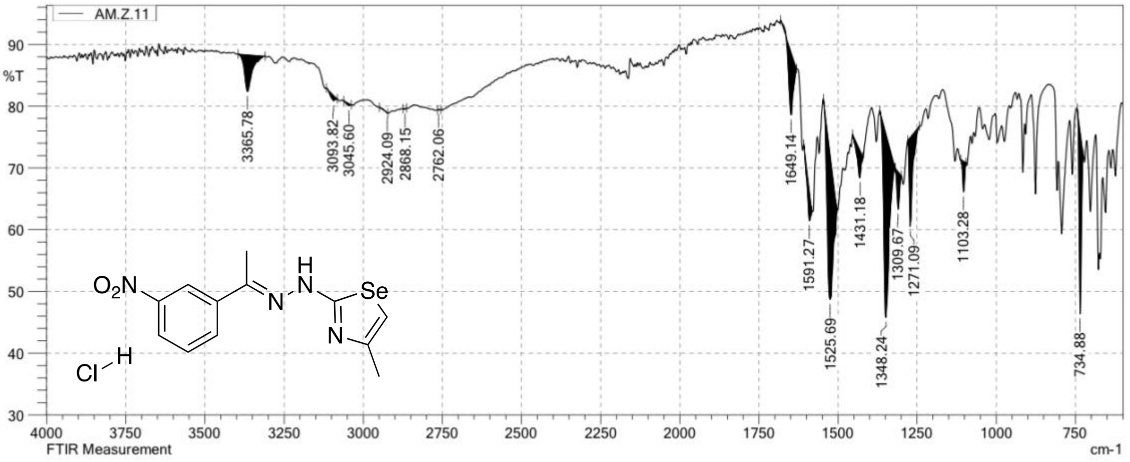

Figure S107. IR (up) and ^{1}H-NMR (down) of compound ***Se*2h**.

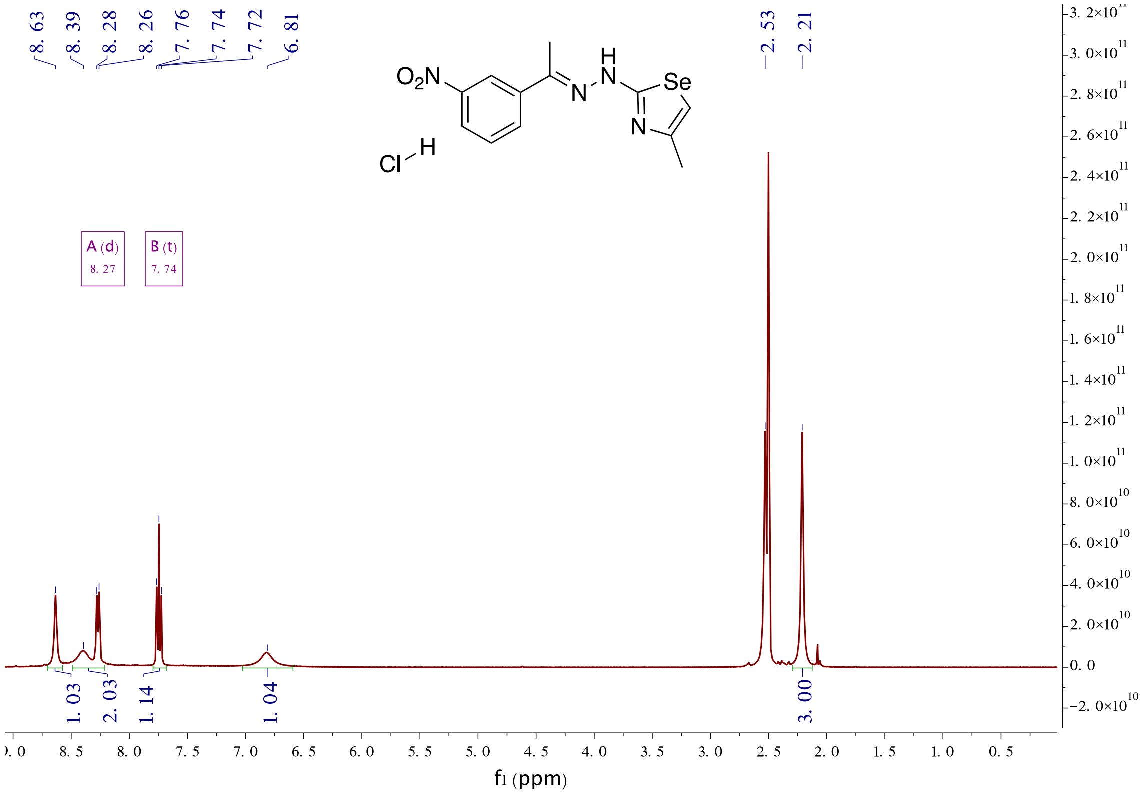

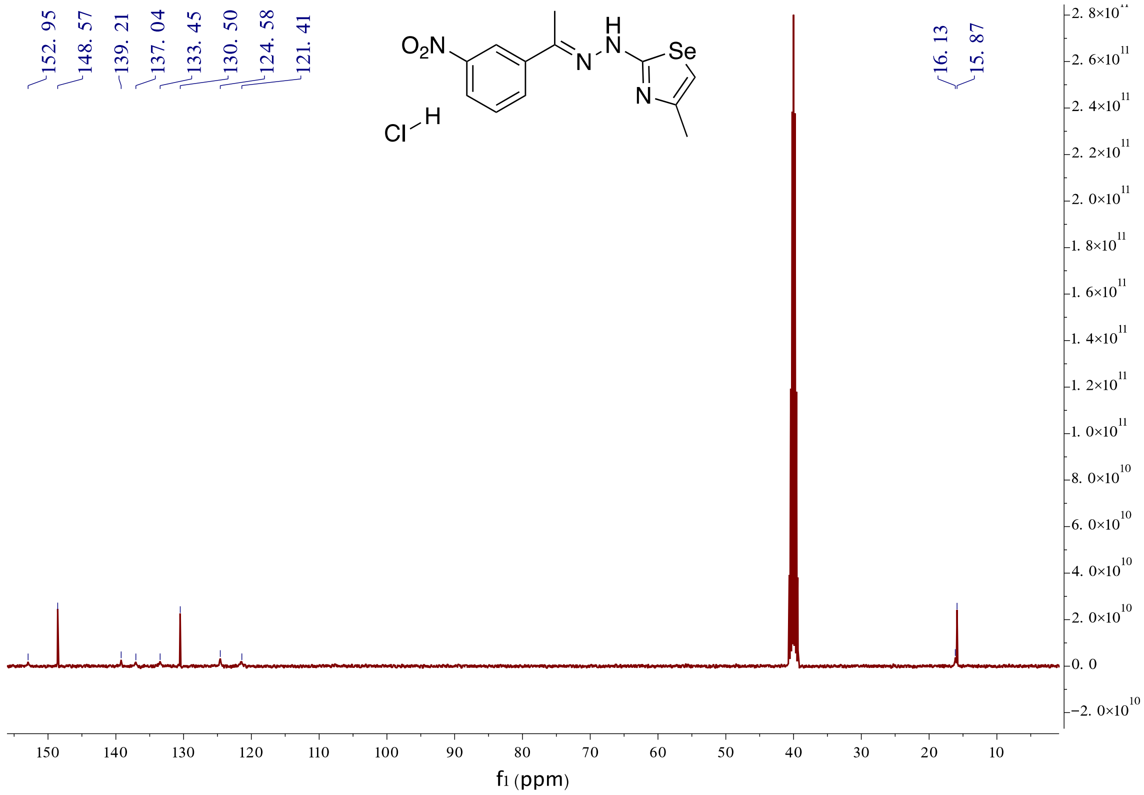

Figure S108. ^{13}C-NMR (up) and qNMR (down) of compound ***Se*2h**.

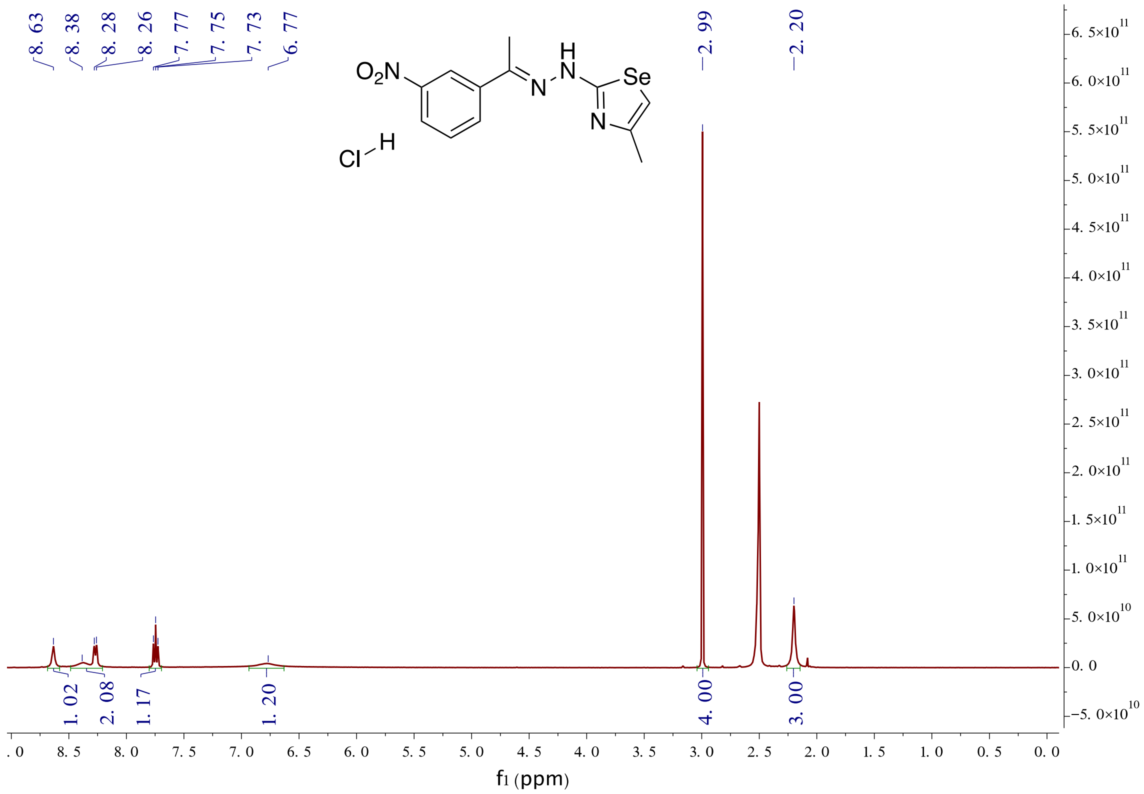

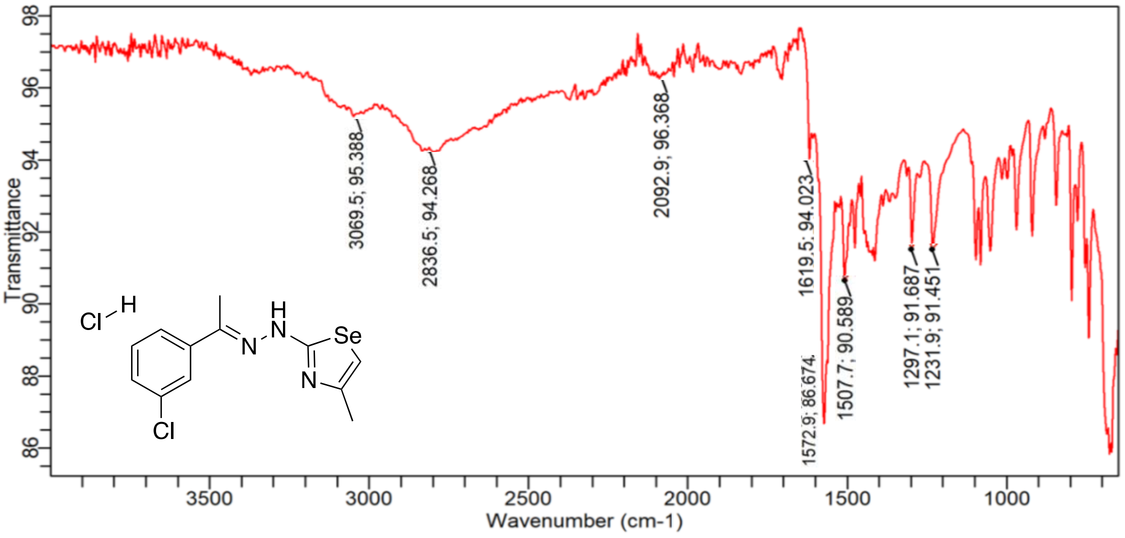

Figure S109. IR (up) and ^{1}H-NMR (down) of compound ***Se*2i**.

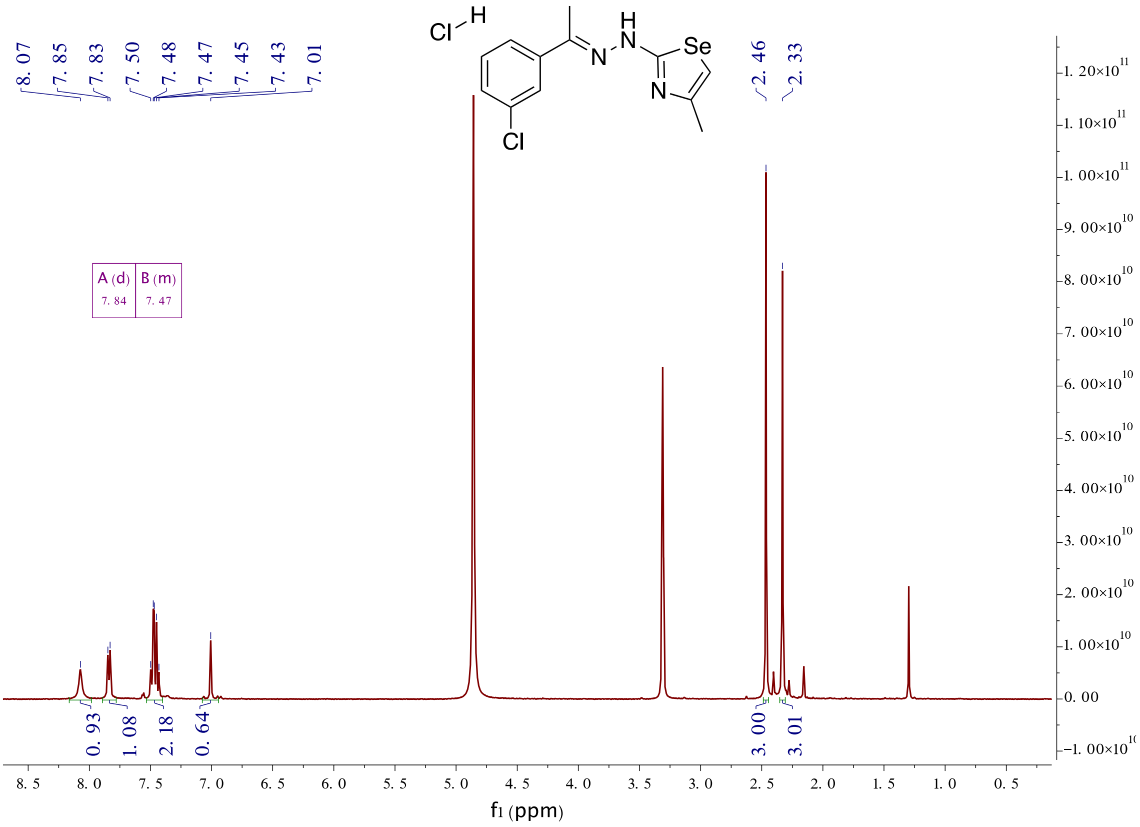

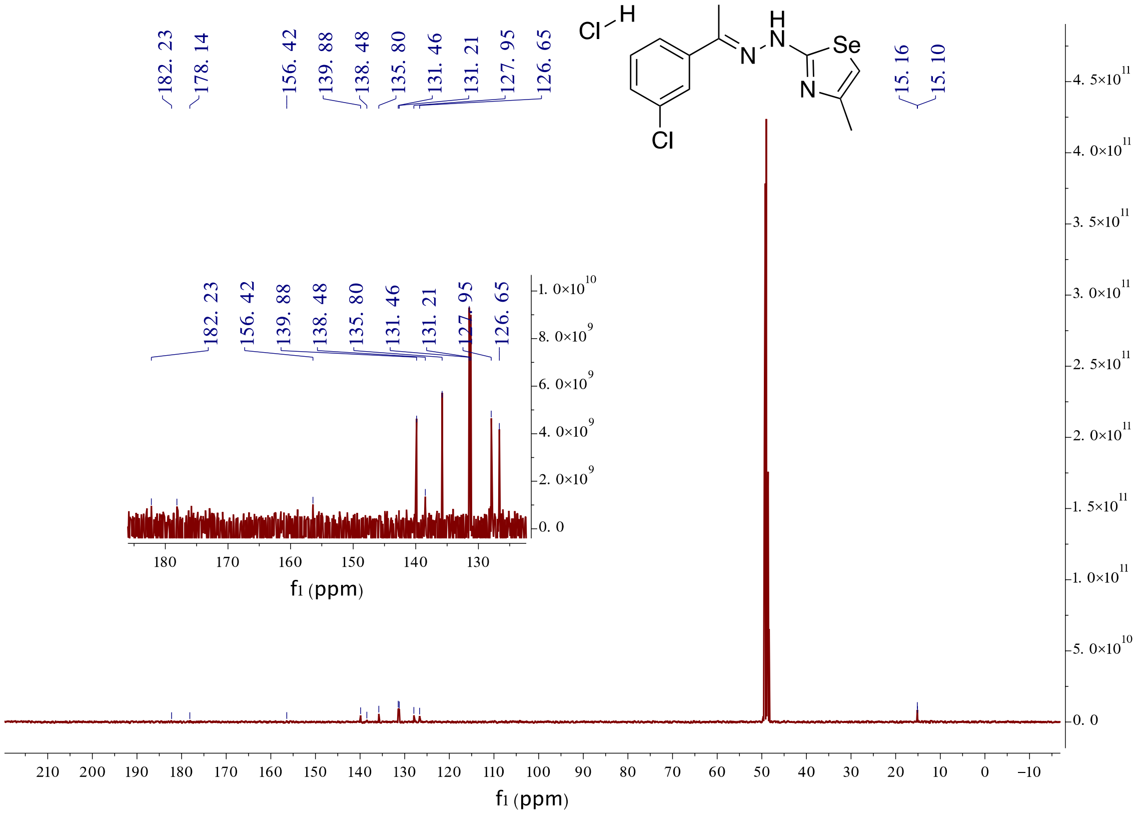

Figure S110. ^{13}C-NMR (up) and qNMR (down) of compound ***Se*2i**.

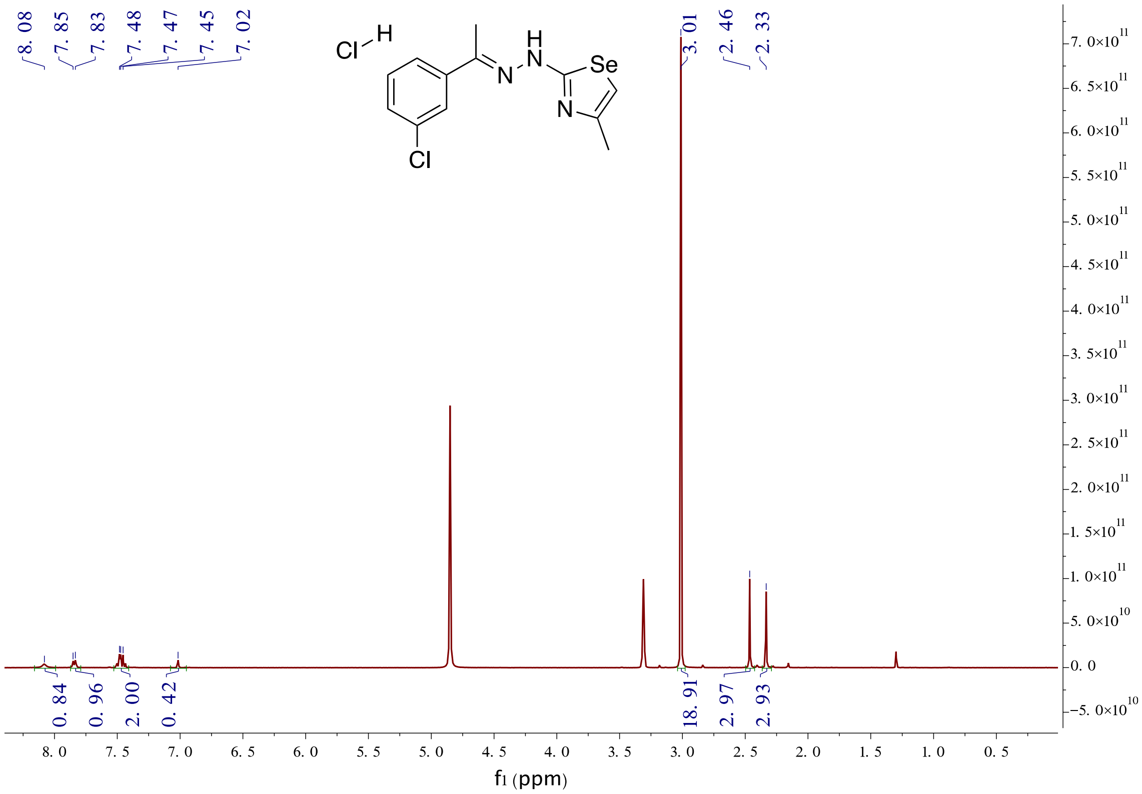

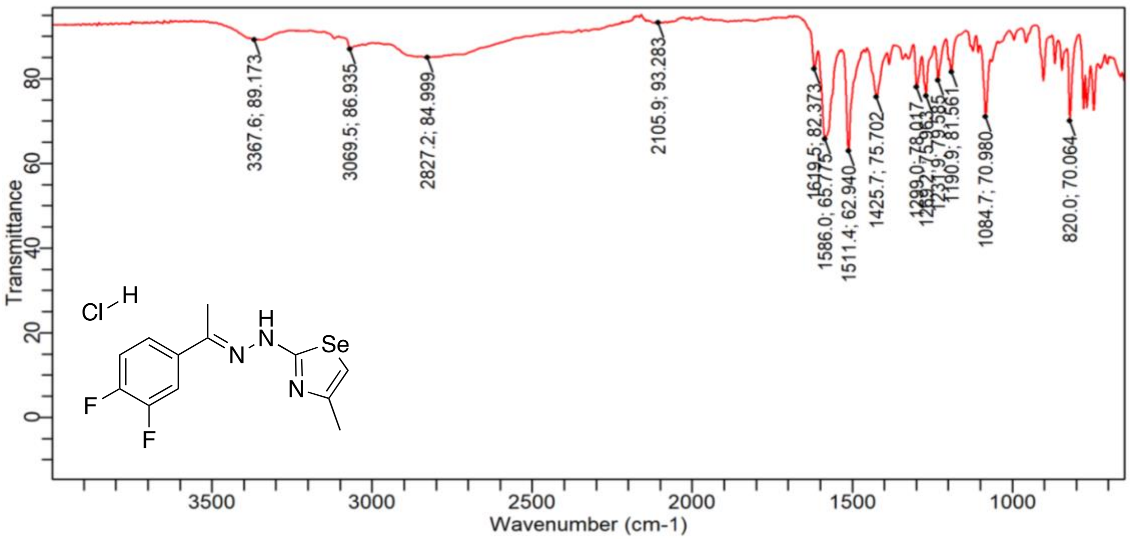

Figure S111. IR (up) and ^{1}H-NMR (down) of compound ***Se*2j**.

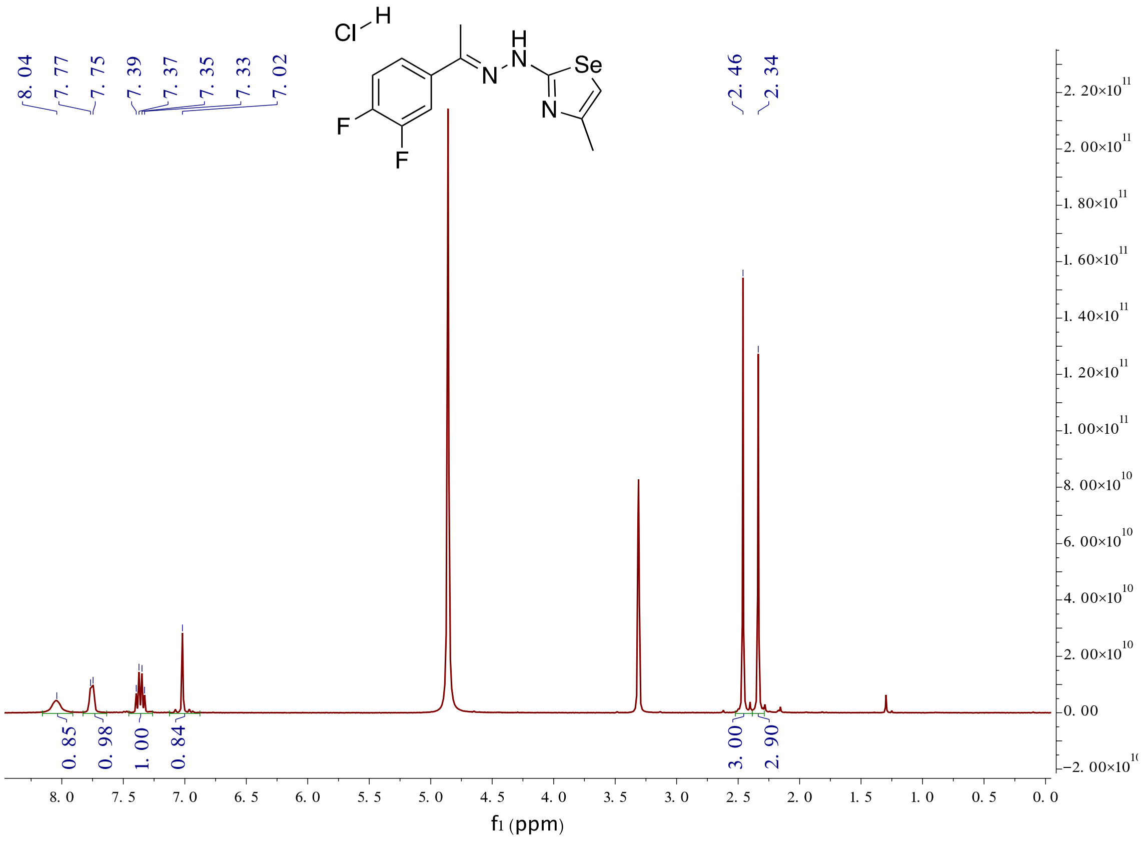

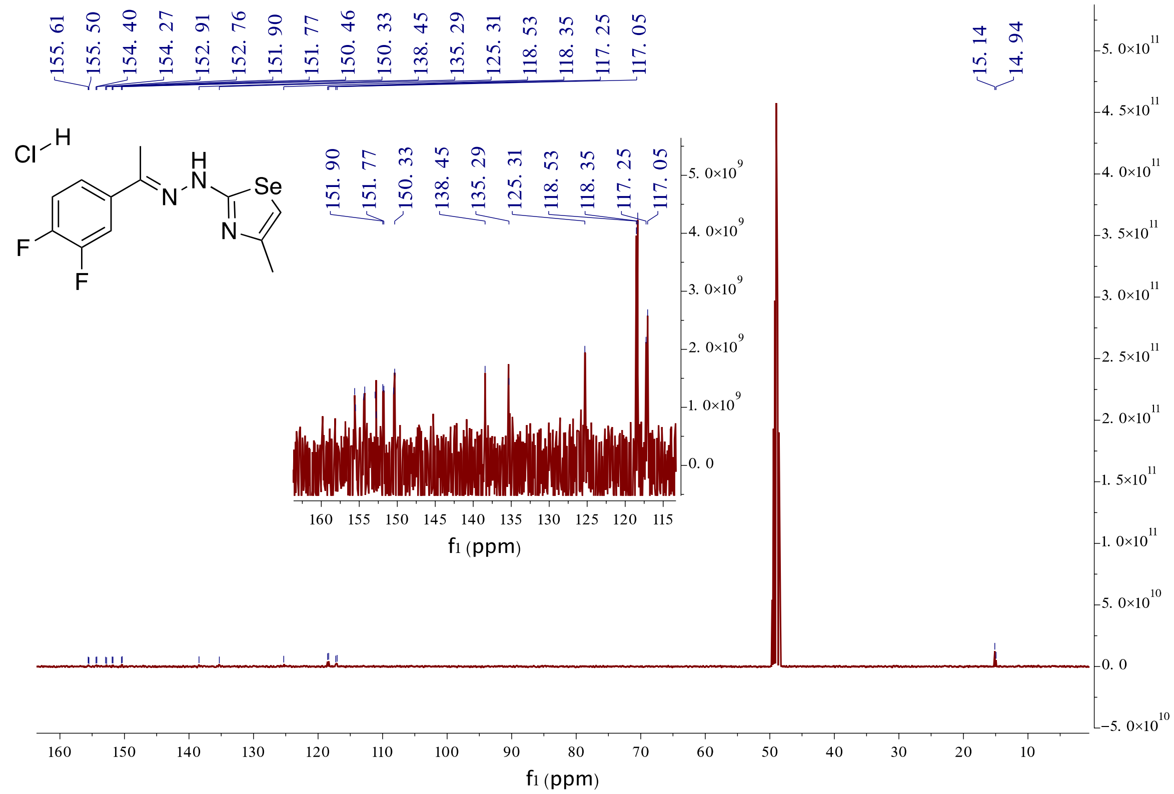

Figure S112. ^{13}C-NMR (up) and qNMR (down) of compound ***Se*2j**.

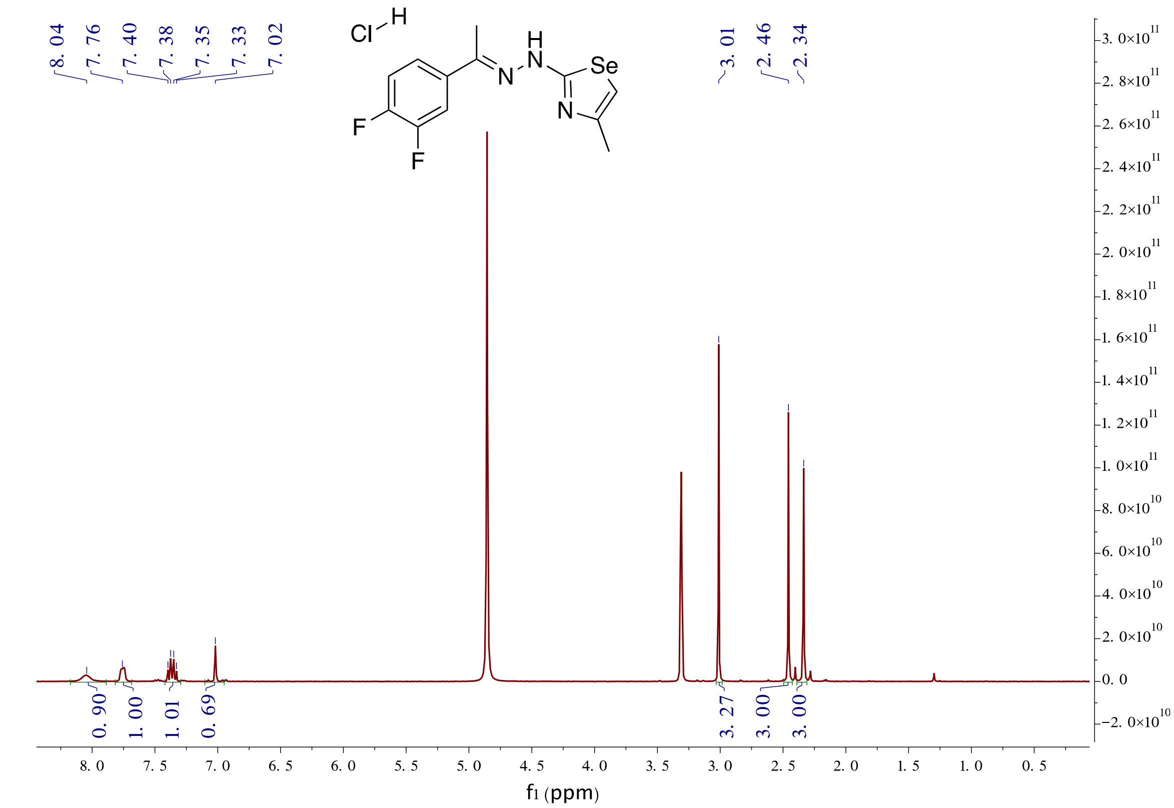

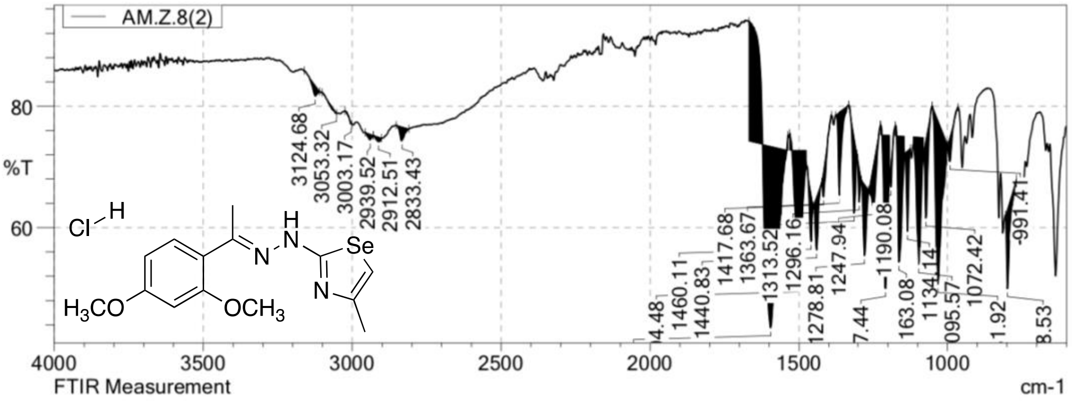

Figure S113. IR (up) and ^{1}H-NMR (down) of compound ***Se*2k**.

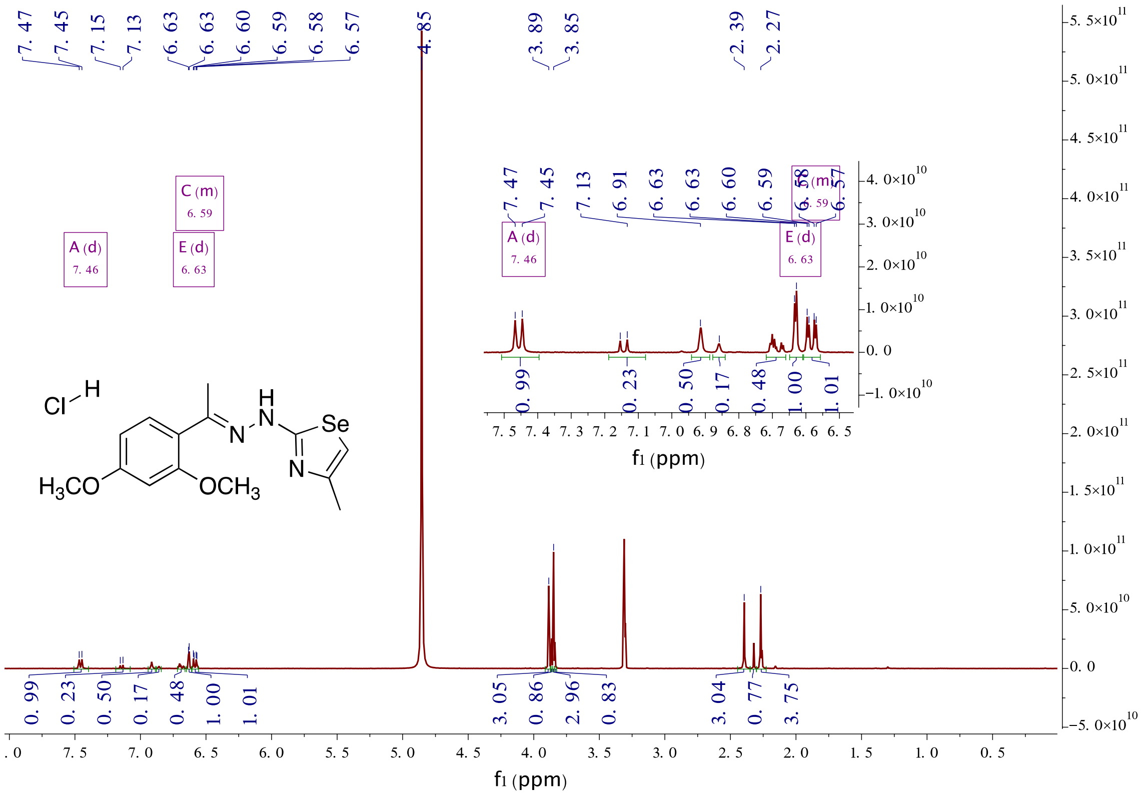

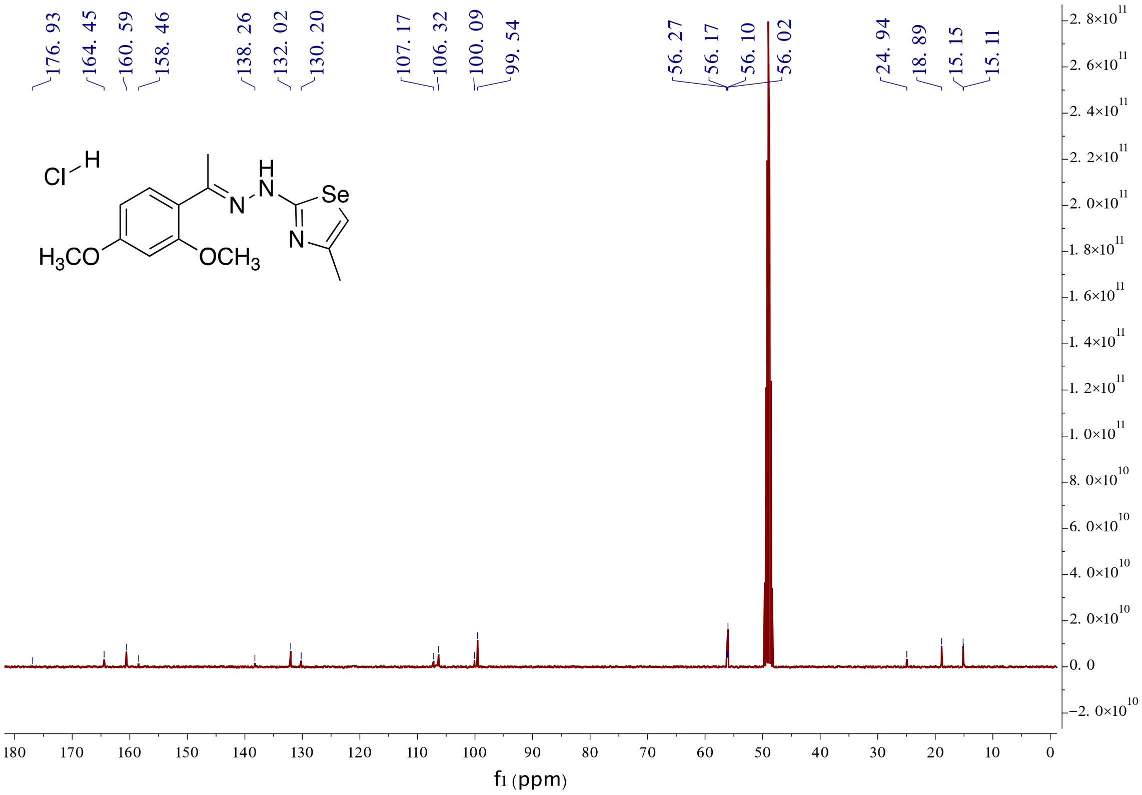

Figure S114. ^{13}C-NMR (up) and qNMR (down) of compound ***Se*2k**.

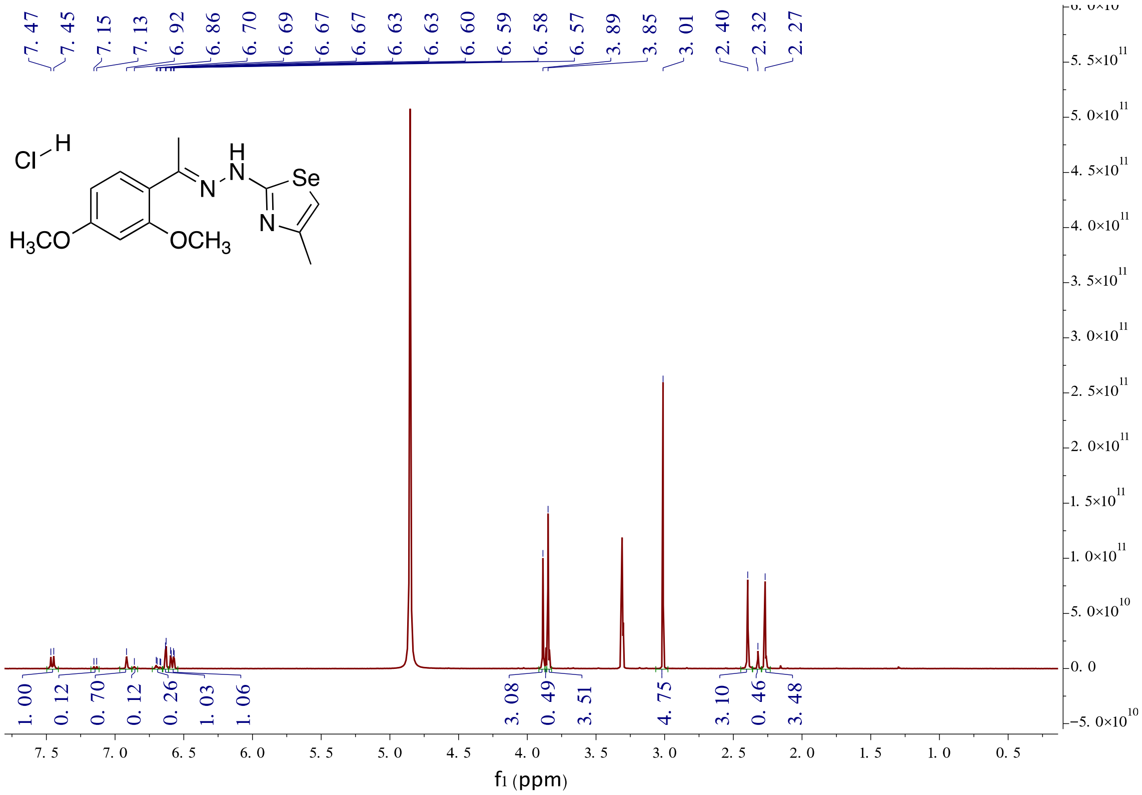

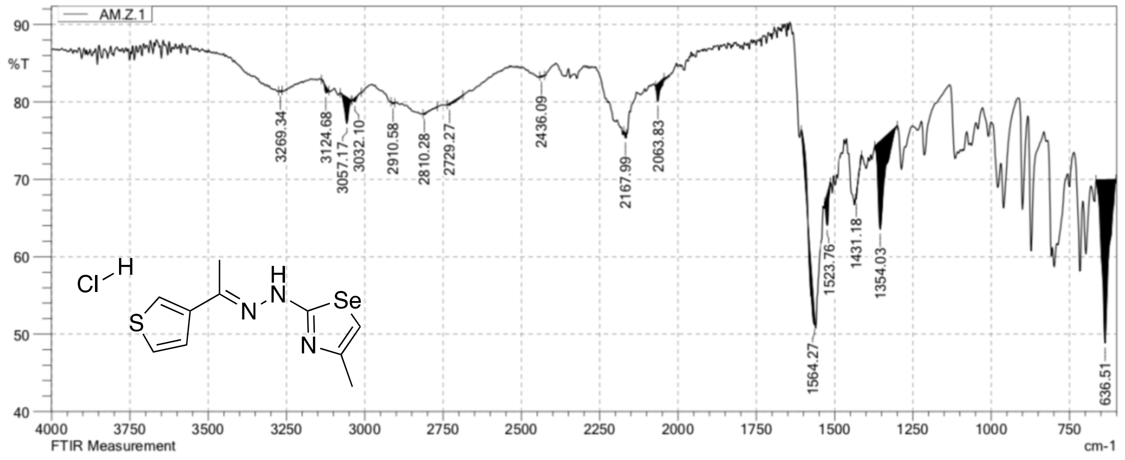

Figure S115. IR (up) and ^{1}H-NMR (down) of compound ***Se*2l**.

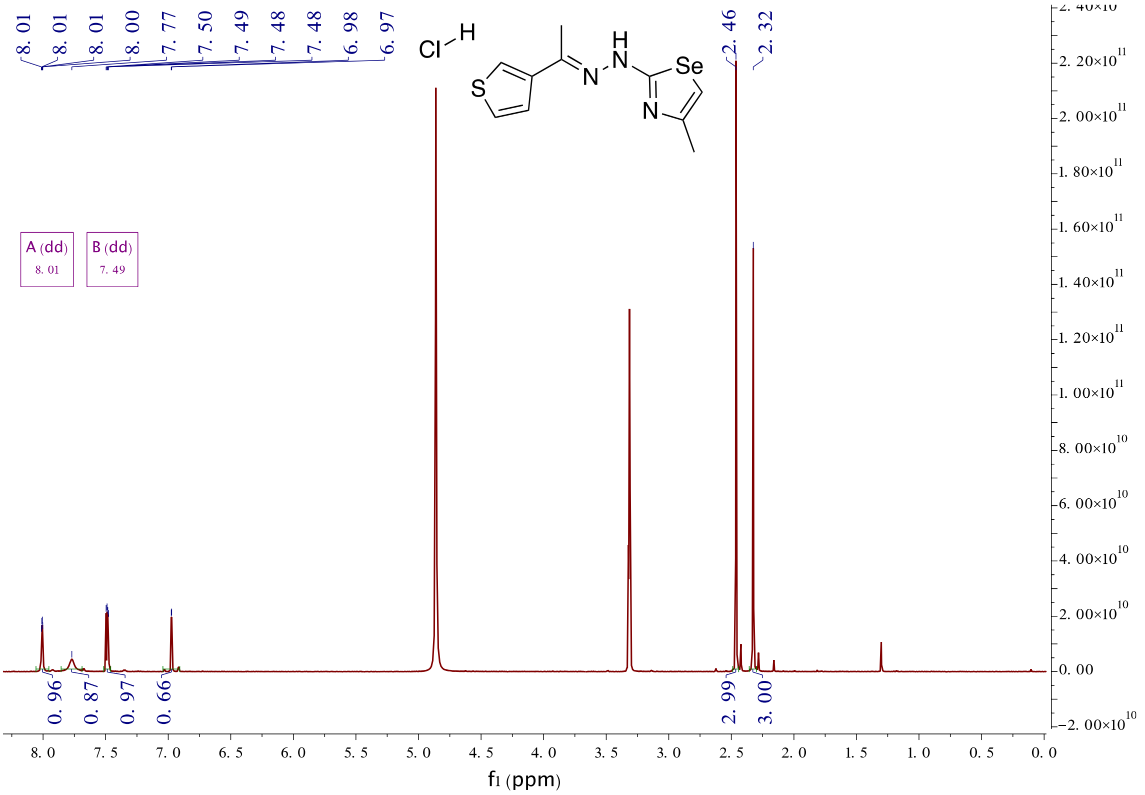

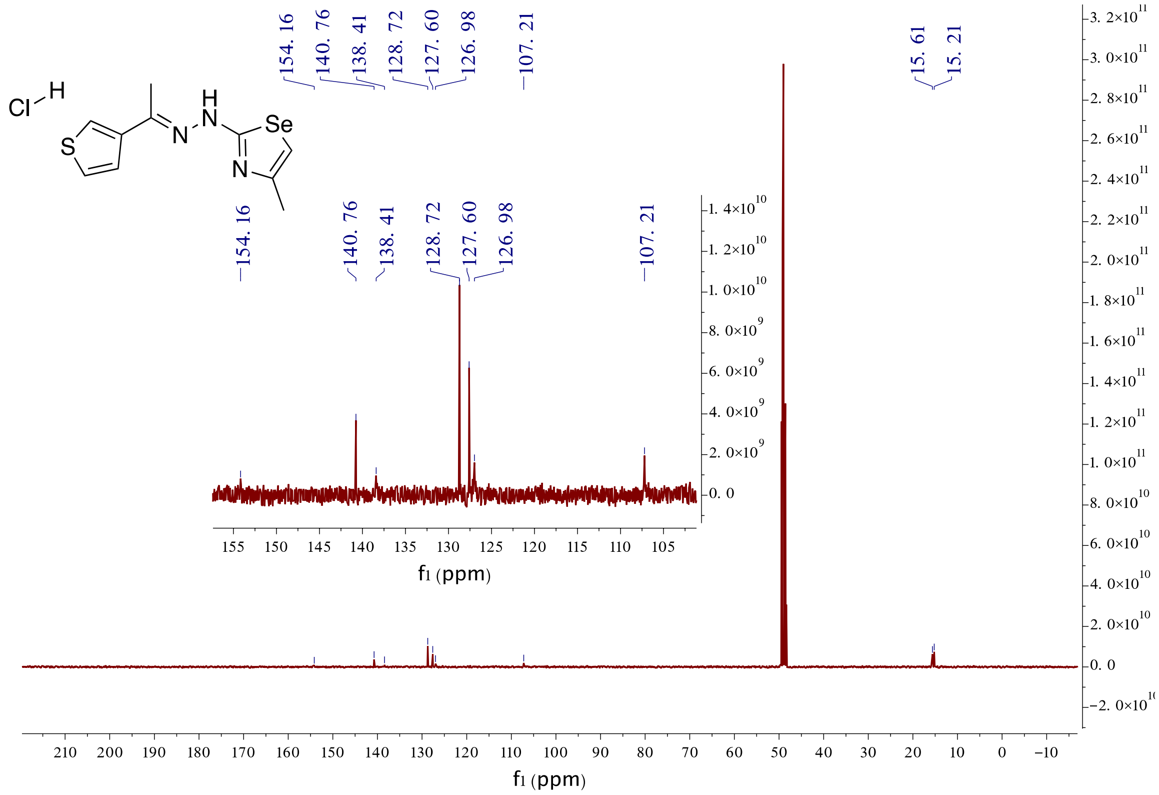

Figure S116. ^{13}C-NMR (up) and qNMR (down) of compound ***Se*2l**.

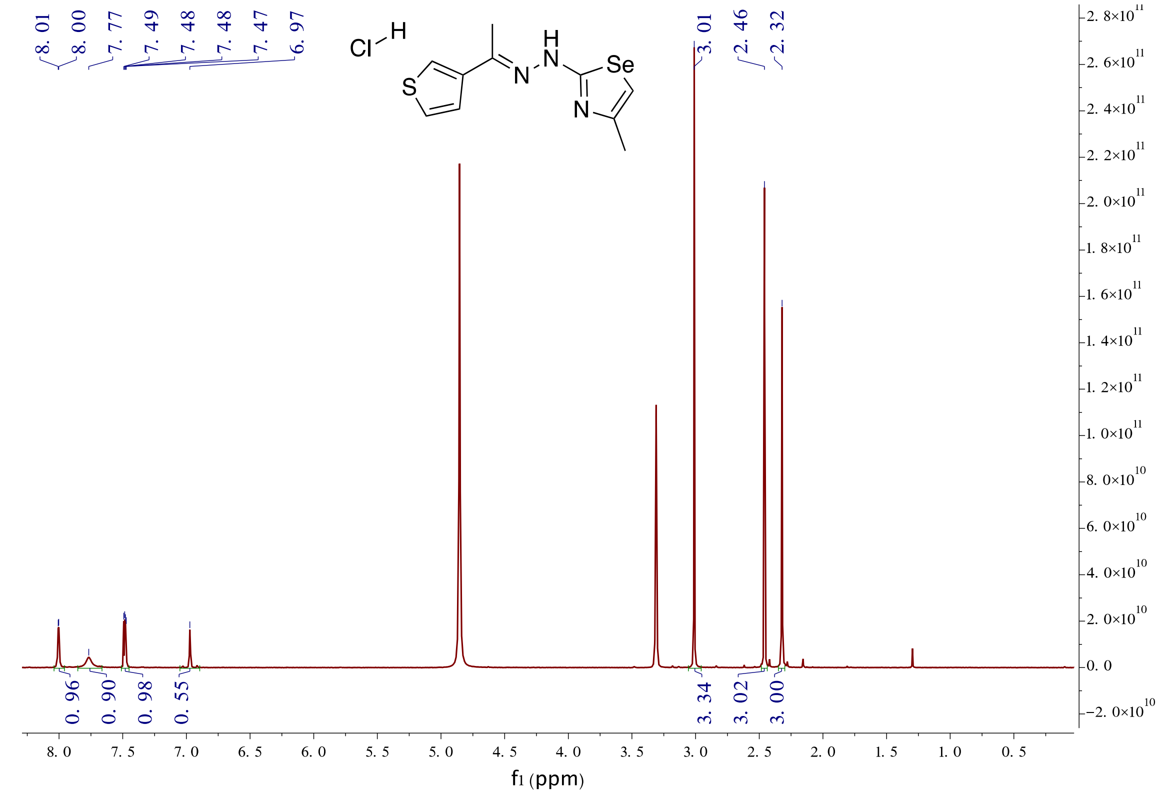

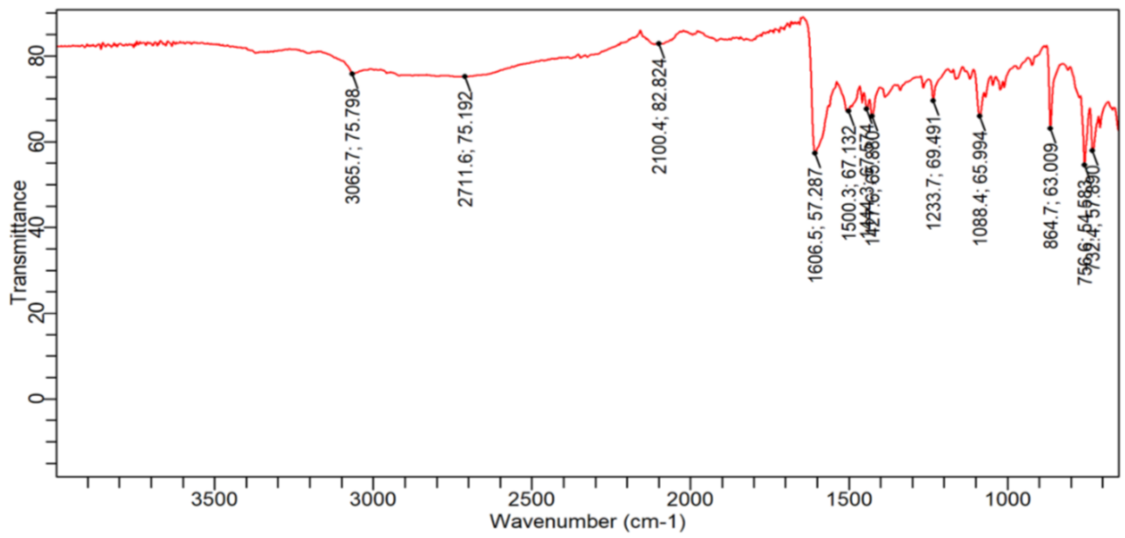

Figure S117. IR (up) and ^{1}H-NMR (down) of compound ***Se*2o**.

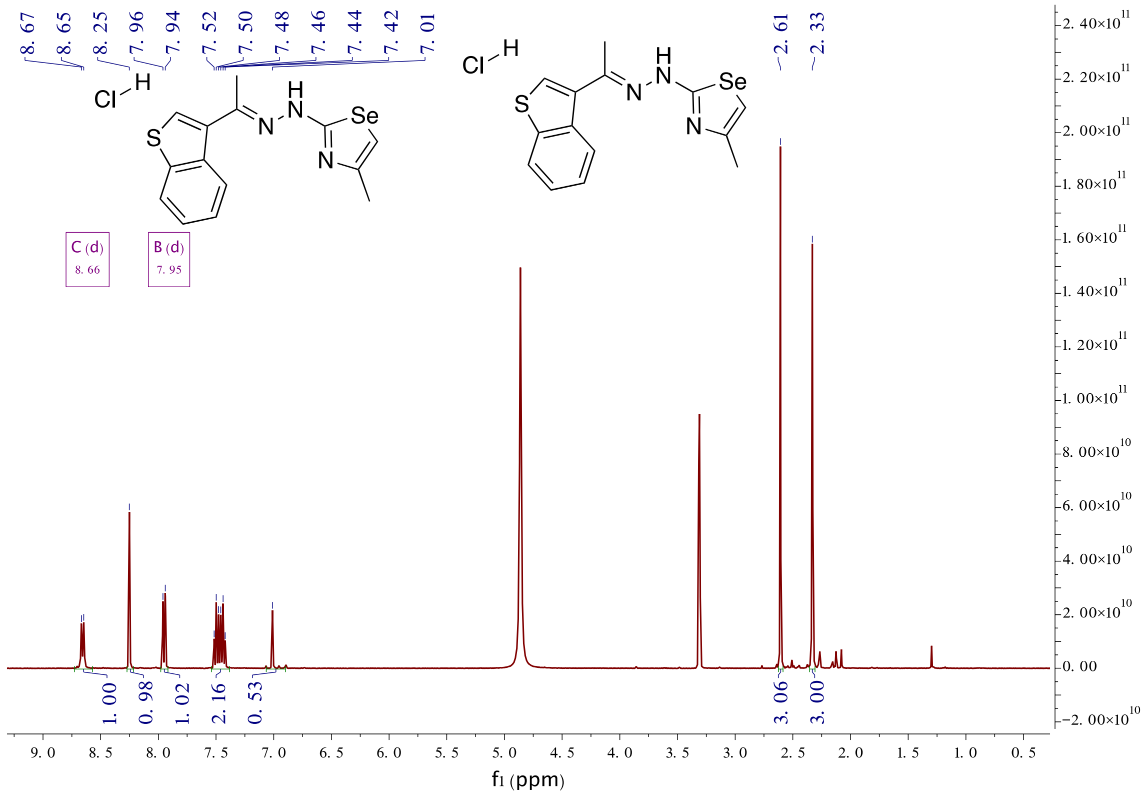

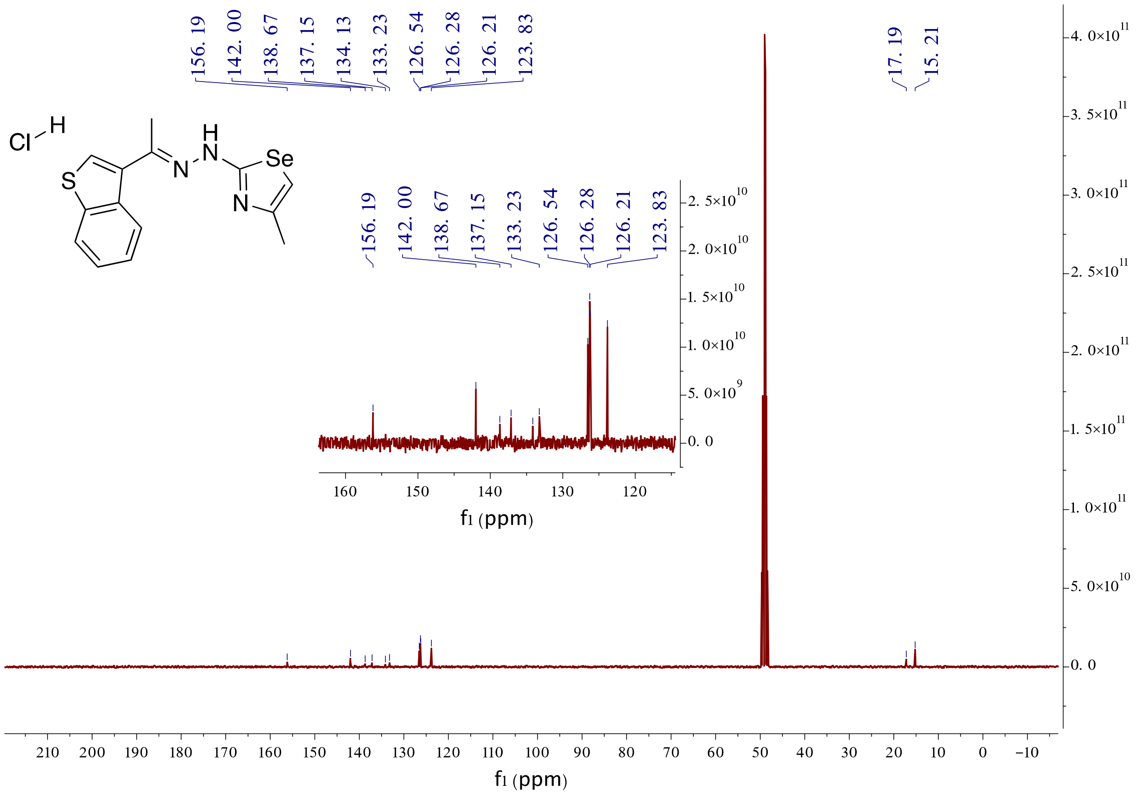

Figure S118. ^{13}C-NMR (up) and qNMR (down) of compound ***Se*2o**.

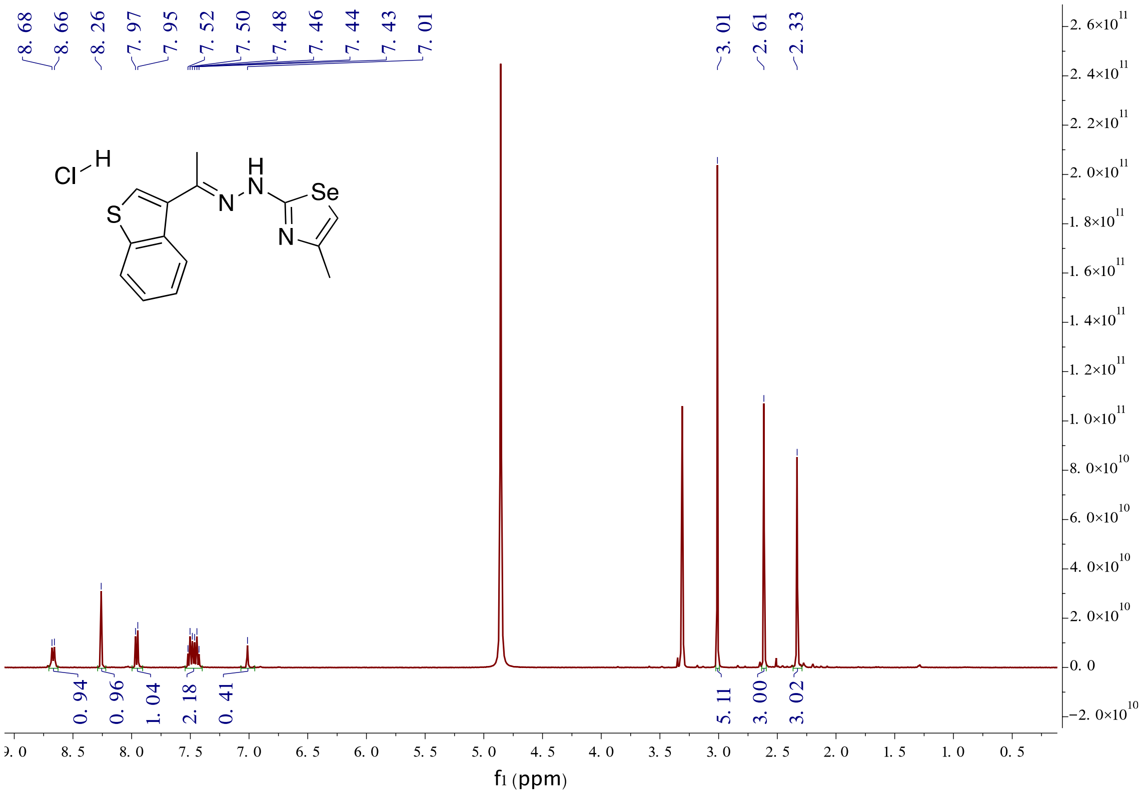

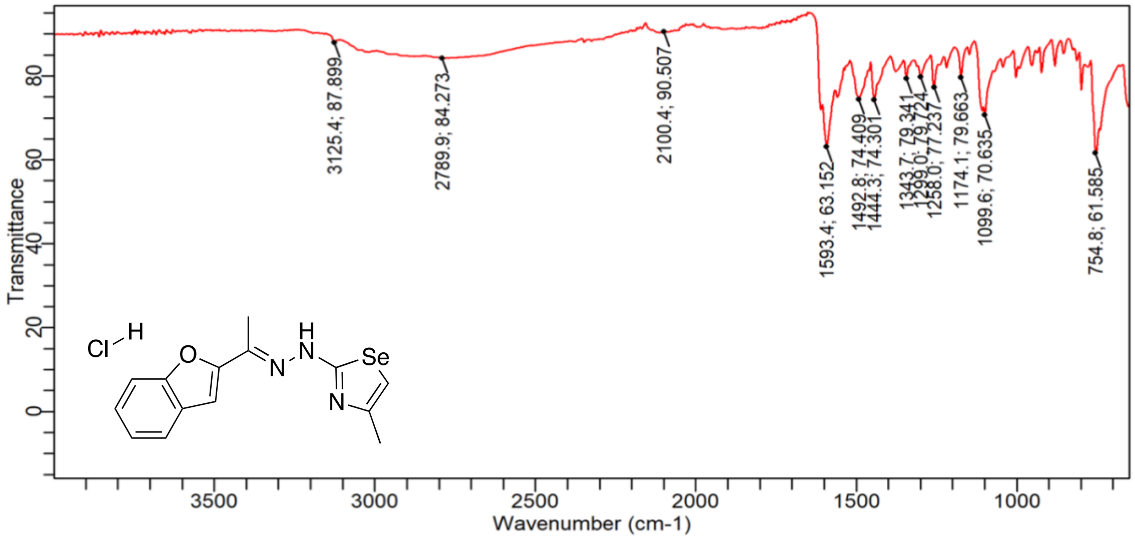

Figure S119. IR (up) and ^{1}H-NMR (down) of compound ***Se*2p**.

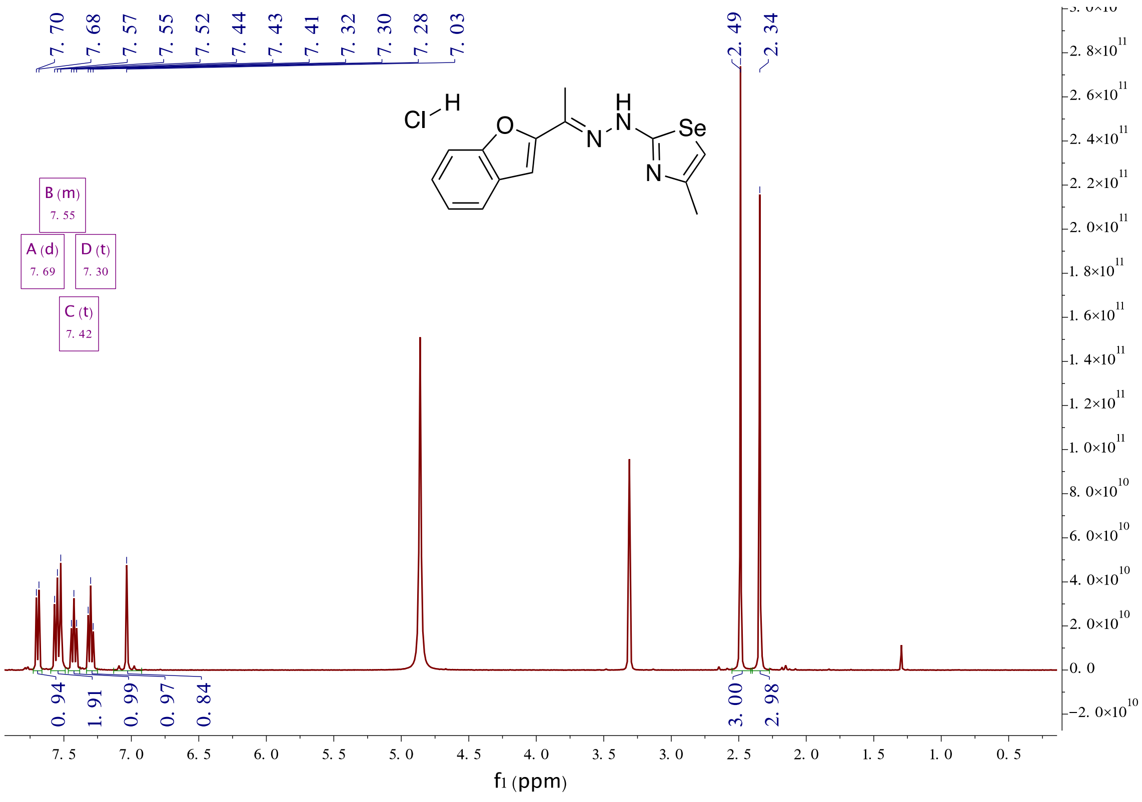

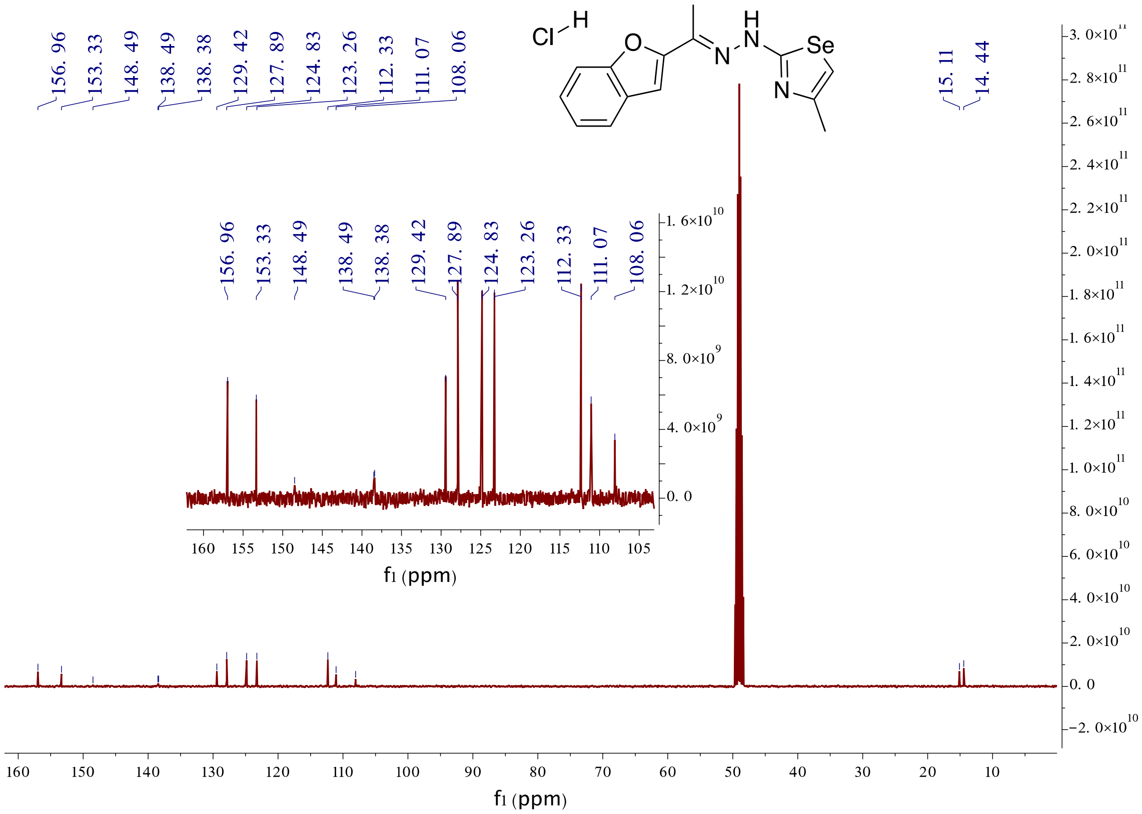

Figure S120. ^{13}C-NMR (up) and qNMR (down) of compound ***Se*2p**.

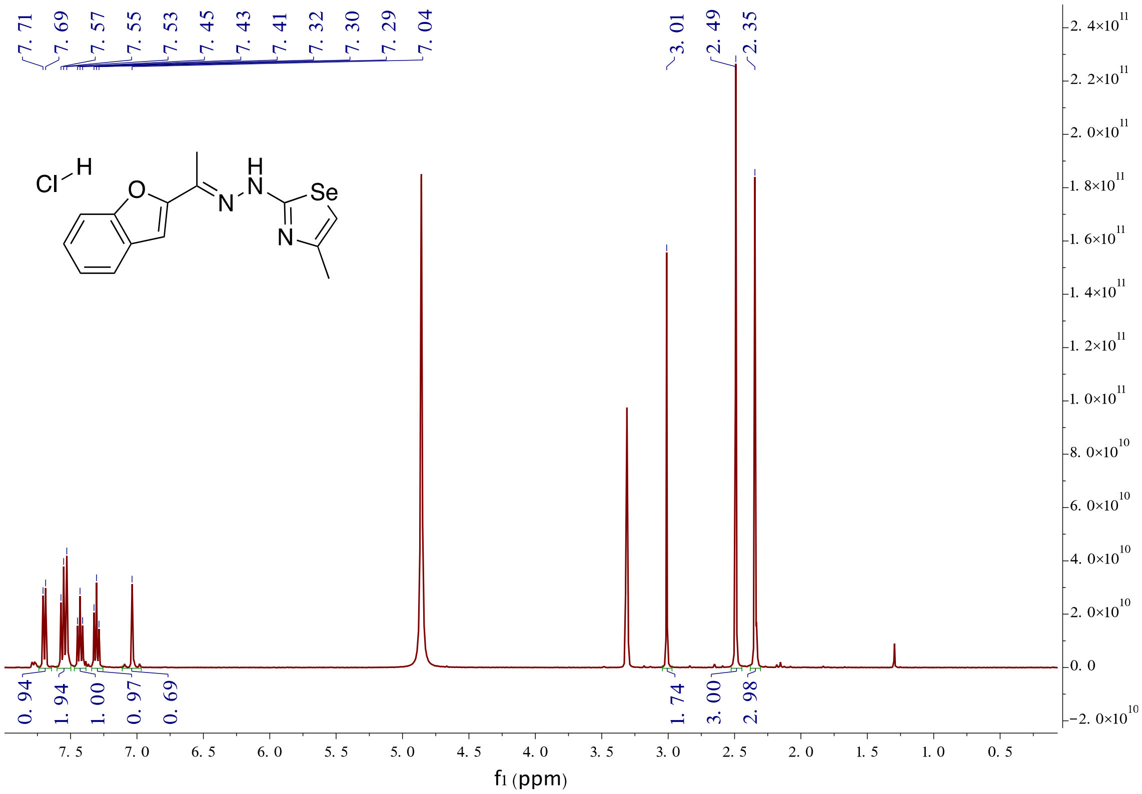

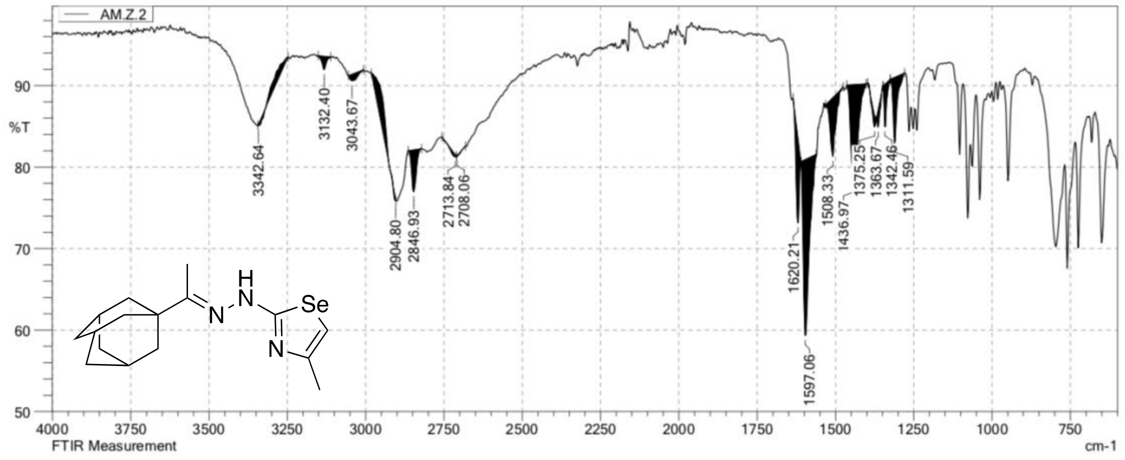

Figure S121. IR (up) and ^{1}H-NMR (down) of compound ***Se*2q**.

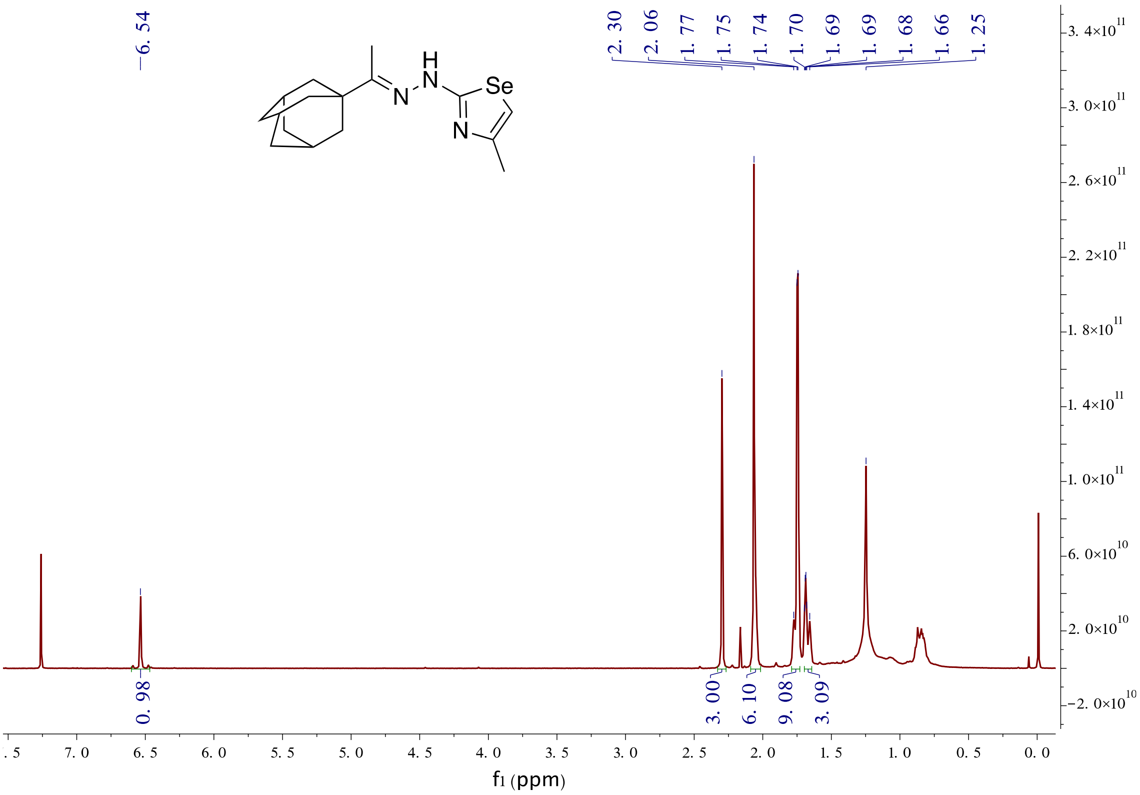

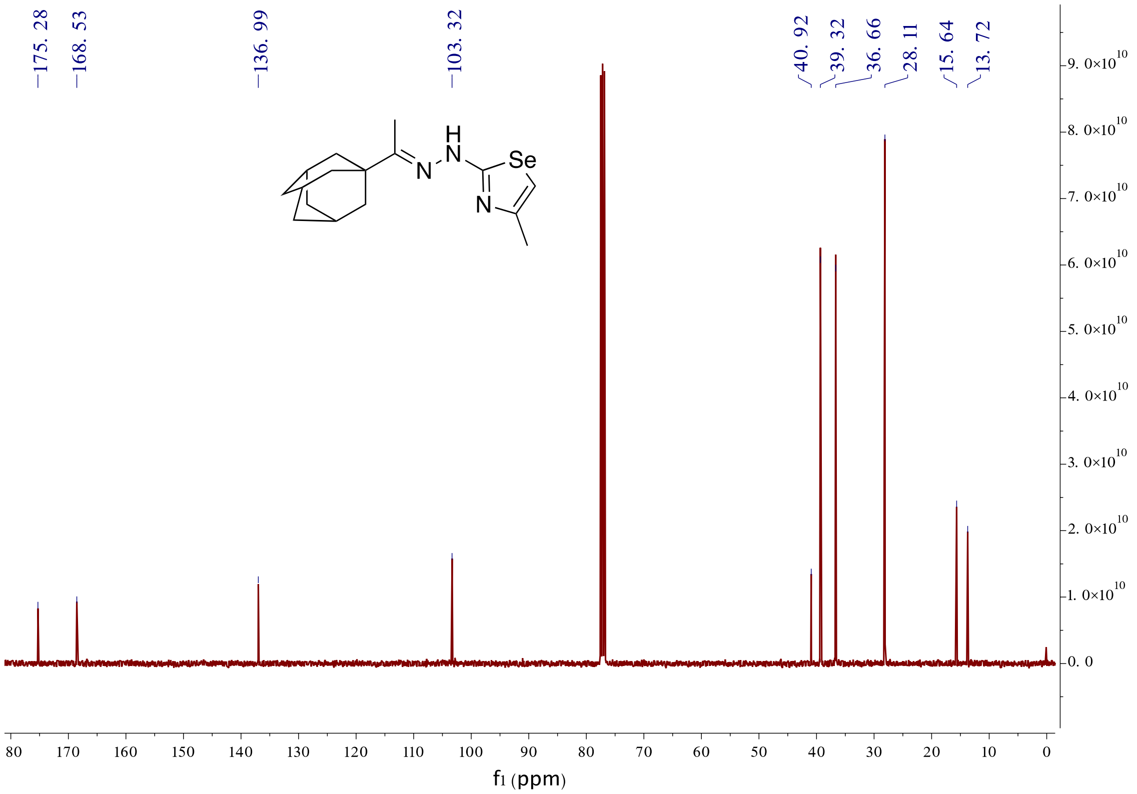

Figure S122. ^{13}C-NMR (up) and qNMR (down) of compound ***Se*2q**.

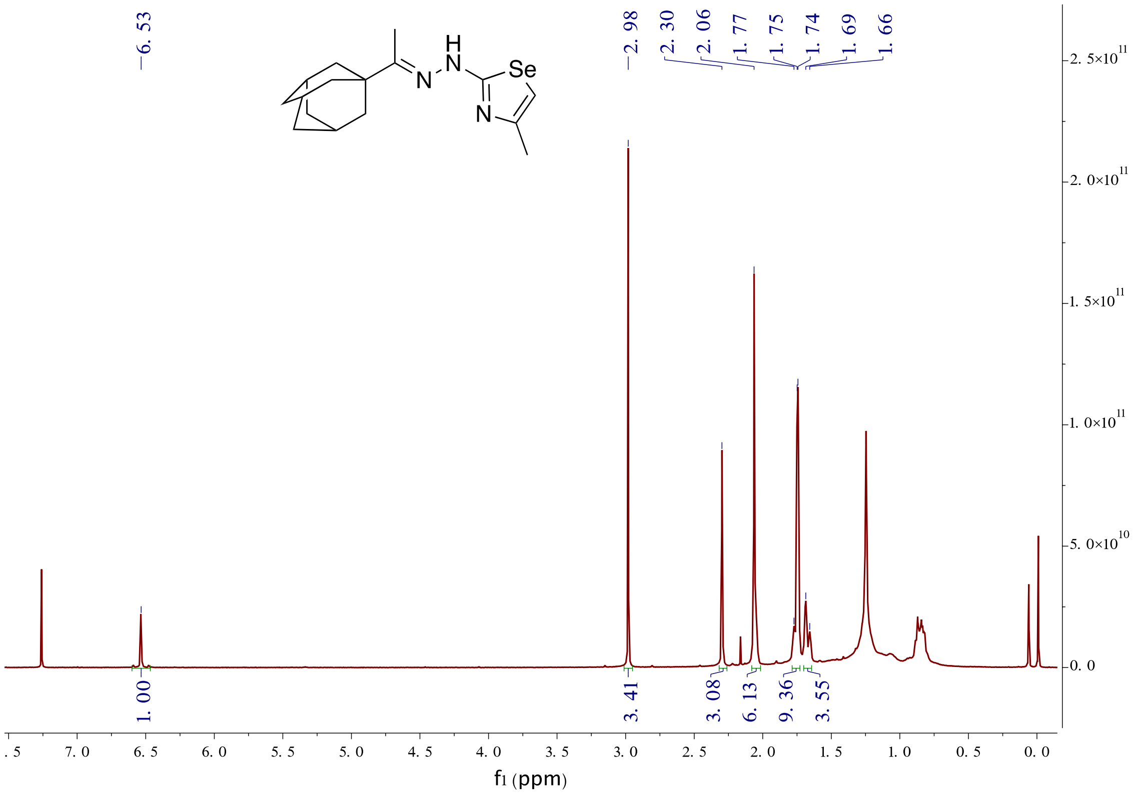

5. Bidimensional spectra of compounds *S*1k and *Se*1k.

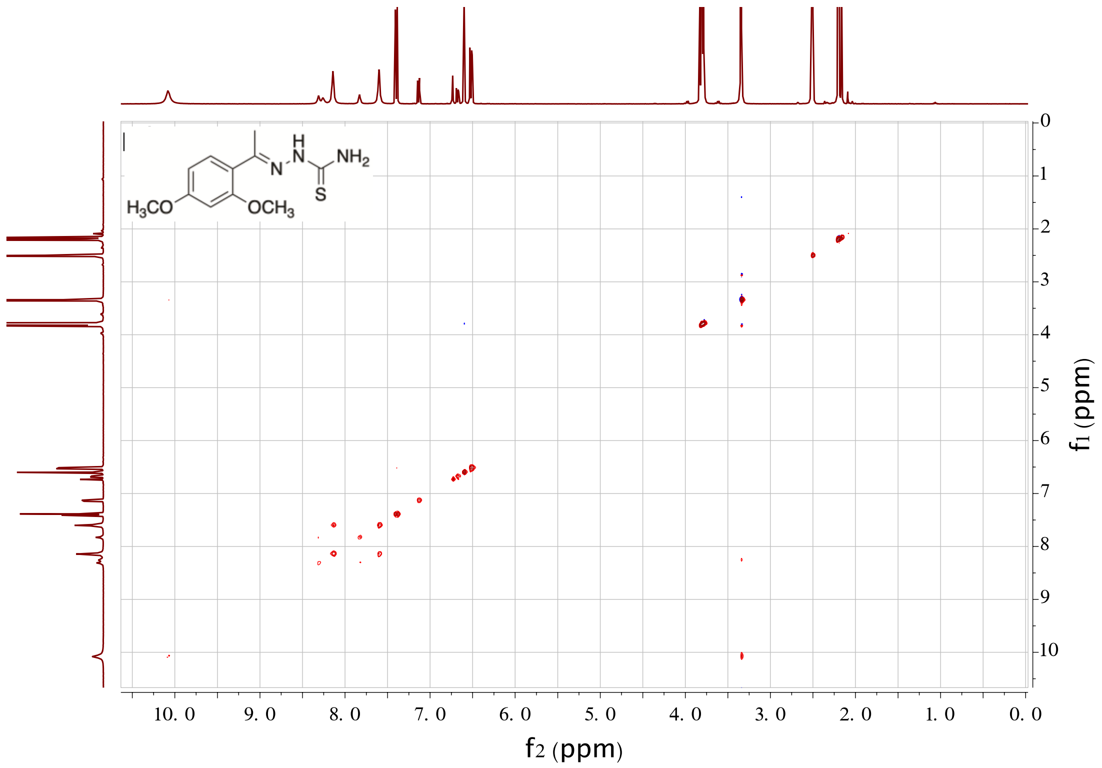

Figure S123. NOESY (up) and HMBC (down) of compound ***S*1k**.

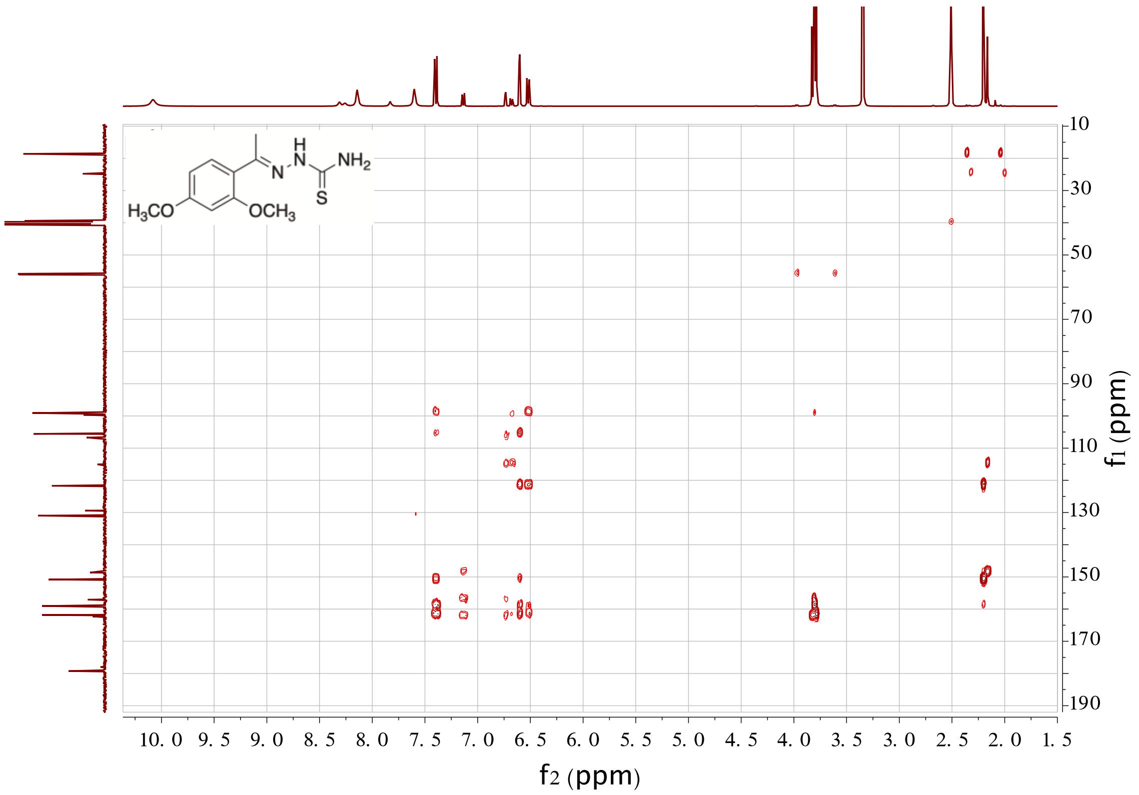

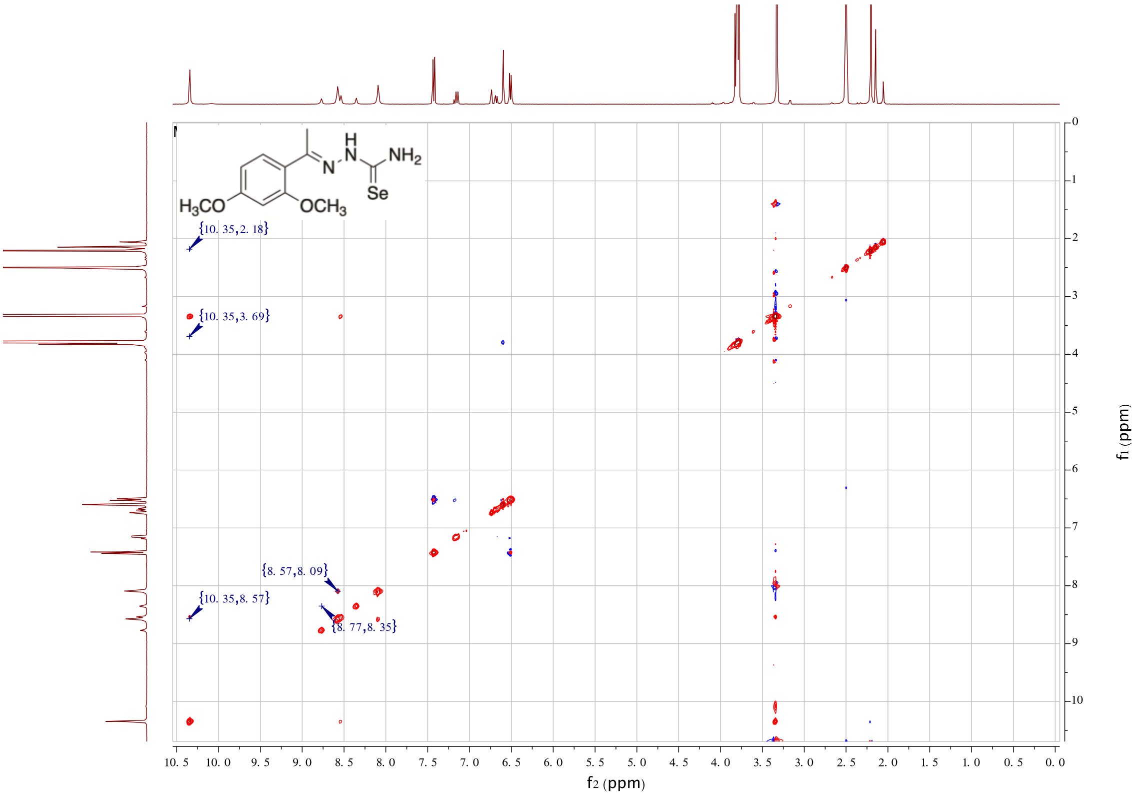

Figure S124. NOESY (up) and HMQC (down) of compound ***Se*1k**.

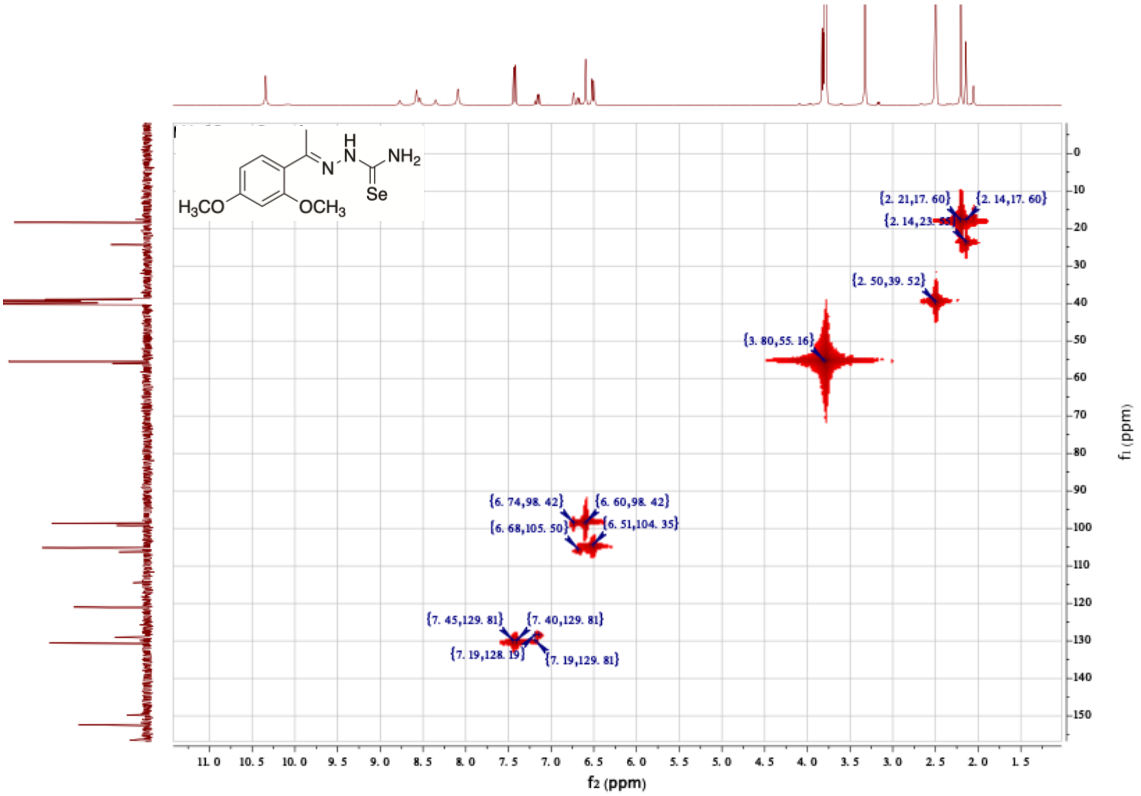

Supplement: Supplementary file 1 [file jm4c01535_si_001.pdf]
